# Genome-wide identification of *MAPKKK* genes and their responses to phytoplasma infection in Chinese jujube (*Ziziphus jujuba* Mill.)

**DOI:** 10.1186/s12864-020-6548-6

**Published:** 2020-02-10

**Authors:** Zhiguo Liu, Lixin Wang, Chaoling Xue, Yuetong Chu, Weilin Gao, Yitong Zhao, Jin Zhao, Mengjun Liu

**Affiliations:** 1 College of Horticulture, Hebei Agricultural University, Baoding, China; 2 Research Center of Chinese Jujube, Hebei Agricultural University, Baoding, China; 3 College of Life Science, Hebei Agricultural University, Baoding, China; 4 Key Laboratory of Hebei Province for Plant Physiology and Molecular Pathology, Hebei Agricultural University, Baoding, Hebei China

**Keywords:** Chinese jujube, *MAPKKKs*, Jujube witches’ broom, Phytoplasma, Expression profiles

## Abstract

**Background:**

Mitogen-activated protein kinase (MAPK) cascades play vital roles in signal transduction in response to a wide range of biotic and abiotic stresses. In a previous study, we identified ten *ZjMAPKs* and five *ZjMAPKKs* in the Chinese jujube genome. We found that some members of *ZjMAPKs* and *ZjMAPKKs* may play key roles in the plant’s response to phytoplasma infection. However, how these *ZjMAPKKs* are modulated by *ZjMAPKKKs* during the response process has not been elucidated. Little information is available regarding *MAPKKKs* in Chinese jujube.

**Results:**

A total of 56 *ZjMAPKKKs* were identified in the jujube genome. All of these kinases contain the key S-TKc (serine/threonine protein kinase) domain, which is distributed among all 12 chromosomes. Phylogenetic analyses show that these *ZjMAPKKKs* can be classified into two subfamilies. Specifically, 41 *ZjMAPKKKs* belong to the Raf subfamily, and 15 belong to the MEKK subfamily. In addition, the *ZjMAPKKKs* in each subfamily share the same conserved motifs and gene structures. Only one pair of *ZjMAPKKKs* (15/16, on chromosome 5) was found to be tandemly duplicated. Using qPCR, the expression profiles of these *MAPKKKs* were investigated in response to infection with phytoplasma. In the three main infected tissues (witches’ broom leaves, phyllody leaves, and apparently normal leaves), *ZjMAPKKK26* and − *45* were significantly upregulated, and *ZjMAPKKK3, − 43* and − *50* were significantly downregulated. *ZjMAPKKK4, − 10, − 25* and − *44* were significantly and highly induced in sterile cultivated tissues infected by phytoplasma, while *ZjMAPKKK6, − 7, − 17, − 18, − 30, − 34, − 35, − 37, − 40, − 41, − 43, − 46, − 52 and − 53* were significantly downregulated.

**Conclusions:**

For the first time, we present an identification and classification analysis of *ZjMAPKKKs.* Some *ZjMAPKKK* genes may play key roles in the response to phytoplasma infection. This study provides an initial understanding of the mechanisms through which *ZjMAPKKKs* are involved in the response of Chinese jujube to phytoplasma infection.

## Background

Mitogen activated protein kinase (MAPK) cascades comprise three specific kinase families: MAP kinase kinase kinases (MAPKKKs), MAP kinase kinases (MAPKKs) and MAP kinases (MAPKs). Essentially, these kinase families are intermediate signalling modules that operate between signal sensing and the activation of related transcription factors. These kinases are involved in plant responses to biotic and abiotic stresses, such as drought, salinity, cold and pathogen attack [1–3]. The conserved serine/threonine MAPKKKs can be activated by plasma membrane receptors which, in turn, phosphorylate MAPKKs, which then activate MAPKs by sequential phosphorylation. Finally, MAPKs regulate other kinases or related transcription factors in response to various stresses [1, 4]. Each MAPK cascade family consists of a number of members, and the number of members varies significantly between families. For example, the MAPKKK family comprises a greater number of members and shows more complex sequence diversity than the other two families. The members belonging to the MAPKKK family can be classified into the subfamilies Raf, ZIK and MEKK according to their characteristic sequence motifs [5]. Structural diversity is found among MAPKKKs in each subfamily. The Raf subfamily has a C-terminal kinase domain and a long N-terminal regulatory domain, while ZIK proteins have only N-terminal kinase domains, and the MEKK subfamily has fewer conserved kinase domains. In addition, a long N-terminal regulatory domain forms the backbone for the Raf and ZIK subfamilies [1, 5].

MAPK cascades have been implicated in signal transduction in distinct innate immunity [6, 7]. In *Arabidopsis*, the *MEKK1-MKK4/5-MPK3/6-WRKY22/WRKY29/FRK1* cascade is involved in innate immunity signal transduction, and the *MEKK1-MKK1/MKK2-MPK4* kinase cascade can negatively activate *MEKK2,* which further leads to SUMM2-mediated immune responses [8, 9]. In tobacco, *NPK1-MEK1-Ntf6* can regulate *WRKY/MYB* transcription factors to participate in the tobacco mosaic virus infection pathway [10]. In addition, the *MAPKKKα-MKK2/MKK4-MPK2/MPK3* cascades participate in the Pto-mediated effect or triggered immunity (ETI) pathway by regulating the transcription factor *TGA* in tomato [11]. Hence, the MAPK pathway is indeed involved in the response to pathogen attack and may also play essential roles in the response to phytoplasma infection in Chinese jujube.

The *MAPKKK* family has now been characterised in the plant kingdom. A total of 80 *MAPKKKs* were first identified in *Arabidopsis* in 2002 [5, 12]. In the following years, an array of different *MAPKKKs* have been identified in a range of plant species, including rice (75 members), *Zea mays* (71 members), *Vitis vinifera* (45 members), *Malus domestica* (72 members) and *Musa nana* (77 members) [13–16]. However, little is known about the biological information and function of the *MAPKKK* gene family in Chinese jujube, even though detailed information for *ZjMAPKKs* and *ZjMAPKs* has previously been reported by our group [17].

Jujube witches’ broom disease (JWB) is caused by ‘*Candidatus Phytoplasma* ziziphi’. JWB is a devastating disease in Asia [18]. Since the 1990s in China and with no effective control methods, JWB disease has severely impacted yields of Chinese jujube [19]. Our group has focused on this disease for many years, and we have published a book, ‘Jujube Witches’ Broom Disease’, which provides detailed information on a number of key questions, including how the phytoplasma infects the plant with a one-year life cycle, how to test for JWB and how to evaluate the severity of JWB. Typical symptoms that can be observed in a plant suffering phytoplasma disease include witches’ broom and phyllody. The physiological and biochemical behaviours of jujube plants infected by this phytoplasma have been widely studied [19–21], but the underlying molecular mechanisms have not been elucidated. Recently, MAPKs have been reported in response to phytoplasma infection of Chinese jujube with analysis of their expression levels in different phytoplasma-infected materials [22]. This study has provided valuable insights into the important roles played by MAPKs during the infection process. In addition, *ZjMPK2*, *ZjMKK2* and *ZjMKK4* have been shown to be the main genes involved in the Chinese jujube-phytoplasma interaction. Additionally, using yeast two-hybrid analyses, it has been demonstrated that ZjMKK2 interacts with ZjMPK2 [17, 23]. All these results demonstrate the important function of MAPK cascades in response to phytoplasma infection in Chinese jujube. However, identification and initial functional analyses of *ZjMAPKKKs* are needed to build our knowledge of the complete MAPK cascade signalling transduction pathway. Thus, the aim of this study was to identify *ZjMAPKKKs* using genome-wide and phylogenetic analyses to predict the gene structure and conserved motifs of *ZjMAPKKKs*. Then, the expression profiles of these *ZjMAPKKKs* in response to phytoplasma infection were investigated by qPCR. The end goal is to expand our understanding of the mechanism through which *ZjMAPKKKs* are involved in the defence responses of Chinese jujube to witches’ broom disease.

## Results

### Genome-wide identification of *ZjMAPKKKs*

A total of 56 *ZjMAPKKKs* were defined. All of these kinases have the key S-TKc (serine/threonine protein kinase) domain and other conserved protein kinase domains (Additional file 2 Table S1). To clearly understand and discriminate between the *MAPKKK* genes, the locus of *ZjMAPKKKs* was designated according to the nomenclature suggestions for *Arabidopsis,* where *Zj* refers to *Ziziphus jujuba*, and the series numbers *ZjMAPKKK1–56* are coded in terms of their chromosome locations (Table 1). The *ZjMAPKKKs* are distributed over all 12 pseudochromosomes, except for *ZjMAPKKK44–56,* which could not be matched to a corresponding chromosome.

**Table 1:** Characteristic of MAPK Kinase Kinases from *Ziziphus jujuba* Mill. (*ZjMAPKKKs*)

Group	Name	Locus ID	Chr	Location	CDS (bp)	Amino acid length (AA)	PI	MW (KD)
	ZjMAPKKK1	LOC107412947	Chr1	512,097–518,184	3351	1116	5.56	123.87
	ZjMAPKKK2	LOC101223021	Chr1	588,730..592155	1062	353	7.16	39.83
	ZjMAPKKK3	LOC107413171	Chr1	6,034,745..6041047	1380	459	8.99	52.10
	ZjMAPKKK4	LOC107414729	Chr1	7,211,380..7216386	1119	372	9.01	42.35
	ZjMAPKKK5	LOC107422643	Chr1	17,322,629..17330021	1707	568	6.55	64.31
	ZjMAPKKK7	LOC107427772	Chr1	28,684,428..28693993	4455	1484	5.27	160.66
	ZjMAPKKK8	LOC107429777	Chr1	31,236,415..31242279	3432	1143	7.67	126.67
	ZjMAPKKK11	LOC107412267	Chr2	23,790,620..23798118	2913	970	5.83	107.05
	ZjMAPKKK13	LOC107415263	Chr4	1,873,602..1877368	1059	352	7.66	39.46
Raf	ZjMAPKKK14	LOC107417160	Chr4	23,620,629..23633413	2208	735	6.1	83.02
	ZjMAPKKK15	LOC107417666	Chr5	2,301,847..2306994	1248	415	8.14	46.31
	ZjMAPKKK16	LOC107417677	Chr5	2,314,642..2318435	1257	418	7.62	46.80
	ZjMAPKKK17	LOC107417907	Chr5	5,814,146..5820584	3789	1262	5.18	139.68
	ZjMAPKKK18	LOC107417903	Chr5	5,822,068..5828809	3825	1274	5.43	140.42
	ZjMAPKKK19	LOC107418405	Chr5	10,268,020..10272429	1074	357	8.97	40.45
	ZjMAPKKK20	LOC107418996	Chr5	18,139,031..18145136	2343	780	6.38	87.04
	ZjMAPKKK22	LOC107421353	Chr6	17,987,031..17997512	2832	943	8.15	105.39
	ZjMAPKKK23	LOC107420099	Chr6	2,889,952..2898479	2856	951	5.53	104.94
	ZjMAPKKK24	LOC107422567	Chr7	14,102,760..14109942	3444	1147	5.87	127.99
	ZjMAPKKK26	LOC107423093	Chr7	21,216,345..21219607	1173	390	7.92	43.53
	ZjMAPKKK27	LOC107423594	Chr7	27,636,786..27641226	1251	416	6.11	46.81
	ZjMAPKKK28	LOC107424157	Chr8	4,344,698..4348053	2049	682	8.81	79.10
	ZjMAPKKK30	LOC107424832	Chr8	9,154,812..9160628	1179	392	9.11	44.23
	ZjMAPKKK32	LOC107426395	Chr9	4,943,575..4946720	1203	400	6.29	44.66
	ZjMAPKKK33	LOC107426719	Chr9	5,733,599..5742140	3945	1314	5.32	144.52
	ZjMAPKKK34	LOC107427400	Chr9	18,534,579..18538786	1032	343	5.84	38.71
	ZjMAPKKK38	LOC107428906	Chr10	10,418,263..10423580	1299	432	8.15	48.84
Raf	ZjMAPKKK39	LOC107428931	Chr10	11,520,310..11525561	1299	432	7.74	48.89
	ZjMAPKKK41	LOC107430036	Chr11	2,650,983..2657196	2181	726	6.97	81.01
	ZjMAPKKK42	LOC107431473	Chr11	19,821,853..19829329	1707	568	5.51	64.02
	ZjMAPKKK43	LOC107432147	Chr12	5,159,272..5168476	2556	851	5.98	93.67
	ZjMAPKKK45	LOC107408109	Unplaced Scaffold	688..6689	1425	474	9.12	53.23
	ZjMAPKKK46	LOC107405634	Unplaced Scaffold	5820..12905	1341	446	5.58	50.42
	ZjMAPKKK47	LOC107407393	Unplaced Scaffold	6471..11148	1125	374	7.13	42.29
	ZjMAPKKK48	LOC107406964	Unplaced Scaffold	13,195..15977	1005	334	7.68	37.91
	ZjMAPKKK49	LOC107404883	Unplaced Scaffold	14,404..17983	1251	416	6.24	46.77
	ZjMAPKKK50	LOC107406505	Unplaced Scaffold	16,102..20361	1059	352	7.17	39.84
	ZjMAPKKK51	LOC107405705	Unplaced Scaffold	33,936..39585	1158	385	7.51	42.93
	ZjMAPKKK52	LOC107403422	Unplaced Scaffold	48,143..64604	1647	548	5.13	61.65
	ZjMAPKKK53	LOC107435406	Unplaced Scaffold	61,008..63977	1479	492	9.34	56.56
	ZjMAPKKK55	LOC107435407	Unplaced Scaffold	134,848..138183	1482	493	9.22	56.35
MEKK	ZjMAPKKK6	LOC107423632	Chr1	18,688,160..18695042	2700	899	9.29	96.87
	ZjMAPKKK9	LOC107432528	Chr1	34,451,996..34453515	1428	475	4.78	53.09
	ZjMAPKKK10	LOC107411974	Chr2	21,809,712..21815908	2046	681	5.64	74.86
	ZjMAPKKK12	LOC107414154	Chr3	19,912,625..19913971	1266	421	4.95	46.85
	ZjMAPKKK21	LOC107420999	Chr6	10,913,503..10919747	1839	612	5.53	67.87
	ZjMAPKKK25	LOC107423026	Chr7	20,770,505..20775489	2058	685	6.78	75.80
	ZjMAPKKK29	LOC107424505	Chr8	6,765,530..6767083	1071	356	4.93	39.97
	ZjMAPKKK31	LOC107425633	Chr8	20,944,047..20949800	1563	520	9.11	56.65
	ZjMAPKKK35	LOC107427543	Chr9	20,198,543..20199481	939	312	6.52	35.52
	ZjMAPKKK36	LOC107428154	Chr10	1,469,136..1470695	762	253	7.01	28.29
	ZjMAPKKK37	LOC107428813	Chr10	8,441,149..8442948	825	274	4.89	30.43
	ZjMAPKKK40	LOC107429056	Chr10	13,522,021..13523553	1035	344	5.36	37.67
	ZjMAPKKK44	LOC107409320	Unplaced Scaffold	335..2009	1335	444	4.8	50.18
	ZjMAPKKK54	LOC107434197	Unplaced Scaffold	108,777..110183	1119	372	5.46	41.39
	ZjMAPKKK56	LOC107435014	Unplaced Scaffold	155,489..160603	1836	611	5.64	67.84

Note: *Chr*: chromosome; *PI*: the theoretical isoelectric point of proteins; *MW*: The theoretical molecular weight of proteins

Specific information for each CDS and amino acid sequence of the *ZjMAPKKKs* is listed in Additional files 1 and 7. Based on the specific conserved signature motif, all the *ZjMAPKKKs* could be grouped into one of the two subfamilies Raf and MEKK. No ZIK subfamily members were identified. As shown in Table 1, the length of the CDS sequences ranged from 762 bp (*ZjMAPKKK36*) to 4455 bp (*ZjMAPKKK7*) with an average length of 1804 bp. The amino acid sequence length of ZjMAPKKKs varied from 253 (*ZjMAPKKK36*) to 1484 (*ZjMAPKKK7*) amino acids (aa); the average length was 600 aa. The predicted molecular weight (Mw) of these proteins ranged from 28.29 (*ZjMAPKKK36*) to 160.66 (*ZjMAPKKK7*), and the theoretical isoelectric points (pI) ranged from 4.78 to 9.34.

### Phylogenetic analyses of *ZjMAPKKK* genes

**Fig. 1:**
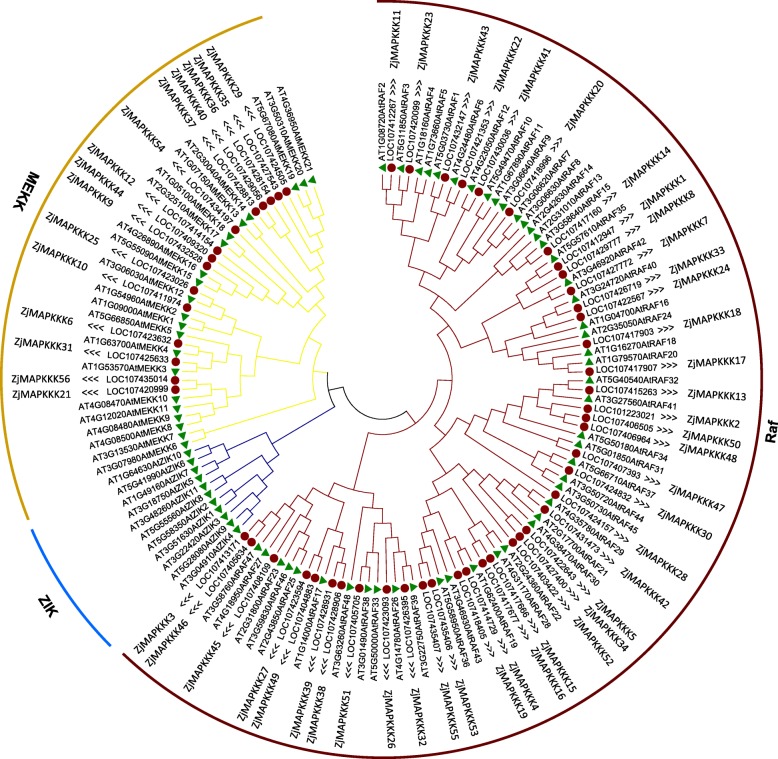
Phylogenetic analyses of ZjMAPKKKs (*Ziziphus jujuba* Mill.) and AtMAPKKKs (*Arabidopsis thaliana*) with a total of 136 protein sequences. MEGA 6.0 was used to construct the phylogenetic tree employing the neighbour-joining (NJ) method. A total of 1000 bootstrap replications were carried out to indicate reliability. The ZjMAPKKKs were clustered into two groups - the Raf and MEKK subfamilies

To assess the phylogenetic relationships between Chinese jujube and *Arobidopsis*, a phylogenetic tree was constructed with all 136 protein sequences (56 ZjMAPKKKs and 80 AtMAPKKKs). As illustrated in Fig. 1, the members of AtMAPKKKs could be clustered into three categories, Raf, ZIK and MEKK, indicating that the method used to build the phylogenetic tree was reliable. However, the 56 members of ZjMAPKKKs could be clustered into only two subfamilies, Raf and MEKK. In addition, the largest Raf subfamily consisted of 41 members, with the remaining 15 members of ZjMAPKKKs belonging to the MEKK subfamily. None could be ascribed to the ZIK subfamily. Moreover, some ZjMAPKKKs located on the same chromosome showed little divergence but clustered into the same group. Examples are ZjMAPKKK36, − 37 and − 40; ZjMAPKKK15 and − 16; and ZjMAPKKK38 and − 39. These results indicate that some duplication of ZjMAPKKKs occurred during the evolution of jujube.

### Conserved domains and gene structure analyses of *ZjMAPKKKs*

**Fig. 2:**
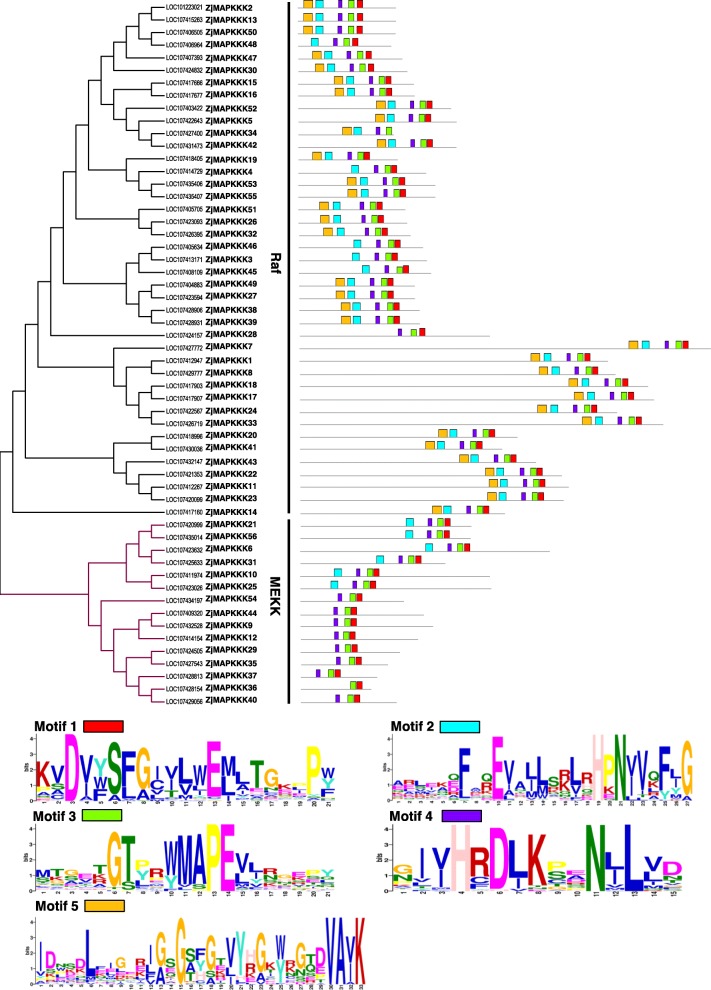
Identification of the conserved motifs of ZjMAPKKKs corresponding to the phylogenetic tree. The MEME database was used to identify the motifs based on protein sequences

Within the analysis of MEME software, five main conserved motifs were identified in all 56 ZjMAPKKKs (Fig. 2). Motifs 1, 3 and 4 were found in all ZjMAPKKKs, while the other two motifs were observed in all Raf subfamily members. The MEKK subfamily members fell into two groups: one contained motifs 1–4, including ZjMAPKKK21, − 56, − 6, − 31, − 10 and − 25*.* The remaining members contained only motifs 1, 3 and 4. These results illustrate that ZjMAPKKKs share the same conserved motifs, which further indicates that the protein structures for each subfamily are highly conserved.

**Fig. 3:**
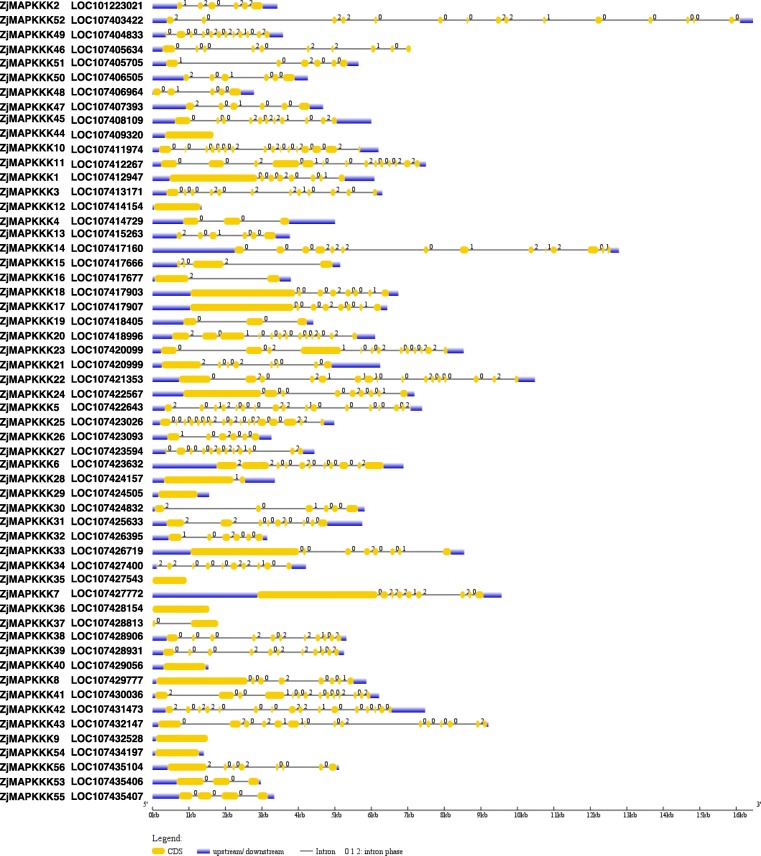
Schematic diagrams of *ZjMAPKKK* structures. The yellow and blue boxes and the black lines indicate exons, UTRs and introns, respectively. The numerals 0, 1 and 2 indicate different intron phases

For the analyses of the exon/intron contents, the differences among *ZjMAPKKKs* were significant. As shown in Fig. 3 and in Additional file 8Table S3, the number of exons in *ZjMAPKKKs* ranged from 1 (*ZjMAPKKK9, − 12, − 29, − 35, − 36, − 44* and *− 54*) to 19 (*ZjMAPKKK42*). Interestingly, the members of *ZjMAPKKKs* containing only 1 exon all belong to the MEKK subfamily (47%). The highest number of exons in this subfamily was 17 (*ZjMAPKKK25* and *− 10*), and the average number was 5.6. This result demonstrates that in this subfamily, significant loss and gains of exons took place during evolution. For the Raf subfamily, the number of exons varied from 2 (*ZjMAPKKK16* and *− 28*) to 19 (*ZjMAPKKK42*) with an average number of 9.56. Although there was significant variation in the number of exons in the Raf and MEKK subfamilies, some exon structure patterns were clearly conserved in close paralogues. For instance, *ZjMAPKKK24* and − *49* have 12 exons, while *ZjMAPKKK37* and − *40* have 2 exons, and they are all closely clustered in the same phylogenetic tree. Collectively, the evolutionarily different organisations of the *ZjMAPKKK* gene structures between the Raf and MEKK subfamilies indicate that tandem and segmental duplication events may have occurred in ancient times and that diverse exon structures may function differently in the jujube genome.

**Fig. 4:**
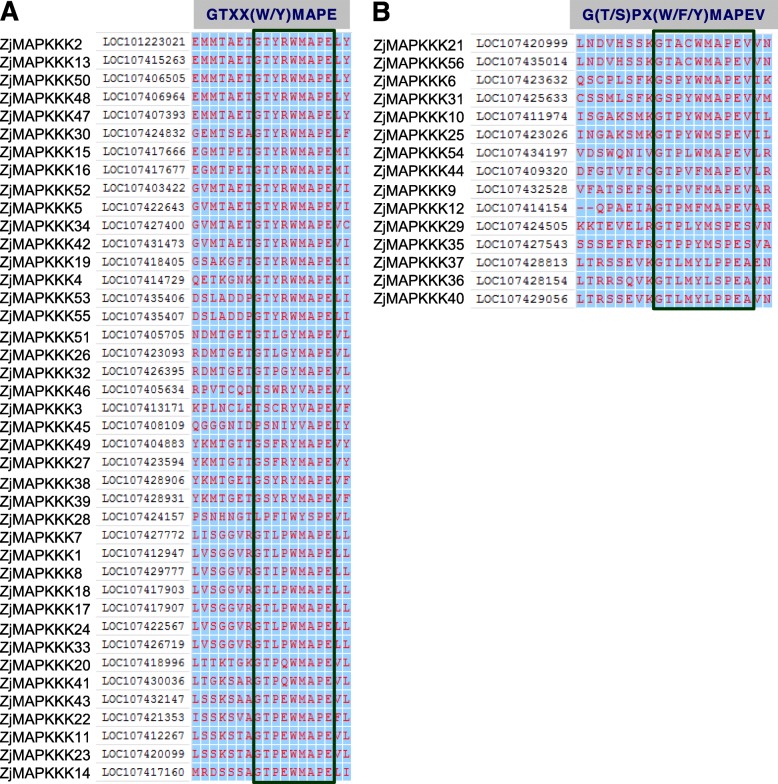
Protein sequence alignment of the Raf subfamily (**a**) and MEKK subfamily (**b**) from *Ziziphus jujuba* Mill. The conserved signature motifs GTXX(W/Y) MAPE and GTPEFMAPE(L/V)(Y/F) were found in the Raf and MEKK subfamilies, respectively

Furthermore, with the multiple protein alignment of *ZjMAPKKKs*, the Raf-specific signature motif GTXX(W/Y) MAPE was found in the Raf subfamily, and the kinase domain was located at the N terminal or C terminal. In contrast, the less conserved MEKK-specific signature motif G(T/S)PX(W/F) MAPEV was observed in the MEKK subfamily, while the kinase domain was located at three positions: the N- or C-terminal or the central part of the proteins (Fig. 4). The features of the signature motifs of ZjMAPKKKs are consistent with other orthologues in other plant species, where they fulfil important roles in a diversity of signal transduction processes.

### Synteny analysis of *ZjMAPKKK* genes

**Fig. 5:**
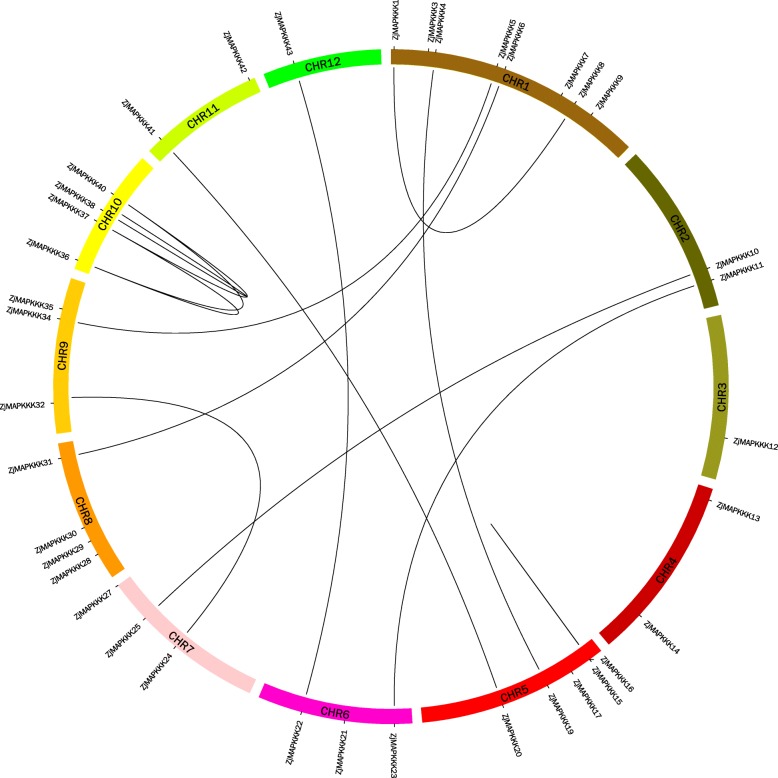
Synteny analysis of *ZjMAPKKK* genes in the Chinese jujube genome. The black lines indicate duplicated *ZjMAPKKK* gene pairs

Tandem duplication events were first analysed according to the principle that two or more genes can be located on a chromosomal region within 200 kb [24] of one another. As shown in Fig. 5, one pair of *ZjMAPKKKs* (15/16) was the only tandem duplication event on LG5. In addition, 13 segmental duplication events with 22 *ZjMAPKKKs* were also identified. These results indicate that some *ZjMAPKKKs* may have been generated by gene duplication and that segmental duplication events were probably a major driving force in *ZjMAPKKK* evolution.

### Phytoplasma detection in different tissues infected by phytoplasma

To characterize the functions of *ZjMAPKKKs* involved in phytoplasma infection, the expression levels of individual *ZjMAPKKKs* were detected by qPCR in two kinds of infected plant material. The first infected plant material was from diseased plants in the field (in vivo). This material showed three levels of symptoms: (a) witches’ broom leaves, (b) phyllody leaves and (c) apparently normal leaves (but from diseased plants). The other infected plant material was from sterile (in vitro) cultured tissues of JWB plantlets. The phytoplasma concentrations in the in vivo material with three levels of symptoms were measured by Xue et al. (2018) [21]. The phytoplasma determination in the in vitro tissues shows fluorescent spots forming a large circle in the phloem of the petiole (Additional file 5Figure S4). These results confirm the subsequent tests on *ZjMAPKKK* function in response to phytoplasma infection.

### Expression analysis of *ZjMAPKKKs* in witches’ broom leaves

**Fig. 6:**
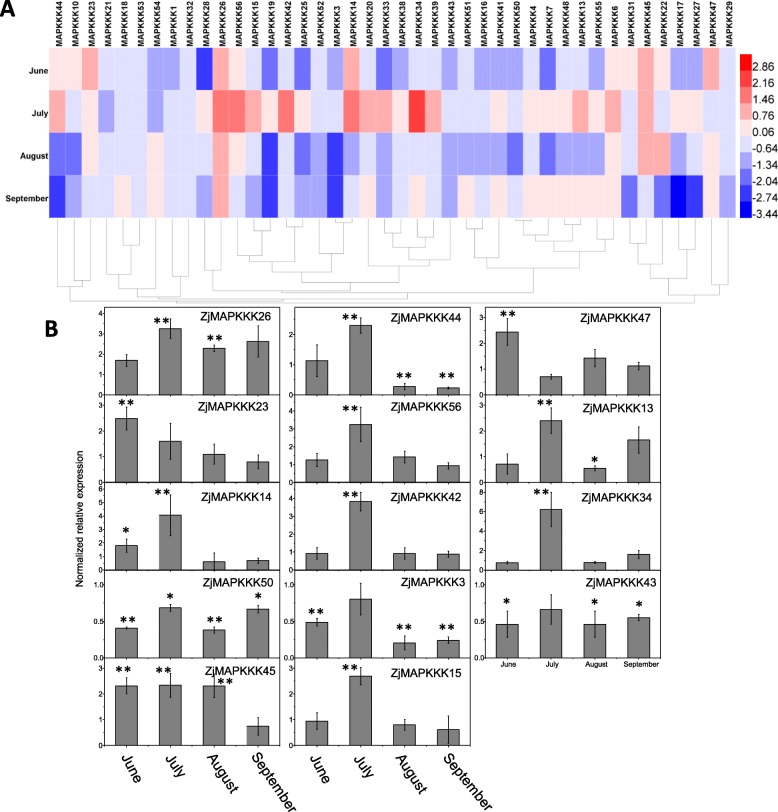
Relative expression profiles of *ZjMAPKKKs* in jujube witch broom leaves from June to September. The expression levels of *ZjMAPKKKs* in the leaves of healthy trees were used as a control. a Heat map analysis of the *ZjMAPKKKs* based on the log2-based fold-change values. b The relative expression levels of the representative members of *ZjMAPKKKs* in three independent replications (the error bar represents standard deviation, SD). Asterisks indicate the corresponding genes significantly up- or downregulated at each time within symptomatic leaves compared with healthy control leaves (*P < 0.05, **P < 0.01)

In Additional file 9 Table S4 and Fig. 6(a), the heat map shows the expression levels of *ZjMAPKKKs* with significantly different patterns in the witches’ broom leaves from June to September. There were 42 candidates expressing at a detectable level, but the expression levels of the other 14 *ZjMAPKKKs* were either not expressed or were expressed at levels below our detection threshold. The *ZjMAPKKKs* genes with too low (or nonexistent) expression were rejected as candidates for further calculation and analysis. Among these genes, the most significant transcript induction took place in the early stage (June or July) when the concentration of witches’ broom began to increase. For example, *ZjMAPKKK13, − 14, − 15, − 23, − 34, − 42, − 44, − 47* and − *56* were significantly induced in June or July, but induction later decreased from August to September, as shown in Fig. 6(b). However, *ZjMAPKKK3, − 43* and − *50* were downregulated from June to September. The expression levels of two *ZjMAPKKK* members (*ZjMAPKKK26* and − *45*) remained high from June to September. However, the clustering of *ZjMAPKKK* expression profiles was not aligned with gene similarities, illustrating that gene function may not rely on gene structure.

### Expression analysis of *ZjMAPKKKs* in phyllody leaves

**Fig. 7:**
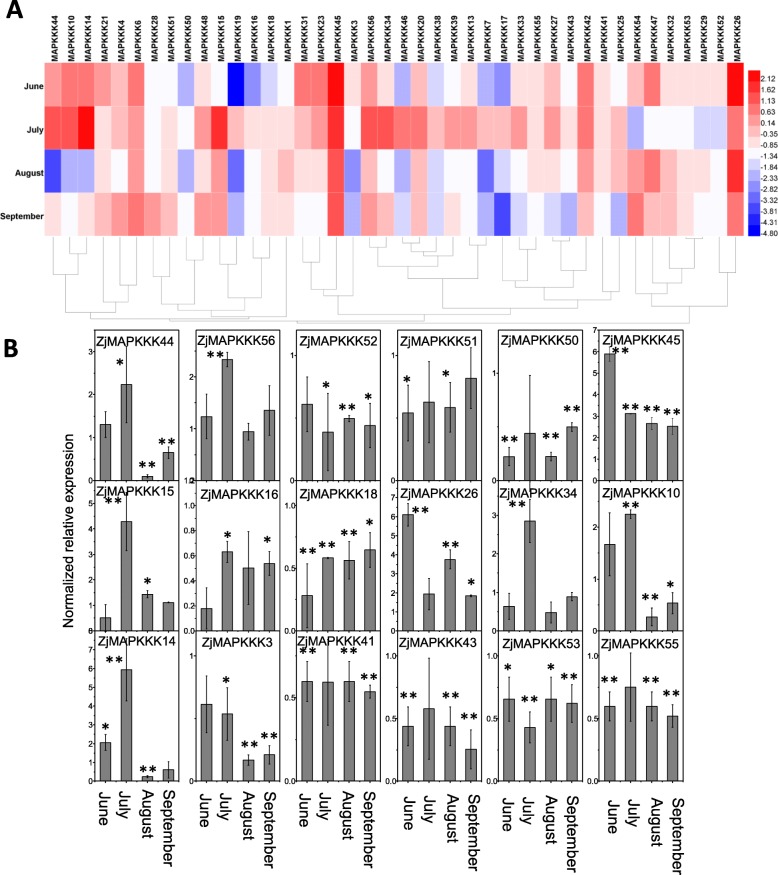
Relative expression profiles of *ZjMAPKKKs* in phyllody leaves from June to September. The expression levels of *ZjMAPKKKs* in the leaves of healthy trees were used as a control. **a** Heat map analysis of the *ZjMAPKKKs* based on the log2-based fold-change values. **b** The relative expression levels of representative members of *ZjMAPKKKs* in three independent replications (the error bar represents standard deviation, SD). Asterisks indicate the corresponding genes significantly up- or downregulated at each time in symptomatic leaves compared with healthy control leaves (*P < 0.05, **P < 0.01)

The transcript abundance of *ZjMAPKKKs* was also investigated in phyllody leaves. The heat map of the expressing *ZjMAPKKKs* is shown in Fig. 7(a). Several of the *ZjMAPKKKs* were highly expressed in June or July, but their expression levels then decreased from August to September. However, most *ZjMAPKKKs* showed no significant changes in expression level. Expression details for all *ZjMAPKKKs* are shown in Fig. 7(b). *ZjMAPKKK10, − 14, − 15 -34, − 44* and *− 56* were all significantly upregulated in the early stage (June or July). However, 10 of the *ZjMAPKKKs* (*ZjMAPKKK3, − 16, − 18, − 41, − 43, − 50, − 51, − 52, − 53* and − *55*) were significantly downregulated. As in the witches’ broom leaves, in the phyllody leaves, *ZjMAPKKK26* and − *45* were highly upregulated from June to September.

### Expression analysis of *ZjMAPKKKs* in apparently normal leaves

**Fig. 8:**
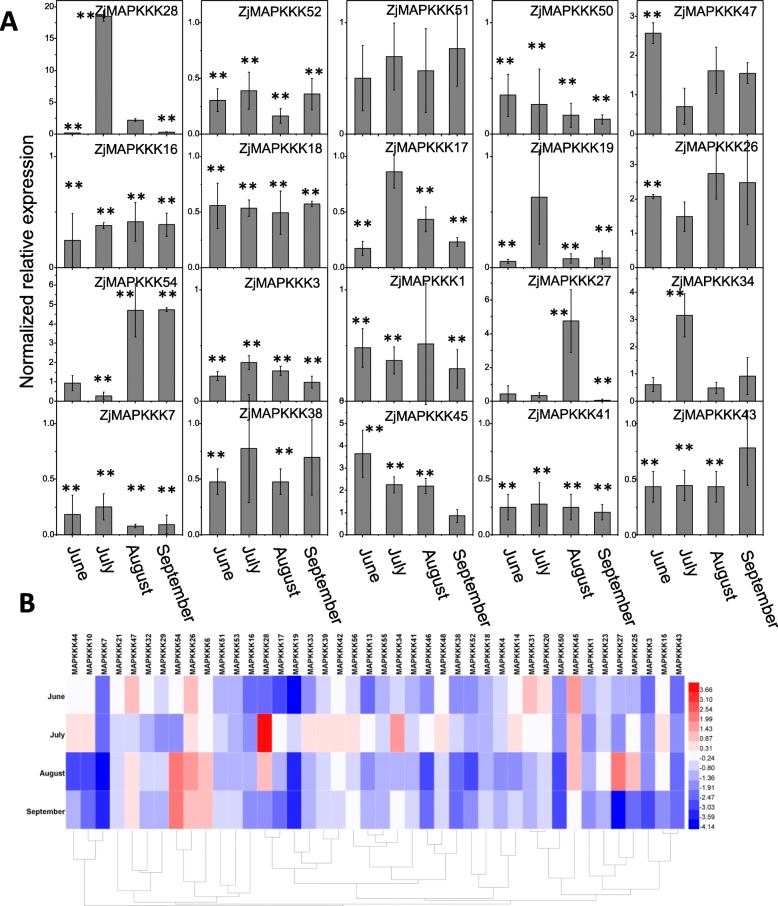
Relative expression profiles of *ZjMAPKKKs* in apparently normal (but infected) leaves from June to September. The expression levels of *ZjMAPKKKs* in the leaves of healthy trees were used as a control. **a** The relative expression levels of representative members of *ZjMAPKKKs* in three independent replications (the error bar represents standard deviation, SD). **b** Heat map analysis of the *ZjMAPKKKs* based on the log2-based fold-change values. Asterisks indicate the corresponding genes significantly up- or downregulated at each time within symptomatic leaves compared with healthy control leaves (*P < 0.05, **P < 0.01)

The apparently normal but asymptomatic infected leaves were used to test which *ZjMAPKKKs* play a role in the phytoplasma infection response. Interestingly, the heat map figure shows different expression patterns for *ZjMAPKKKs* in these leaves (Fig. 8b). A few genes were highly upregulated, but most showed downregulation. For example, *ZjMAPKKK1, − 3, − 7, − 16, − 17, − 18, − 19, − 41, − 43, − 50,* and − *52* were downregulated from June to September, while *ZjMAPKKK28, − 34* and − *47* were significantly upregulated in June or July, while *ZjMAPKKK*27 and − 54 were upregulated from August or September. However, *ZjMAPKKK*26 and − 45 showed the same pattern of high expression in the asymptomatic infected leaves from June to September (Fig. 8a).

To summarise, in the phytoplasma-infected tissues of the three symptomatic severities (apparently normal, phyllody and witches’ broom) and in the 4 months (June through September), *ZjMAPKKK26* was significantly upregulated, and *ZjMAPKKK45* was also highly induced. As the infection developed, the visible disease symptoms increased, becoming gradually more severe, from apparently normal leaves to phyllody leaves to witches’ broom leaves [20]. This progression occurred even though the concentration of JWB decreased gradually from August through September. The expression of *ZjMAPKKK26* increased approximately six-fold in the phyllody leaves in June but not at the other two symptomatic levels. Then, as the infection developed, *ZjMAPKKK26* was upregulated approximately three-fold in the witches’ broom leaves in July but downregulated in the phyllody. Meanwhile, in June, the induction of *ZjMAPKKK45* increased approximately three-fold in the apparently normal (but phytoplasma-infected) leaves and approximately six-fold in the phyllody leaves. Then, during July, August and September, it was downregulated, but induction in the witches’ broom leaves remained approximately constant (Figs. 6, 7 and 8). These results show that *ZjMAPKKK26* responds quickly in phyllody leaves and is highly induced in witches’ broom leaves, while *ZjMAPKKK45* responds more rapidly than *ZjMAPKKK26,* as indicated by its high expression in apparently normal leaves in June. In contrast to *ZjMAPKKK26* and *ZjMAPKKK45,* which were significantly upregulated, *ZjMAPKKK3, ZjMAPKKK43* and *ZjMAPKKK50* were significantly downregulated.

### Expression analysis of the *ZjMAPKKKs* in sterile cultured JWB plantlets

**Fig. 9:**
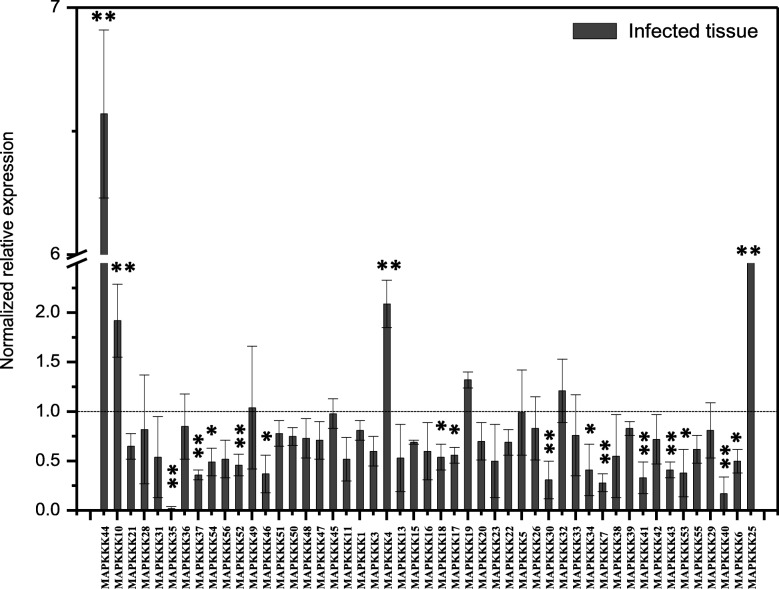
Expression profiles of *ZjMAPKKKs* in phytoplasma diseased plantlets and healthy tissue in in vitro cultured plantlets. Healthy plantlets were used as controls. Three independent replications were carried out (the error bar represents a standard deviation, SD). Asterisks indicate the corresponding genes significantly up- or downregulated at each time within symptomatic leaves compared with healthy control leaves (*P < 0.05, **P < 0.01)

In addition to the *ZjMAPKKK* expression profiles in the in vivo field tissues, we also examined expression levels in the in vitro cultured JWB plantlets, with uninfected plantlets being used as a control. As shown in Fig. 9, the in vitro *ZjMAPKKK* expression profiles differed significantly from the in vivo profiles. Only four of the *ZjMAPKKKs* were significantly induced in the diseased plants - *ZjMAPKKK4, − 10, − 25* and − *44*. *ZjMAPKKK6, − 7, − 17, − 18, − 30, − 34, − 35, − 37, − 40, − 41, − 43, − 46, − 52* and − *53* were significantly downregulated. The other *ZjMAPKKKs* showed no significant change.

## Discussion

The *MAPK* cascades are highly conserved, signal-transducing modules found in eukaryotes. These cascades have been widely studied in the plant kingdom, including in *Arabidopsis*, rice, maize, and apple [5, 9, 12, 13, 15]. Ten *MAPKs* and five *MAPKKs* have already been identified in Chinese jujube [17]. The structures of these genes were mostly shown to be similar to those found in other plants (e.g., *Arabidopsis*, poplar, and apple [17]). In this study, for the first time, we report on *MAPKKKs* in the Chinese jujube genome. We identified 56 *ZjMAPKKKs* in Chinese jujube. This number is somewhat larger than that of *VviMAPKKKs* in grapevine [14] but smaller than that of *MdMAPKKKs* in apple [15]. The *ZjMAPKKKs* can be classified into the two main subfamilies Raf and MEKK, with none belonging to the subfamily ZIK. The disappearance of the ZIK subfamily may be due to a loss of function during the evolution of Chinese jujube because the Raf subfamily is considered to be the origin of the *MAPKKK* family [25]. In addition, the rate of intron loss is known to be more rapid than that of intron gain due to segmental duplication [24]. In this study, we found that the number of conserved motifs and exons was higher in the Raf subfamily than in the MEKK subfamily. We also identified 13 segmental duplication events among 22 *ZjMAPKKKs*. This finding may indicate that the Raf subfamily contains original genes and that segmental duplication occurred during a long evolutionary history. This interpretation is consistent with that for maize [13]. Furthermore, 43 of the *ZjMAPKKKs* were widely located over the 12 chromosomes, while chromosome positions could not be found for *ZjMAPKKK44–56* (Table 1). These findings indicate that evolutionary duplications of *ZjMAPKKKs* took place and that the unknown locations of many *ZjMAPKKKs* may confer a number of paralogous genes and play critical roles in various biological processes. For example, *ZjMAPKKK44, − 45, − 46,* and − *50* may be involved in processes associated with phytoplasma infection. This result is different from that obtained in our previous study on *ZjMAPKs* and *ZjMAPKKs*, which did not show genome duplication in the evolutionary process [17].

MAPK cascades have been shown to be key signalling modules operating in response to biotic and abiotic stresses, particularly associated with pathogen attack [26]. Among the *MAPKKKs*, the MEKK subfamily has been widely studied, while the biological functions of the Raf subfamily are still somewhat obscure. In *Arabidopsis*, the MAPK cascade signalling module *MEKK1-MKK4/MKK5-MPK3/MPK6* is proposed to be activated in the interaction with *flg22* treatment [27]. *EDR1*, a member of the Raf subfamily, is responsible for salicylic acid-induced powdery mildew attack [28]. In tomato, *MAPKKKε* and *MAPKKKα* play important roles in cell death signalling associated with plant immunity [29, 30]. In wheat, a *MAPKKK* named *TaFLR* can be activated by the leaf rust pathogen *Puccinia triticina* [31]*.* In grapevine, *VqMAPKKK38* can be highly induced by powdery mildew infection [32]. All the evidence suggests that members of MAPKKKs are essential in pathogen attack signal transduction. In this study, we demonstrate that several *ZjMAPKKKs*, such as − *26*, − *45*, − *3*, *− 43*, − *50* etc., might play important role in phytoplasma infection in vivo.. Meanwhile, in the in vitro culture of JWB, four *ZjMAPKKKs* (*ZjMAPKKK4, − 10, − 25* and − *44*) were significantly induced in the diseased plants, while 14 *ZjMAPKKKs* (*ZjMAPKKK6, − 7, − 17, − 18, − 30, − 34, − 35, − 37, − 40, − 41, − 43, − 46, − 52* and − *53*) were significantly downregulated. The *ZjMAPKKK* genes response to phytoplasma were different in vivo and in vitro*.* This result is consistent with the findings of our previous work in which we found that *ZjMPK1* was the gene potentially related to phytoplasma infection in vitro [17] but was different from our later finding that *ZjMPK2* was likely regulating along with *ZjMEK2* in vivo [23]. The reason behind this phenomenon may be that many more environmental factors (e.g., light and temperature) affect the development of a phytoplasma infection in vivo. Even so, all the candidates discussed above could be involved in phytoplasma infection signal transduction by recruiting the *MAPKK* and *MAPK* families. Moreover, *ZjMAPKKK43* (homologous to *AtRaf1* in *Arabidopsis*) was downregulated in all the infected tissues, while *ZjMAPKKK10* was highly induced in the in vivo JWB plantlets. This gene is homologous to *AtMEKK1* in *Arabidopsis*. Thus, these two genes may both be key to the response to phytoplasma infection in Chinese jujube because *AtMEKK1* has already been shown to be important in innate immunity in *Arabidopsis* by activating *MKK4/MKK5-MPK3/MPK6* [27]. Moreover, *ZjMKK2* could activate *ZjMPK2* and thus play essential roles in the JWB defence response [23]. Ye et al. [22] have shown that after phytoplasma infection, MAPKs can also be activated and that the transcription factor *WRKY33* is regulated. Taken together, these results indicate the likely *ZjMAPKKKs* that mediate *ZjMKK2-ZjMPK2* to *WRKY* transcription factors in response to phytoplasma infection, which will need to be resolved in future studies. In addition, the scaffold *RACK 1* (Receptor for Activated C Kinase 1) has been identified in *Arabidopsis,* which tethers *MAPKKKs* to the plasma membrane and associates with the Gb subunit involved in immune responses [33]. Therefore, in future studies, it may be worth investigating the functions of these *MAPKKK* candidates and their relationships to *RACK1* in response to phytoplasma infection of Chinese jujube.

## Conclusions

Using a range of informatics analyses of *MAPKKKs* in the Chinese jujube genome, 56 members were identified and named *ZjMAPKKK* according to their locations on the chromosomes. The phylogeny, conserved motifs and intron/exon analyses confirm the identity of these kinases as members of each subfamily. The expression profiles of the *ZjMAPKKKs* were recorded by qPCR in materials at four timings and exhibiting three levels of JWB symptoms (witches’ broom leaves, phyllody leaves and apparently normal leaves) and in sterile in vitro cultures of JWB plantlets. *ZjMAPKKK26* and − *45* were significantly upregulated and *ZjMAPKKK3, − 43* and − *50* were downregulated in the three main infected tissues. Meanwhile, in the sterile cultivated tissues of JWB plantlets, four *ZjMAPKKKs* (*4, 10, 25* and − *44*) were significantly induced in the diseased plants. Additionally, *ZjMAPKKK6, − 7, − 17, − 18, − 30, − 34, − 35, − 37, − 40, − 41, − 43, − 46, − 52* and − *53* were significantly downregulated. Our results provide early insight into certain *ZjMAPKKKs* that could be involved in the plant response to phytoplasma infection.

## Methods

### Identification of *ZjMAPKKKs* in Chinese jujube

The *ZjMAPKKKs* gene family was identified according to our previous study on the identification of *ZjMAPKKs* and *ZjMAPKs* with some modifications [17]. First, the whole protein sequences of *MAPKKKs* in *Arabidopsis* were retrieved from TAIR databases (Additional file 1 Figure S1). These sequences were used as queries to search against the whole jujube genome database (accession JREP00000000) [34]. In addition, the alignments of all *Arabidopsis MAPKKK* sequences were used to construct an HMM profile (http://hmmer.org/download.html) to search for other potential *MAPKKK* members in the jujube genome database. All the potential *ZjMAPKKK* genes that contain a protein kinase domain (PF00069) were confirmed by HMMER tools [35], redundancy was removed, and the remaining sequences were identified as belonging to the *ZjMAPKKK* family. Furthermore, the ExPASy Proteomics Server (http://expasy.org/) was used to calculate the theoretical pI (isoelectric point) and Mw (molecular weight) of the putative *ZjMAPKKKs* [36].

### Gene structure, protein domain and motif analyses of *ZjMAPKKK* genes

The open reading frames (ORFs) of 56 *ZjMAPKKKS* were analysed through the National Center for Biotechnology Information (NCBI, http://www.ncbi.nlm.nih.gov) ORF finder, and the main protein domains of *ZjMAPKKKs* were also recorded by blastp of NCBI **(**Additional file 2Table S1**)**. GSDS (Gene Structure Display Server, http://gsds.cbi.pku.edu.cn/) was used to determine the exon/intron structures of individual *ZjMAPKKKs* by aligning the cDNA sequences with their corresponding genomic DNA sequences [37]. The MEME database was used to identify the conserved motifs of *ZjMAPKKKs* [38]. The ideal motif widths were set to between 6 and 50, and each protein sequence of the *ZjMAPKKKs* subfamily was used to determine the highest number of conserved domains [39].

### Sequence alignment, phylogenetic and gene duplication analysis

All the protein sequences of *ZjMAPKKKs* were aligned by ClusterX software using the default parameter values. The alignments of the protein sequences of 56 *ZjMAPKKKs* and 80 *AtMAPKKKs* were then subjected to phylogenetic analysis using MEGA 6.06 alignment explorer. The parameters of alignment were as follows: gap opening penalty, 10.00; gap extension penalty, 0.50 (both in pairwise alignment and in multiple alignment); protein weight matrix, gonnet; residue-specific penalties, on; hydrophilic penalties, on; gap separation distance, 0; end-gap separation, on; use negative matrix, off; and delay divergent cutoff (%), 30. Phylogenetic trees were constructed by the neighbour-joining (NJ) method. The parameters of the constructed trees were as follows: statistical method, neighbour-joining; scope, all selected taxa; test of phylogeny, bootstrap method; number of bootstrap replications, 1000; substitution types, amino acid; model/method, Poisson model; rates among sites, uniform rates; pattern among lineages, same (homogeneous); and gaps/missing data treatment: complete deletion. The Multiple Collinearity Scan toolkit (MCScanX) was used to analyse the gene duplication events, with the default parameters and the homologous relationships being drawn using Circos software [40, 41].

### Plant materials and treatments

*Ziziphus jujuba* Mill. ‘Dongzao’ was cultivated in the Experimental Station of Chinese Jujube, Hebei Agricultural University and used for experimental material. For each time point, leaf samples were collected from at least three healthy jujube trees and from three trees infected with JWB. All experimental trees were cultivated under natural environmental conditions [19, 21]. Each year, visual symptoms of JWB, such as phyllody leaves (floral organs becoming leaf-like, middle severity) and apparently normal leaves (infected but asymptomatic, or exhibiting minimal symptoms), were first observed in early June. Later, witches’ broom leaves (shoots with small leaves, maximum severity) were observed in the middle of June. Finally, mature witches’ broom leaves, phyllody leaves and apparently normal leaves were collected from the same branch of diseased trees in June, July, August, and September (on the 15th day of each month). The leaves from healthy trees were used as controls for each time point. The use of the above time course is that the phytoplasma content of the branches increased dramatically during the period, reaching a peak in July and August and declining thereafter [19]. The different types of materials are shown in Additional file 3 Figure S2**.** Detection of JWB phytoplasma in the infected materials employed DAPI staining at the histological level and quantitative real-time PCR analysis (qRT-PCR) at the molecular level [21]. Each material was sampled with three replicates, one from each of three different trees. In addition, sterile cultivated JWB diseased plantlets of *Ziziphus jujuba* Mill. ‘Goutouzao’ were used as a parallel testing group, with healthy plantlets being used as controls (as shown in Additional file 4 Figure S3). The presence of JWB phytoplasma in the diseased and healthy sterile cultivated plantlets was detected using DAPI staining at the histological level (Additional file 5 Figure S4) [23]. Ten plantlets were pooled to form one sample, and three independent biological replications were sampled separately. All samples were frozen rapidly in liquid nitrogen and held at − 80 °C pending RNA isolation and qPCR analyses.

### RNA extraction and qRT-PCR analyses

Total RNA was extracted from the leaves using the TIANGEN RNA Extraction Kit. Genomic DNA contamination was removed by digestion with DNase. cDNA synthesis was carried out with the TaKaRa RNA PCR Kit (AMV) Ver. 3.0 (TaKaRa) according to the manufacturer’s protocol using 1 μg of RNA template.

qRT-PCR was carried out on the Bio-Rad iQ™ 5 using TransStart Top Green qPCR SuperMix AQ131 (TransGen Biotech, China). The 20-μL reaction system contained 10 μL of 2 × SYBR Premix ExTaq™, 0.4 μL each of 10 μM primers, 1 μL diluted cDNA and 8.2 μL ddH_2_O. The thermal profile was preincubated for 3 min at 94 °C followed by 40 cycles of 5 s at 94 °C, 15 s at 55~63 °C and 15 s at 72 °C. Relative expression levels of *ZjMAPKKKs* were calculated by the 2^-ΔΔCt^ method [40] using *ZjActin* as an endogenous control for normalisation [41]. The primer sequences of *ZjMAPKKKs* for qPCR are shown in Additional file 6 Table S2.

### Heatmap construction

The expression profiles of all *ZjMAPKKKs* in the different samples are illustrated by a colour gradient heatmap. The heatmap was constructed by heatmap software Heml 1.0 using log2-based expression fold changes.

### Statistical analyses

All data were analysed by the two-sample t-test method using Origin 8.0 software with range tests (*P* < 0.05 and *P* < 0.01).

## Supplementary information

**Additional file 1: Figure S1.** Protein sequences of MAPKKKs from *Ziziphus jujuba* Mill. and *Arabidopsis thaliana*.

**Additional file 2: Table S1.** Number of main protein domains of ZjMAPKKKs*.*

**Additional file 3: Figure S2.** Healthy and diseased in vitro plantlets. A: Healthy plantlets; B: Diseased plantlets.

**Additional file 4: Figure S3.** Tissues showing different JWB disease symptoms. A: Witches’ broom leaves; B: Phyllody leaves; C: Apparently normal leaves; D: Healthy leaves. A, B and C were used as a test group collected from diseased trees. D was used as a control collected from healthy trees.

**Additional file 5: Figure S4.** Determination of phytoplasma in the sieve element in jujube petiole phloem by using 4′,6-diamidino-2-phenylindole (DAPI). A, No fluorescent spots were observed in the sieve element (SE) of healthy plantlets. B, The fluorescent spots formed a large, bright circle in the sieve element (SE) of the diseased plantlets. The numbers and sizes of the fluorescent spots indicate the number of phytoplasmas. Bar = 100 μm.

**Additional file 6: Table S2.** Primer sequences of *ZjMAPKKKs* for qRT-PCR.

**Additional file 7: Figure S5.** CDS sequences of *ZjMAPKKKs.*

**Additional file 8: Table S3.** Number of introns and exons of *ZjMAPKKK* genes

**Additional file 9: Table S4.** Fold-change values of *ZjMAPKKKs* in the leaves of witches’ broom, phyllody and apparently normal symptoms.

## Data Availability

All data and materials are presented in the main paper and additional file. In addition, the whole protein sequences of MAPKKKs in *Arabidopsis* were retrieved from TAIR databases. The CDS and genome sequences of MAPKKKs in jujube were retrieved from the whole jujube genome database (accession JREP00000000) in NCBI.

## Abbreviations

Chr: Chromosome.
JWB: Jujube witches’ broom
MAPK: Mitogen-activated protein kinase
MAPKK: MAPK kinase
MAPKKK: MAPK kinase kinase
MW: The theoretical molecular weight of proteins.
ORF: Open reading frames.
PI: The theoretical isoelectric point of proteins.
qPCR: Quantitative real-time PCR.
RACK 1: Receptor for Activated C Kinase 1.
S-TKc: Serine/threonine protein kinase
*Zj*: *Ziziphus jujuba.*
