# Supplementary material for: Genome-wide identification of MAPKKK genes and their responses to phytoplasma infection in Chinese jujube (Ziziphus jujuba Mill.)
Source: BMC Genomics. 2020 Feb 10;21:142. doi: 10.1186/s12864-020-6548-6 (PMC7011567; doi:10.1186/s12864-020-6548-6)
Supplement: Supplementary file 1 — Additional file 1: Figure S1. Protein sequences of MAPKKKs from Ziziphus jujuba Mill. and Arabidopsis thaliana. [file 12864_2020_6548_MOESM1_ESM.pdf]

>AT1G09000AtMEKK1
MQDFFGSVRRSLVFRPSSDDDNQENQPPFPGVLADKITSCIRKSKIFIKPSFSPPPPANTVDMAPPISWRKGQLI
GRGAFGTVYMGMNLDSGELLAVKQVLIAANFASKEKTQAHIQELEEEVKLLKNLSHPNIVRYLGTVREDDTLNIL
LEFVPGGSISSLLEKFGPFPESVVRTYTRQLLLGLEYLHNHAIMHRDIKGANILVDNKGCIKLADFGASKQVAELAT
MTGAKSMKGTPYWMAPEVILQTGHSFSADIWSVGCTVIEMVTGKAPWSQQYKEVAAIFFIGTTKSHPPIPDTL
SSDAKDFLLKCLQEVPNLRPTASELLKHPFVMGKHKESASTDLGSVLNNLSTPLPLQINNTKSTPDSTCDDVGD
MCNFGSLNYSLVDPVKSIQNKNLWQQNDNGGDEDDMCLIDDENFLTFDGEMSSTLEKDCHLKKSCDDISDM
SIALKSKFDESPGNGEKESTMSMECDQPSYSEDDDELTESKIKAFLDEKAADLKKLQTPLYEEFYNSLITFSPSCME
SNLSNSKREDTARGFLKLPPKSRSPSRGPLGGSPSRATDATSCSKSPGSGGSRELNINNGGDEASQDGVSARVT
DWRGLVVDTKQELSQCVALSEIEKKWKEELDQELERKRQEIMRQAGLGSSPRDRGMSRQREKSRFASPGK

>AT1G54960AtMEKK2
MVFAKSQSPPNNSTVQIKPPIRWRKGQLIGRGAFGTVYMGMNLDSGELLAVKQVLITSNCASKEKTQAHIQEL
EEEVKLLKNLSHPNIVRYLGTVREDETLNILLEFVPGGSISSLLEKFGAFPESVVRTYTNQLLLGLEYLHNHAIMHR
DIKGANILVDNQGCIKLADFGASKQVAELATISGAKSMKGTPYWMAPEVILQTGHSFSADIWSVGCTVIEMVTG
KAPWSQQYKEIAAIFHIGTTKSHPPIPDNISSDANDFLLKCLQQEPNLRPTASELLKHPFVTGKQKESASKDLTSF
MDNSCSPLPSELTNITSYQTSTSDDVGDICNLGSLTCTLAFPEKSIQNNSLCLKSNNGYDDDDDNDMCLIDDENF
LTYNGETGPSLDNNTDAKKSCDTMSEISDILKCKFDENSGNGETETKVSMEVDHPSYSEDENELTESKIKAFLDD
KAAELKKLQTPLYEEFYNGMITCSPICMESNINNNKREEAPRGFLKLPPKSRSPSQGHIGRSPSRATDAACCSKSP
ESGNSSGAPKNSNASAGAEQESNSQSVALSEIERKWKEELDQELERKRREITRQAGMGSSPRDRSLSRHREKSR
FASPGK

>AT1G53570AtMEKK3
MPTWWGRKSCKNKDDNHRGIISTDRDIKSSAVVVDPPLTPTRGGTPRCSREFAGASSAFSGFDSDSTEKKGHP
LPRPLLSPVSIHHQDHVSGSTSGSTSVSSVSSSGSADDQSQLVASRGRGDVKFNVAAAPRSPERVSPKAATITTR
PTSPRHQRLSGVVSLESSTGRNDDGRSSSECHPLPRPPTSPTSPSAVHGSRIGGGYETSPSGFSTWKKGKFLGSG
TFGQVYLGFNSEKGKMCAIKEVKVISDDQTSKECLKQLNQEINLLNQLCHPNIVQYYGSELSEETLSVYLEYVSGG
SIHKLLKDYGSFTEPVIQNYTRQILAGLAYLHGRNTVHRDIKGANILVDPNGEIKLADFGMAKHVTAFSTMLSFKG
SPYWMAPEVVMSQNGYTHAVDIWSLGCTILEMATSKPPWSQFEGVAAIFKIGNSKDTPEIPDHLSNDAKNFIR
LCLQRNPTVRPTASQLLEHPFLRNTTRVASTSLPKDFPPRSYDGNFSLQPTREPYPGRLSHDNYAKQPLSRTIKSP
SRENVRAITSLPVSPCSSPLRQLGPAYKSCFLSPPHPSYAFPGQDSGYNLAEFAASPFRMKKDAMMEPSSFRTQT
PNSPLRSRLV

>AT1G63700AtMEKK4
MPWWSKSKDEKKKTNKESIIDAFNRKLGFASEDRSSGRSRKSRRRRDEIVSERGAISRLPSRSPSPSTRVSRCQSF
AERSPAVPLPRPIVRPHVTSTDSGMNGSQRPGLDANLKPSWLPLPKPHGATSIPDNTGAEPDFATASVSSGSSV
GDIPSDSLLSPLASDCENGNRTPVNISSRDQSMHSNKNSAEMFKPVPNKNRILSASPRRRPLGTHVKNLQIPQR
DLVLCSAPDSLLSSPSRSPMRSFIPDQVSNHGLLISKPYSDVSLLGSGQCSSPGSGYNSGNNSIGGDMATQLFWP
QSRCSPECSPVPSPRMTSPGPSSRIQSGAVTPLHPRAGGSTTGSPTRRLDDNRQQSHRLPLPPLLISNTCPFSPT
YSAATSPSVPRSPARAEATVSPGSRWKKGRLLGMGSFGHVYLGFNSESGEMCAMKEVTLCSDDPKSRESAQQL
GQEISVLSRLRHQNIVQYYGSETVDDKLYIYLEYVSGGSIYKLLQEYGQFGENAIRNYTQQILSGLAYLHAKNTVH
RDIKGANILVDPHGRVKVADFGMAKHITAQSGPLSFKGSPYWMAPEVIKNSNGSNLAVDIWSLGCTVLEMATT
KPPWSQYEGVPAMFKIGNSKELPDIPDHLSEEGKDFVRKCLQRNPANRPTAAQLLDHAFVRNVMPMERPIVS
GEPAEAMNVASSTMRSLDIGHARSLPCLDSEDATNYQQKGLKHGSGFSISQSPRNMSCPISPVGSPIFHSHSPHI

SGRRSPSPISSPHALSGSSTPLTGCGGAIPFHHQRQTTVNFLHEGIGSSRSPGSGGNFYTNSFFQEPSRQQDRSR
SSPRTPPHVFWDNNGSIQPGYNWNKDNQPVLSDHVSQQLLSEHLKLKSLDLRPGFSTPGSTNRGP

>AT5G66850AtMEKK5
MRWLPQISFSSPSSSPSSSLKPVASYSESPDPDRNQDRDRFHRRLFRFNRGRLTRQRKLRHLTDDDVLLGERRAS
TSSSTFDSGLTRSPSAFTAVPRSPSAVPLPLPLPLPEVAGIRNAANARGLDDRDRDPERLISDRTSSGPPLTSVNGG
FARDSRKATENSSYQDFSPRNRNGYWVNIPTMSAPTSPYMSPVPSPQRKSTGHDLPFFYLPPKSNQAWSAPD
MPLDTSGLPPPAFYDITAFSTDNSPIHSPQPRSPRKQIRSPQPSRPSSPLHSVDSSAPPRDSVSSPLHPRLSTDVT
NGRRDCCNVHPLPLPPGATCSSSSAASVPSPQAPLKLDSFPMNSQWKKGKLIGRGTFGSVYVASNSETGALCA
MKEVELFPDDPKSAECIKQLEQEIKLLSNLQHPNIVQYFGSETVEDRFFIYLEYVHPGSINKYIRDHCGTMTESVV
RNFTRHILSGLAYLHNKKTVHRDIKGANLLVDASGVVKLADFGMAKHLTGQRADLSLKGSPYWMAPELMQAV
MQKDSNPDLAFAVDIWSLGCTIIEMFTGKPPWSEFEGAAAMFKVMRDSPPIPESMSPEGKDFLRLCFQRNPA
ERPTASMLLEHRFLKNSLQPTSPSNSDVSQLFNGMNITEPSSRREKPNFKLDQVPRARNMTSSESESGQQQQQ
QQYRSPDLTGTVNRLSPRSTLEAIPSPCPSQRPKPSSSDRRRTGVTSDHL

>AT3G07980AtMEKK6
MARQMTSSQFHKSKTLDNKYMLGDEIGKGAYGRVYIGLDLENGDFVAIKQVSLENIGQEDLNTIMQEIDLLKNL
NHKNIVKYLGSLKTKTHLHIILEYVENGSLANIIKPNKFGPFPESLVTVYIAQVLEGLVYLHEQGVIHRDIKGANILTT
KEGLVKLADFGVATKLNEADFNTHSVVGTPYWMAPEVIELSGVCAASDIWSVGCTIIELLTCVPPYYDLQPMPAL
YRIVQDDTPPIPDSLSPDITDFLRLCFKKDSRQRPDAKTLLSHPWIRNSRRALRSSLRHSGTIRYMKETDSSSEKD
AEGSQEVVESVSAEKVEVTKTNSKSKLPVIGGASFRSEKDQSSPSDLGEEGTDSEDDINSDQGPTLSMHDKSSR
QSGTCSISSDAKGTSQDVLENHEKYDRDEIPGNLETEASEGRRNTLATKLVGKEYSIQSSHSFSQKGEDGLRKAVK
TPSSFGGNELTRFSDPPGDASLHDLFHPLDKVPEGKTNEASTSTPTANVNQGDSPVADGGKNDLATKLRARIAQ
KQMEGETGHSQDGGDLFRLMMGVLKDDVLNIDDLVFDEKVPPENLFPLQAVEFSRLVSSLRPDESEDAIVTSSL
KLVAMFRQRPGQKAVFVTQNGFLPLMDLLDIPKSRVICAVLQLINEIVKDNTDFLENACLVGLIPLVMSFAGFER
DRSREIRKEAAYFLQQLCQSSPLTLQMFISCRGIPVLVGFLEADYAKHREMVHLAIDGMWQVFKLKKSTSRNDF
CRIAAKNGILLRLVNTLYSLSEATRLASISGDALILDGQTPRARSGQLDPNNPIFSQRETSPSVIDHPDGLKTRNGG
GEEPSHALTSNSQSSDVHQPDALHPDGDRPRLSSVVADATEDVIQQHRISLSANRTSTDKLQKLAEGASNGFPV
TQPDQVRPLLSLLEKEPPSRKISGQLDYVKHIAGIERHESRLPLLYASDEKKTNGDLEFIMAEFAEVSGRGKENGN
LDTAPRYSSKTMTKKVMAIERVASTCGIASQTASGVLSGSGVLNARPGSTTSSGLLAHALSADVSMDYLEKVADL
LLEFARAETTVKSYMCSQSLLSRLFQMFNRVEPPILLKILECTNHLSTDPNCLENLQRADAIKQLIPNLELKEGPLV
YQIHHEVLSALFNLCKINKRRQEQAAENGIIPHLMLFVMSDSPLKQYALPLLCDMAHASRNSREQLRAHGGLDV
YLSLLDDEYWSVIALDSIAVCLAQDVDQKVEQAFLKKDAIQKLVNFFQNCPERHFVHILEPFLKIITKSSSINKTLAL
NGLTPLLIARLDHQDAIARLNLLKLIKAVYEKHPKPKQLIVENDLPQKLQNLIEERRDGQRSGGQVLVKQMATSLL
KALHINTIL

>AT3G13530AtMEKK7
MARQMTSSQFHKSKTLDNKYMLGDEIGKGAYGRVYKGLDLENGDFVAIKQVSLENIVQEDLNTIMQEIDLLKN
LNHKNIVKYLGSSKTKTHLHIILEYVENGSLANIIKPNKFGPFPESLVAVYIAQVLEGLVYLHEQGVIHRDIKGANILT
TKEGLVKLADFGVATKLNEADVNTHSVVGTPYWMAPEVIEMSGVCAASDIWSVGCTVIELLTCVPPYYDLQPM
PALFRIVQDDNPPIPDSLSPDITDFLRQCFKKDSRQRPDAKTLLSHPWIRNSRRALQSSLRHSGTIKYMKEATASS
EKDDEGSQDAAESLSGENVGISKTDSKSKLPLVGVSSFRSEKDQSTPSDLGEEGTDNSEDDIMSDQVPTLSIHEK
SSDAKGTPQDVSDFHGKSERGETPENLVTETSEARKNTSAIKHVGKELSIPVDQTSHSFGRKGEERGIRKAVKTP
SSVSGNELARFSDPPGDASLHDLFHPLDKVSEGKPNEASTSMPTSNVNQGDSPVADGGKNDLATKLRATIAQK

QMEGETGHSNDGGDLFRLMMGVLKDDVIDIDGLVFDEKVPAENLFPLQAVEFSRLVSSLRPDESEDAIVSSCQK
LVAMFRQRPEQKVVFVTQHGFLPLMDLLDIPKSRVICAVLQLINEIIKDNTDFQENACLVGLIPVVMSFAGPERD
RSREIRKEAAYFLQQLCQSSPLTLQMFIACRGIPVLVGFLEADYAKYREMVHLAIDGMWQVFKLKRSTPRNDFCR
IAAKNGILLRLINTLYSLNEATRLASISGGLDGQAPRVRSGQLDPNNPIFGQNETSSLSMIDQPDVLKTRHGGGE
EPSHASTSNSQRSDVHQPDALHPDGDKPRVSSVAPDASTSGTEDVRQQHRISLSANRTSTDKLQKLAEGASNG
FPVTQTEQVRPLLSLLDKEPPSRHYSGQLDYVKHITGIERHESRLPLLHGSNEKKNNGDLDFLMAEFAEVSGRGK
ENGSLDTTTRYPSKTMTKKVLAIEGVASTSGIASQTASGVLSGSGVLNARPGSATSSGLLAHMVSTLSADVAREY
LEKVADLLLEFARADTTVKSYMCSQSLLSRLFQMFNRVEPPILLKILECTNHLSTDPNCLENLQRADAIKHLIPNLE
LKDGHLVYQIHHEVLSALFNLCKINKRRQEQAAENGIIPHLMLFIMSDSPLKQYALPLLCDMAHASRNSREQLRA
HGGLDVYLSLLDDEYWSVIALDSIAVCLAQDNDNRKVEQALLKQDAIQKLVDFFQSCPERHFVHILEPFLKIITKS
YRINKTLAVNGLTPLLISRLDHQDAIARLNLLKLIKAVYEHHPRPKQLIVENDLPQKLQNLIEERRDGQRSGGQVL
VKQMATSLLKALHINTIL

>AT4G08500AtMEKK8
MDRILARMKKSTGRRGGDKNITPVRRLERRDAARNINYDAASCSSSSAEDLSVSTSSLMTRSLEFPEPTSFRIGG
GVGEMDRIYRSLGVSGPDDLAISFDAWEACKKRSSSDVVNRFKSFDLDKVRDQDLSEEGPSGVVVGSDSMNH
KVQGQDLSEAGPSGGIVTELSEIGNLITPVDRLVADGVVENRRVMERTPTIVKSKGYLVPNNVVAVGVGVGGGI
KGLRPPVLKPPPAMKRPPIDHRGSSWDFLTHFAPSETVKRPSSSSSSSEDGCDEEEGKEEEAEAEEMGARFIQLG
DTADETCSFTTNEGDSSSTVSNTSPIYPDGGAIITSWQKGQLLGRGSFGSVYEGISGDGDFFAVKEVSLLDQGSQ
AQECIQQLEGEIKLLSQLQHQNIVRYRGTAKDGSNLYIFLELVTQGSLLKLYQRYQLRDSVVSLYTRQILDGLKYLH
DKGFIHRDIKCANILVDANGAVKLADFGLAKVSKFNDIKSCKGTPFWMAPEVINRKDSDGYGSPADIWSLGCTV
LEMCTGQIPYSDLEPVQALFRIGRGTLPEVPDTLSLDARLFILKCLKVNPEERPTAAELLNHPFVRRPLPSVGSGGS
GSASPLLRR

>AT4G08480AtMEKK9
MKKSSDKSPVRQHDTATQINSDAVSSSTSFTDSDSTCSFLTPSMEFPDRISFRRIDFSEAAPTGVVLPSTSSELTRS
NSSENKIPNEDISVSTSSRYLVFDKILALMKKSPGRRGDKTSPARRLDRSDAVRRNIDYDAGEDSSSLLITRSLDFP
NRTSFRVDGVDDGEIDRIYQYIGVSGPEDFAISSDAWKARMEHERSSSDVVNKLKSLDLDSREAGPSGGVVASS
SMNHKFQGHDLSEAGSIGVVVASNFTLSESNKIENLNSLRDKEIVDGDMVENRCGIERKPTILVKSRGYLVHND
DVGVGGGIKGVRPPVLNVPRADKEVVDGGTVESKSGIEWKPTILVKSKGYLVSNDGGIKGVTSPVLNLRPTDKE
VVDSGTVENRRGIKGVRPSVLKPPPVMKLPPVDLPGSSWDILTHFAPDSEIVRRPSSSSSSENGCDEEEAEDDKV
EKEETGDMFIQLEDTTDEACSFTTNEGDSSSTVSNTSPICVSGGSINTSWQKGQLLRQGSFGSVYEAISEDGDFF
AVKEVSLLDQGSQAQECIQQLEGEIALLSQLEHQNILRYRGTDKDGSNLYIFLELVTQGSLLELYRRYQIRDSLISLY
TKQILDGLKYLHHKGFIHRDIKCATILVDANGTVKLADFGLAKVSKLNDIKSRKETLFWMAPEVINRKDNDGYRSP
ADIWSLGCTVLEMCTGQIPYSDLEPVEALFRIRRGTLPEVPDTLSLDARHFILKCLKLNPEERPTATELLNHPFVRR
PLPSSGSGSTSPLIRR

>AT4G08470AtMEKK10
MDVTAIFAGDILVQSREYLIPNDVVDVDGGIKAVRPPIIQPPPGRKLPLIDFPGSSWDFLTYFAPSKTVKRQSSSSS
DNTSDKEEVETEETRGMFVQLGDTAHEACPFATNEADSSSTVSIISPSYASRGSIVPSWLKRKFLGRVSLGFVYEG
SSGSSVGSESTCSLMTPSLEFPDRISFRKKDFSEKGPSRHVWEKRKLTRAKLIENFCNPEDIEPVTSWLKGQLLGE
ESFASVYEAISDSSVGSESTCSLMTPSMEFPDRISFRKRDFSEEGPSGRVKEKRKLMRNKLIENFRKPEDITSWLK
GQLLGRGSYASVYEAISEDGDFFAVKEVSLLDKGIQAQECIQQLEGEIALLSQLQHQNIVRYRGTAKDVSKLYIFLE
LVTQGSVQKLYERYQLSYTVVSLYTRQILAGLNYLHDKGFVHRDIKCANMLVDANGTVKLADFGLAEASKFNDI

MSCKGTLFWMAPEVINRKDSDGNGSPADIWSLGCTVLEMCTGQIPYSDLKPIQAAFKIGRGTLPDVPDTLSLD
ARHFILTCLKVNPEERPTAAELLHHPFVINL

>AT4G12020AtMEKK11
MSEKEELPLTLTSIGAATATSDYHQRVGSSGEGISSSSSDVDPRFMQNSPTGLMISQSSSMCTVPPGMAATPPIS
SGSGLSQQLNNSSSSKLCQVEGCQKGARDASGRCISHGGGRRCQKPDCQKGAEGKTVYCKAHGGGRRCEYLG
CTKGAEGSTDFCIAHGGGRRCNHEDCTRSAWGRTEFCVKHGGGARCKTYGCGKSASGPLPFCRAHGGGKKCS
HEDCTGFARGRSGLCLMHGGGKRCQRENCTKSAEGLSGLCISHGGGRRCQSIGCTKGAKGSKMFCKACITKRP
LTIDGGGNMGGVTTGDALNYLKAVKDKFEDSEKYDTFLEVLNDCKHQGVDTSGVIARLKDLFKGHDDLLLGFN
TYLSKEYQITILPEDDFPIDFLDKVEGPYEMTYQQAQTVQANANMQPQTEYPSSSAVQSFSSGQPQIPTSAPDS
SLLAKSNTSGITIIEHMSQQPLNVDKQVNDGYNWQKYGQKKVKGSKFPLSYYKCTYLGCPSKRKVERSLDGQVA
EIVYKDRHNHEPPNQGKDGSTTYLSGSSTHINCMSSELTASQFSSNKTKIEQQEAASLATTIEYMSEASDNEEDS
NGETSEGEKDEDEPEPKRRITEVQVSELADASDRTVREPRVIFQTTSEVDNLDDGYRWRKYGQKVVKGNPYPR
FSSSKDYDVVIRYGRADISNEDFISHLRASLCRRGISVYEKFNEVDALPKCRVLIIVLTSTYVPSNLLNILEHQHTEDR
VVYPIFYRLSPYDFVCNSKNYERFYLQDEPKKWQAALKEITQMPGYTLTDKSESELIDEIVRDALKVLCSADKVN
MIGMDMQVEEILSLLCIESLDVRSIGIWGTVGIGKTTIAEEIFRKISVQYETCVVLKDLHKEVEVKGHDAVRENFLS
EVLEVEPHVIRISDIKTSFLRSRLQRKRILVILDDVNDYRDVDTFLGTLNYFGPGSRIIMTSRNRRVFVLCKIDHVYE
VKPLDIPKSLLLLDRGTCQIVLSPEVYKTLSLELVKFSNGNPQVLQFLSSIDREWNKLSQEVKTTSPIYIPGIFEKSCC
GLDDNERGIFLDIACFFNRIDKDNVAMLLDGCGFSAHVGFRGLVDKSLLTISQHNLVDMLSFIQATGREIVRQES
ADRPGDRSRLWNADYIRHVFINDTGTSAIEGIFLDMLNLKFDANPNVFEKMCNLRLLKLYCSKAEEKHGVSFPQ
GLEYLPSKLRLLHWEYYPLSSLPKSFNPENLVELNLPSSCAKKLWKGKKARFCTTNSSLEKLKKMRLSYSDQLTKIP
RLSSATNLEHIDLEGCNSLLSLSQSISYLKKLVFLNLKGCSKLENIPSMVDLESLEVLNLSGCSKLGNFPEISPNVKEL
YMGGTMIQEIPSSIKNLVLLEKLDLENSRHLKNLPTSIYKLKHLETLNLSGCISLERFPDSSRRMKCLRFLDLSRTDI
KELPSSISYLTALDELLFVDSRRNSPVVTNPNANSTELMPSESSKLEILGTPADNEVVVGGTVEKTRGIERTPTILVK
SREYLIPDDVVAVGGDIKGLRPPVLQLQPAMKLSHIPRGSTWDFVTHFAPPETVAPPSSSSEAREEEVETEETGA
MFIPLGDKETCSFTVNKGDSSRTISNTSPIYASEGSFITCWQKGQLLGRGSLGSVYEGISADGDFFAFKEVSLLDQ
GSQAHEWIQQVEGGIALLSQLQHQNIVRYRGTTKDESNLYIFLELVTQGSLRKLYQRNQLGDSVVSLYTRQILDG
LKYLHDKGFIHRNIKCANVLVDANGTVKLADFGLAKVMSLWRTPYWNWMAPEVILNPKDYDGYGTPADIWSL
GCTVLEMLTGQIPYSDLEIGTALYNIGTGKLPKIPDILSLDARDFILTCLKVNPEERPTAAELLNHPFVNMPLPSSGS
GSVSSLLRG

>AT3G06030AtMEKK12
MQDILGSVRRSLVFRSSLAGDDGTSGGGLSGFVGKINSSIRSSRIGLFSKPPPGLPAPRKEEAPSIRWRKGELIGC
GAFGRVYMGMNLDSGELLAIKQVLIAPSSASKEKTQGHIRELEEEVQLLKNLSHPNIVRYLGTVRESDSLNILME
FVPGGSISSLLEKFGSFPEPVIIMYTKQLLLGLEYLHNNGIMHRDIKGANILVDNKGCIRLADFGASKKVVELATVN
GAKSMKGTPYWMAPEVILQTGHSFSADIWSVGCTVIEMATGKPPWSEQYQQFAAVLHIGRTKAHPPIPEDLSP
EAKDFLMKCLHKEPSLRLSATELLQHPFVTGKRQEPYPAYRNSLTECGNPITTQGMNVRSSINSLIRRSTCSGLKD
VCELGSLRSSIIYPQKSNNSGFGWRDGDSDDLCQTDMDDLCNIESVRNNVLSQSTDLNKSFNPMCDSTDNWS
CKFDESPKVMKSKSNLLSYQASQLQTGVPCDEETSLTFAGGSSVAEDDYKGTELKIKSFLDEKAQDLKRLQTPLLE
EFHNAMNPGIPQGALGDTNIYNLPNLPSISKTPKRLPSRRLSAISDAMPSPLKSSKRTLNTSRVMQSGTEPTQV
NESTKKGVNNSRCFSEIRRKWEEELYEELERHRENLRHAGAGGKTPLSGHKG

>AT1G07150AtMEKK13
MEKQSIRNTCSSSLMLSSPSSFWVRGACIGRGCFGAVSTAISKTNGEVFAVKSVDLATSLPTQSESLENEISVFRSL

KPHPYIVKFLGDGVSKEGTTTFRNLYLEYLPNGDVASHRAGGKIEDETLLQRYTACLVSALRHVHSQGFVHCDVK
ARNILVSQSSMVKLADFGSAFRIHTPRALITPRGSPLWMAPEVIRREYQGPESDVWSLGCTIIEMFTGKPAWED
HGIDSLSRISFSDELPVFPSKLSEIGRDFLEKCLKRDPNQRWSCDQLLQHPFLSQCHNSSPTESSPRCVLDWVNS
GFDLEEEEEEVGRSEFEDAAKAIICNLATTGGVIWESDGWVEVRCHASEEEGTTMEYSGSTRVESEYNTSSDPN
DDVAGDSAIIDVSMSQNLPPGNGGSAAALPYEFVVVLHLLMEIMVYTTCIFREIVLTMYLLYQYNQSNKLETLSF
NHSLKFCLFAHVIRIGQNYLLRGEMRSSVLITSHCLILITLVYSQIVVF

>AT2G30040AtMEKK14
MEKQNIISNTSSSSSSWIRGSCVGRGCFGTVSKALSKIDGGLFAVKSIDLATCLPSQAESLENEIVILRSMKSHPNI
VRFLGDDVSKEGTASFRNLHLEYSPEGDVANGGIVNETLLRRYVWCLVSALSHVHSNGIVHCDVKSKNVLVFNG
GSSVKLADFGSAVEFEKSTIHVSPRGSPLWMAPEVVRREYQGPESDVWSLGCTVIEMLTGKPAWEDHGFDSLS
RIGFSNDLPFIPVGLSELGRDFLEKCLKRDRSQRWSCDQLLQHPFLCQDHHDSFFTESSPRCVLDWVNSEFDEEE
ESDEWRPESMVSAMARISKLAITGGANWESNGWTEVRDTSEESEAKKEVLVSPRVELESYISLESSSDDSVRQP
RNEESATELASAVTCEAILLVMILVVENIQIYATFYTSSIIMHILYCCSCCCYYHYQNNNKKNNFSKSTSFILSLNFLFG
IACDSDRSIY

>AT5G55090AtMEKK15
MEEQNWIRGPIIGRGSTATVSLGITNSGDFFAVKSAEFSSSAFLQREQSILSKLSSPYIVKYIGSNVTKENDKLMYN
LLMEYVSGGSLHDLIKNSGGKLPEPLIRSYTRQILKGLMYLHDQGIVHCDVKSQNVMIGGEIAKIVDLGCAKTVE
ENENLEFSGTPAFMSPEVARGEEQSFPADVWALGCTVIEMATGSSPWPELNDVVAAIYKIGFTGESPVIPVWLS
EKGQDFLRKCLRKDPKQRWTVEELLQHPFLDEEDNDSDQTGNCLNSSSPSTVLDQRFWDLCETSRSRFIKEDHE
DPFANSTNFLWDDDSLPGDRIKKLAGDESSGEPDWETNGWIEVRGEIEKRNEEEDENCVEATSLEEDEEEVGG
FENWIWDQQDSLFLEYSPEDNIYYFYSYYNIFDEDIILYYDHLEDCFVLKFDDNNKKIFFFSHITNSCFEITNYN

>AT4G26890AtMEKK16
MEINWTRGPIIGRGSTATVSIAISSSGELFAVKSADLSSSSLLQKEQSILSTLSSPHMVKYIGTGLTRESNGLVYNIL
MEYVSGGNLHDLIKNSGGKLPEPEIRSYTRQILNGLVYLHERGIVHCDLKSHNVLVEENGVLKIADMGCAKSVD
KSEFSGTPAFMAPEVARGEEQRFPADVWALGCTMIEMMTGSSPWPELNDVVAAMYKIGFSGESPAIPAWISD
KAKDFLKNCLKEDQKQRWTVEELLKHPFLDDDEESQTSDCLKNKTSSPSTVLDQRFWDSCESSKSHLVSIDHED
PFAEYSESLDSPADRIEKLAGDEFSSLLDWDTEDDGGWIQVRGEKHKETEKRDGDEDVICVEATSSSQTIEEVED
WISNQDSLFSEYSSDDIINNFYSNVAIQGNLIAFHYCTDEDENVSIKNMFRTNNKHLFFKLQAHVKFTNQV

>AT2G32510AtMEKK17
MEWTRGRILGRGSTATVYAAAGHNSDEILAVKSSEVHRSEFLQREAKILSSLSSPYVIGYRGSETKRESNGVVMY
NLLMEYAPYGTLTDAAAKDGGRVDETRVVKYTRDILKGLEYIHSKGIVHCDVKGSNVVISEKGEAKIADFGCAKR
VDPVFESPVMGTPAFMAPEVARGEKQGKESDIWAVGCTMIEMVTGSPPWTKADSREDPVSVLYRVGYSSETP
ELPCLLAEEAKDFLEKCLKREANERWTATQLLNHPFLTTKPDIEPVLVPGLISNSPTSVTDQTFWRSVEEEEEETE
EIQKDSRDLDRLSLWGCYSERIGRLKCVGGLDGTRCDMEGGDWIMVRARCEGTMISGSQKELIISENVLVGEL

>AT1G05100AtMEKK18
MNWTRGKTLGRGSTATVSAATCHESGETLAVKSAEFHRSEFLQREAKILSSLNSPYVIGYRGCEITREPFHNNGE
ATTYSLLMEYAPYGTLTDVATKNGGFIDEARVVKYTRQILLGLEYIHNSKGIAHCDIKGSNVLVGENGEAKIADFG
CAKWVEPEITEPVRGTPAFMAPEAARGERQGKESDIWAVGCTVIEMVTGSQPWIGADFTDPVSVLYRVGYLG
ELPELPCSLTEQAKDFLGKCLKKEATERWTASQLLNHPFLVNKEPELVTGLVTNSPTSVTDQMFWRSVEEEVSED

RSSWWECHEDERIGVLSWIGHVVVESTWDLDGEDWITVRRN

>AT5G67080AtMEKK19
MEWIRGETIGYGTFSTVSLATRSNNDSGEFPPLMAVKSADSYGAASLANEKSVLDNLGDDCNEIVRCFGEDRTV
ENGEEMHNLFLEYASRGSLESYLKKLAGEGVPESTVRRHTGSVLRGLRHIHANGFAHCDLKLGNILLFGDGAVKI
ADFGLAKRIGDLTALNYGVQIRGTPLYMAPESVNDNEYGSEGDVWALGCVVVEMFSGKTAWSLKEGSNFMSL
LLRIGVGDEVPMIPEELSEQGRDFLSKCFVKDPKKRWTAEMLLNHPFVTVDVDHDVLVKEEDFVVNMKTEDVS
TSPRCPFEFPDWVSVSSGSQTIDSPDERVASLVTDMIPDWSVTNSWVTVR

>AT3G50310AtMEKK20
MEWVRGETIGFGTFSTVSTATKSRNSGDFPALIAVKSTDAYGAASLSNEKSVLDSLGDCPEIIRCYGEDSTVENGE
EMHNLLLEYASRGSLASYMKKLGGEGLPESTVRRHTGSVLRGLRHIHAKGFAHCDIKLANILLFNDGSVKIADFG
LAMRVDGDLTALRKSVEIRGTPLYMAPECVNDNEYGSAADVWALGCAVVEMFSGKTAWSVKEGSHFMSLLIRI
GVGDELPKIPEMLSEEGKDFLSKCFVKDPAKRWTAEMLLNHSFVTIDLEDDHRENFVVKVKDEDKVLMSPKCPF
EFDDWDSFTLDSNPSFDSPVERLGSLVSGSIPDWSVGGSWLTVR

>AT4G36950AtMEKK21
MEWIRRETIGHGSFSTVSLATTSGSSSKAFPSLMAVKSSGVVCSAALRNERDVLDDLGDCSEIVRCFGEGRTVEN
GEEIYNLFLEYASGGSLADRIKSSGEALPEFEVRRFTRSIVKGLCHIHGNGFTHCDIKLENVLVFGDGDVKISDFGL
AKRRSGEVCVEIRGTPLYMAPESVNHGEFESPADIWALGCSVVEMSSGKTAWCLEDGVMNNVMSLLVRIGSG
DEVPRIPVELSEEGKDFVSKCFVKNAAERWTAEMLLDHPFLAVDDESGEEDEACSVSPRNPFDFPGWNSVQSP
VNDSVMFGSLVGSPEERISGLVSEKVPDWSVSCDWVNVR

>AT3G51630AtZIK1
MYMEISSASDDSIAYVETDPSGRYGRFREVLGKGAMKTVYKAFDQVLGMEVAWNQVKLNEVFRSPEPLQRLYS
EVHLLKNLNHESIIRYCTSWIDVNRRTFNFITELFTSGTLREYRRKYQKVDIRAIKSWARQILNGLAYLHGHDPPVI
HRDLKCDNIFVNGHLGQVKIGDLGLAAILRGSQNAHSVIGTPEFMAPELYEEDYNELVDIYSFGMCVLEMLTGE
YPYSECTNPAQIYKKVTSGKLPDSFHLIQHTEAQRFVGKCLETVSRRLPAKELLADPFLAATDERDLAPLFRLPQQL
AIQNLAANGTVVEHLPSTTDPTRTTDMSITGKMNSEDHTIFLQVQILDGDGHMRNIQFPFNILSDTPLEVALE
MVKELEITDWDPLEIAAMIENEISLLVPNWRANDSSIRHESFGHEDDEDNGDTEGRTRLFSSASSSHDSPVAVR
ENNDDSSNDVIPDMDDGNRSSNRLLNSSTYHYSPAIDDDQNQQQRRRVRLQQKMRSLVDTRTQVLHRSLME
LINKRRGRGFDPNTNELQPQPSSTDFIRRC

>AT5G58350AtZIK2
MNMNQVAEYVETDPTGRYGRFAEILGRGAMKTVYKAIDEKLGIEVAWSQVKLKEVLRSSVDLQRLYSEVHLLST
LNHKSIIRFYTSWIDVHNHTLNFITELFTSGTLRQYKNKYLRIDIRAIKSWARQILEGLVYLHEHDPPVIHRDLKCD
NIFVNGHLGQVKIGDLGLARMLRDCHSAHSIIGTPEFMAPELYEENYNELIDVYSFGMCFLEMITSEFPYSECNH
PAQIYKKVVGGKLPGAFYRVGDIEAQRFIGKCLVSASKRVSAKELLQDPFLASDESWMVYTSGAGNPKPFLNEN
EMDTLKLEDDELRTEMSIAGKLGAEDNKIDLEVQIAYDNGLANNVFFPFDIMNDTSIDVAKEMVKELEIIDWEP
VEIAKMIDGAISSLVSDWKYEEDDETPHDHHRHRTDSFHSSSSHASSSQASLSNYMARGLQDWVQDDLHDET
YSQSSSHSGSYSNLNYIAVDEYSSQSPVMSRTHNMTRFCPEESSHLQSGQANAYAASSSTNRSLASDNRTLTRN
RSLVDVQRQLLHRSPGEEARKRRLFKTVGDVETVGFQSPYAVSRKPPSSRR

>AT3G22420AtZIK3

MNGEESFVEDCSVFVEIDPSGRYGRYDEILGKGASKTVYRAFDEYEGIEVAWNQVKLRNFTRNPEELEKFFREIH
LLKTLNHQNIMKFYTSWVDTNNLSINFVTELFTSGTLRQYRLRHRRVNIRAVKQWCKQILKGLLYLHSRSPPIIHR
DLKCDNIFINGNQGEVKIGDLGLAAILRKSHAVRCVGTSKPSHHWNFIALIMFFTTLDLPLLCLCVVKGTPEFMA
PEVYDEEYNELVDVYAFGMCVLEMVTFDYPYSECTHPAQIYKKVTSGKKPEAFYLVKDPEVREFVEKCLANVTCR
LTALELLQDPFLQDDNMDGFVMRPIDYYNGYDETGVFLRHPLIDDPLYHDQFESSQICEIDLFANDDEDHVDISI
KGKRNGDDGIFLRLRISDAEGIVSIFFDSFKIIGLKNVTRFRVLGNVGRIRNIYFPFETAIDTAWSVAVEMVSELDIT
NQDVAKIAEMIDAEIAALVPDWKNDTESSQNVNNNKNNNTAGFCGECASNGYIQETVSSGEKSHHNHHEFDS
SEDKSCSSVHGRFADMWGLRESYSDDGEKQSSRKVRSGRWSENEMRRELRWLKARHKIQLMKMRGQTICET
PIEISLTPGTSVSLPLLYRAISLPVDAVDM

>AT3G04910AtZIK4
MNNLSYLEPDYSEFVEVDPTGRYGRYNEVLGKGASKTVYRAFDEYEGIEVAWNQVKLYDFLQSPEDLERLYCEIH
LLKTLKHKNIMKFYTSWVDTANRNINFVTELFTSGTLRQYRLRHKRVNIRAMKHWCRQILRGLHYLHSHDPPVI
HRDLKCDNIFVNGNQGEVKIGDLGLAAILRKSHAAHCVGTPEFMAPEVYEEAYNELVDIYSFGMCILEMVTFDY
PYSECTHPAQIYKKVMSGKKPDALYKVKDPEVKCFIEKCLATVSLRVSARELLDDPFLRIDDGEFDLRSVDMEDSV
GPLYRQPHHLPDYYNYPSNSSSLNRQYSNGNYPSNSSSLNRQYSNGYNSHHEYQNGWAYNPAETEETHGIELF
ESRNNDDQEEEKKSGNVDITIKGKRRDDGGLFLRLRIADKEGRVRNIYFPFDIETDTALSVATEMVAELDMDDH
GVTKIANMIDGEISSLVPSWRPGPEFEECLAAAAAANAASICNNCVSNRTSMGSVMDFLRTNPGANVIQCCR
NGCGETHGRFEEITIRETEVRLRELWKLQQQQESRELSSIDSGHNHSEEEEEEEVLYEDPENMFSCEAGNEINHI
SGSGSFSFMPSKYCDEPSEKTENQVQQELRWLKAKCQIELRDIQDEQLKTRWPESGEEVEISPKDGFLGSVSGL
GREEDTVKEMFGERLVPKCLKRTTSLPVDAIDS

>AT3G18750AtZIK5
MEGTDDASALQEPPDPEVLEVDPTFRYIRYKEVIGKGAFKTVYKAFDEVDGIEVAWNQVRIDDVLQSPNCLERL
YSEVRLLKSLKHNNIIRFYNSWIDDKNKTVNIITELFTSGSLRHYRKKHRKVNMKAVKNWARQILMGLRYLHGQ
EPPIIHRDLKCDNIFINGNHGEVKIGDLGLATVMEQANAKSVIGTPEFMAPELYDENYNELADIYSFGMCMLEM
VTFDYPYCECKNSAQIYKKVSSGIKPASLSRVKDPEVKQFIEKCLLPASERLSAKELLLDPFLQLNGLTMNNPLPLP
DIVMPKEGAFGDRCLMSEGPPTTRPSKTLSIDLDEDSNLPIVTFSDNSGSRCIEVRRAKRGNFFVLKGEENDEQS
VSLILRIVDENGRVRNIHFLFYQEGDTASKVSSEMVEQLELTDQNVTFIAELIDILLVNMIPTWKTDVTVDHLIHS
QLNQNSRSHHNEAKPQKQEETVFHDTCELVSHSCNSDCPRSDEEDKQCVDATKGEDKSSIQEVEEATEPVSLEE
EERLRQELEEIEAKYQEDMKEIATKREEAIMETKKKLSLMKLK

>AT5G41990AtZIK6
MASGSGFLGQISSMEEADFAEKDPSGRYIRYDDVLGRGAFKTVYKAFDEVDGIEVAWNLVSIEDVMQMPGQL
ERLYSEVHLLKALKHENIIKLFYSWVDEKNKTINMITELFTSGSLRVYRKKHRKVDPKAIKNWARQILKGLNYLHS
QNPPVIHRDLKCDNIFVNGNTGEVKIGDLGLATVLQQPTARSVIGTPEFMAPELYEEEYNELVDIYSFGMCMLE
MVTCEYPYNECRNQAQIYKKVTSNIKPQSLGKVDDPQVRQFIEKCLLPASSRPTALELSKDPFLARDGGKDSALL
ASSSTSSKYVRPPQLEHLPMDVDHNENKSVSSNEDYPWSQTIELQRIAENKEFRLRGERSDDVTASMVLRIADP
SGKCRIVHFAFYLESDTATAIAEEMVEELHLTSQEVVVIADMIDDFIMQLLSDRTSSHHNQNSPRLTHEDHEAAN
QQTVNSKDEEAAGQSMKSDISADYYFPYSANDGNAAMEAGRDAESMSSYLDSCSMMSTIYNLSISDNDYPED
LKTELNLIESQFNQSFQDLLKLKEDAIENAKRKWITKKQKAVNIS

>AT1G49160AtZIK7
MEGSEDASAIVEPPDPEVLEIDPTCRYIRYKEVIGKGASKTVFKGFDEVDGIEVAWNQVRIDDLLQSPDCLERLYS

EVRLLKSLKHKNIIRFYNSWIDDKNKTVNIITELFTSGSLRQYRKKHRKVNMKAVKCWARQILTGLKYLHSQDPPII
HRDIKCDNIFINGNHGEVKIGDLGLATVMEQANAKSVIGTPEFMAPELYDENYNELADIYSFGMCMLEMVTFE
YPYCECRNSAQIYKKVSSGIKPASLSKVKDPEVMKFIEKCLLPASERLSAEELLLDSFLNVNGLVMNNPLPLPDIVM
PKEGSFGERCLMSEGPPNARNRTMSMNLDEDNNLPIVISSNNSGTNCIEVRRAKRGNFFVLKGEENDENSVSL
ILRIVDENGRVRNIHFLFFQEGDTASNVSSEMVEQLELTDKNVKFIAELIDVLLVNLIPNWKTDVAVDHLIHPQQ
NQSSKDNHQNGASSQAGESISHSLSSDYCPRSDDEANPTVAATTEDQEAEKPGSLEEEEEDERLKEELEKIEERF
REEMKEITRKREEATMETKNRFFEKKMQQVE

>AT5G55560AtZIK8
MMTCASSDDNESEKDKDSESFVEVDPTGRYGRYGELLGSGAVKKVYRAFDQEEGIEVAWNQVKLRCFSDDPA
MTERLYSEVRLLKNLKNSNIITLYKVWRDERNNTLNFITEICTSGNLREYRKKHRHVSMRALKKWSKQILKGLDYL
HTHDPCIIHRDLNCSNIFVNGNIGQVKIGDLGLAAIVGKNHLAHSILGTPEFMAPELYEENYTEMVDIYSYGMCV
LELVSLEIPYSECDSVAKIYKRVSKGLKPEALNKVNDPEAKAFIEKCIAQPRARPSAAELLCDPFFDGILDDDDEDG
ENNDNNGAGRIVVS

>AT5G28080AtZIK9
MMNNLSHLESDYSEYVEVDPTGRYGRYNEVLGKGSSKTVYRGFDEYQGIEVAWNQVKLYDFLQSPQELERLYC
EIHLLKTLKHKSIMKFYASWVDTDNRNINFVTEMFTSGTLRQYRLKHKRVNIRAVKNWCRQILRGLNYLHTHDP
PVIHRDLKCDNIFINGNQGEVKIGDLGLAACLQHSHAAHCVGTPEFMAPEVYKEEYNQLVDIYSFGMCVLEMV
TFDYPYSECSHPAQIYKRVISGKKPDGLDKVKDPEVRGFIEKCLATVSLRLSACELLDDHFLCIDESDMRRVESEKG
LIDEAGTPLRHSYHIPHYSNGYYSLYNQNQWDYNGDETVESHEIDLLEFQNDDDEEEEDKRFGSVDISIKGKRRD
NGDGLFLRLKTVNKEGCVRNIYFPFDIETDTAISVAREMVEELEMDDRDVTKIANMIDGEIASLVPNWSIFCSSE
SNRSSVGSVMDFNEMQCGRDGCEEKHGRFEEITFEITVNDSDEED

>AT1G64630AtZIK10
MEEADFVQKDPTGRYIRYNDVLGRGAFKTVYKAFDEVEGIEVAWNLMSIEDVLQMPGQLDRLYSEVHLLNSLK
HDNIIKLFYSWVDDHNKSINMITELFTSGSLTLYRKKHRKVDPKAIMNWARQILKGLHYLHSQTPPVIHRDLKCD
NIFVNGNTGKVKIGDLGLAAVMQQPTARSVIGTPEFMAPELYEEEYNELVDIYSFGMCMLEMVTCEYPYRECR
NQAQIYKKVTSGIKPQSLSKVDDPQVKQFIEKCLLPAPSRPTALELLKDQLLAVDGAKDSTLTASSNTTFKPAMPP
QCEYRPMDVEYKKNTSVSICSSAKSSQECALLQTMEVQRVAESTEFKLSGERRDDVAASMALRIAGSSGQARK
VDFDFNLKTDTARAVTGEMVEELDLSSHEVTVIAEMIDELIMKLKANRSLPNANSVYQSKDEEAGESMKSEISA
DYYHRVSSNEGSRLGCCCEAVESLLSSFLDSCSMVSNKQSEDLKTELNVIESQYNQSCQRLLRMKEEAIEKAKRK
WMKLS

>AT3G48260AtZIK11
MRQDENNSEEEFVEIDPTGRYGRYKEVLGKGAFKEVYRAFDQLEGIEVAWNQVKLDDKFCSSEDLDRLYSEVHL
LKTLKHKSIIKFYTSWIDHQHMTINLITEVFTSGNLRQYRKKHKCVDLRALKKWSRQILEGLVYLHSHDPPVIHRD
LKCDNIFINGNQGEVKIGDLGLAAILHRARSAHSVIGTPEFMAPELYEEDYNVLVDIYAFGMCLLELVTFEYPYSEC
TNAAQIYRKVTSGIKPAALLNVTDPQVRAFIEKCIAKVSQRLSAKELLDDPFLKCYKENTENVSSHKENGYNGNGI
VDKLSDSEVGLLTVEGQRKDLNTIFLKLRITDSKGQIRNIHFPFNIETDTSFSVAIEMVEELDLTDDQDISTIAKMID
TEIHSHIPDWTPSRLIGDDSAVQKCLSSPETLHLDRFPSGRKFWSSPKAGAGDSRSPFAPRSNSKLSSAQGPINQ
EVGVIVEKLESLLRKQREEIEEMQRDQERIVTEFLKEFPPEICEEALVRLQVKDSDNLLC

>AT5G03730AtRAF1

MEMPGRRSNYTLLSQFSDDQVSVSVTGAPPPHYDSLSSENRSNHNSGNTGKAKAERGGFDWDPSGGGGGD
HRLNNQPNRVGNNMYASSLGLQRQSSGSSFGESSLSGDYYMPTLSAAANEIESVGFPQDDGFRLGFGGGGGD
LRIQMAADSAGGSSSGKSWAQQTEESYQLQLALALRLSSEATCADDPNFLDPVPDESALRTSPSSAETVSHRFW
VNGCLSYYDKVPDGFYMMNGLDPYIWTLCIDLHESGRIPSIESLRAVDSGVDSSLEAIIVDRRSDPAFKELHNRV
HDISCSCITTKEVVDQLAKLICNRMGGPVIMGEDELVPMWKECIDGLKEIFKVVVPIGSLSVGLCRHRALLFKVL
ADIIDLPCRIAKGCKYCNRDDAASCLVRFGLDREYLVDLVGKPGHLWEPDSLLNGPSSISISSPLRFPRPKPVEPAV
DFRLLAKQYFSDSQSLNLVFDPASDDMGFSMFHRQYDNPGGENDALAENGGGSLPPSANMPPQNMMRAS
NQIEAAPMNAPPISQPVPNRANRELGLDGDDMDIPWCDLNIKEKIGAGSFGTVHRAEWHGSDVAVKILMEQ
DFHAERVNEFLREVAIMKRLRHPNIVLFMGAVTQPPNLSIVTEYLSRGSLYRLLHKSGAREQLDERRRLSMAYDV
AKGMNYLHNRNPPIVHRDLKSPNLLVDKKYTVKVCDFGLSRLKASTFLSSKSAAGTPEWMAPEVLRDEPSNEKS
DVYSFGVILWELATLQQPWGNLNPAQVVAAVGFKCKRLEIPRNLNPQVAAIIEGCWTNEPWKRPSFATIMDLL
RPLIKSAVPPPNRSDL

>AT1G08720AtRAF2
MKHIFKKLHRGGNQEQQNRTNDAAPPSDQNRIHVSANPPQATPSSVTETLPVAGATSSMASPAPTAASNRAD
YMSSEEEYQVQLALAISASNSQSSEDPEKHQIRAATLLSLGSHQRMDSRRDSSEVVAQRLSRQYWEYGVLDYEE
KVVDSFYDVYSLSTDSAKQGEMPSLEDLESNHGTPGFEAVVVNRPIDSSLHELLEIAECIALGCSTTSVSVLVQRL
AELVTEHMGGSAEDSSIVLARWTEKSSEFKAALNTCVFPIGFVKIGISRHRALLFKVLADSVRLPCRLVKGSHYTG
NEDDAVNTIRLEDEREYLVDLMTDPGTLIPADFASASNNTVEPCNSNGNKFPTAQFSNDVPKLSEGEGSSHSSM
ANYSSSLDRRTEAERTDSSYPKVGPLRNIDYSSPSSVTSSTQLENNSSTAIGKGSRGAIIECSRTNMNIVPYNQNSE
EDPKNLFADLNPFQNKGADKLYMPTKSGLNNVDDFHQQKNNPLVGRSPAPMMWKNYSCNEAPKRKENSYIE
NLLPKLHRDPRYGNTQSSYATSSSNGAISSNVHGRDNVTFVSPVAVPSSFTSTENQFRPSIVEDMNRNTNNELD
LQPHTAAVVHGQQNDESHIHDHRKYTSDDISTGCDPRLKDHESTSSSLDSTSYRNDPQVLDDADVGECEIPWN
DLVIAERIGLGSYGEVYHADWHGTEVAVKKFLDQDFSGAALAEFRSEVRIMRRLRHPNVVFFLGAVTRPPNLSIV
TEFLPRGSLYRILHRPKSHIDERRRIKMALDVAMGMNCLHTSTPTIVHRDLKTPNLLVDNNWNVKVGDFGLSRL
KHNTFLSSKSTAGTPEWMAPEVLRNEPSNEKCDVYSFGVILWELATLRLPWRGMNPMQVVGAVGFQNRRLEI
PKELDPVVGRIILECWQTDPNLRPSFAQLTEVLKPLNRLVLPTPQ

>AT5G11850AtRAF3
MSKMKHLLRKLHIGGSSGVGGGFADHHRLDDSTRPMIDPSPILSTSPSPASTSSVSSSGFGNASTTMPRLDTFE
PVGRDLTAVDGVDFNLMEEEYQVQLAMAISVSDPDPRENADTAQLDAAKRISLGVSAPVTDADSAVDFLSLRY
WGHKVINYDQKVRDGFYDVYGITSNSLSQGKMPLLVDLQAISISDNVDYEVILVNRLIDPELQELERRVFALASEC
PDFAPGQVSSDLTQKIANIVVEQMGGPVENADEALRRWMLRSYELRNSLNTTILPLGRVNVGLARHRALLFKV
LADRINLPCMLVKGSYYTGTDDGAVNLIKLDDKSEYIIDLMGAPGALIPSEVPSSFLPVSCTDTRVFPENLDSLQH
SSPVLEKEIETPAFSVSKEADSRSGMVANFFTGNQEENSDRCAVEKHQTERFEHDFGKLMHSQQISGENMPPF
SGKPTCAQKVKVKNVSKYVISAAKNPEFAQKLHAVLLESGASPPPDLFMDINPHNLRGKNLLQELRQESSNSM
VSGIPCYPEKVAEQLRESERNPTAESYQQSVEVDLSMKRNFDLDNTGKASSSENMEVGTADGESAVCDSHDQG
INPLLGEAAKWEIMWEDLQIGERIGIGSYGEVYRAEWNGTEVAVKKFLDQDFSGDALTQFKSEIEIMLRLRHPN
VVLFMGAVTRPPNFSILTEFLPRGSLYRLLHRPNHQLDEKRRMRMALDVAKGMNYLHTSHPTVVHRDLKSPNL
LVDKNWVVKVCDFGLSRMKHHTYLSSKSTAGTPEWMAPEVLRNEPANEKCDVYSFGVILWELATSRVPWKGL
NPMQVVGAVGFQNRRLEIPDDIDLTVAQIIRECWQTEPHLRPSFTQLMQSLKRLQGLNISNRANTSESLM

>AT1G18160AtRAF4
MKMNMKKFLKKLRITPNQRDDGEGSVSNRSNKSSDAEPSPSDSLRSQDNSEFKPFLGLSNWLSSVTHRKSPSS

SNATNSKEDDTTMEHGGPVGSESGMQGLGSSSNSKDPEVEEEYQIQLALELSAREDPEAAQIEAMKQFSLGSR
PSAPENTPAELMAYRYWNYNCLGYDDKIVDGFYDLCGVMNESSLKRIPPLVDLQGTLVSDGVTWDAVLVNSSK
DSNLLRLEQMALDIAAKSKSASSSGFVNSELVRQLAVLVADYMGGPVLDPDSTLRAWWSLSYSLKATLRSMVLP
LGSLTIGLARHRALLFKVLCDSVGVPCRIVKGQQYTGSDDVAMNSIKTDDGREYIVDLMGDPGTLIPADAAGLQ
MDFDDSVYSASPRDVDSSHVASSSSGVESSIEEHTESWSAEHRSRTKGSREENQSAGGGDLMIPNIREAVGSQ
KAPVQHLSSKPTHSFTHARSPSWTEGVSSPAGRRMKVKDVSQYMIDAAKENPQLAQKLHDVLLESGVVAPRN
LFSEVYSESMEATGEIKSVAESNDEKGKDFGTIQQGRNQSNLGPVRFLPPLPRPQSKAITHDLREHSGSGLGHLS
EHCNIDGHSDSSHSETSTDYPRNVPVAVAAAAVVASSMVVAAAKSANSDSSTLELSAAAAAAVMATAAAVSRQ
FELDSLSNGDAGSGGLHGVDSGGERISDRSIGNESSKSDAAIDDVAECEILWEEITVAERIGLGSYGEVYRGDWH
GTAVAVKKFIDQDITGEALEEFRSEVRMMRRLRHPNIVLFMGAVTRPPNLSIVTEFLPRGSLYRLIHRPNNQLDE
RKRLRMALDAARGMNYLHSCNPVIVHRDLKSPNLLVDKNWVVKVCDFGLSRMKVSTYLSSKSTAGTAEWMA
PEVLRNEPADEKCDVYSYGVILWELFTLQQPWGKMNPMQVVGAVGFQHRRLDIPEFVDPGIADIIRKCWQTD
PRLRPSFGEIMDSLKQLQKPIQRAAVPSSSALTTDEQEQ

>AT1G73660AtRAF5
MKVKEETLKNLGDGVVLRPVDHCSSIWSMKMNMKNFLKKLHISPNQSDEAEGSISTTKSNHHKSIDVSSSSSP
RSHHSNSPEIKPFSGLSNWLSSVGHRKIPSPPNSFNAKNRAATVDDTVVVNGSEHVDLGSKDPAVEEENQIQLA
LELSAREDPEATQIEAIKQFSLGSCAPENSPAELIAYRYWNYNCLGYDDKILDGFYDLYGVLNASSAERIPPLLDLQ
GTPVSDGVTWEAVLVNRSGDSNLLRLEQMALDIAAKSRSVSSSGFVNSELVRKLAILVGDYMGGPVVHPESML
RAWRSLSYSLKATLGSMVLPLGSLTIGLARHRALLFKVLCDSVGVPCRIVKGQQYTGSEDVAMNFIKADDGREYI
VDLMGDPGTLIPADAAGLQIDYDESAYSASPGDNDSIHVASSSNGIESSYEENTEFRTGEHRSSTKSSGERNQSG
GGGDLIVHPNISREDVKNQKKVEKAPFQNLSSRPIHSFTHMRSPSWTEGVSSPAAQRMKVKDVSQYMIDAAK
ENPRLAQKLHDVLLESGVVAPPNLFSEVYPQQLEATVESKNSTEAKKERGKDLETTQEGRHQNGFGPVRFLPPL
PRVQSKTNAHDQRDNGKVVSQSDSSHSEASSTEYARTVPAAVAAAAVVASSMVAAAAAKSANSDSSPIELPAA
AAATATAAAVVATAAAVSRQLELGSNSDGDDGSGGHEPQGSGDSNHGPNSGGERISDKSIGNESSKSDCDDVS
DCEILWEEITVGERIGLGSYGEVYRGDWHGTEVAVKKFLDQDLTGEALEEFRSEVRIMKKLRHPNIVLFMGAVTR
PPNLSIVTEFLPRGSLYRLIHRPNNQLDERRRLRMALDAARGMNYLHSCNPMIVHRDLKSPNLLVDKNWVVKV
CDFGLSRMKHSTYLSSKSTAGTAEWMAPEVLRNEPADEKCDVYSYGVILWELFTLQQPWGKMNPMQVVGAV
GFQHRRLDIPDFVDPAIADLISKCWQTDSKLRPSFAEIMASLKRLQKPVTGSNIPRPVPSSSSLPTEHEQKD

>AT4G24480AtRAF6
MPHRTTYFFPRQFPDRGFDSFSLKNDHDKKKSSSNVGESFGFQRDNKSNGVGEDSNKEKESTVFSSNPLLSKSS
AVSDLFSDDRKSEKKHQQQLAAFYEWLAEKKANLSRSSSTTTTHGRGVKPTRFSMSSDADEERELLLSSPADPA
PLPATSSPDSIIDSARTVNIHERNIDRSFDREVSLPRMSSESSFAGSFFSGTTVDGNFSNFSSHTDARETSTTTLVSV
NKEEEEVEVREQGKEQSLAQKSREGYYLQVTLAKWLSSQANLACESVHIQSTESISYRFWVSGCLSYSDKISDGF
YSILGMDPYLWLMCNNSEDGKRIPSLLLLKETEPNDTSMEVVLIDRREDSRLKELEDKAHELYCSSDNMLVLVEK
LGRLVAVYMGGNFQVEQGDLQKRWKLVSNRLKEFRKCIILPIGSLTMGLCRHRAILFKKLADYIGLPCRIARGCRY
CKESHQSSCLVKIDDDRKLSREYVVDLIGEPGNVHDPDSSINGETQCQIPSPLQMSHLTDFSRPCVHSTSPCQTV
ESKTSRTLSENIQRSGSQGQVHKEFELPDNAGTVCCAHIDQTCCAKVSSMVLTESVLRALPLDIPNLSEEKIAPQE
TCKEETVLLEDPTAMKQPNLSVEPEIVEADTRKDKKGRLPVDAISPYLTIEPSLASDWLEVSWNELHIKERVGAGS
FGTVHRAEWHGSDVAVKILSIQDFHDDQFREFLREVCKQAVAIMKRVRHPNVVLFMGAVTERPRLSIITEYLPR
GSLFRLIHRPASGELLDQRRRLRMALDVAKGLNYLHCLNPPVVHWDLKSPNLLVDKNWTVKVCDFGLSRFKAN
TFIPSKSVAGTPEWMAPEFLRGEPTNEKSDVYSFGVVLWELITLQQPWNGLSPAQVVGAVAFQNRRLIIPPNTS
PVLVSLMEACWADEPSQRPAFGSIVDTLKKLLKSPVQLIQMGGDKGVIPTKSAPIL

>AT3G06620AtRAF7

MENPPAEELLKKILELEESQEHLKQEMSRLKVSTELRQRSHSVSPHRPARRNIGEGAPSWRKSGAASFRNASPLR
KESRIQNSMRLRSEVGGGGPSAGKFTDKQYLNILQSMAQAVHAFDLNMRIIFWNAMAEKVYGYSAAEALGEN
PINVIADDRDAAFAMNIARRCVRGESWTGEFPVKSKSGDRFSAVTTCSPFYDDDGALMGIICITSNTAPYLNPRI
SLAKLKAQEEGETSSIPARNSFASKLGLDSRGAVISKLGLDSDQPIQVAIASKISDLASKVSNKVRSKMRAGDNSA
TLSEGGSGDSHQKDHNVFGATLVDHRDDAASSGASTPRGDFIQSPFGVFTCNDEKFVSKPFKDSSDESDGKPAI
HKVLTSKAEEWMVKKGLSWPWKGNEQEGSKGRPTNSVWPWVQNEQKKERCHQINPSAGVQYESHAFESN
KPINNEASSLWSSPINANSTSSASSCGSTSSSVMNKVDTDSEGLEYEILWDDLTIGEQVGQGSCGTVYHGLWFG
SDVAVKVFSKQEYSAEVIESFKQEVLLMKRLRHPNVLLFMGAVTSPQRLCIVSEFLPRGSLFRLLQKSTSKLDWRR
RIHMALDIARGMNYLHHCSPPIIHRDLKSSNLLVDKNWTVKVADFGLSRIKHETYLTSKSGKGTPQWMAPEVLR
NESADEKSDIYSFGVVLWELATEKIPWETLNSMQVIGAVGFMDQRLEIPKDIDPRWISLMESCWHSDTKLRPTF
QELMDKLRDLQRKYMIQFQATRAALSDNSLLKDN

>AT3G06630AtRAF8

MEEELLKKMLELEQSQELLKQEMSRLKLSTELRQPSHPVLPRRPLRRIQGSMNSNPSPGKFTDKQYLNILQSLA
QSVHVLDLNTRIIFWNAMSEKLYGYSAAEVVGRNPVHVIVDDQNAAFALNVARRCANGESWTGEFPVKTKSG
KIFSAVTTCSPFYDDNGTVVGIISITSDIAPYLNPRLSLPRLKPQEPERKLGLDSKGAVISKPGLDSDQPIQVSIASKI
SSLASKLSNKVRSKMRAGDNSACGDSHHSDHDVFGDTLSDHRDDAASSGASTPRGDFIQSPFGVFTCYDDKFP
SKPSKDSSDRKPAIHKVPTSKAEEWMVKKGLSRPWKGNEQEGSRVRPTHSVWSWVENEQEKDKYHQIYPSA
GVKSESHGSESNKPTDDEASNMWSSSINANSTNSASSCGSTSRSVMDKVDIDSDPLEHEILWDDLTIGEQIGRG
SCGTVYHGIWFGSDVAVKVFSKQEYSESVIKSFEKEVSLMKRLRHPNVLLFMGAVTSPQRLCIVSEFVPRGSLFRL
LQRSMSKLDWRRRINMALDIARGMNYLHCCSPPIIHRDLKSSNLLVDRNWTVKVADFGLSRIKHQTYLTSKSGK
GTPQWMAPEVLRNESADEKSDIYSFGVVLWELATEKIPWENLNSMQVIGAVGFMNQRLEIPKDTDPDWISLIE
SCWHR

>AT3G06640AtRAF9

MVKLLQDPITPNKELLKKMIELEKSQEHLMQEMSRLKVSTELRKESRIQCSMNLRPSPWKFTHKQYLNILQSMA
QSVHAFDLNMRIIFWNAMAEKVYGYSAAEAVGQNPIDVMVDDRDAPFAMTIAQLCSNGESWTGKFPVKRRT
GEKFSAVTTCSPFYADDGSLIGIVSITSDVAPYLNPTISLAKLKASEVETSSTPARNSFAFKLGLDTKGAVVSKLGLDS
DQPIQVAIASKISDLASKVRNKVRSKMPAGDSSVTVFEGETGDSHHSDHGVFGATLSDHMDDAASSGASTPRG
DFIQSPFGVFTCNDDKFSSEPFIDSSDGYPITLFTSKAEEWMVKKGLSWPWKGNEQEGSRVKPTYSVWPCVQN
EQKKDKSHQINRYSGVKSKSHASESNKPTNNKASGLRSSCINANSAISRGIISHSTMNKVDTNSNCLEYEILWDD
LTIGEQIGQGSCGTVYHGLWFGSDVAVKLISKQEYSEEVIQSFRQEVSLMQRLRHPNVLLFMGAVTLPQGLCIVS
EFLPRGSLFRLLQRNMSKLDWRRRINMALDIARGMNYLHRCSPPIIHRDLKSSNLLVDKNLTVKVADFGLSRIKH
HTYLTSKSGKGMPQWMAPEVLRNESADEKSDIYSFGVVLWELATEKIPWENLNSMQVIGAVGFMNQRLEIPK
DIDPDWISLIESCWHRDAKLRPTFQELMERLRDLQRKYTIQFQATRWLTMVRTESSQVLKQNH

>AT5G49470AtRAF10

MEKTTPPAEELLKKIRELEESQEHLKREMSRLKVSAEMKQRSHSASPQRPVRRNSNDGTPMWRKTGAASFRH
ASPLRKESHAKVAGGGGEGQSAGKFTDKQYLNILQSMAQAVHVFDLNGQIIFWNSMAEKLYGFSASEALGKD
PIDILVDVQDASVAQNITRRCSSGESWTGEFPVKNKAGERFSVVTTMSPSYDDDGCLIGIICITNDSALFQDPRG
SPAKTRRGQEGETSFSRVTSSVASKLGLDSKEAVVSKLGLDSQQPIQVAIASKISDLVGNKVKSKMRAGDNNAA
NLEGGSGDSHQSDQGFFDAAFADRREDAATSGADTPRGDFIQSPFGVFLRSDEKASTKPFRDSSDESDGNSVV

PKTLTSKAEEWMVKKGLSWPWKGNEREGLEGRRSHSVWPWVRNEQQKQQAYQSNSNHSVKSESQACESIK
ASSNEPMGYWSSSVNVNSTSSSSSCGSTSSSVMNKVDMDSDCLDYEILWEDLTIGEQIGQGSCGTVYHGLWF
GSDVAVKVFSKQEYSEEIITSFRQEVSLMKRLRHPNVLLFMGAVTSPQRLCIVTEFLPRFGLITLANITLPFVLFELR
GQNSDLPYQITFAVEVSSVCCRGTRQNWIGGDVSIWPRIFYLAYYSLDSLIQLLYLVYNMLHIFLTYFFAQARGM
NYLHHCTPPIIHRDLKSSNLLVDKNWTVKVADFGLSRIKHETYLTTKTGRGTPQWMAPEVLRNEAADEKSDVYS
FGVILWELVTEKIPWESLNAMQVIGAVGFMNQRLEVPKNVDPQWISLMESCWHSEPQDRPSFQEIMEKLREL
QRKYTIQFQAARAASIENSALKEK

>AT1G67890AtRAF11
MENPNPPAEKLLKKIRELEESQEDLKREMSKLKVSAEIKRRSHSSSPKRPSRRNSGEGTPLWRKTGAASFRHASP
LRKESHSKDGVAGGGDGPSAGKFTDKQYLNILQSMAQAVHVFDLNGQIIFWNSMAEKLYGFSAAEALGKDSIN
ILVDGQDAAVAKNIFQRCSSGESWTGEFPVKNKMGERFSVVTTISPFYDDDGLLIGIICITNDSALFQRPRVPPAK
NRWQEGDSSFCRGTNGVASRLGFDSKEAVVSKLGLDSQQPIQAAIASKISDLASKVGNKVRSKMRAGDNNAS
HPEGGNGGSHQSDQGFFDAAFSDQREDAETNDASTPRGNLIQSPFGVFLCNDDKSSSKASGESNDENDRNSV
VPKKLTSKTEEWMVKKGLSWPWKGNEREGLERRNAHSVWPWVHNEQQKEEAHHSNSYNSVKSESLASESN
KPANNENMGSVNVNSASSASSCGSTSSSVMNKVDMDSDCLDYEILWEDLTIGEQIGQGSCGTVYHGLWFGSD
VAVKVFSKQEYSEEIITSFKQEVSLMKRLRHPNVLLFMGAVASPQRLCIVTEFLPRGSLFRLLQRNKSKLDLRRRIH
MASDIARGMNYLHHCSPPIIHRDLKSSNLLVDRNWTVKVADFGLSRIKHETYLTTNGRGTPQWMAPEVLRNE
AADEKSDVYSFGVVLWELVTEKIPWENLNAMQVIGAVGFMNQRLEVPKDVDPQWIALMESCWHSEPQCRP
SFQELMDKLRELQRKYTIQFQAARAASIDNSSLKEK

>AT4G23050AtRAF12
MAGNNSESSLYQVLVEWCQRMETSQARLREDVDDLLLQEESRTGKESATGLETDTEKEAEVEVEAEAADSWD
NPTATWERAVSGFYFADSAYRTLMDSMGHAIHVTSAASGEITFWSRSAENLYHWYAEEVVGYRTIDVLVTEEYR
NSLTGIRNRVCRGETWTGQFPFQKKTGELFMALVTKSPVYENGELVGVVTVSSDATLFNRMHPLSNEHQQQA
RSNNRHESNLRKHQWHLPRPQIAAASQVPVVPQYSSAVASNLKASKLLPQRNGDDSFNGNHNSRSRDENVP
VVASTTFEKYGSLADKFLGKLQRKITGSQGTEDNEPILRNGINKSACGSGGSSKASNAVTCTAFRDNGNGKPKR
AEVRISDVYGNGAEGLIHNGDRFQYIGNLGQSKPPRGLESGLVSGMRGTKMSDLNGEIEDAWNTRLSVDPLPI
LGVNSGRQQSPVNQRNNRLVTDSSCEIRWEDLQLGEEVGRGSFAAVHRGVWNGSDVAIKVYFDGDYNAMTL
TECKKEINIMKKLRHPNVLLFMGAVCTEEKSAIIMEYMPRGSLFKILHNTNQPLDKKRRLRMALDVARGMNYL
HRRNPPIVHRDLKSSNLLVDKNWNVKVGDFGLSKWKNATFLSTKSGKGTPQWMAPEVLRSEPSNEKCDVFSF
GVILWELMTTLVPWDRLNSIQVVGVVGFMDRRLDLPEGLNPRIASIIQDCWQTDPAKRPSFEELISQMMSLFR
KPGSGAQEEDD

>AT2G31010AtRAF13
MEERRDDESSPTHQGSELAERVKLLSFESQGEALSKDSPRSVEQDCSPGQRASQHLWDTGILSEPIPNGFYSVV
PDKRVKELYNRLPTPSELHALGEEGVRIEVILVDFQKDKKLAMLKQLITTLVSGSGTNPALVIKKIAGTVSDFYKRPT
LESPSKLALEENAFLFENHGAQLLGQIKRGCCRARAILFKVLADTVGLESRLVVGLPSDGTVNCMDSNKHMSVI
VVLNSVELLVDLIRFPGQLVPRSAKAIFMSHISPAGESDSAENDSCDSPLEPNSPLYERRDPESTEKDENLQFYRKL
EGYPNASGSSLRSLMLRPSTAIERKLSNTSHSEPNVATVFWRRSRRKVIAEQRTASSSPEHPSMRRGRSMLSTGR
NSFRDYTGEASSPSSSSTSEIRKTRRRSFRITPEIGDDIASAVREMYEKSKQNRLLQGREDENSSVIDNNVSGLHL
DDELNSKKTMSLPSSPHAYRCQTFGRRGPSEFAVKDTWNKVVESSTLQNQPLLPYQEWDIDFSELTVGTRVGIG
FFGEVFRGVWNGTDVAIKLFLEQDLTAENMEDFCNEISILSRVRHPNVVLFLGACTKPPRLSMITEYMELGSLYYL
IHMSGQKKKLSWHRRLRMLRDICRGLMCIHRMKIVHRDLKSANCLVDKHWTVKICDFGLSRIMTDENMKDTS

SAGTPEWMAPELIRNRPFTEKCDIFSLGVIMWELSTLRKPWEGVPPEKVVFAVAHEGSRLEIPDGPLSKLIADC
WAEPEERPNCEEILRGLLDCEYTLC

>AT2G42630AtRAF14
MMQSDLLKERGVDDSSPYSPDEKNVSGFQLDSHDLVSGECSTVYPRKSISLPSSPRSYQIQLSERSEHSPQEISHI
WNEVLESPMFQNKPLLPFEEWNIDFSKLKVGASVGSGTSGVVCRGVWNKTEVAIKIFLGQQLTAENMKVFCN
EISILSRLQHPNVILLLGACTKPPQLSLVTEYMSTGSLYDVIRTRKKELSWQRKLKILAEICRGLMYIHKMGIVHRDL
TSANCLLNKSIVKICDFGLSRRMTGTAVKDTEAAGTPEWMAPELIRNEPVTEKSDIFSFGVIMWELSTLSKPWK
GVPKEKVIHIVANEGARLKIPEGPLQKLIADCWSEPEQRPSCKEILHRLKTCEIPIC

>AT3G58640AtRAF15
MGETGDDAGPSEQGPSNQTWWPSEFVEKFGSVYLGSQEETSSTKDSPRNLGQDGLPSSTASNILWSTGSLSEP
IPNGFYSVIPDNRLKQLFNNIPTLEDLHALGDEGLKADVILVDFQKDKKLFRQKQLITKLVSGLNSKPATIIKKIAGLV
ADVYKQSTLQSPAKSTQSFENCGIQLLGQIKHGSCRPRAILFKVLADTVGLQSRLVVGLPSDGAAESVDSYSHISV
TVLLNSVEMLVDLMRFPGQLIPLSTKAIFMSHISAAGESDSAENDSCDSPLEPNSPMFGYPEKFDHENAEKDEN
LSLHRKLDGSPNTSGPPSRNMLLRSASALERKLSFSQSESNMANEFWRQSRRKVIADQRTASSSPEHLSFRART
KSMLSGDKNLARDFTGDVATSSCKSVGGAKMETKRIRRRSISITPEIGDDIVRAVRAMNEALKQNRLSKEQGDD
DSSPNSPNDRTESSHLQKNVSGFHLDAHDQVSGGRSTLSREPLDPQKAISLPSSPQNYRSQYEQSGSSHRNISHI
WDKVLGSPMFQNKPLLPYEEWNIDFSELTVGTRVGIGFFGEVFRGIWNGTDVAIKVFLEQDLTAENMEDFCNE
ISILSRLRHPNVILFLGACTKPPRLSLITEYMEMGSLYYLLHLSGQKKRLSWRRKLKMLRDICRGLMCIHRMGIVH
RDIKSANCLLSNKWTVKICDFGLSRIMTGTTMRDTVSAGTPEWMAPELIRNEPFSEKCDIFSLGVIMWELCTLT
RPWEGVPPERVVYAIAYEGARLEIPEGPLGKLIADCWTEPEQRPSCNEILSRLLDCEYSLC

>AT1G04700AtRAF16
MRMEFPGSSNQHLGRDRFNGEVGCGNNCSQTGEEFSNEFLRDFGAQRRLQHGGVNRNVEGNYNNRHLVYE
DFNRILGLQRVDSNMSEGINSSNGYFAESNVADSPRKMFQTAISDVYLPEVLKLLCSFGGRILQRPGDGKLRYIG
GETRIISIRKHVGLNELMHKTYALCNHPHTIKYQLPGEDLDALISVCSDEDLLHMIEEYQEAETKAGSQRIRVFLVP
STESSESPKIFHERNMNINRNTNQQTDIDHYQYVSALNGIVDVSPQKSSSGQSGTSQTTQFGNASEFSPTFHLR
DSPTSVHTWEHKDSNSPTFMKPYGNTNAVHFMPKMQIPRNSFGQQSPPTSPFSVHKRANTDVPYFADQNGF
FDPYLAAPNFPQQNRFFFETTTQKQKHPEVNLHDRRPSDDIYPHGQAYIGAEKMTLKKNALSDPQLHDESQIN
NGLEAFTKQPWKILRKNLRVVATSKWEDSDDIYFNNPEGKRCKELELTKEVPNSWINRDNNPDSFDQATKKQD
GSNSNSSFSPNYFSPNHQPAAQITSSDSQDSGSSVFSLSVNTNENYLDCSREKFNGFQHDMSLDILIRSHTSATD
QLCSTTKSSDKADYSSPNTNFPVVFLRQEPMIPRHDLETNSDDSDTQKSLPREESIHYSGLPLRKVGSRETTFMH
TQGSDDFFKSKLLGPQLIVEDVTNEVISDNLLSATIVPQVNRESDDDHKSYTREKEITNADHESEMEEKYKKSRN
TDDSFSEAAMVEIEAGIYGLQIIKNTDLEDLHELGSGTFGTVYYGKWRGTDVAIKRIKNSCFSGGSSEQARQTKD
FWREARILANLHHPNVVAFYGVVPDGPGGTMATVTEYMVNGSLRHVLQRKDRLLDRRKKLMITLDSAFGME
YLHMKNIVHFDLKCDNLLVNLRDPQRPICKVGDFGLSRIKRNTLVSGGVRGTLPWMAPELLNGSSNRVSEKVD
VFSFGIVMWEILTGEEPYANLHCGAIIGGIVNNTLRPPVPERCEAEWRKLMEQCWSFDPGVRPSFTEIVERLRS
MTVALQPKRRT

>AT1G14000AtRAF17
MSSDSPAAGDGGEQAAAGTSVPSPSYDKQKEKARVSRTSLILWHAHQNDAAAVRKLLEEDPTLVHARDYDKR
TPLHVASLHGWIDVVKCLLEFGADVNAQDRWKNTPLADAEGARKQKMIELLKSHGGLSYGQNGSHFEPKPVP
PPIPKKCDWEIEPAELDFSNAAMIGKGSFGEIVKAYWRGTPVAVKRILPSLSDDRLVIQDFRHEVDLLVKLRHPNI

VQFLGAVTERKPLMLITEYLRGGDLHQYLKEKGGLTPTTAVNFALDIARGMTYLHNEPNVIIHRDLKPRNVLLVN
SSADHLKVGDFGLSKLIKVQNSHDVYKMTGETGSYRYMAPEVFKHRRYDKKVDVFSFAMILYEMLEGEPPFAN
HEPYEAAKHVSDGHRPTFRSKGCTPDLRELIVKCWDADMNQRPSFLDILKRLEKIKETLPSDHHWGLFTS

>AT1G16270AtRAF18
MDRNRPPHPFQQHAMEPGYVNDSVPQGFTPDQTGLSNANVRPNPADVKPGLHYSIQTGEEFSLEFLRDRVIS
QRSANPIAAGDINYPTGYNGHAGSEFGSDVSRMSMVGNGIRQYERTNPPVHEFGNKLGHIHSAPEASLCQDR
SLGNFHGYASSSASGSLTAKVKVLCSFGGKILPRPGDSKLRYVGGETHIISIRKDISWQELRQKVLEIYYRTHVVKY
QLPGEDLDALVSVSCDEDLLNMMEEYNEMENRGGSQKLRMFLFSVSDLDGALLGVNKSDVDSEFQYVVAVN
DMDLGSRSNSTLNGLDSSSANNLAELDVRNTEGINGVGPSQLTGIDFQQSSMQYSESAPPTSFAQYPQSIPHN
GAFQFQQAVPPNATLQYAPSNPPSSSVHYPQSILPNSTLQYPQSISSSSYGLYPQYYGETEQFPMQYHDHNSSN
YSIPIPFPGQPYPHPGITQQNAPVQVEEPNIKPETKVRDYVEPENRHILATNHQNPPQADDTEVKNREPSVATT
VPSQDAAHMLPPRRDTRQNTPVKPSTYRDAVITEQVPVSGEDDQLSTSSGTCGLVHTDSESNLIDLDYPEPLQP
TRRVYRSERIPREQLEMLNRLSKSDDSLGSQFLMSHPQASTGQQEPAKEAAGISHEDSHIVNDVENISGNVVAS
NETLDKRTVSGGGIETEARNLSHVDTERSHDIPEKQTSSGVLIDINDRFPQDFLSEIFAKALSDDMPSGANPYQH
DGAGVSLNVENHDPKNWSYFRNLADEQFSDRDVAYIDRTPGFPSDMEDGGEIARLHQVAPLTENRVDPQMK
VTESEEFDAMVENLRTSDCEQEDEKSETRNAGLPPVGPSLADYDTSGLQIIMNDDLEELKELGSGTFGTVYHGK
WRGSDVAIKRIKKSCFAGRSSEQERLTGEFWGEAEILSKLHHPNVVAFYGVVKDGPGATLATVTEYMVDGSLRH
VLVRKDRHLDRRKRLIIAMDAAFGMEYLHAKNIVHFDLKCDNLLVNLKDPSRPICKVGDFGLSKIKRNTLVSGGV
RGTLPWMAPELLNGSSSKVSEKVDVFSFGIVLWEILTGEEPYANMHYGAIIGGIVNNTLRPTIPSYCDSDWRILM
EECWAPNPTARPSFTEIAGRLRVMSTAATSNQSKPPAHKASK

>AT1G62400AtRAF19
MEKKRFDSMESWSMILESENVETWEASKGEREEWTADLSQLFIGNKFASGAHSRIYRGIYKQRAVAVKMVRIP
THKEETRAKLEQQFKSEVALLSRLFHPNIVQFIAACKKPPVYCIITEYMSQGNLRMYLNKKEPYSLSIETVLRLALDI
SRGMEYLHSQGVIHRDLKSNNLLLNDEMRVKVADFGTSCLETQCREAKGNMGTYRWMAPEMIKEKPYTRKV
DVYSFGIVLWELTTALLPFQGMTPVQAAFAVAEKNERPPLPASCQPALAHLIKRCWSENPSKRPDFSNIVAVLEK
YDECVKEGLPLTSHASLTKTKKAILDHLKGCVTSISSPFSSSSVPVNA

>AT1G79570AtRAF20
MDKARHQQLFQHSMEPGYRNETVPQPFMPDQTGSASANMRPPNSNGSDVKAVHNFSIQTGEEFSLEFMRD
RVIPQRSSNPNGAGDMNYNTGYMELRGLIGISHTGSECASDVSRFSTVENGTSDIERTNSSLHEFGNKLNHVQS
APQALLSKDSSVGNLHGYKNTSSSASGSVTAKVKILCSFGGKILPRPGDSKLRYVGGETHIISIRKDISWQELRQKI
LEIYYQTRVVKYQLPGEDLDALVSVSSEEDLQNMLEEYNEMENRGGSQKLRMFLFSISDMDDALLGVNKNDGD
SEFQYVVAVNGMDIGSGKNSTLLGLDSSSANNLAELDVRNTEGINTIAGDVVGVGASQLMVNGFQQTSAQQS
ESIPPSSSLHYSQSIPLNAAYQLQQSVPPSSALHYPQSITPGSSLQYPQSITPGSSYQYPQSIIPGSASSYGIYPQYYG
HVVQHGERERFPLYPDHSSNYSAIGETTSSIPIQGHVSQQGGWAEGYPYPGSTPKSTQALAEEQKVSSDMKIRE
EVEPENRKTPGNDHQNPPQIDDVEVRNHNQVREMAVATTPPSQDAHLLPPSRDPRQNTTAKPATYRDAVITG
QVPLSGIEDQLSTSSSTYAPVHSDSESNLIDLNYPEPEQSSQRVYCSERIPREQLELLNRLSKSDNSLSSQFVTSESP
ANTAQQDSGKEAVGKSHDEFKTVNDDANHHTHKDVETIFEKVGVSDETLESEPLHKIVNPDDANKNRVVNGA
DTEIGVSNLSHVNAAMSHVIPEEQASLQGDILIDINDRFPRDFLSEIFSQAISEDTSTVRPYPHDGAAVSMNVQN
HDRKNWSYFQQLAEDQFIQRDVVLDQADSRIPSDRKDGGESSRLPYVSPLSRDGISTNLANPQLTLGQDYGGN
FSEKDGGGTGSIPPALENEQMKVTESEEFGAMVENLRTPDSEPKDEKTETRHAALPPLGSEFDYSGLQIIKNEDL
EELRELGSGTFGTVYHGKWRGSDVAIKRIKKSCFAGRSSEQERLTGEFWGEAEILSKLHHPNVVAFYGVVKDGP

GGTLATVTEYMVDGSLRHVLVRKDRHLDRRKRLIIAMDAAFGMEYLHSKNTVHFDLKCDNLLVNLKDPSRPICK
VGDFGLSKIKRNTLVSGGVRGTLPWMAPELLNGSSSKVSEKVDVFSFGIVLWEILTGEEPYANMHYGAIIGGIVN
NTLRPTIPGFCDDEWRTLMEECWAPNPMARPSFTEIAGRLRVMSSAATSTQSKPSAHRASK

>AT2G17700AtRAF21
MTIKDESESCGSRAVVASPSQENPRHYRMKLDVYSEVLQRLQESNYEEATLPDFEDQLWLHFNRLPARYALDVK
VERAEDVLTHQRLLKLAADPATRPVFEVRSVQVSPRISADSDPAVEEDAQSSHQPSGPGVLAPPTFGSSPNFEAI
TQGSKIVEDVDSVVNATLSTRPMHEITFSTIDKPKLLSQLTSLLGELGLNIQEAHAFSTVDGFSLDVFVVDGWSQE
ETDGLRDALSKEILKLKDQPGSKQKSISFFEHDKSSNELIPACIEIPTDGTDEWEIDVTQLKIEKKVASGSYGDLHR
GTYCSQEVAIKFLKPDRVNNEMLREFSQEVFIMRKVRHKNVVQFLGACTRSPTLCIVTEFMARGSIYDFLHKQK
CAFKLQTLLKVALDVAKGMSYLHQNNIIHRDLKTANLLMDEHGLVKVADFGVARVQIESGVMTAETGTYRWM
APEVIEHKPYNHKADVFSYAIVLWELLTGDIPYAFLTPLQAAVGVVQKGLRPKIPKKTHPKVKGLLERCWHQDPE
QRPLFEEIIEMLQQIMKEVNVVV

>AT2G24360AtRAF22
MLEGAKFNVLAVGNHHNNDNNYYAFTQEFYQKLNEGSNMSMESMQTSNAGGSVSMSVDNSSVGSSDALIG
HPGLKPVRHYSLSVGQSVFRPGRVTHALNDDALAQALMDTRYPTEGLTNYDEWTIDLRKLNMGPAFAQGAFG
KLYKGTYNGEDVAIKILERPENSPEKAQFMEQQFQQEVSMLANLKHPNIVRFIGACRKPMVWCIVTEYAKGGS
VRQFLTRRQNRAVPLKLAVKQALDVARGMAYVHGRNFIHRDLKSDNLLISADKSIKIADFGVARIEVQTEGMTP
ETGTYRWMAPEMIQHRAYNQKVDVYSFGIVLWELITGLLPFQNMTAVQAAFAVVNRGVRPTVPNDCLPVLSD
IMTRCWDANPEVRPCFVEVVKLLEAAETEIMTTARKARFRCCLSQPMTID

>AT2G31800AtRAF23
MANVVGQLKRGISRQFSTGSLRRTLSRQFTRQASHDPRRNNMRFSFGRQSSLDPIRRSPDGSNGPQLAVPDN
LDATMQLLFVACRGDVEGVQDLLDEGIDVNSIDLDGRTALHIAACEGHVDVVKLLLTRKANIDARDRWGSTAA
ADAKYYGNMDVFNILKARGAKVPKTKRTPMVVANPREVPEYELNPQELQVRKADGISKGIYQVAKWNGTKVS
VKILDKDLYKDSDTINAFKHELTLFEKVRHPNVVQFVGAVTQNVPMMIVSEYHPKGDLGSYLQKKGRLSPAKVL
RFALDIARGMNYLHECKPEPVIHCDLKPKNIMLDSGGHLKVAGFGLISFAKLSSDKSKILNHGAHIDPSNYCMAP
EVYKDEIFDRSVDSYSFGVVLYEMIEGVQPFHPKPPEEAVKLMCLEGRRPSFKAKSKSCPQEMRELIEECWDTET
FVRPTFSEIIVRLDKIFVHCSKQGWWKDTFKFPWK

>AT2G35050AtRAF24
MDQAKGYEHVRYTAPDPRDEGLGSINQRFSHDSSTNVNTYVRPPDYGVSTPARPVLNYSIQTGEEFAFEFMRD
RVIMKPQFIPNVYGEHSGMPVSVNLSALGMVHPMSESGPNATVLNIEEKRQSFEHERKPPSRIEDKTYHELVQS
APVISSKNDTGQRRHSLVSSRASDSSLNRAKFLCSFGGKVIPRPRDQKLRYVGGETRIIRISKTISFQELMHKMKEI
FPEARTIKYQLPGEDLDALVSVSSDEDLQNMMEECIVFGNGGSEKPRMFLFSSSDIEEAQFVMEHAEGDSEVQ
YVVAVNGMDLSSRRSSLGLSPPGNNLDELLHGNFDRKIDRAATEPAVASLTPLAGNESLPASQTSQPVTGFSTGN
EPFSQPYLGQQLQFPGLGNHQIYTSGHMASIGYIDEKRSAPLHVQPQPHYIPYSVNPETPLESLVPHYPQKPEQ
GFLREEQIFHVQDPETSSKEAKMRRDDSFQKVNDHPISTVESNLSAKEPKMRRESSTPRVNEYPVSSMPSDLIV
PDDLPKEEAPIVTQTSSSTPDPSSSTLSEKSLRKSEDHVENNLSAKEPKMRKEHSTTRVNEYSVSSVSSDSMVPD
QALKEEAPISMKISNSTPDPKSLVYPEKSLRTSQEKTGAFDTTNEGMKKNQDNQFCLLGGFSVSGHGTSNNSSS
NVSNFDQPVTQQRVFHSERTVRDPTETNRLSKSDDSLASQFVMAQTTSDAFLPISESSETSHEANMESQNVHP
TAPVIPAPDSIWTAEGSMSQSEKKNVETNTPEHVSQTETSAKAVPQGHNEKGDIVVDINDRFPREFLADILKTKE
SLNFPGLGPLHADGAGVSLNIQNNDPKTWSYFRNLAQDEFERKDLSLMDQDHPGFPTSMTNTNGVPIDYSYP

PLQSEKVASSQIHPQIHFDGNIKPDVSTITIPDLNTVDTQEDYSQSQIKGAESTDATLNAGVPLIDFMAADSGMR
SLQVIKNDDLEELKELGSGTFGTVYHGKWRGTDVAIKRIKRSCFIGRSSEQERLTSEFWHEAEILSKLHHPNVMA
FYGVVKDGPGGTLATVTEYMVNGSLRHVLLSNRHLDRRKRLIIAMDAAFGMEYLHSKSIVHFDLKCDNLLVNLK
DPARPICKVGDFGLSKIKRNTLVTGGVRGTLPWMAPELLSGSSSKVSEKVDVFSFGIVLWEILTGEEPYANMHYG
AIIGGIVNNTLRPTVPNYCDPEWRMLMEQCWAPDPFVRPAFPEIARRLRTMSSSAVHTKPHAVNHQIHK

>AT2G43850AtRAF25
MENITAQLKRGISRQFSTGSIRRTLSRQFTRQSSLDPRRTNMRFSFGRQSSLDPIRRSPDSSKSDDEPHMSVPEN
LDSTMQLLFMASKGDVRGIEELLDEGIDVNSIDLDGRTALHIAACEGHLGVVKALLSRRANIDARDRWGSTAAA
DAKYYGNLDVYNLLKARGAKVPKTRKTPMTVSNPREVPEYELNPLEVQVRKSDGISKGAYQVAKWNGTRVSVK
ILDKDSYSDPERINAFRHELTLLEKVRHPNVIQFVGAVTQNIPMMIVVEYNPKGDLSVYLQKKGRLSPSKALRFAL
DIARGMNYLHECKPDPIIHCDLKPKNILLDRGGQLKISGFGMIRLSKISQDKAKVANHKAHIDLSNYYIAPEVYKD
EIFDLRVDAHSFGVILYEITEGVPVFHPRPPEEVARMMCLEGKRPVFKTKSRSYPPDIKELIEKCWHPEAGIRPTFS
EIIIRLDKIVANCSKQGWWKDTFKFPWK

>AT4G14780AtRAF26
MEKKSEEDGNNTTKEKIFRADKIDLKSLDRQLEKHLSRVWSRNLEVNPKAKEEWEIDLAKLETSNVIARGTYGT
VYKGIYDGQDVAVKVLDWEDDGNETTAKTATNRALFRQEVTVWHKLNHPNVTKFVGASMGTTNLNIRSADS
KGSLPQQACCVVVEYLPGGTLKQHLIRHKSKKLAFKAVIKLALDLARGLSYLHSEKIVHRDVKTENMLLDAQKNL
KIADFGVARVEALNPKDMTGETGTLGYMAPEVIDGKPYNRRCDVYSFGICLWEIYCCDMPYPDLSFVDVSSAVV
LHNLRPEIPRCCPTALAGIMKTCWDGNPQKRPEMKEVVKMLEGVDTSKGGGMIPEDQSRGCFCFAPARGP

>AT4G18950AtRAF27
MEEDYQQPRFTIGRQSSMAPEKIPEPSVHSEEEVFEDGEEIDGGVRLMYLANEGDIEGIKELIDSGIDANYRDID
DRTALHVAACQGLKDVVELLLDRKAEVDPKDRWGSTPFADAIFYKNIDVIKILEIHGAKHPMAPMHVKTAREVP
EYEINPSELDFTQSKEITKGTYCMAMWRGIQVAVKKLDDEVLSDDDQVRKFHDELALLQRLRHPNIVQFLGAVT
QSNPMMIVTEYLPRGDLRELLKRKGQLKPATAVRYALDIARGMSYLHEIKGDPIIHRDLEPSNILRDDSGHLKVAD
FGVSKLVTVKEDKPFTCQDISCRYIAPEVFTSEEYDTKADVFSFALIVQEMIEGRMPFAEKEDSEASEAYAGKHRP
LFKAPSKNYPHGLKTLIEECWHEKPAKRPTFREIIKRLESILHHMGHKRQWRMRPLTCFQNFEHKKKHNWDLSS
HDGSSSGSHL

>AT4G31170AtRAF28
MLENPKFDLHAVGNHNNDNNYYAFTQDFYQKLGEEGTNMSVDSMQTSNAGGSVSMSVDNSSVGSSDALIG
HPGLKPMRHPYSLSDGQSVFRPGKVTHALNDDALAQALMDSKYPTEGLVNYEEWTIDLRKLHMGPAFAQGA
FGKLYRGTYNGEDVAIKLLERSDSNPEKAQALEQQFQQEVSMLAFLKHPNIVRFIGACIKPMVWCIVTEYAKGG
SVRQFLTKRQNRAVPLKLAVMQALDVARGMAYVHERNFIHRDLKSDNLLISADRSIKIADFGVARIEVQTEGMT
PETGTYRWMAPEMIQHRPYTQKVDVYSFGIVLWELITGLLPFQNMTAVQAAFAVVNRGVRPTVPADCLPVLG
EIMTRCWDADPEVRPCFAEIVNLLEAAETEIMTNVRKARFRCCMTQPMTVD

>AT4G35780AtRAF29
MAIKEETEESCGSRAVVASITKESPRQHRMKLEVYGEVLQRIQESNYEEANFPGFDDLLWLHFNRLPARYALDV
NVERAEDVLTHQRLLKLAEDPATRPVFEVRCVQVSPTLNGNSGDVDPSDPAVNEDAQSSYNSRSLAPPTFGSSP
NFEALTQAYKDHAQDDDSAVNAQLPNSRPMHEITFSTIDRPKLLSQLTSMLGELGLNIQEAHAFSTADGFSLDV
FVVDGWSQEETEGLKDALKKEIRKFKDQPCSKQKSITFFEHDKSTNELLPACVEIPTDGTDEWEIDMKQLKIEKK

VACGSYGELFRGTYCSQEVAIKILKPERVNAEMLREFSQEVYIMRKVRHKNVVQFIGACTRSPNLCIVTEFMTRG
SIYDFLHKHKGVFKIQSLLKVALDVSKGMNYLHQNNIIHRDLKTANLLMDEHEVVKVADFGVARVQTESGVMT
AETGTYRWMAPEVIEHKPYDHRADVFSYAIVLWELLTGELPYSYLTPLQAAVGVVQKGLRPKIPKETHPKLTELLE
KCWQQDPALRPNFAEIIEMLNQLIREVGDDERHKDKHGGYFSGLKKGHR

>AT4G38470AtRAF30
MVMEDNESCASRVIFDALPTSQATMDRRERIKMEVFDEVLRRLRQSDIEDAHLPGFEDDLWNHFNRLPARYAL
DVNVERAEDVLMHKRLLHSAYDPQNRPAIEVHLVQVQPAGISADLDSTSNDAGHSSPTRKSIHPPPAFGSSPNL
EALALAASLSQDEDADNSVHNNSLYSRPLHEITFSTEDKPKLLFQLTALLAELGLNIQEAHAFSTTDGYSLDVFVV
DGWPYEETERLRISLEKEAAKIELQSQSWPMQQSFSPEKENGQTGARTHVPIPNDGTDVWEINLKHLKFGHKI
ASGSYGDLYKGTYCSQEVAIKVLKPERLDSDLEKEFAQEVFIMRKVRHKNVVQFIGACTKPPHLCIVTEFMPGGS
VYDYLHKQKGVFKLPTLFKVAIDICKGMSYLHQNNIIHRDLKAANLLMDENEVVKVADFGVARVKAQTGVMTA
ETGTYRWMAPEVIEHKPYDHKADVFSYGIVLWELLTGKLPYEYMTPLQAAVGVVQKGLRPTIPKNTHPKLAELL
ERLWEHDSTQRPDFSEIIEQLQEIAKEVGEEGEEKKKSSTGLGGGIFAALRRSTTHH

>AT5G01850AtRAF31
MSSDDTIEESLLVDPKLLFIGSKIGEGAHGKVYQGRYGRQIVAIKVVNRGSKPDQQSSLESRFVREVNMMSRVQ
HHNLVKFIGACKDPLMVIVTELLPGMSLRKYLTSIRPQLLHLPLALSFALDIARALHCLHANGIIHRDLKPDNLLLTE
NHKSVKLADFGLAREESVTEMMTAETGTYRWMAPELYSTVTLRQGEKKHYNNKVDVYSFGIVLWELLTNRMP
FEGMSNLQAAYAAAFKQERPVMPEGISPSLAFIVQSCWVEDPNMRPSFSQIIRLLNEFLLTLTPPPPQPLPETAT
NRTNGRAITEFSIRPKGKFAFIRQLFAAKRNINS

>AT5G40540AtRAF32
MGSVTGFYSNEVFELDPKWVVDPQHLFVGPKIGEGAHAKIYEGKYKNKTVAIKIVKRGESPEEIAKRESRFAREV
SMLSRVQHKNLVKFIGACKEPIMVIVTELLLGGTLRKYLVSLRPGSLDIRVAVGYALDIARAMECLHSHGVIHRDL
KPESLILTADYKTVKLADFGLAREESLTEMMTAETGTYRWMAPELYSTVTLRHGEKKHYNHKVDAYSFAIVLWELI
HNKLPFEGMSNLQAAYAAAFKNVRPSADDLPKDLAMIVTSCWKEDPNDRPNFTEIIQMLLRCLSTISSTELVPP
AIKRVFSSENTVLPPESPGTCSLMTVRDKDQIPTDANSAQNEVRGSFFFFCC

>AT5G50000AtRAF33
MKEGKDGFVRADQIDLKSLDEQLERHLSRALTLEKNKKKDEEDTTAVAIGGSASSSPVTLNGGGFVGKRKQRLE
WEIDPSKLIIKTVLARGTFGTVHRGIYDGQDVAVKLLDWGEEGHRSEAEIVSLRADFAQEVAVWHKLDHPNVTK
FIGATMGASGLQLQTESGPLAMPNNICCVVVEYLPGGALKSYLIKNRRRKLTFKIVVQLALDLARGLSYLHSQKIV
HRDVKTENMLLDKTRTVKIADFGVARVEASNPNDMTGETGTLGYMAPEVLNGNPYNRKCDVYSFGICLWEIY
CCDMPYPDLTFSEVTSAVVRQNLRPDIPRCCPSALAAVMKRCWDANPDKRPEMDEVVPMLESIDTTKGGGMI
PNDQQQGCLCFRRKRGP

>AT5G50180AtRAF34
MDSLTGFRMEPKWQIDPQLLFVGPKIGEGAHAKVYEGKYKNQTVAIKIVHRGETPEEIAKRDSRFLREVEMLSR
VQHKNLVKFIGACKEPVMVIVTELLQGGTLRKYLLNLRPACLETRVAIGFALDIARGMECLHSHGIIHRDLKPENL
LLTADHKTVKLADFGLAREESLTEMMTAETGTYRWMAPELYSTVTLRLGEKKHYNHKVDAYSFAIVLWELLHNK
LPFEGMSNLQAAYAAAFKNVRPSAESLPEELGDIVTSCWNEDPNARPNFTHIIELLLNYLSKVGSPISAIPQRILAS
KNTLLPPDSPGTSSLMAKLDECGETPKAKSEDKRKGLFFCFNQCY

>AT5G57610AtRAF35

MDSGSVNSSVTSLVSSLNDEPHRVKFLCSFLGSILPRPQDGKLRYVGGETRIVSVNRDIRYEELMSKMRELYDGA
AVLKYQQPDEDLDALVSVVNDDDVTNMMEEYDKLGSGDGFTRLRIFLFSTPEQDGSLHYVERDDQRESERRYV
DALNNLIEGTDFRKLQQYPDSPRFNLVDDFSMVEPMLNQLSIETGGGSQRGNEIPTAQYSNLHQLRIPRVGSG
QMLAQRYGEVEGTWSPFYSPRHHGHHDPRTFQEFPSSPSSARYRMPYGEIPDKGLDRMPEEYVRPQASHHPF
YEHQAHIPDSVVWVPAGAMPPESKGGFPGNVLHGGPGGYEGGNGCENCRVPYHRNHQLLEQSNIGNNGFP
PVHCAHCPPNRESFLLNTDPKPTHHGAYPNETFGPDRGWMVQQQVNPNPPRIEEGRSHISNVGRPNDHYTP
DYPVSNYPLGQRAGHEISNEGFHDKPLGGIPLNSANRSAEERGFHYGNNLYPPGPDSIHSAGHSHMHHPQPNI
WQNVSNPIAGPPGLPMQINGTVNQTVIRNPIETAPRYSTGMENQGVLVGSPQRISGFDGMSSLGQPSYPNPH
LQDRAFPLDPNWVPSENPTVHNEHLQVREPLPGPLLQTNLTAAPIMQTPVMQTSVESKLAQGGEQFNYVNTG
ISNGVPYQDKPQPLAGGKKDMGNLVEVNPSAATLEGAELSVERLSFLPELMESVKRAALEGAAEVKAHPEEAK
DQVRPELVENESEHMNAQDEPEIDSDSDNPNNFKIEQTKAEAEAKSRGLQSIRNDDLEEIRELGHGTYGSVYHG
KWKGSDVAIKRIKASCFAGKPSERERLIEDFWKEALLLSSLHHPNVVSFYGIVRDGPDGSLATVAEFMVNGSLKQ
FLQKKDRTIDRRKRLIIAMDTAFGMEYLHGKNIVHFDLKCENLLVNMRDPQRPICKIGDLGLSKVKQKTLVSGGV
RGTLPWMAPELLSGKSNMVSEKIDVYSFGIVMWELLTGEEPYADMHCASIIGGIVNNALRPKIPQWCDPEWK
GLMESCWTSEPTERPSFTEISQKLRTMAAAMNLK

>AT5G58950AtRAF36

MDEEATSWIRRAKFSQTVSYRLNSSKLASLPFMINQDKFSGLKAIPQRSSSSSSASSSDPKLVSSNSQTTGDTSSL
EAADVYVVDSEIQTNPVTHKQRSVSPSPQMAVPDVFKEARSERKRFSTPHPRRVESEKGMKPKLSHKNSFDKR
SFNLRSPSGPIRDLGTLRIQERVKSKKDTGWSKLFDNTGRRVSAVEASEEFRVDMSKLFFGLKFAHGLYSRLYHGK
YEDKAVAVKLITVPDDDDNGCLGARLEKQFTKEVTLLSRLTHPNVIKFVGAYKDPPVYCVLTQYLPEGSLRSFLHK
PENRSLPLKKLIEFAIDIARGMEYIHSRRIIHRDLKPENVLIDEEFHLKIADFGIACEEEYCDMLADDPGTYRWMAP
EMIKRKPHGRKADVYSFGLVLWEMVAGAIPYEDMNPIQAAFAVVHKNIRPAIPGDCPVAMKALIEQCWSVAP
DKRPEFWQIVKVLEQFAISLEREGNLNLSSSKICKDPRKGLKHWIQKLGPVHAGGGGGSSSSGLGGSALPKPKFA

>AT5G66710AtRAF37

MRPRGYQRAPSMQKPTDYPTDKTLHPNYPFLMSSHGLKSFESDDEDDDSDSSNDQFAFTINTELLVDVKDISIG
DFIGEGSSSTVYRGLFRRVVPVSVKIFQPKRTSALSIEQRKKFQREVLLLSKFRHENIVRFIGACIEPKLMIITELME
GNTLQKFMLSVRPKPLDLKLSISFALDIARGMEFLNANGIIHRDLKPSNMLLTGDQKHVKLADFGLAREETKGF
MTFEAGTYRWMAPELFSYDTLEIGEKKHYDHKVDVYSFAIVFWELLTNKTPFKGKNNIFVAYAASKNQRPSVEN
LPEGVVSILQSCWAENPDARPEFKEITYSLTNLLRSLSSDTDATSSNSKANIATEDSTSSLVQERVVCDCPGLKMSK
TKKLKKKTNKLMNMIVPFLKIFKSCMSK

>AT3G01490AtRAF38

MKEKAESGGGVGYVRADQIDLKSLDEQLQRHLSKAWTMEKRKSLSDGEDNVNNTRHNQNNFGHRQLVFQR
PLLGGGYSNNNNSSKNDIIRSTEVEKSRREWEIDPSKLIIKSVIARGTFGTVHRGIYDGQDVAVKLLDWGEEGHR
SDAEIASLRAAFTQEVAVWHKLDHPNVTKFIGAAMGTSEMSIQTENGQMGMPSNVCCVVVEYCPGGALKSF
LIKTRRRKLAFKVVIQLSLDLARGLSYLHSQKIVHRDVKTENMLLDKSRTLKIADFGVARLEASNPNDMTGETGTL
GYMAPEVLNGSPYNRKCDVYSFGICLWEIYCCDMPYPDLSFSEVTSAVVRQNLRPEIPRCCPSSLANVMKRCW
DANPEKRPEMEEVVAMLEAIDTSKGGGMIPPDQQQGCFCFRRHRGP

>AT3G22750AtRAF39

METRNETKASPENNLRNRGADGNNSKKDMIFRADKIDLKNLDIQLEKHLSRVWSRSIEKHPKPKEEWEIELAKL

EMRNVIARGAYGIVYKGIYDGQDVAVKVLDWGEDGYATTAETSALRASFRQEVAVWHKLDHPNVTRFVGASM
GTANLKIPSSAETENSLPQRACCVVVEYIPGGTLKQYLFRNRRKKLAFKVVVQLALDLSRGLSYLHSERIVHRDVK
TENMLLDYQRNLKIADFGVARVEAQNPKDMTGETGTLGYMAPEVLDGKPYNRRCDVYSFGICLWEIYCCDMP
YPDLSFADVSSAVVRQNLRPDIPRCCPTALATIMKRCWEANPEKRPEMEEVVSLLEAVDTTKGGGMIPEDQRP
GCFCFVSGRGP

>AT3G24720AtRAF40
MEASVYGLQIIKNADLEDLTELGSGTYGTVYHGTWRGTDVAIKRIRNSCFAGRSSEQERLTKDFWREAQILSNLH
HPNVVAFYGIVPDGTGGTLATVTEFMVNGSLRHALLKKDRLLDTRKKIIIAMDAAFGMEYLHSKNIVHFDLKCE
NLLVNLRDPQRPICKVGDLGLSRIKRNTLVSGGVRGTLPWMAPELLNGSSTRVSEKVDVFSYGISLWEILTGEEPY
ADMHCGAIIGGIVKNTLRPPIPKSCSPEWKKLMEQCWSVDPDSRPPFTEITCRLRSMSMEVVTKSKRRENKP

>AT3G27560AtRAF41
MGSASGFYSNEEFELDPKWLVDPRHLFVGPKIGEGAHAKVYEGKYRNQTVAIKIIKRGESPEEIAKRDNRFAREI
AMLSKVQHKNLVKFIGACKEPMMVIVTELLLGGTLRKYLVSLRPKRLDIRLAVGFALDIARAMECLHSHGIIHRDL
KPENLILSADHKTVKLADFGLAREESLTEMMTAETGTYRWMAPELYSTVTLRQGEKKHYNHKVDAYSFAIVLWE
LILNKLPFEGMSNLQAAYAAAFKNLRPSAEDLPGDLEMIVTSCWKEDPNERPNFTEIIQMLLRYLTTVSAPQIIPP
PNRRVFSSENIVLSPESPGTCSLMSVRDGDVSRQTVNTADSSEKQTKGSFFSCCS

>AT3G46920AtRAF42
MAHEPSSPSSNLVSNPANLSASGLDYSSDLSNKRVSDGIISGFGSEQVSIDATNRNNPNLGNKRSDMDDEELEK
VKFLCSYNGKIIPRPSDGMLRYVGGQTRIVSVKKNVRFDEFEQKMIQVYGHPVVVKYQLPDEDLDALVSVSSSE
DIDNMMEEFEKLVERSSDGSGKLRVFLFDASSSEVDDSFGILEYGDGVDIGQRYVEAVNGVVVSKESVASGSSN
PNSDFSGVDVVDSLGVGQSDFVATTWTSSNFSPQTYHSNVSRLVPPDPRSSAYVVPMTVHADPPHSFQLETVS
EKPIVGKMQQQQQGYTTPSEHHPPAYVESRQEALRQPDIVHSPIQLLPSSTSLFSQQPFQDSPLSVSSHQFLPA
AHMSMAPLNSQISSTPVLINPVMQTQENLLGNYHAAQKLVPLPTEPRNTAYQGTISPGIPFDGYGGSQVPPSN
HVVLPDGSFYQQVTMAESFQRVNDCHMCQTSFPHMHSDPIMREGNDGSTMYVPYVSSAFYASRPDDIMRI
QQTDKFTGQQSFLNHSNHQERDTLHNANLATAQVETTEPFVNEIVRDVPIKVQVTRQQQHPVDPSVAYAQCR
ELSGLVDNVNIHAPEIYSNCQNFISPVDKIGKEDIMGTSSQQMARKNMFLHDTSGQSPVSPNIDHTDSAKRLTR
VVLPGHESQPKESCVPTQSPLLGNPGLYLQSLVGGQQFDSAEAQSSNPAYDVVESTFDAANLPSSLSSNPDAAN
LPSSLSSSVGGADHKESSKSLFSNQDPWNLQTNSNEDVKPDLLNSSKVILENDLLIGLWFSLKGSEEEHIKQELQN
VAEGVAASVLQSSTPSYHEPPIKVDEYAFNSKGEVSRNDEMKQQSTHFKDIRNQLLERLNFGYSGSDSLDQLQII
KDSDLEELRELGSGTFGTVYHGKWRGTDVAIKRINDRCFAGKPSEQERMIDDFWNEAQNLAGLHHPNVVAFY
GVVLDSPGGSVATVTEYMVNGSLRNALQKNVRNFDRCKRQLIAMDIAFGMEYLHGKKIVHFDLKSDNLLVNLR
DPHRPICKVGDLGLSKVKCQTLISGGVRGTLPWMAPELLNGTSSLVSEKVDVFSFGIVLWELFTGEEPYADLHYG
AIIGGIVSNTLRPQIPDFCDMDWKLLMERCWSAEPSERPSFTEIVNELRTMATKLPSKEQGSTQGPQS

>AT3G46930AtRAF43
MDGEVTSWIRRANFSHTVCYRMITPSLESMPFTVNQEKMQRNPVTNKKRSVSPLPHMALSDAFIEAKSDIKRF
STPHPRRVEPEKGMKAKSSSRKDSSEKKSVNLRSLSHSGPIRDLSTQKVKERGKSKIDKKSSKSVDYRGSKVSSAG
VLEECLIDVSKLSYGDRFAHGKYSQIYHGEYEGKAVALKIITAPEDSDDIFLGARLEKEFIVEATLLSRLSHPNVVKFV
GVNTGNCIITEYVPRGSLRSYLHKLEQKSLPLEQLIDFGLDIAKGMEYIHSREIVHQDLKPENVLIDNDFHLKIADF
GIACEEEYCDVLGDNIGTYRWMAPEVLKRIPHGRKCDVYSFGLLLWEMVAGALPYEEMKFAEQIAYAVIYKKIRP
VIPTDCPAAMKELIERCWSSQTDKRPEFWQIVKVLEHFKKSLTSEGKLNLLPSQICPELKKCPKFWIHIFGSFHHH

SSGGGSSSNNSALPKPKFA

>AT3G50720AtRAF44
MAISPTMMLNANYPFFMSAFGSDDNNDESDNQFDFNISRELLLNPKDIMRGEMIGEGGNSIVYKGRLKNIVP
VAVKIVQPGKTSAVSIQDKQQFQKEVLVLSSMKHENIVRFVGACIEPQLMIVTELVRGGTLQRFMLNSRPSPLDL
KVSLSFALDISRAMEYLHSKGIIHRDLNPRNVLVTGDMKHVKLADFGLAREKTLGGMTCEAGTYRWMAPEVCS
REPLRIGEKKHYDQKIDVYSFALIFWSLLTNKTPFSEIPSISIPYFVNQGKRPSLSNIPDEVVPILECCWAADSKTRLE
FKDITISLESLLKRFCSERSNNEITITEDEAYDDEIEELETTWLLPKRYIKLKKPKKIKQNVMKKILPFFKKFISSKW

>AT3G50730AtRAF45
MISRMIFRNYPSHNESDDEPFHFSISRELLLDRNDVVVGEMIGEGAYSIVYKGLLRNQFPVAVKIMDPSTTSAVT
KAHKKTFQKEVLLLSKMKHDNIVKFVGACIEPQLIIVTELVEGGTLQRFMHSRPGPLDLKMSLSFALDISRAMEF
VHSNGIIHRDLNPRNLLVTGDLKHVKLADFGIAREETRGGMTCEAGTSKWMAPEVVYSPEPLRVGEKKEYDHK
ADIYSFAIVLWQLVTNEEPFPDVPNSLFVPYLVSQGRRPILTKTPDVFVPIVESCWAQDPDARPEFKEISVMLTNL
LRRMSSDSSIGTTLPDGEAYEGEMEESENSPLLQEHFCKVKKPKEKKKKKKLVKMRFPFFKKFKVWLYNYKP

>AT3G59830AtRAF46
MDNIAAQLKRGISRQFSTGSMRRTLSRQFTRQNSLDPRRNNMRFSFGRQSSLDPIRRSPESLSCEPHMSVPEN
LDSTMQLLFMASKGDVNGVEELLNEGIDVNSIDLDGRTALHIASCEGHYDVVKVLLSRRANIDARDRWGSTAA
VDAKYYGNVEVYNLLKARGAKAPKTRKTPMTVGNPKEVPEYELNPLELQVRKVDGISKGTYQVAKWNGTRVS
VKIFDKDSYSDPERVNAFTNELTLLAKARHPNIVQFVGAVTQNLPMMIVVECNPKGDLSVYLQKKGRLSPSKAL
RFALDIARGMNYLHECKPDPIIHCELMPKNILLDRGGQLKISGFGLIKLSKIGEDSAKVVNHEAQIDKSNYYIAPEI
YKDEVFDKRADVHSFGVILYELTEGVSLFHPKPPEEVAESICIEGKRPTIRTKSKSYPPELKELIEECWHPEISVRPIFS
EIIIRLDKIVTNCSKQGWWKDTFKFPWK

>AT3G58760AtRAF47
MTIKPKSPARFKLGRQSSLAPESRTPIDTLTEDEDDDLAAAATAGIGDPTIRLMYLANEGDIDGINKMLDSGTNV
DYRDIDARTALHVAACQGRTDVVELLLSRGAKVDTKDRWGSTPLADAVYYKNHDVIKLLEKHGAKPTIAPMHV
LTDKEVPEYEIHPTELDFSNSVKISKGTFNKASWRGIDVAVKTFGEEMFTDEDKVNAFRDELALLQKIRHPNVVQ
FLGAVTQSTPMMIVTEYLPKGDLRQYLDRKGPLMPAHAVKFALEIARGMNYLHEHKPEAIIHCDLEPPNILRDD
SGHLKVADFGVSKLLVVKKTVKKDRPVVTCLDSSWRYMAPEVYRNEEYDTKVDVFSFALILQEMIEGCEPFHEIE
DREVPKAYIEDERPPFNAPTKSYPFGLQELIQDCWDKEASKRPTFRVIISTLELISDRIARKRSWKVMLGRCLPRFR
LFTKRDYVNPGGSNRSSGSFNR

>AT3G63260AtRAF48
MASGGGEADKSLEIGSGTADPKIGGTGSRSAGEERYFRADTLDFSKWDLHMGQTSTSSVLTNSASTSAPAPAM
QEWEIDLSKLDMKHVLAHGTYGTVYRGVYAGQEVAVKVLDWGEDGYATPAETTALRASFEQEVAVWQKLDH
PNVTKFIGASMGTSDLRIPPAGDTGGRGNGAHPARACCVVVEYVAGGTLKKFLIKKYRAKLPIKDVIQLALDLAR
GLSYLHSKAIVHRDVKSENMLLQPNKTLKIADFGVARVEAQNPQDMTGETGTLGYMAPEVLEGKPYNRKCDV
YSFGVCLWEIYCCDMPYADCSFAEISHAVVHRNLRPEIPKCCPHAVANIMKRCWDPNPDRRPEMEEVVKLLEAI
DTSKGGGMIAPDQFQGCLCFFKPRGP

>LOC107409320
mewtrgsmigrgstatvsvamdvpsgelfavkstelshskllqkeqnllsklsspfivkyrgfdirnecnqpiynlfmeyipqgtlyddiqr

hggrleeslirtytrqilqgleylhgnglvhcdiksqnilmgkknvkiadlgcarlvervpgngdfgtvtfcgtpvfmapevlrgeeqgfeadl
walgctiiematgkspwldvdnlvsalhrigfsddvpefpswlskkakdflgmclsrepkqrmtarellehpfleekdsqsdqvvneffm
nspnsvleslwdsfevngspqnpnhkgsssdsnsnsaaarieelidgatmdpsvsneanwtwsedwievrsnndeannefsdkme
vvlptnepplacslpdcssinieeelgrlgfdedflfkfsfghvkivrtergfvitfeitentivfrnknfmtqemkfflffn

>LOC107411974
mqdmlgsvrrslvfrtsspdgedspspigvgggptftlvdkinscirksrvfskpssplappspspppmakdntppirwrkgeligcgafg
rvymgmnldsgellavkqvliaantdskekaqahikeleeevkllknlshpnivrylgtvreeetlnillefvpggsissllgkfgsfpeavvrt
ytkqlllglaylhkngimhrdikganilvdnkgcikladfgaskqvvelatisgaksmkgtpywmapevilqtghsfsadiwsvgctviem
atgkppwsqqfqevaalfhigttkshppipehlsveakdfllkclqkepnlrpaasellqhpfvtgepmeshpafstsmmensetssps
ntkdletykmmtcletaddcnlgslncstvspeksfkskhlwrinssdddmcqiddsnditmgegrlnsslmpdnfksfnpmsepsd
dwvcklaaspeaecegmnldpdqkihlrggcaetfndrekdfsfpcgpslseddeelteskirafldekalelkklqtplyeefynsmng
acspsyvestcdettprylklppksrspsrgpmgtpsavvdahstgspgsngkrvsnicnasdqssqdipvidgqqepgspsmsfserq
rkwkeeldqelerkremmrqaglggktsspkdrainrqreltrfaspgr

>LOC107414154
mdwtrvrplgngssatvsladsrrfggfiavksvefsqskilqreqrilstlsspfvvsyigedvtlendnklmynlfmeyvpggtltdttirhg
grldesligyytrqvvqgleylhscglvhcdikgrnilvgedgakiadfgcsrwvnqpaeiagtpmfmapevargeeqgfacdiwalgct
viematggspwsnvgcdavgalyriafsgespefpsflseqakdflgqclkrnpkqrwtasqllkhpfleefnscskqikesnssscsptsil
drgiwnsleesetrccenenstvaddnrirrlamcsglpdwtsdqswvtirvnhnyddddedkvevvggsgmasvsyniedfeipap
svrkelvnlsysnvsdrngrdsfedfkncrkssvdcsnfifvrdkdkllsslisrc

>LOC107420999
mhhiprifnrskreksmdtkknprkprlerrnaakhidyeastssssmestpspslhsrsldlsdkmsfrvegiegefdiicrtlglsgiedf
aipsaawearkirsisdilprsrlnrldslsgsareelkdeviqavaelndrvvgsvrirggdnelpqadlaesrgccvandgacdgdfvggg
gggggggggggikgvrppvlkpppsmrlpvidktcstwdilrdfapdeetspaqllhktynssdeeeekeeekhhpreenlqeeeshq
eeeqvhteevvrqreesavrlpenallsescsfttsndddssssttteptsnispnarlrpiitywekgdllgrgsfgsvyegitddgyffavke
vslldqgsqgkqsvyqleqeiallsqfehenivqylgtdkdesklyiflelvtkgslqslyqkynlrdsqvsaytrqilhglkylhdrnvvhrdik
canilvsasgsvkladfglakatklndvhsskgtacwmapevvnrrnqgyglpadiwslgctvlemltrqipysdlewmqaifrigkgvlp
svpdslskdardfilqclkvnpndrptacqlldhpfvkrrlssssgsaspyhfggrs

>LOC107423026
mqdifgsvrrslvfrtqgvddnggfgglvekigtsirksriglfskqslralppapkkddtppirwrkgeligsgafgrvymgmnldsgellai
kqvliaansaskektqaqiweleeevkllknlshpnivrylgtaredeslnillefvpggsissllgkfgsfpesvirmytkqlllgleylhkngi
mhrdikganilvdnkgcikladfgaskkvvelatingaksmkgtpywmspevilqtghsfsadiwsvgctviematgkppwsqqyqev
aaifhigttkshppipehlsaeakdfllkclqeepnlrpsasdllqhpfvtleyqephrvfrtsfmesgnqmatpggnlrnssfnpairrstc
aglkdifdmgsvrcstvypenlsevsshwgpmncddemcqiddqddlmvsslvkfksasvsndltksfnpmseptddwpckfdesl
elarsgtdlspghtiqkaagspgasdkeetgfefahgplaaedddevteskirafldekaldlkklqtplyeefyntlnatdpqrvngimna
vnasnnwnlppksrspnqaprrrlsamvdaaktashgtvshskivsnasivhsralqeiqppqasewkglpldaqegsfspsasfserq
rrwkeelvqelerkremmrhaggktsspkdpvlirqrerlrvafpgk

>LOC107423632
mrnmpwwgksssreakkktskesligtwhrklrtssdskvscgsggarkqcsdtvsekgsrspqesrspspskqvarcqsfadrpsha
qplplpatvgrtdsgisistkprcekgskpsifmplprpvciqsrsnpndldldlvtaslssessvdsddatdsrhrspqatdyengtrtam

gspcslmpkdqssnaipinsrdsknsanpsfnnrisstspkqrplsshvpnlqvpyhgafcsapdssmsspsrspmrvfgaeqvmnta
fwaakpypditlhgsghcsspgsgqnsghnsmggdisaqffwqqsrgspeyspapsprmtspgpgsriqsgavtpihprsvgtpses
qtswpddgkqqshrlplppvavsnsspfahsnsaatspsvprspgraenpaspgsrwkkgkllgrgtfghvyvgfnsengemcamke
vtlfsddakskesakqlmqeiallsrlrhpnivqyygsetvgdkiyiyleyvsggsiykllqdygqfgelairsytqqilsglaylhakntvhrdi
kganilvdpngrvkladfgmakhisgqscplsfkgspywmapeviknsngcnlavdiwslgctvlemattkppwsqyegiaamfkig
nskeqpaipdhlsadgkdfviqclqrnpldrptaaklldhpfvkyaaplekpvsgpelsdaspgvingvkalgigqarnfstldsdrlavhs
srvlktnshasdihiprniscpvspigspllhsrspkylngrmspspissprttsgssspltggsgaipfnhlqqpaylqesygsvpkpsngfy
ingfshhdsshdifqgmqpgsqvfselapsendvlgkhfarpahgerydgqsvladcvsrqllrenvklkpspdlsptshlpshtncf

>LOC107424157
meqfrhigevlgslkaimvfrgniqinqkqcfflfdifcfayesiaeemehnlrfeekhtkwkileqplrdlyrifregetyirqcletkdwwa
kaitlyqnsdcvefhihnllccmpnvieaietageisgldqdvmqkkkhvysnkyrreyrdwklfqwrfgkqylitqdfcnridtvwkedr
wtllnkirekkisgstkyeqrlidlffksldgleplngrlfpcsilvgskdyhvrrrlgsgsqykeilwlgesfvlrhffgdietlsrqqeitsllslshp
nimhylcgftdeekkecflitelmnkdlssynkeicgprkkipfslpvavdlmlqiargmeylhskqifhghlnpsnilvrprgissegnlha
kvsgfglnsirrfttkspsnhngtlpfiwyspevleeqeqtgaaenskyteksdvysfgmvcfelltgkvpfedshlqgdkmsrniragerp
lftfqspkyvtnltkkcwhtdpnqrpsftsicrilryikrflamnpdhniqldppvphvdycdiesrllrnfpscqssvsqipfqmfvyrilek
ekisaslkdnsesgsdgasvcgddhittvddpfpsspiperkslasldvtnrklsikrspdsrlnklpgtprgrsvrppqmspcgrsmrmss
esqlmamsprirrtssghasdseis

>LOC107424505
mewvrgeeigsgsfatislavprkasaeipplmavksseifnsasinnekeildqladcpqvircfgenystekgeefhnlmleyasggslv
dqlkknggrlsesdirrytrtilkglsfihtkgfvhcdiklqnilvfengaakiadfglakktgekktevelrgtplymspesvndneyespsdi
walgcavvemmtgkpawncepgsnicallirigvgeelpqvpeelseegkdflgrcfvkdprkrwtadrllkhpfisdddtvsleelstsp
rspfdfpewvsmqstvrfsklsefspesenwfgreldsyfgssysssssstclenrlrrlmsdetpywlvsdswvtvr

>LOC107425633
mgkklpawwprkssknkeehrqnqlqhqnphgnhnsflkspirtdnkkakdkpksfdevfprgspraskdfgapggsssafsgfdsd
gpekrghplprpsvsstqsigidqavglgsgsfsgssvsssgssedhpvahdhgqfsafrgngdpkfnarsrspnpgsrgptsptsplhplf
sgmglesptgkqedgksqchrlplppgsptspsalsntrsgavtesasctvskwkkgkllgrgsfghvyvgfnsesgqmcaikevrlvsdd
qtskeslkqlhqeiyllsqlshpnivryygselaedtlsvyleyvsggsihkllqeygsfkepviqnytrqitsglaylhgrntvhrdikganilvd
pngevkladfgmakhitncssmlsfkgspywmapevvmntngyslavdiwslgctilematskppwsqyegvaaifkignskdipei
pdylsydakdfvrlclqreplsrptasqlldhpfirdqtttrvaninltkdafpyafdgsrtpvsp

>LOC107427543
mcsrnwkkikvvgsgaygtvhlaiplnlrlssktiavksstledseslqkeqrilehfsncseilqcygneltiengqrvynllleyanggdlm
dliesrqgrlpecevqiytrmilkgllrihkhgyvhcdlkpenilvfrsdfgvqlkisdfglskepgkeddffvnsssefrfrgtppymspesva
fgeiieapldiwslgcivigmisgefawpsldnkdlvrwlafsnkepelprnmsakgknflrkcfirnprerwtaemlldhpfvaeiedhpl
klesipscshsknfcnlslhqvsrpgqcgfm

>LOC107428154
mkrsreddnvdlldlphtmrmreehslsskgcswlrgsmigkggfgsiflaynniptsrfkdfphvmavksveatscselvkekilldivkd
cpfiiqsygedvtvgykgkiiinlfleyasggslmdfiekskgfglvesqvigptesilkgvkrihepgyvhyfglakaaqgltrrsqvkgtlmyl
speavnfgiqdqpsdiwalgcillymltgrnpwdlksgeenhelkerisedspeipngpkrlris

>LOC107428813
mtvdlnprwaiqnqeeeeeeeeeeengnnlgrlafpetallrtksilkgikriheagfvhcdlkpqnillvpqensstttpfvakiadfglaka aqgltrssevkgtlmylppeaenfgiqdqpsdiwslgctvlcmltgkiypwdlkswegnrelkqkisedcpeipnglskkakdflkqcfmr spydrptadmllghpficsglgrgddedkeelmsvhsvqasscklsephdefrasfipllnssfgeteevqgkitpvisdrlsssavcsaa

>LOC107429056
migkggfgsvflafnnkptssfkdfppvmavksveatscselvkekillqivkdspfiirfygedvtvgykgkiiinlfleyasggslmdlieks kasegvglvesqvkkitesilkgikriheagyvhcdlkpqnillvpqensstttpfvakiadfglakaaqgltrssevkgtlmylppeavnfgiq dqpsdvwalgcivlcmltgkiypwdlkswegnrelkqkisedcpeipnglskkaedfleqcfmrspydrptadmllshpficsglgkgd dedkeelmsvhsvqasscklseprdefrasfipllnssfgeteevqgkitpvisdrlsssavcsaa

>LOC107432528
mewsrgpiigrgstatvslatalrsselfcvksaelsssaflqreqsflsklscprivkylgfavsnennvplynlcmeyvpggtisdvirrhggk leesvirsytyqilqgldhlhvnglahcdiksqniligedgakiadlgcaklvgvdgsevfatsefsgtpvfmapevargeeqgfaadiwam gctviematgrcpwaeagdpvsalyrigysgdvpelprwfsdkgkdflskclmrnakerstakellrhpfvesmgchsdqvkeftmisp ssvldqelwdsleelessphepniegcsilsspmerfrmliggtfsfstppnvpnwsvdetwftvrcnidteifedesisdsssssmdstih eeeleslmfndglssdysfeivstignerdflipcnnmeitfvlenvkietyckrfcfiqsicysicvnphfilfiffhlliprfflylfphmlftsfnl atsign

>LOC107434197
mdgnhqwrcpvpgewvrgklvgsgsfgtihmalakstgrlfvvksaqsgigvqaleneadileslnsphivhcigkefsidkngeqqny nvfleymaggnlldvvhifggsldeqvirlytkeillglkylhdngivhcdlkcknvllsssgnvkladfgsakrmtkkttddegfvdswqniv gtplwmapevlrkkeldlasdiwslgctviematgnppwdveitsnpmaailkiacgndkpqfptkfsqvgldflakclerdprkrwka eellnhpfvsgensslrkssreevelvsfspasvldinvngillygegsdsdqeaetkteaedsrlinpfarrchegnwmaarqqrdnhfd ssenwitvr

>LOC107435014
mhhiprifnrskreksmdpkknprkprlerrnaakhidyeaststssssmestpspslhtrsmdlsdkmsfrvegidgefdvicrtlglsgie dfaipsaawearkirsisdilprsrlnrldslsgsareelkdeviqavaelkdrvvgsvrirggnnelpqadlaesrgccvandgacdgdfigg ggggggggggggikgvrppvlkpppsmrlpvidktcstwdilrdfapdeetspaqllhktynssdeeeekeeekhhpreenlqeeeshq eeeqvhteevvrqreesavrlpenallsescsfttsndddsssstteptsnispnarlrpiitywekgdllgrgsfgsvyegitddgyffavke vslldqgsqgkqsvyqleqeiallsqfehenivqylgtdkdesklyiflelvtkgslqslyqkynlrdsqvsaytrqilhglkylhdrnvvhrdik canilvsasgsvkladfglakatklndvhsskgtacwmapevvnrrnqgyglpadiwslgctvlemltrqipysdlewmqaifrigkgvlp svpdslskdardfilqclkvnpndrptacqlldhpfvkrrlssssgsaspyhfggrs

>LOC101223021
meagsrfysatdefrleakwlvdpkhlfvgprigegahakvyegkyknqtvaikivhkgetvdevakkearfarevamlsrvqhknlvkf igackepvmvivtelllggtlrkyllnmrprcldtrvavgfaldiarameclhshgiihrdlkpenllltadhktvkladfglareesltemmta etgtyrwmapelystvtlrqgekkhynhkvdaysfaivlwellhnklpfegmsnlqaayaaafknvrpsaenlpeelaiiltscwqedan arpnfsqiiqmllnylytisppepvipsriftsentvfppespgtsslmavrddsgdtpkakmennprgcffcsndcy

>LOC107403422
mdltegvgessspprsfgsysnydvrndvynrlvesgnedavsnpefreqldshfnrlpasygldvnmdrvedvllhqrllalakdpekr pvyhirflenistrtedngnqlftstlttprslcdaaneevpvshksnhaidfepcskledlnldvrnkskdveerytmvklprredvapipi

hevifstidkpkllsqlsallsdiglnireahvfsttdgysldvfvvdgwpvedtdglweamekavarsegswsrssqshsavekaltvqakl
gdweidrrllkmgeriasgscgdlyhgvylgqdvavkilrsehlndavedefaqevailrevqhknvvrfvgacsqsphlcivteympggs
lydylhknhnvmklpqllkfaidvcrgmeylhqnniihrdlktanllmdthnvvkvadfgvarfqnqggvmtaetgtyrwmapevinh
qpydqkadvfsfaivlwelvtakvpydsmtplqaalgvrqglrpdlpdnahpkllelmqrswdavpsnrpsfseiaaelenllqetpeaa
ngt

>LOC107404883
menfkypssppskrmrgaetidsegpyrllycsskgdtegvvqelekgvisnladydkrtalhlascegctevvvlllekgadvnsidrwg
rtplsdarsfghesickileanggidpvgldsqipcytidysevnmndailigegsygeiylvkwrgtevaaktirssiasnqrvknsfmkela
lwqklrhpnivqflgvlnncsrliflteylcngslhdilrkkgrldpqtavayaldiargmnylhqhkshaiihrdltprnvlqdaagrlkvtdfg
lskiaqekdvlgykmtgttgsfrymapevyrresygkgvdvfsfalivhemfqggpsnladdpeqvadkrayedsrpplssyvypepiri
llqkcwhknpdfrptfediilelesiqdkfqttepmpsscrcsil

>LOC107405634
melevkpqeqphpqagvarftlgkqsslapdrstdsagtavdevidprvklmylanegdlegikelldsgtnvnfsdidgrtalhiaacqg
lsdvvqlllhrgakvdpqdrwgstpladavyyknhdvikllekhgakppmapmhvenarevpeyeispseldfsnsvditkgtfriasw
rgiqvavktleeelftdedkvkaftdelallqkirhpnvvqflgavtqsspmmivteylpkgdlyaylkrkgalkpatavkfaldiargmnyl
hehkpeaiihrdlepsnilrddsghlkvadfgvskllkvantvkedrpvtcqdtswryvapevyrneeydtkvdvfsfalilqemiegcpp
fstkpekevpkayvanerppfrappkyyvyglkelieecwseepfnrptfrqiikrledintqlaqkrqwkavvvlqtdqhe

>LOC107405705
mkegsdgfvradqidlksldeqlerhmsraltmekskklrdeqgntnnfltstasttttttmsfltpkkqrqeweidpskliikgviargtfa
tvhrgvydgqdvavklldwgeeghraeaevaslraaftqevavwhkldhpnvtkfigakmgsaelqiqtdngligmpsniccvvveyla
ggtlksylirnmrkklafkivvqlgldlarglsylhsqkivhrdvktenmlldktrtvkiadfgvarveasnpndmtgetgtlgymapevlng
npynrkcdvysfgiclweiyccdmpypdltfsevtsavvrqnlrpdiprccpsslanvmkrcwdaspekrpemdevvsmleaidtskg
ggmippdqaqgclcfrkyrgp

>LOC107406505
mesgsrffsadefrldakwlidpkhlfvgprigegahakvyegkyknqtvavkivhngetpeeiakrearfarevamlsrvqhknlvkfig
ackepvmvivtelllggtrkyllntrprcldtrvavgfaldiarameclhshgiihrdlkpenllltadhktvkladfglareesltemmtaetg
tyrwmapelystvtlrqgekkhynhkvdaysfaivlwellhnklpfegmsnlqaayaaafknvrpsaenlpeevaliltscwqedpnar
pnfsqiiqmllnylytisppepvipsrifasentvlppespgtsslmavrddseetpkakmenksrglffcfnqcy

>LOC107406964
mvfvlhlvllqlshsisfwsigllfryknqtvavkivhngetpeeiakrearfarevamlsrvqhknlvkfigackepvmvivtelllggtlrkyl
lntrprcldtrvavgfaldiarameclhshgiihrdlkpenllltadhktvkladfglareesltemmtaetgtyrwmapelystvtlrqgek
khynhkvdaysfaivlwellhnklpfegmsnlqaayaaafknvrpsaenlpeevaliltscwqedpnarpnfsqiiqmllnylytisppe
pvipsrifasentvlppespgtsslmavrddseetpkakmenksrglffcfnqcy

>LOC107407393
mscseryrggeereyehqalrrsvdeaepnlvsqngslttqqltidenllvdpkvlfigskigegahgkvyegrycnrivavkvlhrgstsee
raslenrfarevnmmsrvkhenlvkfigackdplmvivtellpgmslrkylvslrpekldlhvaikyaidiarameclhangiihrdlkpdnl
lltanqksvkladfglareesvtemmtaetgtyrwmapelystvtlrqgekkhynnkvdvysfgivlwelltnrmpfegmsnlqaayaa
afkqerpslpedispdlafiiqscwvedpnlrpsfsqivrmlnsflftlspsqsslpdsddvneaaasnvsmndlsartrgkfafirhlfaakr

tknlq

>LOC107408109
menitaqlkrgisrqfstgslrrnlsrqftrqssldprrhnlrfsfgrqssldpirrspsddadltvpenldstmqllfmacrgdtlgvqdllddg
tdvnsidldgrtalhiaaceghvdvvklllsrkanidardrwgstaaadakyyghtevynilkargakapktrktpmavanprevpeyeln
plelhirksdgiakgtyqvakwngtkvavkildkdsysdsesinafkheltllekvrhpnvvqfvgavtqnmpmmivseyhpkgdlrsyl
qkkgrlspskalrfaldiargmnylheckpdpiihcdlkpknilldsggqlkvagfgvirlskispdkaklaqgggnidpsniyvapeiyrgeif
drsvdaysfglilyemiegallsqpkppeetlrmlcidgqrppfksksksyppdvkelieecwdpepvvrptfsevvvrldkivsncskqg
wwkdtfklpwk

>LOC107412267
mkhifkklhigsnhdpgrsnetsntsvtppqtcasdhrtvsgqnsgyppaspsssspspssastvsatgtgipfsatmnrsdymsseee
fqvqlalaisasnsdgrddpekdqiraatllslgthrmdsardkddaasealarhyweynvldyedkvvdgfydvhglsteaiqgkmpsl
adletnlgnsgfevllvnrtidpvleeliqiaqcialdypvsevtilvqklaelvmermggpvkdanfmlarwmerstelrtslhtsvfpigsi
tlglsrhrallfkvladnikmpcrllkgshytgvedgafnvikledereflvdlmaapgtliptdipsakdttfkpynpnssiipthyslndsgv
aysgaypplqgegssqnpafesssvlgrnsrsakaesvpafsgssvdntgsgvdntgsfkipnkvapsnqsdhlpssaigasrfkgnrgan
tvdggvrmnmnvvpynqnpedpknlfadlnpfqikgtgktsvynktienkvdeiqrprnnvipgrppaplmwkslhacnevprtke
ydymeglfprikrepndykvssastsntssekinndgckssdntsscqfnkltmaedgntnyegehprdakdlrsdtvdvakendeidf
ryhrkfthdtvlgahlklkdpespsssvessrnrvdkvfddvdvgeceipwedlvlgeriglgsygevyhadwngtevavkkfldqdfsga
alaefkrevrimrrlrhpnvvlfmgavtrppnlsiiteflprgslyriihrphhqidekrrikmaldvargmnclhasmptivhrdlkspnllv
dknwnvkvgdfglsrlkhntflsskstagtpewmapevlrnepsnekcdvysfgvilwelatmrlpwsgmnpmqvvgavgfqnrrlei
pkevdplvasiisecwqtepnlrpsfaeltvalkplqrlavpsnvdqpssplpqeisvnptqv

>LOC107412947
mcnkeiaclsqsennidhqhphqavylmdspsattnsapnyamspgsndecprvkflcsflgsilprpqdgklryvggetrivsvprdit
yeelmskmrelyegaavlkyqqpdedldalvsvvndddvtnmmeeydklgsgdgftrlriflfshpdqdgsshydgderdterryvda
lnnlndgsdirkqqpdspvisglddghvidqffnplslegglhnqrnelpipqynlhhltiphmgsgqhhqpisqryseleapwspayys
prhhghhdprtlaefpsspsaryrmpfvdlpdkcsdrmpeefsrqqvsprpsyehqpqysdnvgwlpsgaisgeksgfpgnifhtsn
vvegssicehcrmtfqrnqphfehpsmgsglhqvanpcpdcppretfmlnadtklhhgihpneqnnehrslfndsqnhergwilhq
qlnaradevrtnvsgagrlndhytvegpgmnlplshanmvdgrhvssnyvhhragpelgnevfhdqpvpgaplihvpppeesgiryg
nhpfvyggdnlypahghvpghvlwrnaqnpmnaapsyeasnappqvngkvnpvfprgtwegsprfcigvdnqnpwvessqkml
gfdgkavpdyayghalkvnlnilghenqhqfssdlvrpsqeipnsaspldpindlvrleeksipkekieenhlektdnsgvlaiccsgqaki
gdnncelaslesinsnclkttkesgddikpdgkdlsaspdvsklsvsrlsflpdliasakkaalegaeevkaeakedadnkknvstaketaa
kelesanvpgdseldsdcdnldtskieptkaeaeaiakglqtikndd leeirelgsgtygsvyhgkwkgsdvaikrikascfagrpsererli
adfwkealilsslhhpnvvsfygivrdgpdgslatvtefmingslkqflqkkdrtidrrkrliiamdaaigmeylhgknivhfdlkcenllvn
mrdpqrpvckigdlglskvkqhtlvsggvrgtlpwmapellsgksnmvtekidvysfgivmwelltgdepyadmhcasiiggivnntlr
pqiptwcdpewkslmescwasdpatrpsfseisqklrnmaaamnvk

>LOC107413171
memrttplrfalgkqsslapargsdrrnkpevdsggevnpgvrlmylaneadldgirelldsgidvnfrdidnrtalhiaacqgltdvvalll
qrgaevdpkdrwgstpladaiyyknqdvikllekhgakhvpapmhvrharevpeyeidpkeldftnsveidkgtfriaswrgtevavkk
lgdevlvdedkvkafrdelallqkirhpnvvqflgavtqsspmmivteylpkgdlrvflkrkgalkpttavrfaldiargmsylhenkpvpii
hrdleppnilrddsghlkvadfgvsklltvkedkplncletscryvapevfkheeydtkvdvfsfalilqemiegyppfctkqetevpkaya
ardrppfrapakrythglrdlieecwnenpakrptfrqiitkleiihnslshkrrwkvrplkcfqnieamlrkdrsnpssrsgssrssrssi

>LOC107414729
magscfhalrlrrskskplpvpstsktklnsdmdnlerkrfdsleswsmildsenvetwepskedqeewtadlsqlfignkfasgahsriy
rgiykqravavkmvripnqneetrslleqqfksevallsrlfhpnivqfiaacrkppvyciiteymsqgtlrmylnkkepyslstetilrlaldis
rgmeylhsqgvihrdlksnnlllndemrvkvadfgtscletqcqetkgnkgtyrwmapemikektytrkvdvysfgivlwelttallpfqg
mtpvqaafavaeknerppvpascqpalahlmkrcwaanpskrpdfsdivstlekydecvkeglplphhsglvsrnviqrlkgcvsmss
silvha

>LOC107415263
mgsgngvysvgefsldskwlidpkhlfvgprigegahakvyegkyknqtvavkivnkgetqeeiakkearfarevamiskvqhknlvkfi
gackepvmvivtelllggtlrkyllnmrprsldmgvavgfaldiarameclhshgiihrdlkpenllltadyktvkladfglareesltemmt
aetgtyrwmapelystvtlrhgekkhytnkvdaysfaivlwelihnklpfegmsnlqaayaaafknvrpsaenlpedlalivtscwkedp
nsrpnfsqiiqmllhylstisppepiiplrmfksenavlppespgtsslmatrddsgdtpknntedkpkgfffcfnqcy

>LOC107417160
meetrddagpaeqglpsaswwpsdfmerfgsvslgsqddslsntesprnseqdvlssqkasqilwrtgmlsepipngfysvipekslk
eifddipsldelhalwgegfkadvilvdgardkklsmlkqliaalvkglnsnpaamikkiaglvsdfykrpnvespakaaleetsnffenrgl
qmlgqirhgscrprailfkvladavglesrlmvglpsegaiecadsykhmsvivvlnsveflvdlmrfpgqlvrrstkaifmchisaagesds
aendscdsplepnsplygfserldpdstekdenlqfrrfdsnvpgpslrnmmlrtttaaerklslshsepniantfwrrsrrkviaeqrtas
sryyravramnetlkqnrllreqgddrsfshhaddgnrssvpqkndqissqkaislpssphdyrsqisgrsgpsgyvannelvsrwnkvl
esslfnnkpllpyqewnidfseltvetrvgigffgevfrgtwngtdvaikvfleqdltaenmedfcneisilsrlrhpnvilflgactkpphlsm
vteymemgslyylihlsgqkkklswrrrlkmlrdicrglmcihrmkivhrdlksanclvnkhwtvkvcdfglsrimtetpmrdsssagtp
ewmapelirnepftekcdifslgvvmwelctlsrpwegvpterviyavanegsrleipegplgrlisdcwaeqherpscedilarlpece
ynlr

>LOC107417666
mldggpkftgiiglnnhdydlsqgfyhklgegsnmsidsfaslqtsngggsvamsidnssvgsntndshtrilnhqglrrrandnysvaqs
vnrrgrvthalsddalaqalmdsnsptlglenfdewtidlkklnmgpafaqgafgklyrgtykgeevaikilerpendpekaqlmeqqfh
qevmmlatlkhpnivrfigacrkpmvwcivteyakggsvrqflmkrqnravplklavkqaldvargmayvhglglihrdlksdnllifgdk
sikiadfgvarievqtegmtpetgtyrwmapemiqhrpytqkvdvysfgivlwelitgmlpfqnmtavqaafavvnkgvrpivpndcl
pvlseimtrcwdanpdarppftevvrmlenaeteilttvrkarfrccmtqpmttd

>LOC107417677
mleggakytgivglnnrdnnyydlsqgfyhkleegtnmsidsfgslqtsndggsvamsvdnssvgsntndshtrilnhqglrrrpndncs
vqqsvnhrgkvthalsddalaralmdsnsltqglenyeewtidlrklnmgeafaqgafgklyrgtyngedvaikilerpendlekaqlme
qqfqqevkmlatlkhpnivrfigacrkpmvwcivteyakggsvrqflvkrqsravplklavkqaldvargmayvhglglihrdlksdnllifa
dksikiadfgvarievqtegmtpetgtyrwmapemiqhrpytqkvdvysfgivlwelitgmvpfpnmtavqaafavvnkgvrpiipnd
clpalgeimtrcwdanpdvrppftqvvrmleiaeteimttvrkarfrccmvqpmtad

>LOC107417903
meqsrinkqpqynstepgneelqphsqsfvrdpfssmhmntrppdpnmsevkpvlnysiqtgeefafefmrdrvnprkpllpdtvg
dpncapgylelkgmlginhgsesgsdismhriaekgpkqferknssshegrnnhasvpsvprsssgfesgrgvvrgyassgasdsssm
kikvlcsfggkilprpsdgklryvggetriicirkdiswqelmqkalsmcnqthiikyqlpgedldalvsvscdedlqnmmeecsdfakge
gskklrmflfsmsdledvqfglgsmdgdsevqyvvavngmdlaskknsslhglasslannldeldrqsteketnsapidsagissvpltgn

ivspvtiqssesmipssssafvikppiyhgkmvnsgenmqypfhdvhvppipsslvsssiplhvsmaqhggstegqqfsgsraensq
mpvkqvklksenpvpqestpekvfssgkaygvplqphdgnlmnyfpvenatvavtaseggphllssknevkyqepdrvassnnsvn
plqvpksseddfhsttfapgyggsesiaidltyfeqpvipqrvynseripreqaellnrstksddshgsqflishsrsdvsqqdpiaegvdkl
redgnqalqveqststakslhvdthmvddglarlqkygesadsvarmksellqgagsgskhelpkttdnkdvttsdrtlkseqetifpad
glkkhvidenselptvipkasvenpeeplsnqpvhpssevtgedpshddtfvdtqpfpwtkksakhvshdapstaissstqvdiedrfrr
dilsdifskaifsddspgvgllhkdgaglslnienhdpkrwsyfhklaqggflqkdfslidqdhpafssvlekveegdiksyhltpfttdgvlm
dhvdshmkvgeidpkefpaktaadiivvqsnydqsqtkdtesiqfsammenlrmpeleyedgkvesrnvglppldsslgdfdlgalel
qvikndsleelkelgsgtfgtvyhgkwrgtdvaikrikkscftgrsseqerltvefwreadilsklhhpnivafygvvqdgpggtlatvtefm
vdgslrhvllrkdryldrrkrliiamdaafgmeylhsknivhfdlkcdnllvnlkdplrpickvgdfglskikrntlvsggvrgtlpwmapelln
gssikvsekvdvfsfgivlweiltgeepyanmhygaiiggivnntlrptipsycdpewrrlmeqcwapnpaarpsfteiasrlrvmstlas
qtktqvqkpsk

>LOC107417907
mmdqsrtnkqlprnsiehgyeelqppsqsfvgdplsnmhantrppdpnisevrpvlnysiqtgeefafefmrdrvnprkpllpntmg
dpsyapgylelkgilgishaesergsevsmhsisqkgpehfenknssshggrinyssvqsvprtssgyessrgilhgytssgasdsssmk
mkvlcsfggkilprpsdgklryvggetrivrirkdvswqeltqrslsiynqtqvikyqlpgedldalvsvssdedlqnmmeectdigngegs
qklriflfsmsdldetqfslgsmdgdseaqyvvavngmdlgsrksstlhgfasssannledldrqsieketsraavdsvgvssvpfpgnivs
sstshspepvlpgsssayennppfyygnmmhygenmpyqlpdaritsihsplvpgsmpvpvvrdqqggstegqkfggsrvenvhm
pvkqvklksdgsvqqdgtnenvcmsgnayavlsqpyesklmdyspveeasvavtaseggl hllsknevkyqepekvsasndsvhllq
vpksseddhystsstafatgyagsesnamdlsyfeqpvlpqrvyyseripreqaemlnqgellnrlsksddshaseylvshsradisrqd
piaegvdktyedgnlappteqpsmtkplyvdthivdgglaklqkykefadsvtqnsellqdsdvdskhafpnpmdskdavkedrsdq
etirskdsheklpvdeipehahvnqktsvehqedptsdltrhhlseviakdpssddtmgdgqpfprsenlakrasqdapsigiststqvy
iedrfprdflsdifskavlsedspgvgllhndgaglslnvenhepkrwsyfrnlaqegynqndvslmdqdhpgysnvhgrveeedhiay
qhapltadralmdhvnsqtaaesivpcsdhphamdtesvqfgaimenrrmpesdyedgkfetrsaglppldpslgdiidistlqairnd
dleelkelgsgtfgtvyhgkwrgtdvaikrikkscftgrsseqerltvefwreadilsklhhpnvvafygvvqdgpggtlatvteymvdgslr
hvllrkdryldrrkrliiamdaafgmeylhsknivhfdlkcdnllvnlkdplrpickvgdfglskikrntlvsggvrgtlpwmapellngssnkv
sekvdvfsfgivlweiltgeepyanmhygaiiggivnntlrptipsycdpewrrlmeqcwapnplarpsfteiasrlrvmsamasqtktq
vqkask

>LOC107418405
mnlnwlkqisnngksgrrlslgeykravswskylvssgaeikgegqeqwsadmsqlfigfkfasgrhsriyrgiykqrdvaiklisqpeede
nlasllekqfisevallfrlqhpniitfiaackkppvfciiteylaggslrkylhhqephsvplelvlklaldiargmqylhaqgilhrdlksenlllg
edmcvkvadfgisclesqcgsakgftgtyrwmapemikekhhtkkvdvysfgivlwelltaltpfenmtpeqaafavsqknarpplps
acpraishlinrcwssnpdkrphfdeivsilegyaesleqdpgffsyynpspdranfrclskfvvfnrstsskvqkls

>LOC107418996
menppadellrkiqeleaghahlkqemsklmqsdsddvksehlrphpsyhhqrshsispqrsrfgsprrrvgagggggggfdgwgs
ckkgsasfrhssplqresrsreplnaggatgtgpsalnftdrqylnilqsmgqsvhifdpdgrviywnrsaenlygysaeealgqdaiellvd
prdydiannifqrvtmgeswtgqfpvknkmgdrflavvtntpfydddgtligiicvsndsrpfqetklefsdskhsessfnrprntvttklgl
dpqqplqvaiaskisnlatkvsnkvrsriragetnidreggsgdshhsdhgysdsvlsdhredatssgastprgdipqsafgifsqvdeksa
gkpsrdsgdenegksaihkiisskaeawigkkslswpwkgneregsevkssriifpwlqndqdndslhpksnvmkpensaneshrpa
nneasgswsssfnvnstssvsscgstsssavnrvdmdtdcldyeilwedltigeqigqgscgtvyhglwygsdvavkvfskqeysdevils
frqevslmkrlrhpnvllfmgavtspqrlcivteflprgslfrllqrnsskldwrkrvhmaldiargmnylhhfnppiihrdlkssnllvdrnw
tvkvgdfglsrlkhetylttktgkgtpqwmapevlrnepsdeksdiysfgvilwelatekipwdnlnsmqvigavgfmnqrleipkdvdp

qwasiiescwhsdpasrptfqelldklrelqrhytlqfqaarsatgdntqkel

>LOC107420099
mskmkhllrklhigggfndpqrlgdarpvtspnsnhpdpstsstasslssastsssttmagiatvesvadrlggdssgggvdfnfleeefq
vqlamaisasdpdarddpesaqidaakrislgcsaavtdspalvdflslrywnynvvnydekvvdgfydvygvtsdslaqgkmpllvdl
qaisvldnidyevllvnrmvdpelqrlekrayaiskeshvsrhglvlsgliqnianivvdrmggpvgdanemlrrwvvrryelrssmntiil
plgcldvglsrhrallfkvladtinlpcmlvkgsyytgtddgavnliklengseyiidlmgapgtlipaevpssqlqnsflgmrsfsdvkvmpt
gprflraggigplavskvgssrsdevsygdsgskdnertllgenpivrcenefgkplpsphrssqtslgtcgktslaqkkkvknvskyvisaak
dpefaqklhavllesgasppp dlfldissqdldegkvlgriqavdqeiasdgvhintdnlrsslerslipfsggensnyvsvdnmkkrapmv
ladkqneleiisnkskfaltsdtlsegfmllvnepsemtqtdavnidmassdpsnmytrssqeeqfnkpampsqvnsgqrhlenayv
nddnmivmervdngfvigssgqseginpvlgeaaeweipwedlrigerigigsygevyhaewngtevavkkfldqgfsgeallqfkce
veimlrlrhpnvvlfmgavtrpphfsilteflprgslyrllhrpnsqldekrrmkmahdvakgmnylhtshptivhrdlkspnllvdrnwv
vkvcdfglsrmkhhtflsskstagtpewmapevlrnepanekcdvysfgvilwelatcripwkglnpmqvvgavgfqnrrleipeevdp
vvaqimydcwqtepnlrpsfselmlrlrhlqrlvversnstner

>LOC107421353
mphrttyffprqfpdrrfdesskqlladhekkvstatastptsttttttpptptckdtisnfqiesdrkpskqfsvnskssavsqlftghgksht
kqhtqpqqvkpqqqqhfaafcdwlaekkaersttaahvkpprdedrellippppesvpevappesvvkdrsvdrnfdrqvslprvssg
ssyagslfsgttldgnlsidvkdsyskvssfstarheeveveeeeevdknrgslaqktkesyylrlalakrltsqafldhtepllmqvsgpess
naeivsyrlwvsgclsytdkisdgfynilgmnpyvwvmcndpeegkrlpplmslkaiepsetsmevvlidkrgdsrlkelqdkaqelyc
aaentlvlveklgklvaiymggtfpveqgdlhmrwklvskrlrhfqkcivvpigslsmglcrhrailfkkladyiglpcriargckycaadhrs
sclvkieddrqstreyvvdlvgepgnvhgpdssinggflssmpsplqsshlkefqepymdgasccqsvnskhtclptesplfagfgketqk
aeentfsnnlkgvtngpvyqatsgkesspmplemkgnfencviqtpvmpsfhgnqavkrsprkkksakqpklrvnlssesdaeeves
epdnggnfssvtiprylnlepslamdwleiswdelhikerigagsfgtvhraewhgsdvavkvltvqdfhddqlkeflrevaimkrvrhp
nvvlfmgavtkrphlsivteylprgslyrlihrpasgeildqrrrlrmaldvakginylhrlnppivhwdlkspnllvdknwtvkvcdfglsrfk
antfissksvagtpewmapeflrgepsneksdvysfgvilwelvtlqqpwsglspaqvvgavafqnrrlaippnaspvlvslmescwad
dpaqrpafssiveslkkllkspvqliqmegt

>LOC107422567
mtkearagtssqqfykdtpnvvsnnraaadrdvdnfciqtgeefsteflqdraglrrlapvmtnvnqrlptrsglnynqnhqqlayedls
gilglrrvdsecssevsdfvhgsgyfpaevdykvhpnninryhweygaaigqvpskhvdnrdrvppvlptappfyvldspqayhpyvq
gfsdgsvsskmrflcsfggrilprpsdgklryvggetriisirknitweelvkktstifsqphtikyqlpgedldalisicsdedlhhmleeyleq
ertegsqrlrlfliplnesespssvearatqtndvenqyvvavngmlepspkksssghsltsqtsqlgntlehsprfhrdsptstyilenkdh
sprppaqyttvlqgrdpknsdvidrpyaggdegsssflmgklpcqdscnvdalsrchglpltdnhnqtkylveadwanrasdicfhhpg
pggnfvssaqnvqtgvrfervmpressfhsnnsgshqdhlptllsgteikdvphnkmmhalsdsllqehydrpsdgfmplsssikirdk
llpitrssssrecvmqgvekaesqvavhenqftvkkpshsrgkgseellkwthrknssgdqknwnhhegnvdvksknnslessnlpni
nymhkncvssqelqipegmvsaspvtplenlldtrslnsmhdqqssttqriscqrslrrktpaasdefvgfeslatsskvastvssdseasl
hdkeamnrayneieilsfsgeaievpkfeyaisvqsqplddyhdneamespiiveditgiatpgipsssravayveytcsddectspretk
adssiqessgevendsadgrdesismsdaaiaeieagvyglqiiknddleelqelgsgtfgtvyhgkwrgtdvaikrikkscfsgrsseqer
likdfwrearilsnlhhpnvvafygvvpdgpettlatvtefmvngslrhvllkkdivldrrkrlliamdtafgmeylhlknivhfdlkcdnllvn
lrdserpickvgdfglsrikrntlvsggvrgtlpwmapellsgssnrvsekvdvfsfgivmweiltgeepyanmhcgaiiggivnntlrppip
krcdsewkklmeecwsadpasrpsyteitnrlrdmsmalpkkrqnvtsrrv

>LOC107422643

mtimgdtescgsravdfsptlsrkhkqkvdiydevlhrlrdlnvaeswlpgfedelwahfcrlptryaldvnvetaqdvlmhkrllhmar
npvtrpavevrlvqvrsscasnsygkveaqcsehvikqsihpppafglspdlefdanklyipesdgimnskhlyygpmheitistsdkpkl
lcqltsllseiglniqeahafstidgysldvfvvdgwaleeteqlretlakeipriekhsrlnyqaispvgeqehgvqfihnhlnippdendlw
eidasllkyekriasgssgdlykgtfcnqdvaikvlkaghlnetmqrefaqevyimrkvrhknvvqfigactrhpnlcivteymaggsmy
dflkensvlplqsllrvaidvskgmnylhqsniihrdlkaanllmdengvvkvsdfgvarvraqsgvmtaetgtyrwmapeviehkpyd
hkadvfsfgvvlwellagklpyenltplqaavgvvqkglrpkiprhahpilvellercwlqdpslrpefseivgilqnmakkteeragrkekq
sriipvsmqgrspsgivyrkf

>LOC107423093
mdsrtsdgvgsdktpmkmdnqkgiinskaedtgsfnskdmvfradkidlknldiqlekhlsrvltrntdpqkpeeewaidlskldiryv
vaqgtygivyrgtydnqdvavkvldwgedgiataaetaalrasfrqevavwykldhpnvtkfigasmgtsdlkipsknptdgnnshpsr
accvvveylpggtlkkflirnrrkklalkvvvqlaldlarglsylhskkivhrdvktenmlldahrtlkiadfgvarveaqnprdmtgetgtlgy
mapevldgkpynrrcdvysfgiclweiyccdmpypdlsfadissavvrqnlrpeiprccpsalanimkkcwdgnadkrpemdevvr
mleaidtskgggmipedqtpmcfclgrargp

>LOC107423594
menfkypssppskrmrgaetidsegpyrllycsskgdtegvvqelekgvepnladydkrtalhlascegctevvvlllekgadvnsidrw
grtplsdarsfghesickileanggidpvgldsqipcytidysevnmndailigegsygeiylvkwrgtevaaktirssiasnqrvknsfmkel
alwqklrhpnivqflgvlnncsrliflteylcngslhdilrkkgrldpqtavayaldiargmnylhqhkshaiihrdltprnvlqdaagrlkvtdf
glskiaqekdvlgykmtgttgsfrymapevyrresygkgvdvfsfalivhemfqggpsnladdpeqvadkrayedsrpplssylypepir
illqkcwhknpdfrptfediilelesiqdkfqttepmpsscrcsil

>LOC107424832
mqkrapamaehvtqtnkellrlslsysldtignsnrrltandetndsgnefvfnidpsllidphsvkigrligegphsivyeglykskpvavki
iqpmrssavsverkkrfqrevtlqskmqhenivkligasveptmvivtelmkggtlqkylwsirprpldpklsinfaldvsrvmeflhangi
ihrdlkpsnilltedmkqikladfglareeisgemtseagtyrwmapelfsldplpsggklqydhkvdvysfsivlwelltnktpfkgrnsv
mvayatakkirpclenipkdlvpllqscwgedpksrpefleitvslsnyqnllsidikateivetehpksntitkedpagtninpqsknavvk
vkrrrksspsflrcfdycsaq

>LOC107426395
mdsskgegvgdlekdeksnnregdgssgkakalglqgkddlgsiseknmlfradmidfkswdvqlekhlsrvwsrereactrkeewef
dlckldirylvaqgaygtvyrgsyngqdvavkildwgedgmatvaetaalraafrqevavwhkldhpnvtkflgasmgtsnlkipsnsts
sddqnslpsraccviveylpggtlknflirnrrrklaykiviqlaldlsrglsylhskkivhrdvktenmlldvgrnlkiadfgvarveaqnprd
mtgetgtpgymapevldgkpynrkcdvysfgiclweiyccdmpypnlsfadissavvwqnlrpeiprccpsslanimrkcwdanpek
rlemdevvrlleaidtskgggmipddqtpgcfcfapargp

>LOC107426719
mqlnrsenlfdtmtnevpgppgrwirqestsvvanvgknvhgknvlihtgeefsreyvqdsvsgrripsgintaqnhekkvpsvakars
hekrvsavtdmtqnhekkvgydcnqnhqlgyedlarilglrrmnsecasetseflsakgssreievdgyvdklsrcnneegdnghgsrk
pcgelncdtsgfgptgplnyksdshncnnfsgsgvldgsqseklkvlcsfggkilprpsdgklryvggetriisirkniswdelvkrtssicnqp
hsikyqlpdedldalisvssdedlqnmieeyhglerldgsqrlrifliplgesesttsfeagtlqqsnpnyqyvaavngmadpsprknagel
nlaseasqpetktsafpveiksnfnalnpnpflesqninrsynqsppvtpilhqhgdskiihmlphgdnscqgsyesnssfvstqlhpen
ssykyppagasssinyhyhdpykladvgyqdqphggnfnnlnlskelatplvvdqndgdfgglslerpvqkertfhsekpslrledpmgl
lsecndsvdsfrgiphafsdsklqesggksvtahcsqegmspssplnfakaqlslllnsgvsqekptklheninivnpweqnklvddgsle

ghkildlpnsspcceavcrkeptqkgtgntddkfqtsennlfksafmmpnpcekdsltletmkrtnavdplldqegklyggslltagvey
knklpnknpnpsftnvsedtiptssamdfkpmvdnlvehpincrhektvpdllgtsqivssdqncaltgrlidepgnsdsgtadpeipgl
gptvrrlshngnslsdlmpgssndsfvpepalvqpvasnmvheeplllssvnlhplqprgdpginsnlqdqkplqdagfkrevsllddf
vgypnskvenigfggssyeesnvadgknknqlesvvtvedvtdvvlpgiqssstctpynmdetigyvisptatevesvipesesedgkay
dedngpftdamiaemeasiyglqiirnadleelqelgsgtygtvyhgkwrgtdvaikrikkscfagrsseqerltkdfwreaqilsnlhhp
nvvafygvvpdgtggtlatvteymvngslrhvllkkdrsldrrkkliiamdaafgmeylhsknivhfdlkcdnllvnlrdpqrpickvgdfgls
rikrntlvsggvrgtlpwmapellngssnrvsekvdvfsfgismweiltgeepyanmhcgaiiggivkntlrppvpercdpewrklmeq
cwspdpeirpsfteitnrlrtmsialqakgpsnqtrqmkpnmss

>LOC107427400
mypgklsvhpppafgsspnlealaleankvddqdeehsvhahakfsrpmheitfssddkpkllsqltsllaeiglniqeahafstvdgysl
dvfvvdgwpyeeteqlrialerkllkiekqpwsdhqsisshvfepetvikcepdhltiphdgtdvweidlkhlkfenkvasgsygdlykgt
ycsqevaikilkperidtdmqrefaqevfimrkvrhknvvqfigactkppslcivtefmsggsvydylhkqkgvfklpsllkvaidvskgm
nylhqnniihrdlkaanllmdendvvkvadfgvarvkaqsgvmtaetgtyrwmapevcivtclnllflkf

>LOC107427772
mafdqnsipkdlrplnvartvaeepritpatttgrspdgffpssareigspdsvpvfypaavseagfvglgygnaapgvaawcplvpaav
ghqgvssavgygyspnlgtrvagaavdlinsgppmvsgsnpnlgnrigaggadhashdmgakhvygnrvgvvaadqtgndsatssg
yspnlggrtggsgtdqaseeggddsvsgkkvkflcsfggkiyprpsdgmlryvgghtriisvrrdvsfnelvqkmvdtygqpvvikyqlpd
edldalvsvscpddldnmmdeyekllerssdgsaklrvflfsaseldpsgvvqfgdlhdsgqryvdavngimdvvggnitrkesiasatst
qnsdfsgtevvdslgpgqgdvtgplsnsnlspkgnsdtshdtasklvfvdpshavyadasavpfgipvvksgppqtltsrpevelersvp
atlpqqqlglqqpgmeippptsyvqsyvdprqevvnhgdyihlspqmgfpsphllgtagpvftqqqfrdtgagmtphhfipavhmtl
npssscvgirpnmvqplvqpqqtqldsfvdertfgprvvqlpveqsynsyqvqvpsavvgggyswhqvppqehvifsdgsvphqqvi
ypekitrledcymcqkalphahsdtvvqgqkgspsssvsdsistyhslrlddnlrtqpvtrvmatgalgegtleqgieawpkvlghgdpq
tgniqpeatrlpqipegnhenerinlqqvdnidhpripvpqgvigrvadlqasngaflgtipqssqtdsvqqwsasaqcqvkqdtlvnki
vtrdmppvggvpvqtsecmvhespkeyssklpgvipkedsvdtcmsyeqlrpidarmetlrispsetyvnkehgklpvdkfrmeess
dhriqqvggrdvlldktfdkfetsnfipaemlpsssaespymhnsrliesyevaqppmwgnpgsyahsklgvhqmnpnevhygnpa
fagidsahltdrvrpsaewmddtlrlqskvgqtnaeallsnvqdssnslfsnqdpwnlhhdtqfppprpnrvpsrkelfspkdpvsenh
lgnsgelntmedgvqqpfgnmnrdvnseharsakgsaeeqikqelqavaegvaasvfqpstsanpdlrdknesgngskqdgdvens
daavqhneqhkdedvktkmpekanvgfpvsdgigrlqiimnsdleelrelgsgtfgtvyhgkwrgtdvaikrindrcfsgkaseqermr
ddfwneaikladlhhpnvvafygvvldgpggsvatvteymvngslrnalqkneksldkrkrlliamdvafgmeylhgknivhfdlksdnl
lvnlrdphrpickvgdlglskvkcqtlisggvrgtlpwmapellngssslvsekvdvfsfgivlwelltgeepyadlhygaiiggivsntlrpaip
escdlewkslmescwssepserpsfteianhlramaakippkgqnqpqqpsstqppvqk

>LOC107428906
mssdapesaasascsassdkdkqkekarvnrtslilwhahqndaaavrklleedrslvqardydkrtplhvaslhgwigvakclieygad
inaqdrwkntpladaegakkhsmiellksygglsygqngshfepkpvppplpnkcdweiepseldfsnsaiigkgsfgeilkahwrgtp
vavkrilpslsddrlviqdfrhevnllvklrhpnivqflgavtdkkplmliteylrggdlhqylkekgslspstavnfaldiargmaylhnepnv
iihrdlkprnvllvnssadhlkvgdfglsklikvqnshdvykmtgetgsyrymapevfkhrkydkkvdvfsfamilyemlegdpplstyep
yeaakyvaeghrpffrskglitelkelteqcwatdmnqrpsfleilkrlekikenlpsdhhwnifna

>LOC107428931
mssdapesaasascsassdkdkqkekarvnrtslilwhahqndaaavrklleedrslvqardydkrtplhvaslhgwidvakclieygad
inaqdrwkntpladaegakkhsmiellksygglsygqngshfepkpvppplpnkcdweiepseldfsnsaiigkgsfgeilkahwrgtp

vavkrilpslsddrlviqdfrhevnllvklrhpnivqflgavtdkkplmliteylrggdlhqylkekgslspstavnfaldiargmaylhnepnv
iihrdlkprnvllvnssadhlkvgdfglsklikvqnshdvykmtgetgsyrymapevfkhrkydkkvdvfsfamilyemlegdpplstyep
yeaakyvaeghrpffrskglitelkelteqcwatdmnqrpsfleilkrlekikenlpsdhhwnifna

>LOC107429777
matsqfqetpsakfedhpvtdstktinslshrnehshqislsglslyetsmkepplhhhhhlsrrkrptnnnnqlkklklvcsfngcfqtrp
psaklryvggetriisvdrnisflklrskiadlcpkttsfvlkyqlpesdpacfdsgtplvliasdddvrcmideydklelycknarlwvyvcsn
nggdnamvmnqlygnctgikglngleyetaiggggggvnngfeshsqkcenqvfgaqvdgkvvnnlggvrcdcgddslrtillkqrmv
akqsaqihssqgvwgfasaeneagtvccgenrkfdhplidlgpqqpqvsisrdeviqsfedldvldsktagthvprsenwvqfpaprpi
plnpmdgnlrvepnsstqclavncgsvfcngcwipsgslsalqgssnlkqlgsygegllcgfgakqdwrntgpvqmsnfnrenimpwg
avsvpcvdhltgvaypqnsmyngnrvcdgfrssirnhrfgvndsrnqrlcsyhaqdlrnmaemgnhraaarldgrnsmgksyyvlrp
ntsiakqgqsmrvfyphswrqrsvfpeqqvlngranmkdysmnkketllydvqysndklrdqrgpavspsrnskveesylayagacsr
lnsqilssrdaiessnpcmttmktradllsgsncekyeissqtlddnfgeiptsfdpihcyevanvsgfsnglihsngtelgynkkflgdvvav
dslsnhrndskdvqvhkeeiassvnsflgnpspssskevkppafspgsstdassdatlkaqskavdlmeegqhggsklssqnagemkk
nhvqegevqqdslcslsidektnnkeshksskvidgipsdlagfythlatrelqtikncdleyikelgsgtygsvyhgkwkgcdvavkrikp
scftdgtveedrlvadfwkeahmlgqlhhpnivafygvvtdgpvtnlatvteymvngslkqvfrrkdrsidrrkrliiamdaafgmeylh
gkgivhfdlkshnflvnmrdpqrpickigdlglskikqktlvsggvrgtipwmapellnsnnnlvtekvdvysfgivmwelltgeepyvn
mrsediiagiikgnlrpdiptwcdpawrslmekcwsrdpdsrpsfteiskelrtisatmnik

>LOC107430036
mriahpssgkppamaradekspvriyraledrcesleksherlkeqldklvnekkkeevavmmesdsvgvsseypfpvrirgcfvsgs
pyrsileslghavhvceastgeiiywnrsaenlygwknyevvghgvggeilipeehfvalqkiierlrrgqswsgqfpfkkrsgeifmaiatk
splyengvlvgfitvandaaafnrmearnrthedsansqhrgsqsylkriqwhghppiapvpeiassvsnlaskiflrghgddacniskdr
edsvtdtkdanakktgiqaakflaklhikgtdnsgkkddgttvqngssetlsmsnepnsksdskkptscgyivdtdykgdghhrkikisla
akryargheipnstedgsvlassreckecltglecdenskeaepevanlnavqiedgkqfsslggstdsngsssskgdnesntivdceihw
edlhlreeigqgscaivyhgiwngsdvaikvyhgnqytegilqdykkeidimkrlrhpnvllfmgaaysqerlaivteflprgslfrtlhknn
qaldirrrlrmaldvargmnylhhrnppivhrdlkssnllvdknwsvkvgdfglsklkngtfltgksargtpqwmapevlrnepsneksd
vfsfgvilwelmtesipwnhlnslqvvgvvgfmdrrldlpegldphvasviqdcwqsdpqqrpsfediiqrmmilqraaplptrkssep

>LOC107431473
maieeevescgsravsssqgqtrhhrqkldvynevlhriqesnfeeaslpgfddqlwlhfnrlparyaldvnveraedvlthkrllqlakd
hadrpaievrivqvypvdsdssmkedaqssfnnsggqgihppptfgsspnlealalqsnrylvedgdsamsetpyfprpmheitfsta
dkpkllsqltsllseiglniqeahafstvdgfsldvfvvdgwrceeteelrgalereiskykeqssskqnpgavdidigneqakleplcnvleip
tdgidvweidssllkfenklgsgsygdvykgsycsqevavkvlkpervtaemlgefsqevyimrkirhknvvqflgactqhpnlcivtefm
srgslydflhkqkgvfnlasllkvaidiskgmnylhqnniihrdlktanllmdenevvkvadfgvarvqaqsgvmtaetgtyrwmapevi
ehkpydhktdvfsfgivlwelltgelpysllltpvqaavgvvqkglrptipknthprvaelldrcwqqdptrrpnfseiieilkhiakegndkq
drnkdrsagrifsalkwvnh

>LOC107432147
mempgrrsnytllsqvpddqfgggsaamagtgattsyyesvsgegknnkgkvergfdwdavgdhranqqgnrmfssiglqrqssgss
fgesslsgeyyaptlsttaandmdaygylhedvfkigggggdlrakgvdgavgtggsswgkswaqqteesyqlqlalalrlsseatcadd
pnfldpvpdesalrssssssaeavshrfwvngclsyfdkvpdgfylihgmnpyiwtvctdlqengripsleslksvdpsiessieailvdrrs
dpslkelqnrvhgissgcintkevvdqlaklvcnrmggsatigeddfvslwressddlkeclgsvvvplgslsiglcrhrallfkvladtidlpcr
iakgckyctrydassclvrfgidreylvdligkpgclcepdsllngpssilissplrfprlkpvepiidfrslakqyfsdcqslnlvfdeastgpatd

gddskvsmypkqidrkcseesfllvtgqsddssligkvshpgakdrdsqlfktcnpaqnilhsttmakdpaigrreamrvdsskssrlfeg
sqlipskptkeftldiedlnipwcdlvlkerigagsfgtvhradwhgsdvavkilmeqdfhaerfkeflrevaimkrlrhpnivlfmgavtep
pnlsivteylsrgslyrllhksgaremlderrrlsmaydvakgmnylhrrnppivhrdlkspnllvdkkytvkvcdfglsrlkantflssksaag
tpewmapevlrdepsneksdvysfgvilwelatlqqpwsnlnpaqvvaavgfkgkrpeiprdlnshvasiieacwanepwkrpsfasi
meslrplikaptaqpgrpdm

>LOC107435406
meedasswirrtkfshtvchrwdssrlasvpfivqidwisglnsrptnretpgnhnenpsysqiqrnpitnkqrslsplpetllsdefkear
sdrkrfstpnrrrkeidkgivgnvfhkdshvskawnsstsplrhlasmkvnnklknrkespwakyfdhtggrvtaveaadewsvdmsq
lflglkfahgahsrlyhgiyfdepvavkiirvpdddesgalaarlekqfsrevhllsrlhhqnvikfkaacrkppvycviteylsegslraylhkl
ehkslplqklivialdiargmefihsqrvihrdlkpenilidrdfrlkvadfgiaceeaycdsladdpgtyrwmapelikhksygrkvdvysfg
lilwemvagtipyedmnpiqaafavvnknlrpdipgdcppamralieqcwslqpdkrpefwqivkvleqfesslaydgtlslvlnqtgh
dhkkklllwiqklgpvnsnslsmskpkprrkly

>LOC107435407
megevsswirrtkfshtvchrwdssrlasvpfnvqidrisglksrppnreasgnhkenpsysqiqrnpvtnkqrslsplpetllseefkear
sdwkrfstpnrrrkeidkgivgnvfhkdsqdsqvskalnsstsplrhlasmkvndksknrrespwakyfdhaagkvtaveaadewsvd
msklflglkfahgahsrlyhgiyfdepvavkiirvpdddesgalaarlekqfsrevnllsrlhhqnvikfkaacgkppvycviteylsegslray
lhklehkslplqkliaialdiargmefihsqhvihrdlkpenilidqdfrlkvadfgiaceetycdsladdpgtyrwmapelikhkcygrkvd
vysfglilwemvagtipfedmnpiqaafavvnknlrpiipgdcppamralieqcwslqpdkrpefwqivkvleqfvtslaydgtlslvlnq
tchdhkkqllrwiqklgpvhstsssmpkpkpkfy
