# Supplementary material for: Genome-wide identification of MAPKKK genes and their responses to phytoplasma infection in Chinese jujube (Ziziphus jujuba Mill.)
Source: BMC Genomics. 2020 Feb 10;21:142. doi: 10.1186/s12864-020-6548-6 (PMC7011567; doi:10.1186/s12864-020-6548-6)
Supplement: Supplementary file 3 — Additional file 3: Figure S2. Healthy and diseased in vitro plantlets. A: Healthy plantlets; B: Diseased plantlets. [file 12864_2020_6548_MOESM3_ESM.docx]

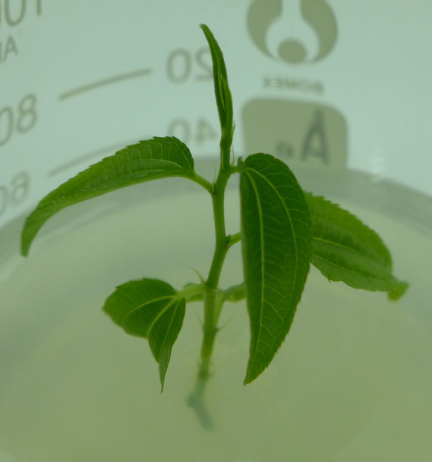

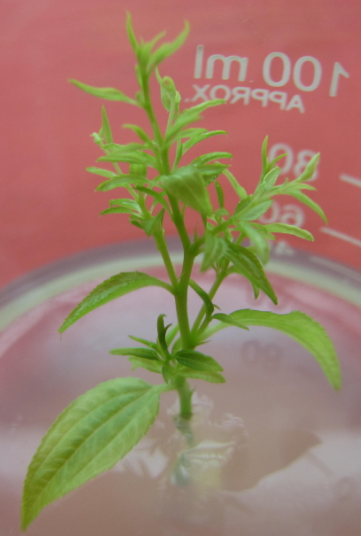


**A**

**B**

**Additional file 3: Fig. S2** Healthy and diseased *in vitro* plantlets. A: Healthy plantlets; B: Diseased plantlets.
