# Supplementary material for: Genome-wide identification of MAPKKK genes and their responses to phytoplasma infection in Chinese jujube (Ziziphus jujuba Mill.)
Source: BMC Genomics. 2020 Feb 10;21:142. doi: 10.1186/s12864-020-6548-6 (PMC7011567; doi:10.1186/s12864-020-6548-6)
Supplement: Supplementary file 4 — Additional file 4: Figure S3. Tissues showing different JWB disease symptoms. A: Witches’ broom leaves; B: Phyllody leaves; C: Apparently normal leaves; D: Healthy leaves. A, B and C were used as a test group collected from diseased trees. D was used as a control collected from healthy trees. [file 12864_2020_6548_MOESM4_ESM.docx]

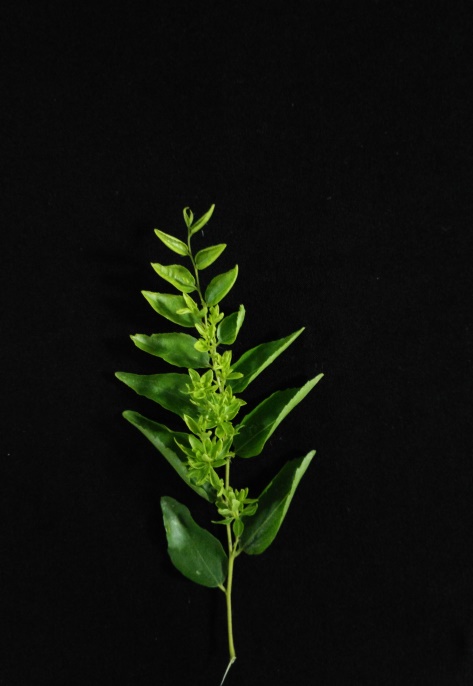

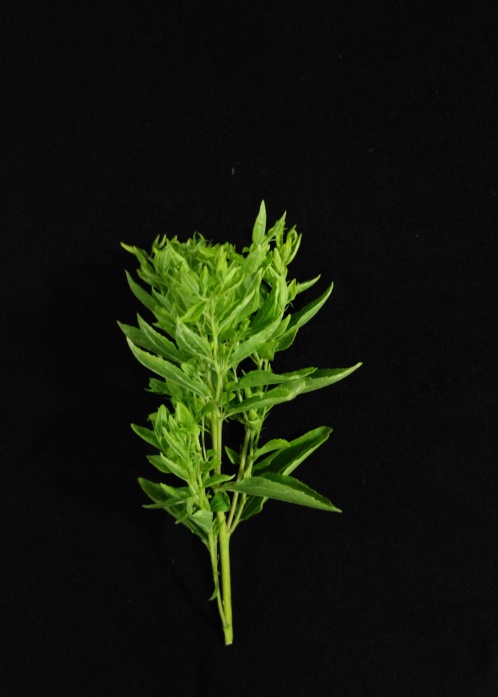

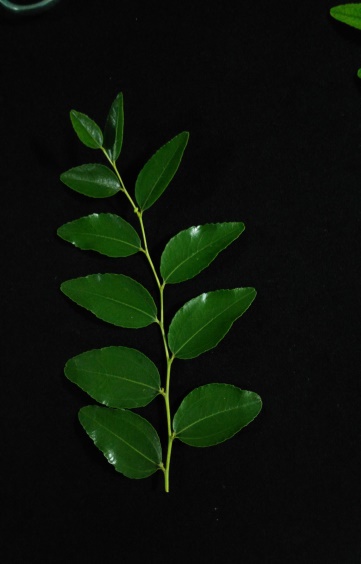

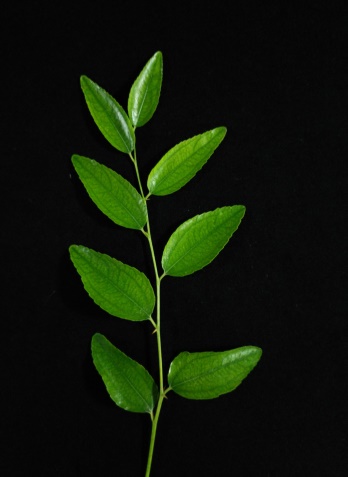

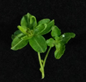


**A**

**B**

**C**

**D**

**Additional file 4: Fig. S3** Tissues showing different JWB disease symptoms. A: Witches’ broom leaves; B: Phyllody leaves; C: Apparently normal leaves; D: Healthy leaves. A, B and C were used as a test group collected from diseased trees. D was used as a control collected from healthy trees.
