# Supplementary material for: Genome-wide identification of MAPKKK genes and their responses to phytoplasma infection in Chinese jujube (Ziziphus jujuba Mill.)
Source: BMC Genomics. 2020 Feb 10;21:142. doi: 10.1186/s12864-020-6548-6 (PMC7011567; doi:10.1186/s12864-020-6548-6)
Supplement: Supplementary file 5 — Additional file 5: Figure S4. Determination of phytoplasma in the sieve element in jujube petiole phloem by using 4′,6-diamidino-2-phenylindole (DAPI). A, No fluorescent spots were observed in the sieve element (SE) of healthy plantlets. B, The fluorescent spots formed a large, bright circle in the sieve element (SE) of the diseased plantlets. The numbers and sizes of the fluorescent spots indicate the number of phytoplasmas. Bar = 100 μm. [file 12864_2020_6548_MOESM5_ESM.docx]

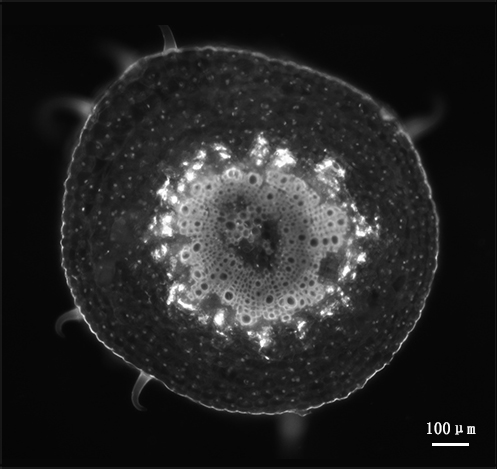


B


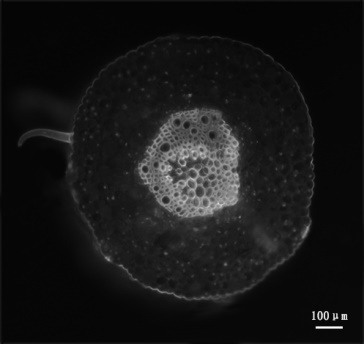


A

B

A

**Additional file 5: Fig. S4:** Determination of phytoplasma in the sieve element in jujube petiole phloem by using 4’,6-diamidino-2-phenylindole (DAPI). A, No fluorescent spots were observed in the sieve element (SE) of healthy plantlets. B, The fluorescent spots formed a large, bright circle in the sieve element (SE) of the diseased plantlets. The numbers and sizes of the fluorescent spots indicate the number of phytoplasmas. Bar = 100 µm.
