# Supplementary material for: Genome-wide identification of MAPKKK genes and their responses to phytoplasma infection in Chinese jujube (Ziziphus jujuba Mill.)
Source: BMC Genomics. 2020 Feb 10;21:142. doi: 10.1186/s12864-020-6548-6 (PMC7011567; doi:10.1186/s12864-020-6548-6)
Supplement: Supplementary file 7 — Additional file 7: Figure S5. CDS sequences of ZjMAPKKKs. [file 12864_2020_6548_MOESM7_ESM.pdf]

>LOC107412947 (MAPKKK1)
ATGTGCAATAAGGAAATTGCATGCTTAAGCCAGTCTGAAAACAATATTGATCATCAACA
TCCTCATCAGGCTGTGTACTTGATGGATAGCCCTTCTGCTACAACAAACTCGGCCCCTA
ATTATGCTATGTCACCTGGCTCAAATGATGAGTGCCCGCGTGTTAAATTTCTATGTAGTT
TTCTAGGTAGCATATTGCCTCGCCCACAGGATGGGAAGCTCCGGTATGTGGGCGGAGA
GACGCGAATCGTGAGTGTGCCTCGGGATATTACTTATGAGGAGTTGATGAGCAAGATGC
GAGAACTATATGAAGGGGCAGCAGTGTTGAAGTATCAGCAACCAGATGAAGATCTTGA
TGCTCTTGTCTCGGTTGTCAATGATGATGATGTGACTAACATGATGGAAGAGTATGACA
AGTTAGGTTCCGGGGATGGTTTCACCAGGCTCAGAATTTTCTTGTTTTCTCATCCCGAC
CAAGATGGTTCGTCACACTATGATGGGGATGAAAGGGATACTGAGAGGAGATATGTGG
ATGCCTTGAACAATTTGAATGATGGATCTGATATTAGGAAGCAACAACCTGACTCCCCG
GTGATTAGTGGCCTTGATGATGGCCATGTAATAGATCAGTTCTTTAACCCATTGAGCCTT
GAGGGTGGCCTTCATAACCAGAGGAATGAATTGCCCATCCCACAGTATAACTTGCATCA
CCTCACGATTCCTCATATGGGATCAGGGCAACACCATCAACCAATTAGTCAGAGGTATA
GTGAATTGGAAGCTCCGTGGAGTCCTGCATACTATTCTCCTAGGCATCATGGGCACCAT
GATCCTAGAACACTAGCAGAATTTCCTTCTTCCCCTTCTGCACGTTATCGTATGCCATTT
GTAGACTTACCTGATAAATGCTCAGATAGAATGCCAGAAGAATTTTCTCGGCAGCAAGT
AAGTCCCCGGCCTTCATATGAGCACCAACCTCAATATTCTGATAATGTTGGATGGCTTC
CAAGTGGAGCCATATCAGGTGAAAAGTCAGGTTTTCCAGGTAACATATTTCACACATCC
AATGTTGTTGAAGGAAGTAGTATCTGCGAGCACTGCAGGATGACTTTCCAAAGAAATC
AACCACATTTTGAGCATCCCAGTATGGGGAGTGGGCTTCACCAGGTTGCTAATCCGTGT
CCTGATTGCCCACCAAGGGAGACCTTCATGCTCAATGCTGATACAAAGTTGCACCACG
GAATTCACCCAAATGAACAGAATAATGAGCATCGATCATTATTTAATGACAGCCAAAAT
CACGAGAGAGGATGGATTCTGCATCAACAATTGAATGCTCGAGCTGATGAAGTAAGAA
CAAATGTATCTGGGGCTGGGAGATTGAATGACCATTATACTGTTGAAGGTCCTGGTATG
AATTTGCCTCTTAGTCATGCTAATATGGTAGATGGTCGTCATGTGTCTTCAAATTACGTT
CACCACCGAGCTGGACCTGAACTAGGAAATGAGGTGTTTCATGACCAACCTGTGCCTG
GTGCACCACTTATACATGTCCCTCCTCCTGAAGAAAGTGGTATTCGATATGGGAATCATC
CTTTTGTCTATGGAGGAGATAATCTTTATCCAGCTCATGGACATGTTCCAGGACATGTTT
TATGGAGAAATGCTCAGAACCCAATGAATGCCGCTCCATCTTATGAAGCATCCAATGCA
CCACCTCAAGTCAATGGTAAAGTTAATCCAGTATTCCCCAGAGGCACGTGGGAGGGTA
GTCCAAGATTTTGTATTGGAGTGGATAATCAGAATCCCTGGGTTGAATCCTCACAGAAA
ATGTTAGGTTTTGATGGGAAAGCGGTCCCAGACTATGCCTACGGCCATGCTTTAAAAGT
GAACCTCAATATACTTGGTCATGAAAATCAACATCAATTTTCTTCAGACCTTGTAAGAC
CCTCACAAGAAATTCCGAACTCTGCTAGTCCTTTGGATCCTATTAATGATCTTGTGAGGT
TGGAGGAGAAAAGTATACCTAAAGAAAAAATAGAGGAAAATCATTTAGAGAAAACTG
ATAACTCTGGTGTGCTGGCTATTTGTTGTTCTGGTCAAGCCAAAATTGGTGATAATAATT
GTGAGCTGGCATCTCTTGAGTCCATTAACTCAAATTGCTTGAAAACCACAAAAGAGAG
TGGTGATGACATCAAACCAGATGGAAAGGATCTTTCTGCTTCTCCTGATGTTTCAAAGC
TTTCGGTTAGCAGGTTGAGTTTCTTACCTGATTTGATTGCTTCTGCAAAAAAGGCTGCA
TTAGAAGGGGCTGAGGAAGTGAAAGCTGAAGCTAAAGAGGATGCAGACAATAAGAAG
AATGTCTCGACAGCCAAAGAAACAGCTGCTAAAGAATTAGAATCAGCGAATGTTCCTG
GGGATTCAGAATTGGATTCGGATTGTGATAATCTAGACACTTCCAAAATTGAGCCAACA
AAGGCTGAGGCAGAAGCTATTGCCAAGGGATTGCAGACAATAAAGAATGATGATCTGG

AGGAGATTCGAGAACTAGGTTCTGGAACATATGGGTCTGTTTATCATGGGAAGTGGAA
GGGTTCTGATGTAGCAATAAAGAGAATAAAAGCTAGCTGCTTTGCTGGGAGACCATCA
GAAAGAGAACGACTGATTGCAGACTTCTGGAAGGAGGCTTTGATACTAAGCTCACTAC
ATCATCCGAATGTCGTCTCCTTTTATGGTATTGTACGTGATGGTCCTGATGGATCTTTAGC
AACTGTAACAGAGTTCATGATCAATGGATCTCTGAAGCAGTTTTTGCAGAAAAAGGAC
AGAACAATTGACCGTCGGAAGAGACTCATCATAGCTATGGATGCTGCAATTGGGATGG
AGTATTTGCATGGGAAGAACATTGTACATTTTGATTTGAAATGTGAAAATCTGTTGGTA
AATATGAGAGATCCACAGCGGCCTGTGTGCAAGATTGGAGATTTGGGCTTGTCAAAGG
TAAAACAACATACTTTAGTGTCAGGAGGTGTTCGTGGAACTTTGCCCTGGATGGCACC
TGAGCTTCTTAGTGGGAAAAGTAACATGGTTACGGAGAAGATTGATGTTTACTCATTTG
GGATTGTTATGTGGGAATTACTCACTGGAGATGAACCTTATGCAGACATGCATTGTGCT
TCTATAATTGGAGGAATTGTGAACAACACATTACGTCCCCAAATTCCTACGTGGTGTGA
TCCTGAGTGGAAGTCTTTGATGGAAAGTTGTTGGGCCTCTGATCCAGCAACTAGGCCA
TCGTTTTCTGAAATCTCTCAGAAGCTGAGGAATATGGCTGCTGCAATGAATGTGAAATG
A

>LOC101223021(MAPKKK2)

ATGGAAGCTGGGAGTAGATTTTACTCTGCTACAGATGAGTTCAGATTGGAAGCCAAAG
GTTGGTTGATCCCAAACATCTATTTGTTGGACCTAGAATTGGAGAGGGAGCCCATGCCA
AAGTTTACGAGGGCAAATATAAGAACCAGACTGTTGCTATCAAAATTGTTCATAAAGGG
GAAACAGTTGATGAGGTTGCAAAGAAAGAGGCTCGGTTTGCTCGTGAGGTTGCAATG
TTGTCCAGAGTACAACATAAAAATCTTGTCAAGTTTATTGGTGCCTGCAAGGAGCCTGT
AATGGTGATAGTAACCGAACTTTTATTAGGAGGGACCTTACGAAAGTACCTGCTCAACA
TGCGCCCACGGTGCTTGGACACACGTGTGGCAGTTGGTTTTGCACTTGATATTGCTCGT
GCTATGGAATGCCTTCACTCACATGGCATCATACACCGTGATTTGAAGCCTGAGAACCT
TCTGTTGACAGCAGACCACAAAACAGTTAAATTAGCTGATTTTGGTTTGGCAAGAGAA
GAGTCGTTGACGGAGATGATGACTGCAGAGACAGGAACCTACCGTTGGATGGCTCCA
GAGTTGTACAGCACTGTTACCCTAAGGCAAGGGGAGAAGAAACACTACAACCATAAA
GTGGATGCCTATAGCTTTGCTATTGTGTTATGGGAACTGCTGCACAACAAGTTGCCATTT
GAAGGCATGTCAAATCTTCAAGCAGCATATGCAGCTGCTTTTAAGAATGTAAGGCCTAG
CGCAGAGAATCTTCCCGAGGAACTGGCTATCATTCTAACATCATGCTGGCAAGAGGATG
CAAATGCTCGTCCAAATTTCTCCCAAATTATCCAAATGTTACTTAATTACCTTTATACTAT
TTCACCCCCTGAACCGGTGATTCCTTCCCGAATTTTCACTTCTGAGAACACGGTTTTTC
CTCCTGAATCTCCTGGAACAAGCTCGCTGATGGCAGTCCGTGACGATTCAGGGGACAC
TCCCAAAGCTAAAATGGAAAATAATCCCAGAGGTTGTTTCTTCTGCTCCAATGACTGTT
ACTAA

>LOC107413171(MAPKKK3)

ATGGAGATGAGAACGACGCCGTTGCGTTTCGCACTGGGGAAGCAGTCGTCTCTGGCAC
CGGCCCGGGGCTCGGACCGCCGCAACAAGCCGGAGGTGGACTCCGGCGGGGAGGTG
AATCCCGGGGTGCGGCTGATGTACTTGGCCAACGAAGCTGACCTTGACGGCATACGCG
AGCTCTTGGATTCCGGCATCGACGTCAATTTCCGCGACATCGATAATCGGACGGCTCTG
CATATCGCTGCGTGCCAGGGCTTGACCGACGTCGTTGCGTTGCTGCTGCAGCGTGGTG
CTGAGGTTGATCCTAAGGACCGCTGGGGAAGCACTCCTCTTGCAGATGCTATATACTAT
AAAAACCAAGACGTGATCAAGCTTTTGGAGAAACATGGCGCTAAGCATGTGCCGGCG
CCTATGCACGTCAGACATGCCCGTGAAGTCCCCGAATATGAAATTGATCCAAAAGAACT

TGATTTCACCAACAGCGTTGAAATTGATAAGGGGACCTTCCGCATAGCATCATGGCGTG
GAACAGAAGTTGCTGTCAAAAAGCTAGGGGATGAAGTGCTTGTTGATGAGGATAAAGT
GAAGGCATTTAGAGATGAACTTGCATTGCTTCAGAAGATACGGCATCCAAATGTAGTCC
AATTTCTTGGTGCTGTGACTCAAAGTAGTCCAATGATGATTGTGACTGAATATTTGCCC
AAGGGTGATCTTCGTGTATTCTTGAAAAGAAAAGGGGCTTTAAAACCAACAACTGCTG
TCAGATTTGCACTTGATATTGCAAGGGGAATGAGTTATTTGCATGAGAATAAGCCGGTT
CCAATAATTCACCGTGATCTCGAGCCTCCAAATATATTGCGGGACGATTCTGGGCACC
TAAAAGTTGCAGACTTTGGAGTTAGCAAGTTGTTGACGGTTAAAGAAGATAAGCCTCT
GAATTGTCTAGAAACTTCTTGCCGATATGTTGCCCCGGAGGTTTTTAAACATGAAGAAT
ATGACACAAAAGTGGATGTATTCTCATTTGCTTTGATTCTGCAAGAGATGATTGAAGGC
TATCCACCCTTTTGTACTAAGCAAGAAACTGAAGTTCCTAAAGCATACGCTGCAAGAGA
TCGCCCACCTTTTAGAGCTCCAGCTAAGCGCTATACGCATGGGCTAAGAGATTTGATTG
AGGAGTGCTGGAATGAGAACCCAGCCAAGAGACCAACATTTAGGCAGATAATAACA
AAGCTGGAGATCATCCACAACAGTCTCAGTCATAAGAGGCGTTGGAAGGTTAGACCAT
TGAAATGCTTTCAAAATATAGAAGCTATGTTGAGGAAAGATCGTTCCAATCCAAGCAGC
CGCAGTGGCTCATCTCGATCTTCTCGCAGCAGCATATAG
>LOC107414729(MAPKKK4)
ATGGCGGGTTCATGCTTTCATGCACTTCGTCTTAGAAGGTCGAAGAGCAAGCCTTTGCC
AGTCCCTTCCACTTCGAAAACCAAGCTGAATTCTGACATGGACAATTTAGAGAGGAAA
AGGTTTGATAGCTTGGAATCTTGGTCTATGATATTGGACTCTGAAAATGTGGAAACTTG
GGAACCATCGAAGGAGGATCAGGAGGAGTGGACCGCTGATCTTTCGCAGCTTTTTATT
GGCAACAAATTCGCTTCAGGAGCACATAGTAGGATTTACCGTGGAATTTACAAGCAGC
GAGCTGTTGCTGTGAAAATGGTAAGGATTCCAAACCAGAATGAGGAGACCAGATCCTT
ACTTGAACAGCAATTCAAGTCTGAAGTTGCTCTGCTTTCGCGACTCTTTCATCCAAATA
TAGTGCAGTTTATCGCAGCTTGTAGAAAGCCTCCTGTCTACTGTATCATCACAGAATACA
TGTCTCAAGGAACGCTGAGGATGTATCTAAACAAGAAGGAGCCATACTCACTTTCAAC
AGAAACAATACTCAGGTTAGCTCTTGACATATCCCGAGGAATGGAGTACCTTCATTCGC
AAGGGGTTATCCACAGGGACCTCAAATCCAATAACTTGCTTCTTAATGATGAAATGAGG
GTTAAGGTAGCAGATTTTGGTACGTCGTGTCTTGAAACACAATGCCAGGAGACCAAGG
GAAACAAGGGAACTTATCGTTGGATGGCACCGGAGATGATCAAGGAGAAAACTTATAC
TAGGAAAGTTGATGTTTATAGCTTTGGGATTGTGCTATGGGAGCTCACAACCGCTTTGC
TTCCCTTCCAAGGAATGACCCCAGTGCAGGCTGCATTTGCTGTGGCTGAGAAGAACGA
GCGGCCTCCAGTGCCAGCTAGTTGCCAGCCTGCACTCGCACACTTGATGAAGCGATGC
TGGGCAGCAAACCCCTCGAAGCGGCCTGATTTCAGTGACATTGTGTCCACTTTGGAGA
AGTATGATGAGTGTGTGAAGGAGGGTCTTCCTCTTCCTCACCACTCAGGGCTGGTCAG
CCGGAATGTTATTCAACGCTTGAAAGGCTGTGTATCTATGAGCTCCTCCATACTTGTACA
TGCCTGA
>LOC107422643（MAPKKK5）
ATGACGATCATGGGAGACACAGAGAGTTGCGGTAGCAGAGCCGTTGATTTCTCGCCGA
CTCTGAGCCGAAAGCATAAACAGAAGGTTGATATCTACGACGAAGTTCTTCATCGACT
CAGGGACTTGAATGTGGCAGAGTCATGGCTTCCTGGTTTCGAAGATGAGCTCTGGGCT
CATTTTTGTCGCCTTCCTACTAGATATGCATTGGACGTGAATGTGGAAACCGCACAAGA
TGTTCTTATGCATAAAAGATTACTGCATATGGCACGTAATCCTGTTACTAGACCTGCAGT
TGAAGTCCGTCTTGTGCAGGTTCGTTCGTCTTGTGCCTCAAACTCATATGGAAAAGTGG

AAGCACAGTGTTCTGAGCATGTCATCAAACAGAGTATTCATCCACCACCAGCCTTTGGT
TTATCACCTGACCTCGAATTTGATGCAAACAAACTCTATATTCCAGAGTCCGATGGCATC
ATGAATTCCAAGCATCTATATTACGGGCCAATGCATGAGATTACAATTTCAACAAGTGAC
AAGCCAAAACTTCTCTGTCAGTTGACATCGTTATTGTCTGAGATTGGGCTGAACATTCA
AGAAGCCCATGCTTTTTCCACAATAGATGGCTACTCTTTGGATGTTTTTGTTGTTGATGG
TTGGGCACTTGAGGAGACTGAGCAGCTTAGAGAAACTCTGGCAAAAGAAATACCAAG
GATTGAGAAGCATTCGAGGTTAAATTATCAGGCTATTTCCCCTGTTGGAGAGCAAGAAC
ATGGAGTGCAGTTTATTCATAATCATTTAAACATACCACCTGATGAAAATGATCTCTGGG
AAATTGATGCAAGCTTGTTGAAATATGAAAAGAGAATAGCATCTGGTTCGTCTGGCGAT
TTGTATAAAGGCACTTTCTGTAATCAAGATGTGGCTATTAAAGTTCTAAAGGCTGGGCA
CCTGAATGAAACAATGCAGAGGGAATTTGCTCAAGAAGTCTATATCATGAGGAAGGTT
CGGCATAAGAATGTTGTCCAATTCATTGGGGCGTGTACCAGACATCCAAACCTATGCAT
TGTTACTGAATATATGGCTGGTGGAAGCATGTATGACTTTCTCAAAGAAAACAGTGTCT
TACCTCTTCAGTCCTTGCTCAGAGTAGCAATTGATGTTTCCAAGGGCATGAACTATTTG
CACCAAAGTAACATAATCCATAGAGACCTGAAAGCTGCTAATCTTTTAATGGATGAAAA
TGGAGTTGTTAAGGTTTCTGATTTTGGTGTTGCTAGAGTGCGAGCTCAATCTGGCGTGA
TGACTGCAGAAACTGGAACTTATCGTTGGATGGCTCCAGAGGTTATTGAACACAAACC
ATATGATCACAAAGCTGATGTCTTTAGCTTTGGGGTTGTGCTGTGGGAGCTGCTTGCCG
GAAAGCTTCCTTATGAGAACTTAACCCCGCTACAAGCAGCTGTCGGCGTGGTCCAGAA
GGGCCTGAGGCCTAAAATTCCAAGGCATGCTCATCCGATTTTGGTTGAACTGTTGGAG
CGGTGCTGGCTACAAGACCCATCATTAAGACCTGAATTCTCGGAAATTGTAGGAATTTT
ACAGAACATGGCCAAGAAGACGGAGGAGAGGGCTGGCAGAAAGGAAAAGCAATCAA
GGATAATACCAGTTAGTATGCAGGGAAGAAGCCCAAGTGGCATAGTTTATCGCAAATTT
TAG

>LOC107423632(MAPKKK6)

ATGAGAAATATGCCATGGTGGGGGAAGTCGTCGTCGAGAGAAGCAAAGAAGAAGACA
AGCAAGGAAAGTCTAATCGGTACATGGCACCGAAAATTGAGGACTTCATCTGATAG
TAAAGTGAGCTGTGGATCAGGAGGGGCTCGAAAACAATGCAGTGACACAGTTTCAGA
AAAGGGGTCTCGCTCCCCTCAAGAATCTAGATCTCCTTCTCCTTCTAAACAAGTGGCAA
GGTGTCAAAGTTTTGCTGATAGGCCTTCTCATGCTCAACCACTTCCACTCCCTGCAACC
GTGGGTCGTACAGATTCTGGAATTAGCATATCAACAAAGCCAAGATGCGAAAAGGGCT
CCAAGCCATCAATTTTTATGCCTCTCCCAAGGCCTGTATGCATCCAAAGCAGATCAAAT
CCTAATGATTTAGATCTAGATTTGGTTACAGCTTCACTGTCTAGCGAGAGTTCTGTGGAT
AGTGATGATGCAACTGACTCACGCCATCGTAGTCCACAAGCCACTGACTATGAAAATG
GGACTAGAACTGCCATGGGGAGTCCTTGCAGCTTGATGCCCAAGGATCAATCCTCAAA
TGCCATCCCAATAAACTCTAGAGATTCTAAAAACTCAGCTAACCCTTCTTTCAACAATC
GAATTTCCTCTACATCTCCTAAACAGAGGCCCTTGAGCAGTCACGTGCCAAACCTACA
GGTTCCTTATCATGGTGCATTCTGCAGTGCTCCAGACAGCTCCATGTCAAGTCCTTCCA
GAAGTCCAATGAGAGTATTTGGCGCTGAGCAGGTGATGAACACTGCTTTTTGGGCTGC
GAAGCCTTACCCCGACATCACTTTACATGGATCTGGCCACTGCTCAAGTCCTGGTTCAG
GTCAAAATTCTGGGCATAATTCTATGGGAGGGGATATCTCTGCACAGTTCTTTTGGCAA
CAAAGCAGGGGTAGCCCTGAGTACTCTCCAGCACCTAGTCCCAGAATGACTAGCCCTG
GCCCAGGTTCCAGAATTCAAAGTGGTGCTGTCACACCTATCCATCCTAGATCAGTTGGA
ACACCATCTGAGTCACAGACAAGTTGGCCTGATGATGGGAAGCAACAGAGTCACCGTT

TGCCTCTTCCTCCAGTGGCGGTTTCTAATTCTTCTCCTTTTGCTCATTCCAATTCTGCAG
CAACTTCTCCTTCTGTGCCGCGAAGCCCTGGAAGAGCAGAGAATCCAGCAAGTCCTGG
ATCACGCTGGAAAAAGGGAAAGCTATTGGGCAGAGGGACATTTGGACACGTTTACGTT
GGTTTTAACAGTGAGAATGGAGAAATGTGTGCAATGAAGGAGGTTACTTTATTTTCAGA
TGATGCAAAATCTAAGGAAAGTGCTAAGCAATTAATGCAGGAAATTGCTCTGCTCAGC
CGTTTGCGGCATCCAAACATTGTGCAGTATTATGGGTCTGAAACAGTGGGGGACAAAA
TTTATATATACCTGGAGTATGTATCTGGTGGGTCAATCTATAAACTTCTTCAAGACTATGG
ACAGTTTGGCGAGCTAGCAATTCGTAGTTATACTCAACAAATCTTGTCAGGCCTTGCAT
ATTTACATGCTAAAAACACTGTTCACAGGGATATCAAAGGAGCAAATATACTTGTGGAC
CCAAATGGCCGTGTTAAATTGGCAGACTTTGGGATGGCAAAGCATATCAGTGGGCAGT
CATGTCCTTTGTCATTCAAGGGAAGCCCATACTGGATGGCGCCTGAGGTTATAAAGAAC
TCAAATGGTTGCAACCTTGCTGTGGATATCTGGAGTCTTGGCTGCACTGTTTTGGAGAT
GGCTACAACAAAACCACCCTGGAGCCAGTATGAAGGGATTGCTGCCATGTTTAAGATT
GGGAATAGCAAGGAGCAACCAGCGATTCCAGATCATCTCTCAGCCGATGGAAAGGATT
TTGTAATCCAATGCTTACAACGAAATCCACTTGATCGTCCCACAGCTGCTAAACTTTTG
GATCACCCTTTTGTGAAATATGCTGCACCTCTGGAAAAACCTGTTTCGGGCCCAGAGCT
GTCAGATGCCTCCCCTGGTGTTATTAATGGAGTGAAAGCTCTGGGAATTGGGCAAGCTA
GAAATTTCTCAACCTTGGATTCAGATAGACTTGCAGTTCATTCCTCGAGAGTGTTGAAA
ACTAATTCCCACGCCAGTGATATCCATATTCCGAGGAACATATCATGCCCTGTCTCACCA
ATTGGAAGCCCTCTATTGCACTCAAGGTCTCCAAAATACCTAAATGGAAGAATGTCCCC
TTCTCCTATCTCTAGTCCACGAACCACTTCCGGTTCATCCTCTCCTCTGACAGGTGGCA
GTGGTGCAATTCCTTTTAATCACCTTCAACAGCCAGCATACTTGCAAGAGAGTTATGGA
AGCGTACCAAAGCCCTCAAATGGTTTTTATATTAATGGTTTCTCTCATCACGATTCAAGT
CATGACATCTTCCAAGGGATGCAACCAGGATCTCAAGTGTTCTCTGAACTGGCACCCA
GCGAGAATGATGTCCTAGGAAAGCATTTTGCAAGACCTGCCCATGGAGAACGGTATGA
TGGACAATCAGTTTTGGCTGATTGTGTGTCTCGGCAACTCTTGAGGGAAAATGTCAAA
CTAAAACCATCTCCGGACTTGAGCCCCACTTCTCATTTGCCCAGCCATACAAACTGTTT
CTAA

>LOC107427772（MAPKKK7）

ATGGCGTTTGATCAAAATTCTATTCCAAAAGATCTAAGACCGTTAAACGTAGCTCGAAC
TGTGGCCGAAGAACCCCGCATTACGCCAGCTACTACAACGGGTCGAAGCCCAGATGGT
TTTTTCCCCAGCTCAGCTCGTGAGATTGGAAGCCCTGATTCTGTACCTGTTTTTTATCCG
GCAGCAGTTTCGGAAGCTGGGTTTGTTGGTCTCGGATATGGAAATGCGGCTCCGGGTG
TGGCTGCTTGGTGTCCTCTTGTGCCTGCAGCAGTGGGGCATCAAGGTGTGAGCTCGGC
TGTTGGATATGGTTATAGTCCTAATTTGGGCACTCGAGTTGCTGGTGCTGCTGTAGACCT
TATAAACAGTGGTCCTCCTATGGTGTCTGGATCCAATCCGAATTTGGGCAATCGGATTGT
GCCGGTGGTGCAGATCATGCTAGTCATGATATGGGAGCCAAGCATGTCTATGGTAATAG
AGTTGGCGTTGTTGCTGCTGATCAGACAGGGAATGATTCGGCTACTAGCTCTGGTTATA
GTCCTAATTTGGGCGGTCGAACTGGTGGCAGTGGGACCGATCAAGCAAGTGAAGAGG
GTGGAGATGATTCTGTGTCAGGGAAGAAAGTAAAATTTTTATGTAGTTTTGGTGGAAA
GATTTACCCCAGACCTAGTGACGGGATGTTGAGATATGTTGGAGGGCATACAAGGATTA
TTAGTGTTAGAAGAGATGTGAGCTTCAATGAGCTTGTGCAGAAGATGGTGGATACCTAT
GGGCAACCAGTGGTTATAAAATATCAGCTACCTGATGAGGACCTTGATGCACTGGTGTC
TGTTTCATGCCCCGATGATCTTGACAATATGATGGATGAGTATGAAAAGCTTCTTGAAA

GGTCTTCAGATGGGTCGGCTAAATTAAGGGTGTTTCTGTTTTCTGCTTCAGAACTAGAT
CCATCTGGTGTGGTGCAGTTTGGGGATTTGCACGATAGTGGGCAGCGATATGTTGATGC
AGTGAATGGTATTATGGATGTGGTTGGTGGCAATATCACAAGGAAGGAGAGTATAGCAA
GTGCAACTTCAACGCAAAATTCTGATTTCAGTGGAACTGAGGTTGTTGATAGCTTGGGT
CCTGGCCAAGGGGATGTTACTGGACCTCTATCAAACAGCAATTTGTCGCCCAAGGGAA
ATTCAGATACTTCGCATGATACTGCTTCAAAGTTGGTGTTTGTGGATCCCAGTCATGCA
GTTTATGCTGATGCTTCTGCAGTACCATTTGGCATTCCTGTGGTTAAGTCTGGACCTCCC
CAAACTCTGACTTCTCGGCCAGAGGTTGAACTAGAAAGGTCTGTGCCTGCAACATTAC
CACAGCAGCAACTGGGTCTGCAGCAACCTGGAATGGAAATTCCACCTCCAACATCCTA
TGTCCAATCATATGTTGATCCTCGCCAAGAAGTTGTGAACCATGGAGATTATATTCATCT
CTCTCCTCAAATGGGGTTTCCAAGTCCTCATCTGTTGGGTACAGCAGGCCCCGTATTTA
CCCAACAGCAGTTCCGTGATACTGGTGCTGGCATGACGCCACATCATTTTATTCCTGCA
GTGCACATGACATTGAATCCCTCATCTTCTTGTGTTGGTATAAGACCAAATATGGTTCAA
CCTTTGGTGCAACCCCAGCAAACTCAGTTGGATAGTTTTGTGGATGAAAGAACATTTG
GTCCAAGGGTTGTCCAGCTTCCTGTTGAACAAAGCTACAATTCTTATCAGGTTCAGGTC
CCATCAGCAGTGGTAGGAGGTGGTTATAGCTGGCATCAGGTTCCACCGCAGGAGCATG
TAATTTTCTCTGATGGATCAGTACCCCACCAGCAGGTAATATACCCTGAGAAAATCACA
AGGTTGGAGGATTGTTACATGTGTCAGAAAGCATTGCCTCATGCACATTCTGATACTGT
GGTACAGGGCCAAAAGGGCAGTCCTTCAAGTTCTGTATCTGATTCAATCTCAACTTATC
ATAGTCTTCGTTTGGATGACAATCTGAGAACTCAGCCCGTGACTAGGGTTATGGCGACT
GGAGCATTAGGGGAAGGCACTCTTGAACAGGGAATTGAGGCTTGGCCTAAGGTCCTTG
GTCATGGGGATCCTCAAACTGGAAACATTCAACCAGAGGCAACTAGGCTTCCACAGAT
TCCTGAGGGAAATCATGAAAATGAAAGGATTAACCTGCAACAAGTTGATAATATTGATC
ATCCTAGAATTCCTGTTCCACAGGGTGTGATTGGGAGGGTTGCTGATCTGCAAGCATCT
AACGGTGCATTTTTGGGCACTATTCCTCAATCTTCTCAAACTGATTCTGTGCAGCAATG
GTCTGCTTCGGCTCAGTGCCAGGTTAAACAAGATACATTGGTGAATAAAATTGTTACTC
GGGACATGCCTCCTGTTGGAGGTGTGCCTGTTCAAACTTCAGAGTGCATGGTTCATGA
ATCTCCGAAGGAGTATTCTAGCAAACTTCCAGGTGTCATTCCAAAAGAAGATTCTGTAG
ACACTTGCATGTCATATGAGCAACTGAGACCAATTGATGCGAGGATGGAAACCCTCAG
GATATCCCCCAGTGAAACGTATGTCAATAAGGAGCATGGTAAGCTACCTGTTGATAAAT
TTAGGATGGAAGAGAGCTCTGATCATAGAATACAGCAAGTTGGAGGGAGGGATGTGCT
TCTTGATAAGACCTTTGACAAATTCGAGACTTCTAACTTTATTCCAGCTGAAATGCTCC
CCTCTTCCTCCGCAGAATCTCCATATATGCATAATTCAAGGCTCATAGAATCCTATGAGG
TGGCACAACCTCCTATGTGGGGTAATCCTGGATCTTATGCACACTCAAAACTAGGAGTG
CATCAAATGAATCCAAATGAAGTTCATTATGGCAATCCTGCATTTGCTGGAATTGACTCT
GCTCATTTAACTGATAGGGTAAGACCTTCTGCTGAATGGATGGATGACACACTAAGATT
GCAGTCTAAAGTAGGGCAAACAAATGCTGAAGCTCTTCTCTCAAATGTTCAGGATTCC
TCAAACTCACTCTTTAGCAATCAAGATCCCTGGAATTTGCACCATGACACCCAGTTTCC
ACCTCCAAGACCTAATAGGGTTCCCTCAAGAAAAGAACTCTTTTCTCCTAAAGATCCTG
TAAGTGAGAACCATTTGGGCAATAGTGGGGAACTAAACACAATGGAGGATGGAGTTCA
ACAGCCATTTGGCAATATGAACAGGGATGTCAATTCAGAGCATGCTCGATCTGCCAAA
GGCTCAGCAGAGGAACAGATCAAGCAAGAACTTCAAGCTGTTGCTGAGGGCGTAGCT
GCTTCTGTTTTTCAGCCATCTACATCTGCTAATCCTGACTTGCGCGATAAAAACGAATCC
GGTAACGGTTCTAAGCAAGATGGAGATGTTGAAAACAGTGATGCTGCCGTACAGCACA

ACGAACAGCATAAAGATGAGGATGTAAAAACTAAGATGCCAGAAAAAGCAAATGTCG
GTTTCCCAGTATCAGATGGCATTGGCCGCTTGCAGATTATAATGAACAGTGACCTTGAA
GAGTTGAGAGAATTGGGTTCTGGCACCTTTGGCACTGTTTATCATGGAAAATGGAGGG
GTACTGATGTAGCAATCAAACGGATCAATGATAGGTGCTTTTCTGGAAAAGCTTCAGAA
CAAGAGCGCATGAGAGATGATTTCTGGAATGAGGCCATTAAGCTTGCTGACTTGCACC
ATCCAAATGTGGTAGCTTTCTACGGTGTTGTGCTTGATGGTCCTGGAGGTTCTGTGGCT
ACAGTTACCGAGTATATGGTCAATGGTTCTCTAAGAAATGCTTTGCAGAAGAATGAGAA
GAGTCTTGACAAGCGTAAGCGTCTTTTAATTGCAATGGATGTGGCATTTGGAATGGAGT
ACCTGCATGGAAAGAATATAGTTCACTTTGATTTAAAAAGTGACAACTTGCTTGTAAAT
CTGCGAGATCCTCACCGTCCTATATGCAAGGTCGGTGATTTGGGACTATCAAAAGTGAA
ATGTCAGACATTGATATCTGGTGGTGTGCGAGGAACACTTCCATGGATGGCACCGGAG
CTTCTGAATGGTAGCAGCAGCCTTGTCTCTGAGAAGGTTGATGTATTTTCATTTGGCATT
GTGTTATGGGAGCTCCTTACTGGGGAAGAACCGTATGCAGATTTGCATTATGGGGCCAT
CATAGGTGGCATTGTAAGCAACACCTTACGGCCAGCCATTCCCGAATCTTGTGATTTAG
AATGGAAATCACTAATGGAGAGCTGCTGGTCATCAGAGCCATCCGAGAGACCAAGCT
TCACCGAAATTGCAAACCATTTACGAGCTATGGCAGCAAAGATCCCTCCCAAAGGTCA
AAACCAACCTCAGCAACCCTCCTCAACACAGCCTCCTGTTCAAAAATGA

>LOC107429777（MAPKKK8）

ATGATGGCAACTTCGCAATTTCAAGAAACTCCAAGTGCAAAATTTGAAGACCACCCAG
TCACAGACTCCACAAAAACTATAAACTCTCTCTCTCATCGCAACGAACACTCGCACCA
AATTTCCCTCTCTGGTCTCTCTCTCTACGAAACCTCCATGAAAGAACCTCCTCTTCACC
ATCATCACCATCTCTCTCGTCGCAAGAGACCCACCAATAATAATAACCAGCTCAAGAAA
CTCAAGCTCGTATGCAGCTTCAATGGCTGTTTCCAAACCCGTCCACCTTCAGCCAAGCT
CCGCTACGTCGGCGGCGAGACTCGTATCATCTCCGTCGACCGGAATATCAGCTTCTTGA
AGCTCCGATCAAAGATTGCGGATCTATGCCCCAAAACCACCTCCTTCGTCCTCAAATAC
CAGCTTCCCGAATCGGACCCTGCCTGTTTCGACTCCGGTACTCCTCTGGTCCTGATCGC
CTCCGACGACGACGTTCGCTGCATGATAGACGAGTACGATAAACTCGAGCTTTACTGTA
AGAATGCCAGGCTCTGGGTTTACGTGTGTAGCAATAATGGCGGTGATAATGCGATGGTG
ATGAATCAATTGTATGGGAATTGTACGGGAATTAAGGGTTTGAATGGGCTAGAATATGA
AACTGCAATTGGTGGTGGCGGTGGTGGTGTTAATAATGGGTTTGAGTCTCACTCTCAAA
AATGTGAGAACCAGGTGTTTGGAGCTCAGGTTGATGGTAAGGTTGTTAACAATTTAGG
TGGAGTACGGTGTGATTGTGGTGATGATTCGCTCAGAACAATTCTGCTGAAACAGCGA
ATGGTGGCAAAACAATCGGCTCAAATTCATAGCAGTCAAGGTGTTTGGGGTTTTGCTTC
TGCTGAGAATGAAGCCGGAACTGTATGCTGCGGTGAAAATCGAAAGTTTGATCACCCA
CTGATAGATTTAGGACCTCAACAACCCCAAGTCTCAATTAGCAGAGACGAGGTGATTC
AGAGTTTTGAGGACCTCGACGTGTTAGATAGCAAAACAGCAGGTACGCATGTGCCTAG
AAGTGAGAATTGGGTTCAGTTTCCAGCGCCCCGTCCCATTCCCTTGAATCCCATGGATG
GGAATCTGCGTGTGGAACCAAATAGTTCGACGCAATGTTTGGCGGTGAACTGTGGCAG
CGTTTTCTGTAATGGGTGTTGGATTCCCAGTGGGTCGCTTTCAGCACTGCAAGGTTCGT
CAAACTTGAAGCAACTGGGAAGTTATGGTGAAGGTTTGTTGTGTGGCTTTGGTGCTAA
GCAGGATTGGAGAAACACTGGACCTGTGCAGATGAGCAATTTCAACAGGGAGAACAT
TATGCCTTGGGGAGCAGTTTCAGTTCCTTGTGTTGATCATTTGACTGGAGTTGCTTATCC
CCAGAATTCTATGTACAACGGCAACAGGGTTTGTGATGGCTTTCGGAGCAGTATAAGA
AATCATAGGTTTGGAGTAAACGATTCTAGAAATCAGCGTCTCTGTTCATACCATGCTCA

GGATCTTCGAAACATGGCTGAAATGGGTAACCATCGAGCTGCTGCAAGATTGGATGGA
AGGAATTCAATGGGAAAAAGCTACTATGTGCTCAGACCAAATACGAGCATAGCAAAAC
AGGGGCAGTCTATGAGAGTATTTTATCCCCACTCGTGGAGGCAGAGGTCTGTTTTTCCA
GAGCAGCAAGTGTTAAATGGACGGGCTAATATGAAGGATTATAGCATGAATAAGAAAG
AGACTCTGTTGTATGACGTTCAGTATAGCAATGATAAACTACGGGATCAGAGAGGCCCT
GCAGTTTCACCGAGCAGAAATTCAAAGGTTGAGGAATCTTATTTAGCTTATGCCGGAGC
ATGCAGCAGATTGAACAGCCAAATTCTCTCATCTCGTGATGCGATTGAGAGTTCCAATC
CTTGCATGACGACAATGAAGACTCGGGCTGATCTCTTATCTGGTTCTAATTGTGAGAAG
TATGAAATTTCTTCTCAGACTTTGGATGATAATTTCGGTGAAATACCCACAAGTTTTGAT
CCTATCCATTGTTATGAGGTTGCAAACGTCTCTGGTTTTTCTAATGGTCTGATTCATAGC
AATGGAACAGAATTGGGATATAATAAGAAATTTCTAGGTGATGTAGTAGCCGTAGATTC
TCTTAGCAACCATAGAAATGACTCTAAAGATGTACAAGTACACAAGGAAGAAATTGCA
TCTTCGGTTAATAGCTTTCTGGGTAACCCTTCACCATCGTCATCCAAAGAAGTTAAGCC
TCCTGCATTCTCACCTGGTTCTAGCACAGATGCTTCTTCTGATGCCACTCTAAAAGCTC
AATCTAAGGCTGTTGATCTCATGGAGGAAGGACAGCATGGTGGGTCTAAGCTTTCCTCC
CAGAATGCAGGAGAGATGAAAAAAAATCACGTGCAAGAGGGAGAGGTCCAACAAGA
TTCTTTGTGCAGTTTAAGCATTGATGAAAAGACCAACAATAAAGAAAGTCATAAATCCT
CAAAGGTGATCGATGGAATCCCTAGTGACCTGGCAGGATTTTATACTCATCTTGCTACA
CGAGAGCTGCAGACCATTAAAAATTGTGACCTTGAGTATATAAAAGAACTTGGTTCTGG
CACTTATGGAAGTGTATATCATGGAAAATGGAAGGGGTGTGATGTCGCTGTTAAGAGGA
TTAAACCGAGTTGTTTTACTGATGGCACAGTAGAGGAGGATCGATTGGTTGCAGACTTC
TGGAAGGAAGCTCACATGCTTGGTCAGCTTCACCATCCAAATATAGTGGCATTTTATGG
TGTAGTTACAGATGGACCAGTGACAAATCTGGCAACAGTGACAGAGTATATGGTGAAT
GGCTCCTTAAAACAAGTTTTTCGAAGAAAAGATCGAAGTATTGATCGTCGTAAGAGGC
TGATTATAGCAATGGATGCAGCCTTTGGTATGGAATATCTACATGGAAAGGGCATTGTCC
ACTTTGATTTGAAATCTCATAATTTCCTTGTGAATATGAGGGACCCTCAACGGCCAATAT
GCAAGATTGGTGATCTGGGCTTATCTAAAATAAAGCAAAAGACGCTTGTTTCTGGTGG
GGTACGTGGTACAATACCATGGATGGCACCAGAGCTTTTGAACAGTAATAACAACTTAG
TAACTGAAAAGGTTGATGTCTACTCATTTGGGATTGTTATGTGGGAGTTATTGACTGGA
GAGGAACCCTATGTAAACATGCGCTCAGAGGACATAATAGCTGGTATAATTAAAGGCAA
TTTGCGACCTGATATCCCAACTTGGTGTGATCCAGCATGGAGGTCATTGATGGAGAAGT
GCTGGTCACGTGATCCTGATTCTAGGCCATCCTTCACAGAGATTTCAAAGGAGCTTCGC
ACCATATCAGCAACCATGAACATCAAATGA

>LOC107432528（MAPKKK9）

ATGGAGTGGAGCAGAGGACCCATCATCGGCCGAGGCTCCACCGCCACCGTCTCACTCG
CCACCGCCCTCCGGTCCAGTGAACTCTTCTGTGTCAAGTCCGCAGAGCTCTCTTCTTCA
GCTTTCTTGCAAAGAGAACAGAGTTTTCTCTCCAAATTGAGCTGTCCTCGGATTGTTAA
GTACTTGGGTTTTGCTGTTAGTAACGAAAACAATGTGCCTTTGTACAATCTTTGCATGG
AGTACGTGCCCGGTGGCACAATTTCCGATGTAATTCGAAGACATGGAGGGAAGCTGGA
GGAGTCAGTGATCAGGTCCTACACGTATCAGATTCTACAAGGTTTGGATCATCTCCATG
TTAATGGGTTGGCGCATTGTGATATCAAGAGCCAGAACATTCTGATCGGAGAAGACGG
AGCAAAGATTGCCGATTTGGGTTGTGCTAAATTGGTTGGAGTTGATGGGAGTGAAGTC
TTTGCCACGTCAGAGTTTTCCGGCACACCGGTTTTCATGGCGCCTGAGGTCGCACGTG
GAGAGGAGCAGGGATTTGCAGCTGATATATGGGCTATGGGTTGTACGGTCATTGAAATG

GCTACTGGCAGATGCCCTTGGGCTGAGGCAGGTGACCCGGTATCTGCTCTTTATCGGAT
CGGGTATTCCGGCGATGTGCCGGAGCTTCCGAGGTGGTTTTCGGATAAGGGTAAGGAT
TTTCTGAGCAAGTGCTTGATGAGGAATGCAAAAGAGAGGTCGACAGCTAAAGAACTT
CTTAGACATCCATTTGTTGAATCTATGGGGTGTCATTCAGATCAAGTAAAGGAATTCAC
AATGATTAGTCCGAGTAGTGTCTTGGATCAAGAATTGTGGGATTCATTGGAAGAATTGG
AGTCATCTCCTCATGAACCAAACATTGAAGGCTGTTCAATTCTGAGTTCTCCAATGGAA
AGGTTCAGAATGTTGATTGGAGGTACTTTTAGTTTTTCTACACCTCCGAATGTACCCAAT
TGGAGTGTGGATGAAACTTGGTTCACAGTCAGATGCAATATTGACACTGAGATCTTTGA
AGATGAGTCGATTTCGGACTCAAGTTCATCATCCATGGATTCGACCATTCATGAAGAAG
AGCTTGAAAGTTTAATGTTTAATGATGGATTGTCATCTGATTATTCTTTTGAAATAGTTAG
TACTATTGGCAATGAGAGAGATTTTTTGATACCATGTAACAATATGGAAATTACTTTTGT
ATTAGAAAATGTCAAAATTGAAACATACTGCAAAAGATTTTGTTTTATTCAATCTATATG
TTATTCTATATGTGTTAATCCCCACTTTATTTTATTTATTTTTTTTCACCTTCTCATACCGCG
CTTCTTTCTTTATTTATTCCCTCACATGCTTTTCACCAGCTTCAATCTTGCAACAAGCATT
GGTAATTAA

>LOC107411974（MAPKKK10）

ATGCAAGACATGTTGGGATCAGTTCGGCGATCACTTGTGTTCCGTACTTCTTCTCCGGA
CGGCGAAGATTCGCCTTCTCCAATCGGAGTAGGAGGAGGACCAACCTTCACTTTGGTC
GACAAGATCAATTCCTGCATTCGTAAATCCAGAGTCTTCTCCAAACCCTCATCGCCTCT
GGCTCCCCCGTCTCCTTCGCCGCCTCCCATGGCCAAGGACAACACGCCGCCGATCCGA
TGGCGCAAGGGCGAGTTGATCGGTTGCGGCGCCTTTGGCCGTGTCTACATGGGCATGA
ATCTTGACTCCGGAGAGCTTCTGGCCGTTAAACAGGTTTTGATTGCAGCTAATACTGAT
TCAAAGGAGAAAGCTCAGGCTCACATCAAGGAGCTTGAGGAGGAAGTGAAGCTTCTG
AAAAATTTATCTCATCCGAATATTGTTAGATATCTTGGAACAGTGAGGGAGGAGGAAAC
ATTAAACATTCTATTGGAATTTGTACCTGGTGGATCCATATCATCACTTTTGGGAAAATTT
GGATCTTTCCCAGAGGCTGTTGTTAGAACATACACAAAGCAGTTATTGTTGGGTCTAGC
ATATCTACATAAGAATGGAATTATGCACAGGGACATTAAGGGGGCCAATATCCTTGTTGA
TAATAAAGGATGCATTAAACTTGCAGATTTTGGTGCATCAAAACAGGTTGTTGAGCTGG
CAACTATTTCTGGTGCAAAGTCTATGAAGGGTACTCCATACTGGATGGCTCCTGAAGTC
ATTCTCCAGACGGGTCATAGCTTCTCTGCTGATATATGGAGTGTTGGATGTACTGTAATC
GAAATGGCCACTGGAAAGCCTCCCTGGAGTCAACAGTTTCAAGAGGTGGCTGCTTTGT
TTCATATAGGGACAACAAAGTCCCATCCACCAATACCTGAGCATCTCTCTGTTGAGGCG
AAAGATTTTCTTCTGAAATGCCTTCAAAAGGAACCAAATTTGAGGCCAGCTGCATCTG
AGTTGCTTCAGCATCCCTTTGTAACAGGGGAACCCATGGAATCTCATCCTGCTTTTAGT
ACTTCTATGATGGAAAATTCTGAAACCTCTTCACCATCGAACACCAAAGATCTGGAAA
CCTATAAAATGATGACTTGCCTTGAGACAGCTGATGACTGTAATTTGGGTAGTTTGAATT
GCTCTACTGTGTCTCCTGAGAAGTCATTCAAAAGTAAACATCTTTGGAGAATAAACAG
CAGTGATGATGACATGTGTCAGATTGATGACAGCAATGATATTACGATGGGTGAAGGAA
GGCTCAACTCATCCTTGATGCCTGACAACTTTAAGAGCTTTAATCCCATGAGCGAACCC
TCTGATGATTGGGTTTGCAAATTAGCTGCAAGTCCAGAAGCAGAATGTGAAGGAATGA
ATTTGGATCCTGATCAAAAAATTCATTTGCGTGGTGGCTGCGCAGAAACTTTTAATGAT
AGGGAAAAAGATTTTTCTTTTCCTTGTGGACCTTCACTCTCTGAGGATGATGAAGAACT
TACTGAATCTAAAATTAGAGCGTTTTTGGATGAGAAGGCTCTAGAATTGAAGAAACTGC
AAACGCCTTTATATGAAGAGTTCTACAACAGCATGAATGGAGCTTGCTCTCCTAGTTAT

GTGGAAAGCACATGTGATGAAACTACTCCAAGATACTTGAAATTACCTCCCAAAAGTA
GATCACCAAGTCGGGGTCCAATGGGTACCCCATCTGCAGTGGTTGATGCTCATAGTACT
GGAAGCCCTGGGAGTAATGGTAAGCGTGTGTCAAATATTTGCAATGCAAGTGATCAAA
GTTCACAGGACATTCCTGTAATTGATGGTCAGCAGGAGCCTGGTAGTCCAAGAGATGA
TGCGCCAAGCTGGTTTGGGAGGGAAAACATCATCACCTAA

>LOC107412267（MAPKKK11）

ATGAAGCACATTTTCAAGAAGCTTCATATAGGAAGCAACCACGACCCTGGTCGATCCA
ACGAAACTTCTAATACCTCCGTCACGCCGCCACAGACATGCGCCTCCGATCACCGCAC
TGTTTCCGGTCAGAACTCCGGCTATCCTCCCGCAAGCCCATCTTCGTCTTCGCCGTCGC
CGTCTTCGGCTTCCACCGTCAGCGCAACTGGCACCGGAATACCTTTCTCAGCAACTATG
AATCGGTCTGATTACATGTCGTCTGAAGAGGAATTTCAGGTTCAGCTTGCCCTAGCGAT
CAGCGCTTCGAATTCGGATGGTCGCGATGATCCGGAGAAGGATCAAATCCGAGCGGCG
ACTCTCTTGAGCTTGGGGACTCATCGAATGGATTCCGCGAGGGACAAGGACGACGCTG
CCTCCGAGGCCTTGGCCAGGCACTACTGGGAATATAACGTGCTTGACTACGAAGACAA
AGTGGTGGATGGTTTTTATGATGTGCATGGATTGTCCACAGAAGCTATTCAAGGAAAAA
TGCCTTCACTTGCAGATCTTGAGACAAACCTTGGAAACTCTGGCTTTGAAGTTTTATTA
GTCAACCGAACAATTGATCCTGTGTTGGAAGAGTTAATTCAAATTGCACAATGTATTGC
ACTAGACTACCCTGTCAGTGAGGTTACCATTTTGGTACAGAAGCTGGCTGAGCTCGTTA
TGGAACGTATGGGTGGGCCTGTAAAAGATGCTAATTTCATGTTGGCTAGGTGGATGGAA
AGAAGTACTGAGTTGAGGACATCTCTACACACCAGTGTATTTCCTATTGGGTCCATAAC
TCTTGGCCTTTCTCGTCACCGTGCTTTGCTTTTCAAGGTATTGGCTGACAATATCAAGAT
GCCTTGTAGACTTCTAAAAGGTAGTCATTACACAGGTGTCGAGGATGGTGCTTTCAACG
TAATAAAGTTGGAGGATGAAAGGGAGTTTTTGGTTGATCTCATGGCAGCCCCTGGAAC
ACTTATTCCAACTGACATTCCAAGTGCAAAAGACACTACCTTTAAGCCATACAACCCAA
ATAGCAGTATCATTCCAACACATTATTCACTTAATGATTCTGGAGTGGCTTATTCAGGAG
CATACCCTCCACTTCAAGGTGAGGGAAGCAGCCAAAATCCTGCATTTGAAAGCAGCTC
AGTGCTAGGTCGAAATTCAAGGTCTGCAAAGGCAGAATCTGTGCCTGCATTCTCTGGT
TCTAGTGTCGACAACACTGGTTCTGGTGTCGACAACACTGGTTCTTTTAAAATACCTAA
TAAAGTGGCTCCTTCAAATCAGTCAGATCATCTTCCATCATCAGCCATTGGGGCTTCTC
GCTTTAAAGGGAACCGTGGAGCCAATACAGTTGATGGAGGTGTAAGGATGAATATGAA
TGTAGTTCCATATAATCAAAACCCAGAGGATCCTAAGAATCTTTTTGCCGATCTTAATCC
ATTCCAAATAAAAGGAACTGGCAAAACTTCCGTGTATAACAAAACCATAGAGAATAAA
GTTGATGAGATCCAGAGACCAAGGAATAATGTTATCCCTGGCCGACCTCCTGCACCATT
AATGTGGAAAAGTCTGCATGCTTGCAATGAAGTCCCTAGAACAAAAGAGTACGATTATA
TGGAAGGTCTCTTTCCAAGAATCAAACGTGAACCTAATGATTATAAGTTTAATAAGTTG
ACTATGGCGGAGGATGGAAACACTAACTATGAGGGAGAACATCCGAGGGATGCGAAA
GATTTGCGGAGTGACACAGTAGATGTGGCAAAAGAAAATGACGAGATTGATTTCCGTT
ACCATAGAAAATTCACACATGACACAGTTTTGGGTGCCCATTTGAAGTTGAAGGATCC
AGAAAGTCCTAGCTCATCGGTTGAATCCAGCAGAAATAGGGTTGATAAAGTATTTGATG
ATGTAGATGTAGGTGAATGTGAAATTCCGTGGGAAGATCTAGTTCTTGGTGAAAGGATT
GGCCTAGGTTCATATGGTGAAGTTTACCATGCGGATTGGAATGGAACAGAGGTTGCTGT
GAAGAAGTTCTTAGACCAGGATTTCTCAGGCGCTGCTTTGGCTGAGTTCAAAAGGGAA
GTACGGATAATGCGTCGGCTGCGTCATCCAAATGTTGTTCTATTTATGGGTGCTGTTACG
CGACCTCCAAACCTCTCTATTATTACTGAGTTTCTTCCAAGAGGAAGCCTATATCGGATC

ATACATCGTCCTCATCATCAAATTGATGAGAAGCGTAGAATTAAGATGGCTCTTGATGTG
GCTAGGGGTATGAATTGCTTGCATGCTAGTATGCCCACAATTGTTCACAGAGATTTGAA
ATCACCTAACCTTTTGGTTGACAAAAACTGGAATGTTAAGGTAGGTGATTTTGGATTGT
CACGGTTGAAACACAACACTTTTCTGTCATCCAAGTCAACTGCTGGAACGCCTGAGTG
GATGGCACCTGAAGTTCTCCGCAATGAGCCCTCAAATGAAAAGTGTGATGTATATAGCT
TTGGAGTAATTTTGTGGGAGCTTGCTACTATGAGATTGCCTTGGAGCGGGATGAATCCA
ATGCAAGTTGTCGGCGCTGTGGGTTTCCAGAATCGTCGCCTTGAAATACCAAAGGAAG
TGGATCCTTTGGTTGCAAGTATAATTTCTGAGTGTTGGCAGACTGAACCAAACTTGCGG
CCCTCGTTTGCGGAGCTGACGGTGGCTCTCAAGCCCTTGCAACGGCTTGCTGTCCCTT
CCAATGTGGACCAGCCAAGTTCACCTCTACCGCAAGAAATCTCGGTGAATCCTACTCA
AGTGTGA

>LOC107414154（MAPKKK12）

ATGGACTGGACCAGGGTCCGTCCCCTCGGAAATGGCTCATCGGCCACCGTCTCTCTCG
CCGACTCTCGCCGCTTCGGTGGCTTTATCGCCGTCAAGTCGGTGGAGTTTTCCCAATCC
AAAATTTTGCAAAGAGAGCAGAGGATTCTATCTACTTTGAGCAGTCCTTTTGTGGTGAG
CTACATAGGTGAAGATGTTACCTTAGAGAACGACAACAAGCTCATGTACAATCTATTCA
TGGAGTATGTACCTGGTGGTACACTCACCGATACAACTATCCGCCACGGTGGTCGTCTC
GATGAATCGCTGATCGGATACTACACACGGCAAGTCGTTCAGGGGTTGGAGTATTTGCA
TTCATGTGGGTTGGTACATTGTGACATCAAAGGTAGGAACATCCTGGTCGGGGAAGAT
GGTGCGAAGATTGCAGATTTCGGATGCTCCCGGTGGGTTAATCAGCCGGCGGAGATCG
CCGGAACTCCAATGTTTATGGCTCCGGAGGTGGCTCGTGGTGAAGAACAGGGGTTTGC
TTGTGATATATGGGCTTTGGGTTGTACGGTCATTGAAATGGCTACTGGAGGTTCACCATG
GTCTAATGTGGGTTGTGACGCAGTTGGTGCTCTTTATCGGATTGCATTTTCCGGTGAGT
CGCCGGAGTTTCCGAGTTTTCTTTCCGAGCAAGCAAAGGATTTCTTGGGACAGTGCTT
AAAGAGGAATCCGAAACAGAGGTGGACGGCTAGTCAGCTTCTGAAGCATCCGTTTCTT
GAGGAATTCAATTCATGTTCCAAGCAAATCAAAGAATCTAATTCAAGTAGTTGTTCTCC
AACAAGTATTCTCGATCGGGGAATTTGGAACTCGCTGGAGGAATCAGAGACGAGGTGT
TGTGAGAATGAAAATTCTACTGTTGCTGACGATAATAGGATCAGACGGTTGGCAATGTG
TTCAGGGTTGCCTGATTGGACTTCGGATCAGAGTTGGGTCACAATCAGAGTGAATCATA
ATTATGATGATGATGATGAAGATAAGGTTGAAGTGGTTGGTGGGTCAGGAATGGCTTCA
GTTAGTTATAATATAGAAGATTTTGAAATTCCAGCTCCTTCTGTTAGGAAGGAGTTGGTG
AATTTATCGTATAGCAATGTTAGTGATAGAAATGGTAGAGATAGTTTTGAAGACTTCAAG
AACTGTAGGAAAAGCAGTGTAGATTGTAGCAATTTCATTTTTGTTAGGGACAAAGACA
AATTGTTATCTTCTTTAATTTCAAGATGTTAG

>LOC107415263(MAPKKK13)

ATGGGATCTGGAAATGGTGTTTACTCGGTTGGAGAGTTCAGCTTGGATTCCAAGTGGTT
GATCGATCCGAAGCATCTTTTTGTTGGTCCGAGGATTGGGGAAGGCGCGCATGCCAAA
GTGTACGAAGGAAAATATAAGAATCAAACAGTTGCTGTTAAAATTGTCAATAAAGGAG
AAACCCAAGAAGAGATTGCCAAGAAAGAAGCTCGGTTTGCGAGGGAGGTTGCAATGA
TATCCAAAGTTCAACACAAAAATTTAGTGAAGTTTATTGGAGCTTGCAAGGAACCTGT
CATGGTCATTGTAACTGAGCTACTATTGGGTGGAACATTGCGGAAATACCTTTTGAATAT
GCGGCCAAGAAGCTTGGATATGGGCGTGGCAGTTGGGTTTGCACTTGATATTGCTCGC
GCAATGGAATGCTTACACTCCCATGGGATAATTCATCGGGACCTGAAACCTGAAAACTT
GCTCTTGACTGCAGACTATAAAACTGTTAAACTTGCGGATTTTGGCTTAGCTAGAGAA

GAATCATTGACAGAGATGATGACCGCTGAGACTGGGACGTATCGGTGGATGGCTCCTG
AGCTTTACAGCACGGTTACATTAAGGCACGGAGAGAAGAAGCATTATACGAACAAGGT
GGATGCCTACAGCTTTGCAATTGTGTTGTGGGAGCTCATCCATAATAAGTTGCCTTTTG
AAGGCATGTCAAATCTACAGGCTGCATATGCAGCTGCTTTTAAGAATGTGAGGCCCAGT
GCTGAAAACCTACCTGAAGATTTGGCTTTGATTGTAACTTCATGTTGGAAAGAAGACC
CAAACTCTCGACCAAACTTCAGCCAAATCATACAGATGTTGCTGCACTATCTTTCTAC
CATTTCACCTCCAGAGCCTATCATCCCCCTTAGGATGTTCAAATCCGAGAATGCTGTTCT
GCCACCGGAGTCACCCGGTACTAGTTCGCTGATGGCTACTAGAGATGACTCAGGGGAT
ACTCCAAAAAACAACACAGAAGACAAACCTAAAGGATTTTTCTTCTGCTTTAACCAGT
GTTACTGA

>LOC107417160（MAPKKK14）

ATGGAGGAGACACGAGATGATGCAGGGCCAGCAGAGCAGGGGCTTCCTAGTGCTTCA
TGGTGGCCTTCAGATTTTATGGAAAGATTTGGATCTGTTTCTTTGGGTTCTCAAGATGAT
AGCTTAAGCAATACAGAATCACCCAGAAATTCTGAGCAAGATGTGTTGTCATCTCAGA
AAGCATCACAGATTCTGTGGCGTACTGGAATGCTTTCTGAACCAATTCCAAATGGTTTC
TATTCTGTTATTCCGGAGAAAAGCCTCAAGGAGATTTTTGATGATATTCCCTCTCTTGAT
GAACTTCATGCTTTGTGGGGAGAGGGTTTTAAAGCTGATGTCATTCTTGTAGATGGCGC
CAGAGATAAAAAGCTGTCCATGCTGAAGCAATTGATTGCTGCACTAGTGAAAGGATTG
AACTCAAATCCAGCTGCAATGATTAAAAAGATTGCAGGATTGGTTTCTGATTTCTATAA
ACGGCCAAACGTGGAAAGTCCAGCAAAAGCTGCACTAGAAGAAACCTCCAACTTTTT
TGAGAATCGTGGTCTGCAGATGCTTGGGCAAATAAGGCATGGTTCATGCCGTCCTCGA
GCAATATTGTTTAAAGTCTTGGCAGATGCTGTTGGTCTTGAAAGTAGGCTCATGGTGGG
TTTGCCTAGTGAGGGGGCTATTGAATGTGCGGACTCATATAAACATATGTCTGTAATAGT
TGTGTTGAATTCTGTTGAATTCTTGGTTGATCTTATGCGGTTTCCTGGCCAGTTAGTTCG
ACGATCAACCAAGGCAATTTTCATGTGTCATATTTCTGCAGCTGGGGAGAGTGATTCTG
CTGAGAATGATTCATGTGATTCACCATTAGAACCAAATAGTCCTCTATATGGGTTTTCAG
AGAGATTAGATCCTGACAGTACTGAAAAAGATGAGAACCTTCAGTTCCGAAGGTTTGA
TTCAAATGTGCCAGGACCTTCACTACGAAACATGATGTTGCGAACTACTACTGCTGCCG
AGAGGAAATTAAGTTTATCTCATAGTGAACCCAACATTGCAAATACATTTTGGCGGCGT
AGTCGGAGGAAGGTGATTGCTGAACAGCGGACTGCTAGTTCAAGTTACAGGGCTGTAC
GGGCAATGAATGAAACATTGAAGCAAAATCGGCTGTTGAGAGAGCAGGGTGATGATAG
ATCATTTTCCCATCATGCGGATGATGGTAATAGAAGTTCTGTTCCGCAGAAAAATGATCA
AATAAGTTCTCAGAAGGCAATTTCATTACCCTCATCACCACATGATTATAGGAGTCAAAT
TTCTGGGCGAAGTGGACCTTCAGGTTATGTGGCAAATAATGAATTGGTCTCAAGATGGA
ATAAAGTTCTTGAGTCTTCCTTGTTTAATAACAAGCCTCTGTTACCTTACCAAGAATGG
AATATCGATTTCTCAGAGTTGACCGTTGAAACTCGCGTTGGTATAGGGTTCTTTGGAGA
AGTTTTTCGTGGCACATGGAACGGCACAGATGTTGCAATCAAGGTTTTCTTAGAGCAA
GATTTAACTGCTGAAAACATGGAAGACTTCTGCAATGAGATATCCATTCTTAGCCGACT
TCGACATCCTAATGTTATTTTATTTCTGGGTGCATGCACCAAGCCTCCACACTTGTCAAT
GGTTACTGAATACATGGAGATGGGTTCCTTGTATTATTTGATCCATTTAAGCGGTCAGAA
AAAGAAGCTTAGCTGGAGGAGGAGGTTAAAAATGTTGCGTGATATTTGCAGGGGATTG
ATGTGCATACACAGGATGAAGATAGTTCACCGAGACCTAAAAAGTGCAAATTGTCTTG
TGAATAAGCATTGGACAGTCAAGGTCTGTGATTTTGGTCTCTCTAGAATAATGACAGAG
ACACCCATGAGGGACTCCTCATCTGCAGGAACTCCAGAATGGATGGCACCCGAACTTA

TTCGTAACGAACCCTTCACTGAAAAATGTGATATTTTTAGCCTAGGGGTTGTAATGTGG
GAGCTTTGCACTCTGAGTAGACCATGGGAAGGTGTTCCAACAGAGAGGGTAATATATG
CTGTTGCGAATGAGGGATCGAGATTGGAGATTCCTGAAGGTCCACTAGGAAGGTTAAT
TTCAGATTGTTGGGCAGAACAGCATGAACGGCCAAGCTGTGAGGATATACTCGCCCGT
TTGCCAGAATGCGAGTACAATCTTCGCTGA

>LOC107417666（MAPKKK15）

ATGGCGATTTGGCCTGGGCTTTGCCTAAAATGTACTTTGGGGCCCCATAAAAGGTATCC
AAAATATTGGATTTTGAGGGCTCCAGAAATCCTAGATATGTTTGTGAGGCTTCTTCTTGT
GAGATCAACCTTATACTGTCCGGGATTTTACCATAAGCTCGGTGAGGGTTCCAACATGT
CCATAGACAGTTTTGCTAGTTTGCAGACAAGCAATGGTGGAGGATCTGTCGCAATGTCT
ATAGATAACAGCAGTGTTGGTTCAAATACGAATGATTCACATACTCGCATCTTGAACCA
CCAAGGGTTGCGACGTCGTGCAAATGACAACTACTCTGTTGCACAGAGTGTTAACCGA
CGAGGAAGAGTTACACATGCCCTAAGTGATGATGCATTGGCCCAAGCCCTAATGGATAG
CAATTCCCCAACCTTGGGTCTCGAGAATTTTGACGAGTGGACAATTGATTTAAAGAAG
CTTAATATGGGGCCAGCTTTTGCCCAAGGGGCTTTTGGTAAGCTATATAGAGGTACTTAT
AAAGGTGAGGAGGTTGCTATCAAGATCTTGGAGAGGCCAGAAAATGACCCAGAAAAG
GCACAGTTGATGGAGCAGCAATTTCATCAGGAGGTTATGATGCTGGCCACATTAAAGCA
TCCCAACATAGTTAGGTTTATTGGTGCATGTCGGAAACCAATGGTATGGTGCATCGTCA
CAGAATATGCAAAAGGGGGTTCAGTCCGGCAGTTTTTGATGAAGAGACAAAACCGTGC
TGTTCCATTGAAATTGGCAGTAAAACAGGCCTTAGATGTTGCCAGAGGAATGGCATATG
TCCATGGGCTTGGGTTGATTCATAGGGATTTGAAATCTGACAACCTTTTAATCTTTGGGG
ACAAGTCCATTAAGATTGCGGATTTTGGTGTTGCACGCATTGAGGTGCAAACTGAAGG
AATGACACCGGAGACAGGGACTTACCGCTGGATGGCTCCGGAGATGATCCAACACAG
GCCTTACACACAGAAAGTAGATGTGTACAGCTTTGGGATTGTCCTTTGGGAATTGATAA
CAGGAATGCTTCCTTTCCAGAACATGACAGCAGTGCAGGCAGCATTTGCAGTCGTCAA
TAAAGGTGTCCGTCCCATTGTTCCGAATGACTGCTTGCCTGTTCTAAGTGAGATCATGA
CACGGTGCTGGGATGCCAACCCTGATGCGAGGCCACCCTTCACGGAGGTTGTCAGGAT
GCTGGAGAATGCAGAAACTGAGATATTGACAACTGTGCGCAAGGCCCGCTTTCGATGT
TGTATGACACAACCCATGACTACTGATTGA

>LOC107417677(MAPKKK16)

ATGTTGGAGGGTGGTGCCAAGTATACTGGAATTGTTGGCCTGAATAACCGAGATAACA
ACTATTATGATTTGTCCCAAGGATTCTACCATAAGCTTGAGGAGGGTACCAACATGTCCA
TTGACAGTTTTGGAAGCTTGCAAACAAGCAATGATGGAGGGTCTGTTGCGATGTCTGT
AGATAACAGCAGTGTTGGTTCAAATACAAATGATTCACACACCCGCATCTTGAACCATC
AGGGTTTGAGACGGCGGCCAAATGACAACTGCTCTGTGCAACAAAGTGTTAATCATCG
AGGAAAAGTCACACATGCTTTAAGTGATGATGCCCTTGCTCGAGCTCTCATGGACAGC
AATTCCTTGACGCAGGGCCTTGAAAATTATGAGGAGTGGACAATTGATTTAAGGAAGC
TAAATATGGGGGAGGCTTTTGCGCAAGGTGCTTTTGGTAAGCTTTACAGAGGTACTTAC
AATGGTGAGGATGTTGCCATTAAGATCTTGGAGCGGCCAGAAAATGACTTGGAAAAGG
CTCAACTGATGGAACAACAGTTTCAGCAGGAAGTCAAGATGCTTGCTACTCTAAAGCA
TCCAAACATTGTTCGTTTTATTGGGGCCTGTCGTAAACCAATGGTTTGGTGCATTGTGA
CAGAGTATGCCAAGGGTGGTTCAGTTCGGCAGTTCTTAGTGAAGAGACAAAGTCGTGC
AGTTCCACTAAAATTGGCAGTTAAACAAGCATTGGATGTTGCAAGGGGAATGGCATAT
GTCCATGGGCTAGGGTTGATACACCGGGACCTAAAATCTGACAACCTCTTGATTTTTGC

AGACAAATCTATCAAAATTGCTGATTTTGGGGTTGCCCGGATTGAGGTGCAGACAGAA
GGAATGACACCAGAGACTGGGACATACCGCTGGATGGCTCCCGAGATGATTCAGCACA
GGCCTTACACTCAGAAAGTGGATGTTTATAGCTTTGGAATTGTTCTTTGGGAACTGATT
ACGGGGATGGTTCCCTTCCCGAACATGACGGCTGTGCAGGCAGCATTTGCAGTAGTCA
ATAAGGGTGTTCGTCCGATCATTCCAAATGATTGCTTACCTGCTCTTGGTGAGATCATGA
CCCGATGCTGGGATGCCAATCCTGATGTCAGGCCACCCTTCACCCAAGTTGTCAGAATG
CTCGAGATTGCAGAGACTGAGATCATGACTACTGTCCGCAAAGCCCGTTTCCGCTGTT
GCATGGTCCAACCAATGACAGCTGACTGA

>LOC107417907(MAPKKK17)

ATGATGGATCAATCAAGAACAAATAAGCAGCTTCCACGTAATTCCATTGAACATGGATA
TGAGGAGCTACAGCCCCCATCCCAGTCATTTGTGGGTGATCCTCTTAGCAATATGCATG
CTAATACCAGACCCCCTGACCCTAATATTTCAGAAGTTAGACCTGTACTTAATTACTCCA
TACAAACAGGTGAGGAATTTGCTTTTGAATTTATGCGTGATCGGGTGAATCCTAGGAAG
CCTTTGCTTCCCAACACTATGGGTGATCCCAGTTATGCACCAGGCTATTTGGAATTAAA
AGGCATTTTAGGTATCAGCCATGCAGAGTCTGAAAGAGGGTCGGAAGTTTCAATGCAC
AGCATATCACAAAAAGGTCCAGAACACTTTGAGAATAAAAACTCATCTTCTCATGGAG
GCAGAATTAACTATTCGTCAGTTCAATCGGTACCACGAACTTCATCAGGCTATGAGAGT
AGTCGAGGAATTCTTCATGGCTATACCTCTTCTGGAGCCTCTGATAGCTCATCAATGAA
GATGAAAGTTCTTTGCAGCTTTGGGGGTAAAATTTTACCCCGGCCAAGTGATGGAAAG
CTGAGGTATGTTGGAGGTGAAACACGGATTGTCCGAATTAGAAAGGATGTTTCCTGGC
AGGAGCTTACCCAGAGAAGTTTATCGATTTATAACCAAACTCAAGTAATTAAGTATCAG
CTCCCCGGAGAGGATCTCGATGCCTTGGTTTCGGTATCTTCTGATGAGGATCTGCAGAA
TATGATGGAGGAATGCACTGATATAGGCAATGGAGAGGGATCGCAAAAGCTAAGGATC
TTTTTATTTTCTATGAGTGATTTGGATGAAACTCAGTTTAGTCTGGGCAGCATGGATGGT
GATTCTGAGGCTCAGTATGTAGTTGCTGTTAATGGAATGGACCTTGGGTCAAGAAAATC
CTCGACTTTGCATGGTTTTGCAAGCTCATCAGCCAATAATTTAGAAGATCTAGATAGAC
AGAGTATTGAGAAAGAGACAAGTAGAGCTGCAGTAGACTCAGTGGGGGTTAGCAGTG
TACCTTTTCCTGGAAATATTGTTTCATCATCAACCAGTCATTCTCCAGAGCCTGTCCTAC
CTGGTTCCTCAAGTGCATATGAAAATAATCCTCCTTTTTATTATGGCAATATGATGCATTA
TGGAGAAAATATGCCATACCAGCTACCTGATGCCCGCATTACTTCTATTCACTCGCCTTT
AGTTCCTGGTTCAATGCCTGTTCCTGTGGTAAGGGATCAACAAGGAGGCTCAACAGAA
GGGCAAAAATTTGGTGGATCAAGGGTTGAGAACGTGCACATGCCAGTAAAACAGGTG
AAACTGAAATCTGATGGTTCAGTGCAGCAAGACGGTACCAATGAAAATGTTTGCATGT
CAGGGAATGCATATGCTGTCCTTTCACAGCCATACGAGAGTAAATTAATGGATTATTCTC
CTGTTGAAGAGGCATCAGTTGCAGTCACTGCTTCTGAGGGTGGTCTGCATTTGTTGTCA
AAAAATGAGGTAAAGTACCAGGAGCCTGAAAAGGTTTCTGCATCAAATGATTCTGTGC
ACCTGTTGCAGGTTCCTAAATCTAGTGAGGATGATCATTATTCTACATCTAGTACTGCAT
TTGCTACTGGCTATGCTGGATCTGAGTCTAATGCAATGGATTTAAGTTACTTTGAACAAC
CTGTGCTTCCTCAAAGAGTTTATTATTCAGAAAGAATTCCCAGGGAGCAGGCAGAAAT
GCTTAATCAGGGAGAGTTGCTTAATCGATTATCAAAATCGGACGACTCACATGCTTCTG
AATATCTTGTATCTCATTCACGTGCTGATATTTCCCGGCAGGATCCAATAGCAGAAGGAG
TTGACAAAACATATGAAGATGGGAACCTGGCTCCCCCAACTGAACAACCCTCAATGAC
AAAGCCATTATATGTGGATACTCATATTGTTGATGGTGGACTTGCCAAACTTCAGAAGTA
CAAAGAATTTGCTGATTCTGTTACTCAGAATTCGGAGCTCTTGCAGGACAGTGATGTTG

ATTCAAAGCATGCATTTCCAAATCCTATGGATAGTAAAGATGCTGTGAAAGAGGATAGG
TCTGACCAAGAAACAATACGCTCAAAAGATTCCCATGAGAAGCTCCCAGTTGATGAAA
TCCCAGAACATGCACATGTAAATCAGAAAACTTCTGTTGAGCACCAAGAAGATCCTAC
ATCTGATCTTACAAGACATCATTTAAGTGAGGTCATTGCTAAGGATCCCAGTAGTGATG
ACACCATGGGTGATGGACAACCATTTCCAAGGTCTGAGAACTTGGCTAAACGTGCTTC
TCAGGATGCACCCTCAATTGGTATATCCACATCAACGCAAGTTTATATTGAAGATAGGTT
TCCCCGTGATTTCCTTTCTGACATATTCTCCAAAGCTGTACTTTCTGAAGATTCCCCTGG
TGTTGGTTTGCTGCACAATGATGGGGCTGGCTTGAGTTTGAACGTGGAAAATCACGAA
CCTAAACGTTGGTCATATTTTCGGAATTTGGCACAAGAAGGATATAATCAAAATGATGTT
TCTCTAATGGACCAGGATCATCCTGGATATTCAAATGTGCATGGAAGAGTTGAAGAAGA
AGATCATATAGCATATCAGCATGCACCTTTAACAGCAGATAGAGCTCTGATGGACCATGT
AAATTCCCAGACTGCAGCAGAAAGTATTGTTCCATGTTCAGATCATCCCCATGCAATGG
ACACTGAAAGTGTGCAGTTTGGTGCTATAATGGAAAATCGAAGAATGCCAGAATCAGA
CTATGAGGATGGGAAGTTTGAAACCAGGTCTGCTGGTCTACCTCCTCTTGATCCTTCCC
TGGGAGATATAATTGACATCAGTACCTTGCAGGCCATTAGGAATGATGATCTTGAAGAG
CTGAAGGAGCTGGGTTCGGGTACCTTTGGGACTGTGTATCATGGAAAATGGAGGGGAA
CAGATGTTGCAATTAAGAGAATAAAGAAGAGTTGCTTTACTGGTAGATCATCAGAGCA
AGAGAGATTGACTGTAGAGTTCTGGCGGGAAGCTGACATTCTCTCAAAGCTTCACCAT
CCAAATGTGGTAGCATTTTATGGTGTAGTGCAAGATGGACCGGGAGGGACATTAGCTAC
TGTAACTGAATACATGGTTGATGGTTCTCTCAGGCATGTTTTACTTCGCAAAGACAGGT
ATCTTGATCGTCGCAAGCGGCTCATAATTGCTATGGATGCAGCATTTGGAATGGAATATT
TGCACTCAAAGAATATTGTGCATTTTGACTTGAAGTGTGACAACCTGCTTGTGAACTTA
AAAGATCCACTACGTCCAATTTGCAAGGTAGGTGATTTTGGCCTTTCAAAAATCAAGCG
AAATACCCTGGTTTCTGGTGGTGTTCGGGGAACTTTACCATGGATGGCACCGGAGTTGC
TAAATGGCAGCAGCAACAAGGTCTCTGAAAAGGTTGATGTATTCTCTTTTGGCATTGTT
TTATGGGAGATTCTCACCGGTGAGGAGCCATATGCCAACATGCATTACGGTGCAATTAT
AGGAGGTATTGTGAATAATACATTAAGACCAACAATACCAAGTTACTGTGATCCTGAAT
GGAGAAGGCTGATGGAGCAGTGTTGGGCGCCTAATCCTTTGGCTAGGCCATCCTTCA
CAGAGATCGCTAGTCGTTTACGTGTAATGTCTGCAATGGCTAGCCAAACCAAAACACA
GGTTCAGAAGGCATCTAAATGA

>LOC107417903(MAPKKK18)

ATGGAGCAGTCAAGAATAAATAAACAGCCTCAATACAATTCCACTGAACCCGGAAATG
AGGAACTACAGCCGCATTCTCAGTCATTTGTGCGTGATCCTTTTAGCAGTATGCATATGA
ATACAAGACCCCCTGACCCCAATATGTCAGAAGTTAAACCTGTCCTTAATTACTCCATAC
AGACAGGCGAGGAGTTTGCCTTTGAATTCATGCGTGATCGGGTGAATCCTAGGAAGCC
TTTGCTTCCAGATACTGTTGGTGATCCCAATTGTGCACCAGGTTATCTGGAACTTAAGG
GCATGTTAGGTATCAATCATGGGTCTGAAAGTGGGTCTGATATTTCAATGCACAGAATA
GCCGAGAAAGGTCCCAAACAGTTTGAGAGAAAAAATTCATCTTCACATGAAGGCAGA
AATAACCATGCTTCAGTTCCATCAGTTCCACGAAGTTCATCAGGCTTTGAAAGTGGTAG
AGGAGTTGTTCGTGGCTATGCCTCTTCTGGAGCCTCTGATAGCTCATCAATGAAGATAA
AGGTACTCTGCAGCTTTGGTGGTAAAATTCTACCCCGGCCTAGTGATGGAAAGCTCCG
GTATGTTGGAGGTGAAACACGAATTATCTGCATAAGAAAGGATATTTCCTGGCAAGAGC
TTATGCAGAAAGCTTTATCGATGTGCAACCAAACTCATATAATCAAGTATCAGCTCCCTG
GAGAGGATCTTGATGCCTTGGTTTCTGTATCATGTGATGAGGATTTGCAGAATATGATGG

AGGAATGCTCTGATTTTGCAAAGGGAGAAGGGTCAAAAAAGCTTAGGATGTTTTTATT
CTCCATGAGTGACTTAGAAGATGTTCAGTTTGGTCTTGGTAGCATGGATGGTGACTCTG
AGGTTCAGTATGTGGTTGCTGTTAATGGCATGGATCTTGCGTCAAAGAAAAACTCATCT
CTGCATGGTTTGGCAAGCTCTTTGGCAAATAATTTAGATGAGCTAGATAGACAAAGTAC
TGAAAAAGAGACTAACAGTGCTCCAATAGACTCAGCAGGGATTAGCAGTGTTCCTTTG
ACGGGCAATATTGTTTCACCAGTCACGATTCAATCTTCAGAATCTATGATTCCTAGTTCC
TCTAGTGCTTTTGTAATAAAGCCTCCTATTTATCATGGGAAGATGGTTAATTCTGGAGAA
AATATGCAGTACCCATTTCATGATGTCCATGTTCCTCCTATTCCTTCCTCTTTAGTTTCTA
GTTCAATTCCCCTCCATGTGAGTATGGCTCAACATGGAGGTTCAACTGAAGGGCAGCA
GTTCAGTGGATCAAGAGCTGAAAATTCTCAGATGCCAGTAAAGCAAGTGAAACTAAAA
TCTGAGAATCCAGTGCCGCAAGAAAGTACTCCTGAAAAGGTTTTCTCCTCGGGGAAAG
CCTATGGTGTCCCTTTACAACCACATGACGGTAATTTGATGAATTATTTTCCTGTTGAAA
ATGCGACAGTTGCAGTTACTGCTTCGGAGGGGGGTCCTCATTTATTGTCTTCAAAAAAT
GAGGTGAAGTACCAGGAGCCTGACAGGGTTGCTTCATCAAATAACTCTGTGAATCCTT
TGCAGGTTCCCAAGTCTAGTGAGGATGATTTTCATTCCACGACATTTGCCCCTGGTTAT
GGTGGGTCCGAATCCATTGCAATTGACTTGACTTATTTTGAGCAGCCTGTTATTCCTCAG
AGAGTTTACAATTCAGAAAGAATTCCCAGGGAGCAGGCGGAGTTGCTTAATCGATCAA
CAAAATCTGATGATTCACATGGTTCTCAATTTCTCATATCTCATTCTCGTTCTGATGTTTC
CCAGCAGGATCCAATTGCAGAAGGTGTTGACAAATTGCGTGAAGATGGAAACCAGGC
TCTGCAGGTTGAACAATCCACCTCAACAGCAAAGTCATTACATGTGGATACTCATATGG
TTGATGATGGGCTTGCCAGACTTCAAAAGTACGGAGAGTCTGCAGATTCAGTTGCTCG
GATGAAGTCAGAACTATTGCAAGGTGCAGGAAGTGGGTCAAAGCATGAGTTACCAAA
AACTACAGATAATAAAGATGTTACAACCAGTGATAGGACTCTTAAATCTGAGCAAGAA
ACAATTTTCCCTGCAGATGGCCTTAAAAAGCATGTAATAGATGAAAACTCTGAACTTCC
AACTGTAATCCCAAAAGCTTCTGTTGAGAACCCTGAGGAACCTTTGTCTAATCAGCCA
GTTCATCCTTCTAGTGAGGTCACTGGCGAGGATCCTAGTCATGATGACACTTTCGTTGA
TACACAACCTTTTCCTTGGACCAAGAAGTCAGCTAAACATGTTTCTCATGACGCACCCT
CAACTGCTATATCCTCATCTACGCAGGTTGATATTGAAGATCGATTTCGTCGTGATATCCT
GTCTGACATATTTTCCAAGGCAATATTCTCTGATGATTCCCCTGGTGTTGGTTTGCTGCA
CAAAGATGGGGCTGGCTTGAGTTTAAACATTGAAAATCATGATCCCAAACGTTGGTCA
TATTTTCATAAATTGGCACAAGGAGGGTTTCTTCAGAAAGACTTTTCTTTGATTGACCA
GGATCATCCTGCATTTTCATCTGTGCTTGAAAAAGTTGAAGAAGGAGATATCAAATCTT
ATCATCTTACACCTTTTACTACAGATGGAGTTTTGATGGACCATGTTGATTCCCATATGA
AAGTTGGTGAAATTGATCCTAAAGAATTTCCTGCAAAGACTGCAGCTGACATTATAGTT
GTGCAGTCAAATTATGATCAATCTCAAACGAAGGACACTGAAAGTATACAGTTTAGTGC
TATGATGGAAAACCTAAGAATGCCAGAGCTGGAATATGAGGATGGGAAGGTTGAAAGT
AGAAATGTTGGTTTACCTCCACTAGATTCTTCCCTGGGAGATTTTGATCTCGGTGCCTTA
GAGTTACAGGTCATTAAAAATGACGATCTTGAAGAGCTGAAGGAACTGGGTTCAGGTA
CTTTTGGGACTGTGTATCATGGAAAATGGAGGGGAACAGATGTTGCAATTAAGAGAAT
AAAGAAGAGTTGCTTTACTGGGAGATCATCAGAGCAAGAGAGATTGACTGTAGAGTTC
TGGCGGGAAGCTGACATTCTTTCAAAGCTTCACCATCCAAATATAGTAGCCTTTTATGG
TGTAGTGCAAGATGGACCAGGGGGAACGCTAGCAACTGTAACTGAGTTCATGGTTGAT
GGTTCTCTTAGGCATGTTCTACTTCGTAAAGACAGGTATCTTGATCGTCGCAAGCGGTT
AATAATTGCTATGGATGCAGCATTTGGGATGGAATACTTGCACTCAAAGAATATTGTGCA

TTTTGACTTGAAATGTGACAACTTGCTTGTGAACTTGAAAGATCCTTTACGTCCAATTT
GCAAGGTCGGTGATTTTGGCCTCTCAAAGATCAAACGAAATACCCTGGTTTCTGGTGG
TGTTCGTGGAACATTACCATGGATGGCGCCAGAGTTGCTTAACGGAAGCAGCATTAAG
GTCTCTGAAAAGGTTGATGTCTTCTCTTTTGGTATTGTTTATGGGAGATTCTAACCGGT
GAGGAGCCATATGCTAACATGCATTATGGTGCAATTATAGGAGGTATTGTGAATAATACA
TTGAGACCGACAATCCCAAGTTATTGTGATCCTGAATGGAGACGGCTGATGGAGCAGT
GTTGGGCTCCTAATCCTGCAGCTAGACCATCCTTCACAGAAATTGCTAGCCGTTTACGT
GTAATGTCTACATTGGCTAGCCAAACTAAAACCCAGGTTCAGAAGCCATCTAAGTGA

>LOC107418405(MAPKKK19)

ATGAATCTTAACTGGCTTAAACAAATTTCCAACAATGGCAAGTCCGGAAGGAGGCTCT
CACTGGGAGAGTACAAAAGGGCCGTGTCTTGGTCCAAGTATTTGGTATCTTCTGGTGC
AGAGATTAAAGGTGAAGGACAAGAACAATGGAGTGCCGACATGTCCCAATTGTTCATT
GGCTTTAAATTTGCTTCAGGAAGACATAGTAGGATTTATAGAGGGATTTATAAGCAGAG
AGATGTGGCTATTAAGCTGATTAGCCAACCCGAGGAGGATGAAAATTTGGCTTCTTTGC
TTGAGAAGCAATTTATTTCTGAGGTTGCTTTGCTTTTTCGGTTGCAGCATCCAAATATAA
TCACATTTATTGCAGCATGTAAGAAACCTCCTGTGTTCTGTATCATCACTGAGTATTTG
GCAGGGGGCTCATTAAGGAAATACCTTCATCACCAGGAGCCACATTCTGTTCCACTCG
AACTTGTTCTGAAATTAGCCCTTGACATTGCACGGGGGATGCAATATCTTCATGCACAG
GGTATACTTCATAGGGATCTCAAATCAGAAAATTTACTGCTAGGGGAAGATATGTGTGT
CAAAGTTGCAGATTTTGGTATTTCATGCTTAGAATCTCAATGTGGCAGTGCCAAAGGAT
TCACAGGCACATACCGTTGGATGGCACCTGAAATGATTAAAGAGAAACACCACACAAA
GAAAGTTGATGTTTACAGTTTTGGCATAGTTCTTTGGGAGCTTTTAACAGCATTGACAC
CATTTGAGAACATGACTCCTGAACAGGCTGCATTTGCAGTGTCCCAGAAGAATGCAAG
ACCACCTTTGCCATCAGCATGTCCAAGGGCAATCAGTCATCTCATTAATAGATGCTGGT
CAAGCAATCCGGATAAACGACCTCATTTTGATGAAATAGTTTCGATTCTTGAAGGTTAT
GCAGAGTCTCTTGAGCAGGATCCAGGGTTTTTCTCCTACTATAATCCTTCACCAGATCG
TGCCAATTTTCGATGCTTGTCGAAATTTGTTGTTTTCAATAGATCTACCTCTTCAAAAGT
CCAAAAACTTTCTTAA

>LOC107418996（MAPKKK20）

ATGGAGAACCCACCGGCAGATGAGCTTCTTCGGAAGATCCAAGAGCTGGAAGCGGGT
CACGCCCACCTGAAGCAAGAAATGTCGAAGCTGATGCAATCTGATTCGGACGATGTGA
AATCGGAGCATCTTCGTCCTCATCCTTCATACCACCACCAGAGGTCCCACTCAATATCG
CCTCAGCGATCCAGGTTTGGTTCGCCAAGGAGGAGGGTTGGAGCTGGTGGTGGTGGT
GGTGGCGGAGGCTTTGATGGTTGGGGTTCATGCAAGAAAGGTTCGGCGTCGTTTCGGC
ATTCGTCTCCTTTGCAAAGGGAGAGTCGGAGTCGTGAACCTTTAAATGCCGGAGGAGC
AACTGGAACAGGACCTTCGGCTCTCAACTTTACTGATAGGCAGTATCTGAATATCTTGC
AGTCCATGGGGCAGTCTGTTCATATATTTGATCCTGATGGTCGTGTTATTTACTGGAACA
GGTCAGCTGAAAATTTGTACGGTTACTCTGCAGAAGAAGCTCTAGGCCAAGATGCTATT
GAGCTTTTGGTAGATCCCCGTGACTATGACATAGCCAATAATATATTTCAACGTGTCACA
ATGGGGGAGAGCTGGACTGGTCAGTTTCCTGTGAAGAATAAAATGGGGGACAGGTTTT
TGGCTGTTGTAACCAATACTCCTTTCTACGATGATGATGGCACTTTGATTGGGATTATTT
GTGTTTCAAATGATTCACGCCCTTTTCAAGAGACCAAGCTTGAATTTTCTGATTCTAAG
CACTCTGAAAGCAGCTTCAATCGTCCCAGAAACACTGTAACAACTAAACTTGGTCTTG
ATCCTCAGCAGCCACTTCAAGTTGCCATTGCGTCGAAGATATCAAATTTGGCAACCAAG

GTGAGCAACAAAGTTAGGTCTAGGATTCGTGCCGGAGAGACCAACATTGATCGAGAA
GGTGGAAGTGGAGATAGTCATCATTCTGATCATGGTTATTCAGATTCTGTTCTCTCTGAC
CACAGGGAAGATGCAACCTCAAGTGGAGCTAGCACACCTAGAGGTGATATACCCCAAA
GTGCTTTTGGTATATTTTCCCAAGTTGATGAGAAATCTGCAGGAAAACCCTCCAGGGAT
TCTGGAGATGAGAATGAAGGAAAATCTGCAATTCACAAGATTATATCATCAAAGGCAG
AAGCATGGATTGGTAAGAAATCCCTTTCTTGGCCGTGGAAAGGCAATGAGCGAGAAGG
GTCTGAGGTTAAGAGTTCTCGTATTATTTTTCCTTGGTTACAAAATGATCAAGATAATGA
TTCTCTTCATCCGAAGAGTAATGTTATGAAGCCAGAAAACAGTGCGAATGAGAGCCAT
CGGCCTGCGAATAATGAGGCTTCTGGATCCTGGTCATCGTCTTTCAATGTTAACAGCAC
AAGCAGTGTCAGCAGTTGTGGAAGTACAAGCAGTAGTGCTGTGAATAGGGTAGACATG
GATACTGACTGCCTAGATTATGAAATCTTATGGGAAGACTTGACAATTGGAGAGCAGAT
TGGTCAAGGTTCTTGTGGAACTGTGTATCACGGCCTGTGGTATGGATCAGATGTTGCTG
TCAAGGTATTCTCAAAGCAAGAATATTCAGATGAAGTAATACTTTCCTTCAGACAAGAG
GTATCTCTTATGAAAAGACTGCGGCATCCAAATGTTCTGCTCTTTATGGGAGCTGTTACT
TCACCTCAGCGTCTTTGCATTGTAACAGAGTTCCTACCACGGGGAAGTTTGTTTCGCTT
ACTACAAAGGAACTCATCAAAACTAGATTGGAGAAAGCGTGTTCATATGGCTTTGGATA
TAGCACGGGGCATGAATTATCTTCATCATTTTAACCCACCTATCATTCATCGAGATTTGA
AGTCTTCGAATCTCCTTGTTGATAGGAACTGGACTGTCAAGGTTGGTGATTTTGGTCTT
TCACGTCTTAAGCATGAAACATATCTCACAACTAAGACTGGGAAGGGAACGCCTCAGT
GGATGGCGCCAGAAGTTCTTCGCAATGAACCCTCAGATGAGAAGTCTGACATATACAG
CTTTGGTGTTATATTGTGGGAGCTTGCTACTGAGAAGATTCCTTGGGATAATCTCAACTC
AATGCAGGTGATTGGAGCAGTAGGGTTCATGAATCAAAGGCTGGAGATCCCAAAAGAT
GTAGACCCACAATGGGCTTCAATAATTGAGAGCTGCTGGCATAGTGATCCAGCTTCTCG
GCCAACATTTCAAGAACTGCTGGATAAGCTCAGGGAGCTTCAAAGACACTATACTCTC
CAGTTCCAAGCAGCACGTTCTGCCACCGGAGATAACACTCAAAAGGAGTTGTAG

>LOC107420999(MAPKKK21)

ATGCATCACATACCACGAATTTTCAACCGTAGCAAGCGAGAAAAATCCATGGATACGA
AGAAGAACCCTAGGAAGCCAAGGCTTGAGCGCCGCAATGCTGCGAAGCACATTGATT
ACGAAGCTTCCACGTCTTCTTCGTCGATGGAGTCGACGCCGTCGCCGTCGCTCCACAG
TCGATCGCTGGATTTGTCGGACAAGATGAGCTTCCGTGTCGAGGGAATTGAGGGCGAG
TTCGACATAATATGCCGGACCTTGGGGCTTTCGGGTATAGAAGATTTCGCTATCCCGTCG
GCGGCATGGGAGGCTCGGAAGATCCGATCGATCTCCGATATTCTACCTAGGTCCAGGCT
TAATCGGTTAGATAGTTTGAGTGGTTCAGCCAGGGAGGAACTGAAGGATGAGGTTATT
CAAGCTGTGGCCGAATTGAACGATAGGGTCGTCGGTAGCGTTAGAATTAGGGGTGGTG
ATAATGAGTTGCCTCAGGCTGATCTTGCCGAGTCCCGTGGTTGTTGTGTTGCTAACGAT
GGAGCGTGCGATGGTGATTTCGTCGGTGGTGGTGGTGGTGGTGGGGGAGGAGGCGGA
GGAGGAGGAATTAAGGGTGTTCGGCCGCCGGTGCTTAAGCCTCCTCCTTCGATGAGGC
TTCCGGTGATTGATAAGACGTGCTCGACTTGGGACATTCTGAGGGACTTTGCGCCCGAT
GAAGAAACATCACCTGCGCAACTTCTCCATAAAACGTATAATTCTTCCGATGAAGAAG
AAGAAAAAGAAGAAGAAAAGCACCACCCAAGAGAAGAAAACCTCCAAGAAGAAGA
ATCACATCAAGAAGAAGAACAAGTCCACACGGAAGAAGTTGTTAGGCAAAGAGAGG
AGTCTGCGGTGAGGCTTCCGGAGAATGCGTTACTCTCCGAGTCGTGTTCGTTCACTAC
GTCCAATGACGACGACTCTTCTAGCAGTACCACAGAGCCCACGTCGAATATTTCGCCG
AATGCGAGATTAAGGCCCATCATCACGTATTGGGAAAAGGGCGATCTTCTGGGTCGCG

GATCGTTTGGATCTGTTTATGAAGGAATAACTGATGATGGGTACTTTTTTGCTGTGAAG
GAGGTTTCCTTGCTTGATCAGGGAAGTCAAGGAAAGCAAAGTGTTTATCAACTTGAGC
AGGAGATTGCACTGTTAAGCCAGTTTGAACATGAAAACATTGTTCAATATTTGGGCAC
TGATAAGGATGAATCAAAACTTTATATTTTTCTAGAACTTGTAACTAAAGGTTCTCTGCA
AAGTCTTTATCAGAAGTATAACCTTCGAGATTCACAAGTTTCTGCCTACACAAGACAGA
TACTGCATGGTCTGAAGTATCTCCATGACCGAAATGTGGTTCACAGGGATATTAAATGT
GCAAATATATTGGTCTCTGCCAGTGGATCTGTTAAGCTTGCAGATTTTGGGTTGGCTAA
GGCAACCAAATTGAATGATGTTCACTCTTCCAAGGGGACTGCATGCTGGATGGCACCT
GAGGTTGTTAATAGGAGGAACCAAGGATATGGGCTGCCGGCTGATATATGGAGCCTGG
GATGCACTGTGTTGGAGATGTTAACCAGACAGATCCCGTACTCTGATTTGGAGTGGATG
CAGGCAATATTTCGAATAGGAAAGGGTGTGCTACCTTCTGTTCCTGATTCTCTCTCGAA
AGATGCACGGGATTTTATCTTGCAGTGCTTGAAAGTTAATCCAAATGATCGTCCTACTG
CTTGTCAGCTGTTAGACCACCCATTTGTGAAGAGACGTCTTTCCTCATCTTCTGGCTCT
GCATCTCCTTACCATTTTGGGGGGCGGAGTTAA

>LOC107421353（MAPKKK22）

ATGCCTCACAGAACAACTTACTTCTTTCCGAGGCAATTCCCGGATCGACGATTCGATGA
ATCTTCCAAACAGCTGTTGGCGGATCACGAGAAAAAGGTATCAACAGCGACCGCTAGC
ACACCCACTTCCACCACCACCACCACCACCCCCCCTACCCCTACATGCAAGGACACTA
TCAGTAACTTTCAAATCGAAAGCGACAGAAAGCCATCAAAACAGTTCTCTGTCAACAG
CAAATCCTCAGCTGTATCGCAGCTCTTCACGGGTCATGGAAAATCACACACCAAACAG
CACACACAGCCACAGCAAGTCAAACCGCAACAGCAACAACATTTCGCAGCCTTCTGC
GATTGGTTGGCCGAGAAGAAAGCCGAACGTTCCACCACCGCGGCGCACGTGAAACCA
CCGCGCGATGAGGACCGTGAGCTTCTTATCCCACCACCTCCTGAGTCGGTTCCCGAAG
TGGCACCGCCTGAGTCGGTGGTTAAGGATCGGAGTGTGGATAGGAATTTCGACCGGCA
GGTTTCGTTGCCGAGAGTTTCCAGTGGGAGTAGCTATGCGGGAAGTTTGTTCTCTGGG
ACGACTCTAGATGGGAATCTGTCGATCGATGTTAAGGACAGTTATTCGAAAGTGTCGTC
GTTTTCCACGGCGAGACATGAGGAAGTGGAGGTGGAGGAGGAAGAGGAGGTGGACA
AGAATAGGGGGAGCTTGGCGCAGAAGACTAAGGAGAGTTATTATCTACGGCTTGCGTT
GGCTAAAAGGCTCACTTCTCAAGCTTTTCTTGATCATACCGAGCCTCTTCTTATGCAAG
TTTCTGGACCTGAGTCCTCTAACGCCGAAATCGTTTCTTATCGACTCTGGGTGAGTGGC
TGCTTGTCTTACACTGACAAGATATCAGATGGTTTTTATAACATTTTGGGGATGAATCCA
TATGTGTGGGTGATGTGCAATGATCCGGAGGAAGGAAAACGCCTACCACCATTGATGT
CACTTAAAGCAATCGAACCCAGTGAGACATCTATGGAAGTGGTCCTTATTGATAAGCGA
GGAGACTCTCGTCTGAAGGAGCTTCAAGATAAGGCGCAAGAGCTGTATTGCGCTGCAG
AGAATACGTTAGTGTTGGTGGAAAAACTAGGGAAACTTGTTGCCATCTATATGGGGGG
CACTTTCCCTGTTGAACAAGGGGATCTTCACATGCGCTGGAAACTGGTAAGCAAAAGG
TTGAGGCATTTTCAGAAATGTATTGTGGTCCCCATCGGCAGTCTTTCTATGGGGCTTTGC
AGGCATCGCGCTATTCTTTTCAAGAAGTTGGCAGACTACATAGGCTTGCCTTGTCGGAT
AGCTCGCGGTTGCAAGTATTGTGCAGCTGATCATCGGTCTTCTTGTCTTGTCAAAATTG
AAGATGACAGGCAATCCACAAGGGAATATGTAGTTGATTTAGTTGGAGAACCAGGAAA
TGTGCATGGTCCAGATTCCTCGATCAATGGAGGATTTCTTTCTTCAATGCCTTCACCATT
ACAAAGTTCTCATCTCAAAGAATTCCAAGAACCTTACATGGATGGTGCATCTTGTTGTC
AAAGTGTAAACTCAAAACACACATGTCTTCCAACTGAAAGTCCCCTTTTTGCAGGCTT
TGGGAAAGAAACCCAGAAGGCTGAGGAAAATACTTTTTCCAATAATCTAAAGGGTGTT

ACAAATGGTCCAGTTTATCAAGCCACATCTGGGAAGGAATCTTCTCCAATGCCTTTGGA
GATGAAGGGGAATTTTGAGAATTGTGTCATCCAGACTCCGGTGATGCCTTCTTTCCATG
GAAATCAAGCTGTAAAGAGGAGCCCAAGGAAAAAAAAGAGTGCCAAGCAACCGAA
ATTAAGAGTGAACTTATCCAGTGAATCAGATGCTGAGGAGGTTGAGAGTGAACCTGAC
AATGGAGGCAACTTTTCTTCTGTGACAATCCCCAGATACTTGAATCTTGAACCATCTCT
TGCAATGGATTGGCTTGAGATCTCATGGGATGAGTTGCATATCAAGGAGCGTATTGGTG
CTGGTTCATTTGGGACTGTGCATCGTGCTGAATGGCATGGATCGGATGTTGCTGTTAAG
GTTCTTACGGTCCAGGATTTCCATGATGATCAGTTAAAGGAATTTTTGAGAGAGGTTGC
AATAATGAAACGTGTACGCCATCCAAATGTAGTCCTTTTCATGGGTGCAGTTACAAAGC
GTCCACATCTGTCAATAGTGACTGAATATCTGCCTAGGGGTAGTCTATATCGCCTTATAC
ACAGGCCTGCTTCCGGTGAAATTTTGGATCAGAGGCGGCGATTACGCATGGCACTGGA
TGTGGCTAAGGGGATCAACTATCTCCATCGTTTAAACCCTCCTATAGTTCACTGGGATCT
TAAATCTCCCAATTTGTTGGTTGATAAAAATTGGACAGTCAAGGTATGTGATTTTGGGC
TGTCCAGATTTAAAGCAAACACCTTTATATCTTCCAAGTCTGTTGCTGGCACGCCTGAG
TGGATGGCGCCAGAATTTCTTCGTGGAGAGCCCTCAAATGAGAAGTCTGATGTTTACA
GTTTTGGAGTGATCCTTTGGGAACTTGTAACCTTGCAACAACCTTGGAGTGGACTAAG
CCCTGCACAGGTTGTTGGAGCTGTTGCATTCCAAAATCGGAGGCTCGCAATCCCACCA
AATGCGTCTCCAGTGTTGGTTTCCCTGATGGAATCTTGCTGGGCTGACGATCCCGCTCA
GCGGCCAGCTTTTAGTAGTATAGTTGAATCGTTAAAGAAGTTGCTGAAGTCTCCAGTGC
AACTGATACAGATGGAGGGGACGTAA

>LOC107420099(MAPKKK23)

ATGTCGAAAATGAAGCATCTACTGAGGAAGCTTCACATCGGCGGGGGTTTCAATGACC
CCCAAAGATTGGGCGACGCCCGACCCGTAACTAGCCCGAATTCGAATCATCCCGATCC
GTCCACCTCTTCTACGGCCTCGTCGTTGTCGTCGGCCTCGACTTCCAGCTCCACTACAA
TGGCGGGAATTGCGACTGTTGAATCTGTTGCCGATCGTTTGGGTGGGGATAGTAGTGGT
GGTGGCGTGGATTTCAATTTTTTGGAGGAGGAGTTTCAGGTGCAGTTGGCCATGGCGA
TTAGCGCTTCGGATCCCGATGCGCGCGACGACCCCGAGTCCGCTCAGATCGACGCCGC
TAAGCGGATTAGCCTGGGTTGCTCTGCTGCTGTCACCGACAGCCCAGCCCTTGTTGATT
TTCTTTCGCTTCGATACTGGAACTATAATGTTGTAAATTATGATGAAAAGGTGGTGGATG
GTTTTTATGACGTATATGGTGTTACATCAGATTCACTTGCACAAGGGAAGATGCCATTAT
TGGTTGATCTTCAAGCAATATCTGTTTTAGATAATATTGATTACGAGGTGTTGTTGGTCA
ACCGCATGGTTGATCCTGAGCTTCAGCGGTTGGAGAAAAGGGCATATGCAATATCAAA
AGAATCTCATGTTTCTAGACATGGTTTAGTTTTAAGTGGCTTGATCCAGAACATTGCTAA
TATTGTTGTTGATAGAATGGGTGGCCCAGTTGGTGATGCCAATGAAATGTTGAGAAGGT
GGGTTGTGCGGAGGTATGAATTGAGGAGTTCAATGAACACTATCATACTTCCCCTTGGA
TGCCTTGATGTTGGACTTTCACGCCACAGAGCCTTGCTCTTTAAGGTATTAGCTGATAC
GATTAATCTTCCATGTATGCTGGTCAAAGGGAGTTATTACACTGGTACTGATGATGGAGC
TGTGAACTTAATTAAACTTGAAAACGGAAGTGAATATATTATTGATCTGATGGGGGCTC
CTGGCACACTAATTCCGGCTGAGGTACCCAGTAGTCAGCTCCAAAATTCTTTCCTTGGT
ATGAGGAGCTTTTCAGATGTGAAAGTGATGCCCACTGGTCCAAGATTTCTTCGTGCTGG
AGGAATTGGACCACTAGCGGTTTCCAAAGTAGGAAGTTCAAGGTCAGATGAAGTATCA
TATGGAGACTCTGGGTCAAAAGATAATGAGAGGACCCTTCTTGGAGAAAATCCAATTG
TGAGATGTGAGAATGAGTTTGGTAAGCCTCTACCATCTCCACATAGATCATCTCAAACT
TCACTTGGCACTTGTGGAAAAACATCGTTGGCACAGAAAAAGAAAGTAAAAAATGTAT

CAAAATATGTCATCAGTGCAGCAAAGGACCCGGAATTTGCACAGAAACTGCATGCTGT
CTTGTTAGAAAGTGGTGCTTCACCTCCTCCAGATTTATTTCTGGATATAAGTTCACAGGA
TCTTGATGAAGGCAAAGTGCTTGGTCGAATCCAAGCAGTAGATCAGGAAATTGCATCT
GACGGAGTGCATATAAACACAGATAACTTAAGGTCAAGTCTTGAGAGATCTCTCATACC
TTTTTCTGGAGGGGAGAATTCTAACTATGTTAGTGTGGATAACATGAAAAAACGAGCTC
CTATGGTTTTAGCTGACAAGCAAAATGAATTGGAGATAATTTCCAATAAGTCTAAATTTG
CTCTAACTTCTGATACTCTTAGCGAGGGATTTATGCTTCTTGTTAATGAACCTAGTGAAA
TGACCCAGACTGATGCTGTAAATATAGATATGGCTTCTTCTGATCCATCAAATATGTACA
CACGATCTTCACAGGAAGAACAGTTTAACAAACCTGCCATGCCTTCTCAAGTCAACTC
TGGTCAAAGACATCTTGAAAATGCCTATGTCAATGATGATAACATGATTGTGATGGAAA
GAGTTGATAATGGCTTTGTTATTGGTTCTTCTGGTCAAAGTGAGGGAATTAACCCAGTG
CTGGGGGAAGCTGCAGAGTGGGAAATTCCATGGGAGGATCTTCGGATTGGTGAGCGCA
TTGGTATTGGTTCATATGGTGAGGTTTATCATGCGGAATGGAATGGCACTGAAGTTGCT
GTGAAGAAGTTTCTAGACCAAGGATTTTCTGGTGAAGCATTGCTTCAGTTTAAATGTGA
AGTTGAAATCATGTTAAGACTGAGACATCCAAATGTTGTTCTTTTCATGGGGGCAGTTA
CTCGGCCCCCACATTTCTCTATACTGACAGAGTTTCTTCCCAGGGGGAGTTTATATAGAT
TACTGCATCGCCCTAATTCTCAACTTGATGAAAAAAGGCGAATGAAAATGGCACATGA
TGTGGCCAAGGGAATGAATTACCTTCATACTAGCCATCCTACTATTGTGCATCGAGATCT
GAAGTCTCCAAATCTCCTTGTTGATAGAAACTGGGTTGTTAAGGTTTGTGATTTTGGTT
TGTCACGCATGAAGCACCACACATTTCTGTCTTCAAAGTCTACTGCTGGAACGCCTGA
ATGGATGGCACCTGAGGTCTTAAGAAATGAACCAGCCAATGAGAAGTGTGATGTGTAC
AGTTTTGGTGTGATATTATGGGAATTGGCTACCTGCCGTATCCCATGGAAAGGCTTGAA
CCCCATGCAGGTTGTTGGAGCTGTTGGGTTCCAGAATAGGCGTCTTGAAATTCCAGAA
GAGGTTGATCCAGTGGTAGCCCAGATAATGTATGATTGTTGGCAAACGGAGCCAAATTT
ACGACCCTCTTTCTCGGAACTCATGCTCCGCCTCCGACATCTTCAACGTCTAGTTGTTG
AAAGATCCAATTCCACAAATGAACGATAA

>LOC107422567(MAPKKK24)

ATGACCAAGGAGGCTCGAGCTGGTACTTCCAGCCAACAGTTCTACAAAGATACACCAA
ATGTTGTGTCAAACAACAGAGCTGCAGCAGATAGAGATGTAGATAACTTTTGTATACAG
ACCGGTGAGGAATTCTCGACAGAGTTTCTGCAGGATCGTGCTGGGCTAAGGAGATTAG
CACCGGTCATGACTAATGTCAATCAGCGCCTGCCAACAAGATCTGGTTTAAATTATAAT
CAAAACCATCAGCAGCTGGCATATGAGGATCTCAGTGGCATTCTTGGGTTAAGAAGAG
TGGACTCTGAATGCAGCTCGGAAGTATCAGATTTTGTTCATGGTTCAGGATATTTTCCTG
CTGAAGTAGATTACAAGGTGCATCCTAATAATATAAACAGATATCATTGGGAATATGGTG
CTGCTATTGGACAAGTACCAAGTAAGCACGTTGATAACCGTGATCGTGTTCCGCCGGTA
CTACCAACTGCTCCACCGTTTTATGTACTCGATTCGCCTCAGGCATACCATCCTTATGTG
CAAGGGTTTTCTGACGGTTCTGTGTCTAGCAAGATGAGATTTCTCTGCAGCTTTGGGGG
AAGAATATTACCAAGACCAAGTGATGGGAAGCTTAGATATGTTGGCGGAGAAACACGA
ATCATATCCATCAGGAAGAACATTACCTGGGAGGAACTAGTGAAGAAGACTAGCACAA
TTTTTAGCCAACCACACACTATCAAGTACCAACTTCCAGGTGAGGATCTGGATGCTCTT
ATATCCATTTGTTCCGATGAGGACCTTCATCATATGTTAGAGGAATATCTAGAGCAGGAA
AGAACCGAGGGTTCTCAAAGATTAAGGCTTTTTCTTATACCTTTAAATGAATCTGAAAG
CCCAAGTTCTGTTGAAGCAAGGGCTACCCAGACAAATGATGTTGAAAATCAGTATGTT
GTTGCTGTTAATGGGATGTTGGAACCAAGTCCCAAGAAGAGTTCAAGTGGGCACAGTT

TGACAAGCCAGACTAGCCAATTGGGCAATACCTTAGAACATAGTCCAAGATTTCATAGG
GACTCACCTACATCTACATATATTTTGGAGAATAAGGATCATAGTCCAAGACCTCCTGCT
CAGTATACAACTGTACTTCAGGGTAGGGATCCCAAGAATTCTGATGTGATAGATCGACC
ATACGCTGGCGGTGATGAAGGGAGTTCTTCCTTTCTTATGGGAAAACTTCCATGTCAAG
ATTCCTGTAACGTTGATGCTTTGAGTCGTTGTCATGGGCTTCCACTCACAGATAACCATA
ATCAGACCAAGTATTTGGTAGAGGCTGATTGGGCGAATAGGGCGTCCGACATCTGCTTT
CACCATCCTGGGCCAGGAGGAAACTTTGTGTCTTCCGCGCAAAATGTTCAAACTGGTG
TACGCTTTGAGAGAGTGATGCCTAGGGAGAGCTCATTCCATTCTAACAATTCTGGTTCT
CACCAAGATCACCTACCAACTTTGTTGTCCGGAACTGAGATCAAAGATGTTCCTCACA
ACAAAATGATGCATGCCCTTTCTGACTCTCTGCTGCAAGAACATTATGACAGACCCTCA
GATGGATTTATGCCCCTGTCGTCATCAAAAATTAAGAGAGACAAGTTGCTTCCAATAAC
AAGGTCTAGTTCATCAAGAGAATGTGTAATGCAAGGTGTTGAGAAAGCTGAATCCCAA
GTAGCTGTGCATGAAAATCAATTCACAGTCAAGAAACCAAGCCATTCCAGGGGAAAG
GGGAGTGAGGAGTTGTTGAAGTGGACACACAGGAAAAACAGTTCTGGTGATCAGAAA
AATTGGAATCACCATGAAGGAAATGTTGATGTTAAATCAAAGAATAATTCTCTTGAAAG
TAGCAACTTACCAAATATCAACTACATGCATAAAAATTGTGTATCCTCACAAGAGTTGC
AAATCCCTGAAGGAATGGTTTCTGCTTCCCCAGTGACACCATTAGAAAATTTGTTGGAT
ACTAGGAGCCTAAATTCTATGCATGATCAGCAAAGTTCTACCACTCAGAGGATAAGTTG
TCAAAGATCCCTCAGACGAAAAACACCTGCTGCATCTGATGAATTTGTTGGCTTTGAAT
CTCTAGCTACAAGCTCTAAAGTGGCTTCAACTGTCAGTTCAGATAGTGAAGCATCTCTT
CATGATAAGGAAGCTATGAATCGTGCTTACAATGAGATTGAAATATTAAGTTTTAGCGG
AGAAGCTATTGAAGTGCCTAAGTTTGAATATGCCATCTCTGTCCAATCACAGCCTTTAG
ATGATTATCATGACAATGAGGCAATGGAATCACCGATTATTGTTGAAGACATAACTGGC
ATAGCAACTCCTGGCATTCCTTCCTCCTCTCGAGCGGTTGCATATGTAGAATACACATGT
AGTGATGATGAATGTACATCTCCTAGAGAAACAAAAGCAGACAGTAGCATTCAGGAAT
CTTCTGGAGAGGTTGAGAATGATAGTGCTGACGGTAGGGATGAATCTATAAGTATGAGT
GATGCAGCTATAGCGGAGATTGAAGCAGGAGTTTACGGTTTGCAGATCATAAAGAATGA
TGATCTTGAAGAACTGCAAGAGCTAGGATCTGGTACATTTGGAACTGTTTACCATGGGA
AGTGGAGAGGAACAGATGTTGCTATTAAGAGAATTAAAAAGAGTTGCTTTTCAGGCAG
ATCATCGGAGCAAGAGCGGCTGATCAAAGACTTTTGGCGAGAAGCACGTATACTGTCA
AATCTTCACCATCCAAATGTCGTAGCATTTTATGGGGTTGTTCCTGATGGACCAGAGAC
AACATTGGCAACTGTAACTGAGTTTATGGTCAATGGCTCATTGAGGCATGTCCTACTAA
AGAAAGATATAGTGCTTGATCGTCGAAAGAGGCTCCTAATTGCGATGGACACAGCTTTT
GGCATGGAATATTTGCATTTGAAAAATATAGTTCATTTTGATTTAAAATGTGACAATTTG
CTAGTGAACTTAAGGGATTCTGAGCGACCCATATGCAAGGTTGGAGATTTTGGGTTGTC
GAGAATTAAACGCAACACGCTAGTATCTGGTGGTGTGAGAGGAACCCTTCCATGGATG
GCACCAGAGTTATTAAGTGGTAGCAGTAACCGGGTGTCTGAGAAGGTTGATGTTTTCTC
ATTTGGTATTGTGATGTGGGAGATCCTGACTGGGGAAGAACCATATGCTAACATGCACT
GTGGTGCTATTATAGGGGGAATTGTAAATAACACACTAAGGCCTCCTATTCCAAAACGT
TGTGATTCTGAATGGAAAAAGTTAATGGAAGAGTGCTGGTCAGCTGACCCTGCATCCC
GGCCATCATATACAGAAATTACAAACAGGCTACGGGATATGTCAATGGCGCTGCCAAAA
AAGCGACAGAATGTTACAAGTAGAAGAGTCTAA

>LOC107423026(MAPKKK25)

ATGCAAGACATCTTCGGATCGGTCCGGCGGTCCTTAGTGTTCCGAACTCAAGGTGTGG

ATGATAATGGCGGATTCGGTGGTCTGGTCGAGAAAATCGGCACCAGCATTCGCAAATC
GCGAATCGGATTGTTCTCCAAACAGTCTCTTCGGGCTCTCCCTCCGGCTCCGAAGAAG
GACGATACGCCGCCGATCCGGTGGCGGAAGGGCGAGTTGATTGGTTCCGGTGCGTTTG
GTAGGGTTTACATGGGGATGAACCTGGACTCCGGTGAGCTTCTTGCTATCAAACAGGTT
CTGATCGCGGCAAATAGTGCTTCCAAGGAGAAAACACAGGCTCAGATTTGGGAGTTGG
AGGAAGAAGTAAAGCTTCTGAAGAATCTTTCACATCCAAATATAGTTAGGTACTTGGGA
ACTGCTAGAGAGGATGAGTCGTTGAACATTCTGTTGGAATTTGTTCCGGGTGGATCGAT
ATCATCTCTACTGGGGAAATTTGGATCCTTTCCTGAGTCTGTCATAAGAATGTACACGA
AACAGCTATTATTGGGTCTTGAGTACCTTCACAAGAATGGAATTATGCATAGGGACATC
AAGGGTGCAAACATCCTTGTTGATAATAAGGGATGCATTAAACTTGCAGACTTTGGTGC
CTCAAAGAAAGTTGTTGAGCTGGCTACTATAAATGGTGCCAAGTCAATGAAGGGTACT
CCATATTGGATGTCTCCTGAAGTTATTCTTCAAACTGGGCATAGCTTCTCTGCTGATATAT
GGAGTGTTGGATGTACTGTGATCGAGATGGCTACTGGAAAGCCCCCATGGAGCCAGCA
GTATCAGGAGGTTGCAGCTATCTTCCATATTGGAACAACAAAATCTCATCCACCAATCC
CTGAGCATCTTTCTGCTGAGGCTAAAGATTTTCTGTTGAAATGCTTACAAGAGGAACCA
AACTTAAGGCCCTCTGCATCAGACTTGTTGCAGCATCCATTTGTAACTTTGGAGTATCA
GGAACCTCATAGAGTATTCCGCACTTCATTTATGGAATCTGGAAACCAGATGGCAACAC
CTGGGGGAAATCTTAGGAACTCCTTCAACCCTGCAATTAGAAGGTCAACCTGCGCAGG
CTTGAAAGATATTTTTGATATGGGTAGTGTGAGGTGCTCAACTGTATATCCTGAGAATTT
GTCAGAAGTGAGCTCTCACTGGGGACCAATGAATTGTGACGATGAGATGTGTCAGATT
GATGATCAAGATGATCTTATGGTTAGTTCATTAGTCAAGTTCAAATCTGCCTCAGTCTCT
AATGATTTAACAAAGAGTTTTAATCCTATGTCTGAACCAACTGATGATTGGCCATGTAA
GTTTGACGAGAGTCTAGAGTTGGCTAGAAGTGGAACAGACTTATCTCCTGGTCACACA
ATTCAAAAGGCTGCCGGCAGTCCTGGGGCATCTGATAAGGAGGAGACTGGTTTTGAAT
TTGCCCACGGGCCATTGGCTGCTGAGGATGACGATGAAGTTACAGAGTCAAAAATTAG
AGCTTTTCTTGATGAAAAGGCATTAGATCTGAAGAAGCTGCAAACCCCTCTATATGAGG
AGTTCTATAACACATTGAATGCAACTGATCCTCAAAGAGTGAATGGAATTATGAATGCT
GTAAATGCTTCAAATAATTGGAATTTGCCTCCCAAGAGCAGGTCTCCCAATCAGGCACC
TCGTAGAAGACTGTCTGCAATGGTTGATGCTGCAAAAACTGCAAGCCATGGGACTGTG
AGCCATTCCAAAATTGTATCAAATGCTAGCATTGTGCATAGTCGAGCTTTACAGGAAAT
CCAGCCCCCTCAAGCTAGTGAATGGAAAGGGCTTCCTCTTGATGCTCAGGAAGGATCA
TTTAGTCCAAGTGCAAGCTTCTCTGAGAGGCAAAGGAGGTGGAAAGAAGAACTTGTT
CAAGAGCTTGAAAGGAAGCGAGAGATGATGCGACATGCAGGAGGAAAGACATCATCC
CCAAAGGATCCGGTTCTAATTCGACAGAGAGAGCGGCTACGAGTTGCATTCCCTGGAA
AATGA

>LOC107423093（MAPKKK26）

ATGGATTCTAGGACAAGTGATGGTGTTGGAAGTGACAAAACACCGATGAAGATGGACA
ATCAAAAAGGCATTATAAACTCAAAGGCTGAAGATACGGGAAGTTTCAATAGCAAAGA
TATGGTTTTCAGAGCCGATAAAATTGATCTGAAGAACTTAGATATACAGCTTGAGAAGC
ATCTGAGCAGGGTTTTGACAAGAAACACCGATCCCCAAAAGCCTGAAGAAGAGTGGG
CCATTGATTTATCAAAATTGGATATTAGATATGTTGTAGCTCAGGGGACCTATGGTATTG
TGTACAGAGGCACTTATGATAATCAAGATGTTGCAGTCAAAGTGTTGGACTGGGGGA
GGACGGTATTGCTACAGCTGCTGAAACTGCTGCTTTGCGGGCATCATTTCGGCAAGAG
GTTGCTGTTTGGTACAAGCTTGACCATCCTAATGTTACAAAGTTTATTGGAGCTTCAATG

GGAACTTCAGATCTAAAGATTCCTTCAAAAAATCCTACCGATGGTAACAATTCCCATCC
TTCTAGGGCATGTTGTGTTGTTGTAGAGTATCTTCCCGGTGGAACACTCAAAAAGTTTC
TAATTAGAAATAGAAGAAAGAAACTTGCACTAAAGGTTGTGGTCCAACTTGCTTTGGA
CCTCGCTAGAGGTCTTAGTTATTTGCATTCAAAGAAAATTGTACATCGTGATGTCAAAA
CAGAAAATATGTTACTAGATGCTCATAGGACATTGAAAATAGCTGATTTTGGTGTTGCTC
GTGTTGAAGCTCAGAATCCAAGGGACATGACTGGTGAAACCGGTACCCTTGGATACAT
GGCTCCGGAGGTTCTTGATGGAAAGCCATACAATAGAAGGTGCGACGTGTACAGCTTT
GGCATATGCTTGTGGGAAATTTATTGTTGTGATATGCCTTACCCAGACCTTAGCTTTGCT
GATATATCATCTGCTGTTGTTAGACAGAATTTACGACCGGAAATTCCAAGATGCTGTCCA
AGTGCATTGGCAAATATAATGAAAAAATGCTGGGATGGAAATGCAGACAAACGTCCTG
AAATGGATGAAGTGGTGAGAATGTTGGAAGCAATTGATACGAGTAAAGGAGGAGGGA
TGATACCTGAGGACCAAACTCCAATGTGTTTCTGTTTGGGCCGTGCTCGCGGCCCTTGA

>LOC107423594(MAPKKK27)

ATGGAAAACTTCAAGTATCCATCTTCACCACCTTCCAAGAGAATGAGGGGAGCAGAAA
CAATAGATTCAGAAGGTCCGTACAGGCTGCTATACTGTTCAAGTAAGGGAGACACAGA
AGGGGTAGTGCAGGAATTAGAGAAAGGTGTGGAGCCTAATCTGGCTGATTATGATAAA
AGAACTGCTCTCCATCTGGCATCGTGTGAAGGTTGTACGGAGGTTGTTGTTCTACTTCT
AGAGAAAGGTGCAGATGTCAATTCCATTGATCGTTGGGGGCGAACTCCACTGTCAGAT
GCTCGTAGCTTTGGTCATGAAAGCATCTGCAAGATATTGGAGGCTAATGGTGGAATTGA
TCCGGTGGGGCTTGACTCCCAGATTCCATGCTATACTATTGATTATTCTGAGGTGAACAT
GAATGACGCAATACTTATTGGAGAGGGGTCATATGGTGAAATATATTTGGTGAAATGGA
GAGGTACAGAAGTTGCTGCAAAGACGATTCGCTCCTCTATTGCATCAAATCAAAGGGT
CAAGAATTCCTTTATGAAAGAACTTGCTTTGTGGCAAAAGTTGCGCCACCCCAATATAG
TTCAGTTCCTTGGTGTTCTAAACAATTGTAGCCGATTGATCTTCCTTACCGAGTATCTTT
GCAATGGAAGTTTGCATGACATATTAAGAAAGAAAGGAAGACTTGATCCTCAAACTGC
AGTTGCTTATGCTCTTGATATTGCCAGAGGTATGAATTATCTCCATCAGCATAAATCACAT
GCTATAATTCACAGAGATTTGACCCCAAGGAATGTGTTACAAGATGCAGCTGGCCGACT
CAAGGTTACTGATTTTGGATTGAGCAAAATTGCACAGGAAAAAGATGTTTTAGGTTATA
AAATGACTGGAACAACTGGTTCCTTTCGATACATGGCACCGGAGGTATATCGCCGTGAA
TCTTATGGGAAAGGTGTTGATGTCTTCTCCTTTGCTCTAATCGTACACGAGATGTTTCAA
GGAGGACCATCAAATCTGGCAGATGATCCAGAACAAGTAGCAGATAAACGCGCATATG
AAGATTCCAGACCGCCTCTCTCCTCTTATTTATACCCTGAACCAATTAGGATACTTCTTC
AAAAATGCTGGCACAAGAATCCGGATTTCAGACCTACATTTGAAGATATAATTTTAGAG
CTTGAGTCTATTCAAGACAAGTTTCAGACTACCGAACCAATGCCTTCAAGTTGCAGGT
GTTCCATCCTATGA

>LOC107424157(MAPKKK28)

ATGGAGCAATTTCGACATATTGGAGAGGTGTTAGGAAGTTTAAAAGCTATAATGGTATT
TCGAGGTAATATCCAAATCAATCAGAAACAATGCTTTTTCCTGTTCGATATCTTCTGCTT
TGCATATGAATCAATAGCAGAAGAGATGGAACATAACCTCAGATTCGAAGAGAAGCAC
ACGAAATGGAAAATTCTTGAGCAGCCATTGAGAGACCTCTACAGGATATTTAGAGAAG
GAGAAACCTATATTAGACAATGCTTGGAAACAAAGGACTGGTGGGCAAAAGCCATAAC
TCTTTATCAGAACTCGGATTGCGTTGAGTTCCATATTCACAACTTGCTTTGCTGCATGCC
AAATGTCATTGAGGCAATTGAAACTGCTGGAGAAATCTCCGGATTGGATCAGGATGTG
ATGCAAAAGAAGAAACATGTCTACTCCAACAAGTACAGGAGAGAGTACAGAGATTGG

AAACTTTTCCAGTGGAGATTTGGGAAGCAGTACCTGATCACTCAGGATTTCTGCAACC
GGATTGACACTGTTTGGAAAGAAGATAGATGGACTTTGCTTAACAAAATCAGGGAAAA
GAAAATCTCAGGCTCAACGAAATACGAGCAGCGTCTCATAGACCTCTTTTTCAAAAGC
TTAGATGGCTTGGAGCCATTGAATGGGAGGCTCTTCCCCTGTTCAATTCTTGTGGGATC
CAAGGACTACCATGTGAGACGAAGATTAGGGAGTGGCAGCCAATACAAAGAGATCCTA
TGGTTAGGTGAAAGCTTTGTATTGAGACACTTCTTTGGTGACATCGAAACCCTTCAGCC
GCAGCAGGAGATCACTTCGTTGCTATCTCTTTCCCATCCAAACATAATGCACTATCTTTG
TGGTTTTACTGATGAGGAGAAGAAAGAGTGTTTTCTGATCACTGAACTAATGAACAAA
GATCTTAGCAGCTACAACAAAGAGATTTGCGGGCCAAGGAAAAAAATCCCGTTTTCGC
TTCCTGTTGCGGTTGATCTGATGCTTCAAATTGCAAGAGGGATGGAATATCTTCACTCA
AAGCAAATCTTCCATGGTCATTTAAATCCTTCCAACATACTTGTAAGACCAAGAGGTAT
TTCCTCAGAAGGAAACTTGCATGCAAAAGTTTCAGGTTTTGGCTTAAATTCTATTAGGA
GATTTACCACTAAAAGCCCTTCTAATCATAATGGAACACTTCCATTCATCTGGTATTCTC
CTGAAGTTTTGGAGGAACAAGAACAAACTGGAGCTGCTGAGAACTCAAAGTATACAG
AGAAGTCAGATGTTTACAGCTTTGGGATGGTTTGCTTTGAGCTTCTGACCGGAAAAGT
CCCTTTTGAGGATAGTCATCTCCAAGGTGACAAAATGAGCAGAAACATCAGGGCAGGA
GAGAGGCCGCTGTTCACTTTCCAGTCACCAAAATATGTGACCAATTTGACAAAGAAAT
GCTGGCATACTGACCCAAATCAAAGGCCAAGCTTCACATCTATCTGTAGGATTCTTCGT
TACATAAAGAGGTTTCTAGCAATGAATCCGGATCATAATATTCAGCTTGATCCACCCGTC
CCCCATGTAGATTACTGTGACATCGAGTCAAGGCTTCTCAGGAATTTCCCATCTTGCCA
ATCATCGGTATCGCAGATCCCATTTCAGATGTTTGTTTATAGAATTTTAGAAAAAGAAAA
AATAAGTGCAAGCCTAAAAGATAATTCAGAATCAGGAAGTGATGGAGCTTCAGTTTGC
GGGGATGATCATATAACTACAGTGGATGATCCATTCCCATCATCACCAATACCAGAAAG
GAAGTCTTTAGCATCACTTGATGTTACGAACAGAAAACTTTCCATTAAGAGATCTCCAG
ATTCAAGACTGAACAAACTACCAGGGACACCGAGAGGACGATCAGTAAGGCCTCCAC
AAATGTCGCCTTGTGGGCGTAGTATGCGAATGAGTTCAGAAAGCCAGCTAATGGCAAT
GAGTCCAAGAATACGAAGAACATCATCTGGTCATGCATCAGATTCAGAGATCTCATGA
>LOC107424505(MAPKKK29)
ATGGAGTGGGTTCGAGGAGAAGAAATCGGTAGTGGAAGCTTCGCTACAATCAGTTTAG
CAGTACCCAGAAAAGCCTCAGCTGAAATTCCTCCATTGATGGCCGTGAAATCTTCGGA
AATCTTCAACTCTGCTTCGATCAATAACGAGAAAGAAATACTGGATCAGCTCGCAGATT
GCCCGCAAGTGATTCGGTGTTTCGGAGAAAACTACAGCACTGAGAAAGGCGAAGAAT
TTCATAACTTGATGTTGGAGTATGCTTCCGGAGGCAGCTTGGTTGATCAGCTCAAGAAA
AATGGCGGTCGGTTGTCGGAATCGGATATCCGACGATATACGAGGACGATACTCAAAGG
TCTTTCGTTTATCCATACGAAAGGTTTTGTTCACTGCGATATAAAGCTTCAGAATATCCT
AGTGTTCGAAAACGGCGCGGCTAAGATTGCAGACTTTGGATTGGCGAAGAAAACAGG
GGAGAAGAAAACAGAGGTAGAGCTGAGAGGCACTCCTCTGTATATGTCTCCTGAATCT
GTAAACGACAATGAGTACGAATCGCCTTCGGATATTTGGGCACTTGGGTGCGCAGTGG
TTGAGATGATGACCGGAAAACCGGCTTGGAATTGCGAACCGGGTTCGAATATCTGTGC
GCTTTTGATAAGAATTGGAGTTGGAGAAGAATTGCCCCAAGTTCCAGAGGAGTTGTCT
GAGGAAGGAAAAGATTTTCTGGGAAGATGTTTTGTGAAAGATCCGAGAAAACGATGG
ACGGCTGATAGGCTTTTGAAACATCCGTTTATCTCCGACGACGACACTGTTTCATTGGA
GGAGTTATCTACGTCGCCGAGAAGCCCTTTTGATTTCCCAGAATGGGTTTCGATGCAAT
CGACGGTGCGATTTTCAAAACTGTCTGAATTTTCACCGGAATCGGAAAATTGGTTCGG

AAGGGAATTGGATTCTTACTTTGGTTCGTCGTATTCTTCCTCTTCTTCTTCTACTTGTTTA
GAAAATCGGCTCCGGCGGCTTATGTCCGATGAGACACCCTATTGGTTAGTTTCAGACAG
TTGGGTCACAGTAAGGTGA

>LOC107424832(MAPKKK30)

ATGCAAAAACGAGCTCCAGCAATGGCGGAACATGTGACGCAAACAAACAAAGAGCTC
TTAAGGCTCTCGCTTTCCTACTCTCTGGACACTATCGGCAACTCCAACCGTCGACTCAC
TGCAAACGACGAAACCAATGATTCTGGCAACGAATTCGTCTTCAACATTGACCCCAGC
TTGCTTATTGACCCTCACAGCGTCAAGATAGGCCGTTTGATTGGTGAAGGCCCTCACTC
CATCGTCTATGAAGGATTGTACAAATCCAAGCCCGTTGCTGTTAAGATCATACAACCAA
TGAGATCATCAGCTGTAAGTGTGGAACGTAAAAAAAGATTTCAGAGGGAGGTTACACT
GCAATCAAAGATGCAACATGAAAACATTGTCAAGCTTATCGGTGCCTCAGTGGAGCCG
ACTATGGTAATAGTTACCGAACTCATGAAAGGCGGTACACTGCAAAAGTACTTGTGGA
GCATCCGGCCAAGGCCTCTAGATCCAAAGCTTTCAATAAATTTTGCACTGGATGTCTCT
AGAGTTATGGAATTTTTGCATGCAAATGGCATCATCCACCGTGACCTGAAGCCAAGCA
ACATACTTCTTACAGAAGACATGAAACAGATTAAGCTTGCTGACTTCGGGTTAGCTAGA
GAGGAGATCTCGGGTGAAATGACTAGTGAAGCAGGAACTTATCGGTGGATGGCTCCCG
AGTTATTTAGCCTGGACCCCCTTCCTAGTGGAGGAAAGTTACAATATGATCACAAAGTG
GATGTGTACAGCTTCTCAATTGTTCTTTGGGAGCTGCTCACAAACAAAACTCCATTCAA
GGGAAGGAATAGCGTGATGGTTGCATATGCTACTGCCAAAAAAATAAGACCATGTCTG
GAGAACATTCCCAAGGATTTAGTCCCTCTCCTGCAGTCCTGTTGGGGAGAAGACCCGA
AAAGCCGACCAGAGTTTCTGGAAATCACAGTCTCCCTCTCCAACTACCAGAATTTACT
CTCAATAGATATTAAAGCTACCGAGATTGTCGAGACTGAACATCCCAAAAGCAACACA
ATTACGAAGGAAGACCCTGCAGGAACCAATATCAATCCTCAGTCCAAAAATGCTGTTG
TAAAGGTTAAGAGACGTAGGAAATCTTCACCCAGTTTCCTCCGTTGCTTTGATTATTGC
TCTGCTCAATGA

>LOC107425633(MAPKKK31)

ATGGGTAAAAAATTGCCTGCTTGGTGGCCGAGAAAATCGAGTAAGAACAAAGAAGAA
CATCGACAGAATCAGCTTCAGCACCAAAACCCACATGGAAACCACAACAGCTTCCTAA
AATCTCCCATCAGGACCGATAATAAGAAAGCCAAAGACAAGCCCAAGAGCTTTGACGA
AGTCTTCCCCCGCGGTTCGCCGAGGGCCAGCAAGGACTTTGGTGCTCCGGGTGGATCA
TCTTCTGCGTTTTCGGGTTTCGATTCGGATGGTCCAGAGAAGCGTGGCCATCCTCTGCC
TCGGCCATCTGTATCGTCCACGCAAAGCATTGGGATTGATCAGGCGGTTGGGTTGGGAT
CCGGGTCGTTCTCGGGTTCCAGCGTTAGCTCTTCTGGGTCTTCGGAGGACCACCCGGT
TGCTCATGATCATGGTCAATTTAGTGCTTTCAGGTTGGGTTTGAAGGGATCACCTGCTTT
GGGATTTGGAGGAAATGGCGATCCGAAATTCAACGCAAGGTCAAGAAGCCCAAATCC
AGGGTCTAGGGGACCCACTAGCCCCACATCCCCTCTCCATCCTCTGTTTAGTGGCATGG
GTCTCGAGTCTCCAACGGGGAAGCAAGAAGATGGAAAGAGCCAATGTCATCGGTTGC
CTCTTCCGCCAGGTTCCCCTACTAGCCCTTCTGCCTTGTCTAACACTAGAAGTGGTGCT
GTTACTGAAAGCGCATCCTGCACAGTCTCAAAATGGAAAAAGGGAAAGCTTCTAGGG
AGGGGAAGTTTTGGCCATGTTTATGTTGGATTTAACAGTGAAAGTGGACAAATGTGTG
CGATAAAAGAAGTCAGGCTTGTTTCTGATGATCAGACATCGAAAGAAAGTCTTAAGC
AACTGCACCAGGAGATATATTTGCTTAGTCAGCTCTCACATCCGAACATTGTTCGCTACT
ATGGAAGTGAATTGGCTGAAGACACCCTCTCAGTTTATTTGGAATATGTGTCTGGTGGT
TCGATCCACAAATTACTTCAAGAATATGGCTCCTTTAAAGAACCTGTTATTCAAAATTAT

ACCAGGCAGATTACTTCTGGGCTTGCATACTTACATGGAAGAAACACAGTGCACAGGG
ACATCAAAGGGGCAAACATATTAGTAGATCCCAATGGCGAAGTCAAGTTGGCAGATTT
TGGCATGGCTAAACATATAACAAATTGTTCGTCGATGCTCTCTTTTAAGGGAAGTCCATA
CTGGATGGCTCCTGAGGTTGTAATGAATACCAATGGATACAGTCTTGCGGTGGATATATG
GAGCTTGGGGTGTACAATTCTTGAAATGGCTACGTCCAAACCTCCATGGAGTCAGTATG
AAGGGGTGGCTGCAATATTTAAAATTGGGAACAGCAAAGATATTCCAGAAATCCCTGAT
TATCTTTCCTATGATGCAAAGGATTTTGTGAGGCTATGCTTGCAACGAGAGCCATTGTC
ACGTCCTACAGCATCCCAACTGTTAGACCACCCTTTCATTCGGGACCAAACAACAACA
AGAGTTGCTAACATCAATCTTACCAAAGATGCCTTTCCCTATGCTTTTGATGGAAGCAG
GACACCGGTAAGTCCATGA

>LOC107426395(MAPKKK32)

ATGGATTCAAGCAAAGGTGAGGGTGTTGGAGACCTTGAGAAGGATGAGAAATCAAAT
AACAGAGAAGGAGATGGTTCCTCTGGTAAAGCCAAGGCGTTGGGTCTACAGGGAAAG
GATGATTTGGGAAGCATAAGTGAGAAAAATATGCTATTCAGAGCGGATATGATTGATTTT
AAGAGCTGGGATGTGCAGTTGGAGAAGCACTTGAGCAGGGTTTGGTCTAGGGAGAGA
GAAGCGTGTACAAGGAAGGAGGAGTGGGAATTCGATTTGTGTAAACTGGATATAAGAT
ATCTGGTTGCTCAGGGAGCTTATGGTACTGTATATCGTGGTTCCTACAATGGTCAAGATG
TTGCTGTGAAGATTTTGGATTGGGGAGAAGATGGTATGGCCACGGTTGCTGAAACAGC
TGCTCTTCGGGCAGCATTTCGGCAAGAGGTTGCTGTTTGGCATAAGCTTGATCATCCAA
ATGTTACGAAGTTTCTTGGCGCTTCGATGGGAACTTCAAATCTTAAAATCCCTTCCAAC
AGCACATCAAGTGACGACCAAAATTCTCTTCCTTCCAGGGCTTGTTGCGTTATTGTTGA
ATACCTTCCAGGCGGGACACTAAAAAACTTTTTAATCAGGAATAGGAGAAGGAAACTT
GCCTATAAGATTGTGATTCAACTTGCTTTGGATCTTTCTAGAGGTTTGAGCTACCTTCAC
TCAAAAAAGATTGTACACCGGGATGTCAAAACTGAAAACATGTTGCTAGATGTTGGTA
GGAATCTGAAAATTGCTGATTTCGGTGTTGCTAGAGTCGAAGCCCAGAACCCAAGGGA
TATGACAGGGGAGACTGGAACTCCTGGTTACATGGCCCCAGAGGTTCTTGATGGTAAG
CCTTACAATAGAAAATGTGACGTGTACAGTTTTGGAATATGCTTATGGGAAATCTATTGT
TGTGATATGCCTTACCCTAATCTAAGTTTTGCTGATATATCCTCTGCAGTTGTGTGGCAG
AATTTACGACCGGAGATCCCAAGGTGTTGTCCAAGTTCTCTGGCAAACATCATGCGAA
AATGTTGGGATGCAAACCCAGAGAAACGCCTTGAAATGGATGAGGTAGTGAGGCTGTT
AGAAGCAATAGATACAAGCAAGGGAGGTGGAATGATTCCTGATGACCAGACTCCTGG
TTGTTTCTGTTTTGCCCCAGCTCGTGGCCCCTAA

>LOC107426719(MAPKKK33)

ATGCAGCTCAATAGGTCTGAGAACTTGTTTGATACCATGACCAATGAGGTTCCTGGTCC
ACCAGGACGTTGGATCAGGCAAGAATCTACCTCAGTTGTTGCAAATGTGGGGAAGAAT
GTACATGGTAAAAACGTCTTAATACACACAGGTGAGGAATTTTCCAGGGAGTATGTTCA
GGATAGTGTTTCTGGGCGAAGAATTCCTTCTGGAATTAATACGGCTCAGAACCATGAAA
AGAAAGTTCCTTCTGTTGCTAAAGCTAGGAGCCACGAAAAGAGAGTTTCTGCTGTAAC
TGATATGACTCAGAACCATGAAAAGAAAGTTGGATATGATTGTAATCAGAATCATCAGC
TGGGATATGAGGATCTTGCTCGTATTCTGGGGTTAAGGAGGATGAATTCAGAGTGTGCT
TCGGAAACATCTGAATTTCTCTCTGCAAAAGGATCTTCCAGAGAGATTGAAGTTGACG
GTTATGTAGATAAATTAAGCAGATGCAACAATGAAGAAGGAGATAATGGGCATGGATCA
AGGAAGCCTTGTGGTGAACTAAATTGTGATACTTCTGGTTTTGGACCAACTGGTCCACT
CAATTATAAATCTGATTCCCATAATTGCAACAACTTTAGTGGGTCAGGAGTTTTAGATGG

TTCTCAATCTGAGAAGTTGAAAGTTCTCTGCAGTTTTGGTGGAAAAATTTTGCCTAGGC
CCAGTGATGGGAAACTCAGATATGTTGGGGGAGAGACACGTATCATCTCAATCCGGAA
AAATATCTCATGGGATGAGCTTGTGAAGAGAACGTCAAGTATTTGCAACCAACCTCATT
CTATTAAGTACCAGCTTCCGGACGAGGATCTTGATGCTCTTATATCTGTGTCTTCTGATG
AGGATCTTCAAAATATGATAGAGGAGTACCATGGGCTTGAAAGGCTTGATGGTTCTCAA
AGACTACGGATCTTTTTGATTCCTTTAGGTGAATCTGAAAGCACAACTTCCTTTGAGGC
AGGCACCTTACAGCAAAGCAATCCAAATTACCAATATGTTGCAGCTGTTAATGGTATGG
CAGATCCTAGTCCTAGGAAAAATGCTGGAGAGCTGAATTTAGCAAGTGAAGCAAGTCA
ACCGGAAACTAAGACATCTGCATTTCCTGTGGAGATTAAGAGTAACTTTAATGCCTTGA
ATCCTAACCCATTTTTAGAATCCCAGAATATAAACAGATCTTATAATCAGTCTCCTCCTGT
TACTCCAATTCTGCATCAGCATGGAGATTCCAAGATTATTCACATGCTACCACATGGTGA
TAACTCATGCCAGGGTAGTTATGAGAGCAACTCTTCTTTTGTTTCTACTCAGCTACATCC
TGAGAACTCCAGTTACAAATACCCTCCAGCTGGGGCATCAAGTTCAATAAATTACCATT
ATCATGACCCTTATAAGCTTGCTGACGTTGGATATCAAGATCAGCCACATGGTGGAAAT
TTCAATAACCTCAATCTGAGCAAAGAGTTGGCAACTCCTTTGGTTGTCGATCAAAATGA
TGGTGATTTTGGTGGACTCTCCCTTGAGAGGCCAGTGCAAAAGGAAAGGACATTTCAT
TCTGAAAAGCCTAGTTTGCGTCTGGAAGATCCTATGGGTCTGTTGTCGGAATGTAATGA
CTCTGTTGATTCTTTCCGGGGCATACCACATGCATTTTCTGACTCCAAATTGCAAGAAA
GTGGAGGGAAGTCTGTAACTGCACACTGTTCACAAGAAGGAATGAGCCCATCATCCCC
CTTGAACTTTGCAAAGGCTCAATTGTCTTTGCTGCTGAACTCAGGTGTCTCACAAGAA
AAGCCAACCAAACTGCATGAGAATATCAATATCGTCAATCCCTGGGAGCAAAATAAGTT
AGTAGATGATGGGTCACTTGAAGGGCACAAAATACTGGATCTCCCAAATTCTTCTCCAT
GTTGCGAAGCAGTGTGCAGGAAGGAACCTACTCAAAAGGGTACTGGTAATACTGATGA
CAAGTTTCAAACTTCTGAAAATAATCTCTTTAAATCTGCGTTTATGATGCCTAATCCATG
TGAGAAAGATTCTTTGACTTTGGAAACAATGAAGAGAACCAATGCAGTAGACCCTCTA
TTGGACCAAGAAGGAAAGCTTTATGGAGGGAGTTTGCTTACCGCAGGTGTGGAATACA
AGAACAAATTGCCTAATAAAAACCCAAATCCATCTTTTACAAATGTCTCTGAAGATACA
ATACCTACATCCTCAGCCATGGATTTCAAACCTATGGTAGATAACTTGGTCGAGCATCCT
ATTAACTGTCGACATGAAAAAACTGTCCCTGATCTATTGGGCACAAGCCAAATAGTTTC
CAGTGATCAGAATTGTGCTCTGACTGGGAGATTGATTGATGAACCAGGAAACAGTGAC
TCAGGTACCGCAGATCCAGAAATTCCAGGCTTAGGTCCGACTGTGAGAAGGCTCTCAC
ACAATGGAAATTCTTTGTCTGATCTAATGCCTGGGTCGTCCAATGATTCATTTGTCCCTG
AACCTGCACTGGTTCAGCCTGTTGCTAGTAATATGGTTCATGAAGAACCCTTGCTATTA
AGCTCTGTAAATTTGCATCCATTGCAACCTCGTGGTGATCCTGGTATAAACTCAAATTTG
CAAGACCAGAAACCGTTGCAAGATGCTGGGTTTAAGCGAGAGGTTTCTCTCCTTGATG
ATGATTTTGTTGGCTATCCAAATTCAAAGGTTGAGAATATTGGCTTTGGAGGATCCAGTT
ATGAAGAATCTAATGTTGCAGATGGTAAAAATAAAAATCAGTTGGAGTCAGTGGTTACT
GTGGAGGATGTGACTGATGTTGTTCTTCCTGGCATTCAGTCTTCATCCACATGTACTCCA
TATAACATGGATGAAACCATTGGTTATGTTATATCCCCTACTGCAACAGAAGTGGAAAG
CGTCATTCCCGAGTCTGAGTCGGAGGATGGTAAAGCATATGATGAGGACAATGGACCA
TTTACTGATGCAATGATAGCTGAGATGGAAGCCAGCATCTATGGCTTGCAGATTATAAG
GAATGCTGACCTTGAAGAACTTCAGGAGTTAGGATCTGGTACATATGGAACTGTTTATC
ATGGAAAATGGCGAGGAACAGATGTTGCCATAAAGAGAATTAAAAAGAGCTGCTTTGC
AGGGAGATCATCTGAGCAAGAACGGTTGACGAAAGACTTCTGGAGAGAAGCACAGAT

CCTCTCAAATCTTCATCACCCAAATGTGGTTGCATTTTATGGGGTAGTACCTGATGGAAC
TGGAGGAACGTTGGCAACAGTAACTGAATATATGGTGAACGGATCACTTAGGCATGTC
CTACTTAAGAAGGATAGATCGCTTGACCGTCGAAAAAAGCTTATTATTGCCATGGATGC
GGCCTTTGGCATGGAATACTTGCATTCAAAGAATATTGTCCATTTTGATTTGAAATGTGA
TAACTTATTAGTCAATTTGAGGGATCCACAAAGGCCTATATGCAAGGTTGGAGATTTCG
GATTATCAAGAATTAAACGAAACACTCTAGTATCAGGTGGCGTGAGAGGAACACTTCC
ATGGATGGCACCAGAACTGTTGAATGGTAGTAGCAACCGGGTTTCTGAGAAGGTTGAT
GTTTTCTCATTTGGGATTTCAATGTGGGAAATCTTGACTGGGGAGGAACCCTATGCGAA
TATGCATTGTGGTGCCATCATTGGGGGAATTGTGAAAAACACTCTTCGACCTCCTGTCC
CGGAACGCTGTGATCCTGAGTGGAGGAAGCTGATGGAACAATGCTGGTCACCTGATCC
TGAAATCCGACCATCATTTACTGAAATAACTAATAGGCTGCGAACTATGTCTATTGCGCT
TCAGGCAAAAGGACCTAGTAATCAGACAAGACAGATGAAGCCTAACATGTCCTCGTAA

>LOC107427400(MAPKKK34)

ATGTATCCTGGCAAACTAAGTGTCCATCCACCACCTGCCTTTGGTTCATCTCCTAACCTT
GAAGCCCTTGCACTAGAAGCCAACAAAGTAGATGATCAAGATGAGGAACATTCTGTAC
ATGCCCATGCAAAATTTTCCCGACCCATGCATGAAATTACTTTCTCGTCAGATGACAAG
CCAAAACTACTCAGTCAGCTGACTTCCTTGCTTGCGGAGATTGGTTTGAACATCCAAG
AAGCACATGCATTTTCCACAGTTGATGGTTACTCCTTGGATGTCTTTGTTGTTGATGGCT
GGCCTTATGAGGAAACGGAGCAGCTTAGAATTGCATTGGAAAGGAAACTTCTGAAAAT
TGAGAAGCAGCCTTGGTCAGATCATCAATCAATATCTTCTCATGTTTTTGAGCCTGAAA
CGGTGATCAAATGTGAACCTGACCATCTAACTATACCTCATGATGGAACTGATGTATGG
GAAATTGATCTGAAACACTTGAAGTTCGAAAACAAAGTTGCATCTGGATCATATGGTGA
TCTGTACAAAGGTACATACTGTAGTCAGGAGGTGGCCATCAAAATCCTCAAGCCTGAG
CGCATAGATACAGACATGCAGCGAGAGTTTGCCCAGGAAGTCTTTATAATGAGGAAAG
TTCGACACAAGAATGTTGTACAATTCATAGGTGCATGTACCAAGCCTCCAAGTCTGTGT
ATTGTAACAGAATTTATGTCTGGTGGAAGTGTATATGACTACTTGCATAAACAAAAGGG
TGTTTTTAAGCTCCCATCCTTGCTCAAGGTTGCAATAGATGTTTCCAAGGGAATGAACT
ACTTGCACCAAAATAACATAATTCATAGAGATTTAAAAGCTGCAAATCTCTTGATGGAT
GAAAATGATGTTGTTAAGGTTGCTGATTTTGGGGTGGCCAGGGTAAAAGCTCAATCTG
GAGTTATGACAGCAGAAACTGGGACTTATCGTTGGATGGCTCCCGAGGTGTGTATAGTT
ACTTGTTTGAACCTTCTATTTTTGAAATTCTAG

>LOC107427543(MAPKKK35)

ATGTGCTCCAGAAATTGGAAGAAGATCAAAGTTGTTGGTTCAGGTGCTTATGGAACCG
TTCACCTGGCCATACCTCTAAACCTTCGCCTTTCTTCCAAAACCATCGCCGTGAAATCT
TCCACGCTCGAAGACTCCGAGTCTCTTCAAAAAGAACAAAGGATTTTGGAACACTTTT
CGAATTGCTCTGAGATCCTTCAATGCTACGGCAACGAATTGACAATTGAAAACGGCCA
AAGGGTTTACAATTTGCTACTTGAATACGCCAATGGCGGTGACCTAATGGACTTGATCG
AATCCCGACAAGGTAGATTACCCGAATGCGAGGTTCAAATTTACACCAGGATGATATTG
AAAGGTCTTCTTCGCATTCATAAACATGGATACGTTCATTGCGATCTTAAGCCGGAAAA
CATCCTCGTATTTAGGTCTGATTTCGGCGTGCAGCTTAAGATTTCCGATTTCGGATTGTC
CAAAGAACCTGGGAAAGAAGATGATTTCTTTGTGAATTCTAGTAGTGAGTTTAGGTTCC
GTGGAACTCCACCTTATATGTCGCCGGAATCGGTTGCTTTCGGTGAGATAATCGAAGCG
CCGTTGGATATTTGGTCGCTTGGTTGCATAGTGATTGGGATGATTAGTGGAGAGTTCGC
ATGGCCAAGCTTGGACAACAAGGATTTGGTTCGTTGGCTTGCTTTTAGCAATAAAGAG

CCTGAATTGCCACGTAATATGTCTGCCAAAGGGAAAAATTTCTTGAGGAAGTGCTTCAT
AAGAAATCCTAGAGAAAGATGGACGGCTGAGATGCTGTTGGATCATCCATTCGTTGCT
GAAATTGAAGATCACCCACTTAAGTTGGAATCCATCCCTTCTTGCTCACATTCAAAAAA
CTTTTGCAATCTTTCTCTTCATCAAGTTTCAAGGCCCGGCCAATGTGGATTTATGTAA

>LOC107428154(MAPKKK36)

无数据

>LOC107428813(MAPKKK37)

ATGACCGTCGATCTAAACCCTAGATGGGCAATTCAGAACCAAGAAGAAGAAGAAGAA
GAAGAAGAAGAAGAAAATGGTAACAACCTCGGCCGGTTGGCTTTCCCAGAAACCGCT
CTACTTCGTACGAAATCAATTCTTAAAGGGATTAAACGCATTCATGAAGCTGGTTTTGT
GCATTGTGATTTGAAGCCGCAGAACATTTTACTTGTCCCCCAAGAAAACTCCTCTACCA
CTCCTTTTGTGGCCAAGATTGCAGATTTTGGCCTTGCAAAGGCAGCTCAAGGGCTTAC
AAGGAGTTCCGAAGTGAAAGGAACTCTCATGTACTTACCGCCTGAAGCGGAGAACTTC
GGTATCCAAGACCAACCCTCTGACATTTGGTCTCTTGGTTGTACTGTGCTATGTATGTTG
ACTGGAAAAATTTATCCATGGGATTTGAAATCTTGGGAAGGAAATCGTGAGCTTAAGC
AGAAGATTTCAGAGGATTGTCCTGAAATTCCAAATGGGTTATCTAAAAAGGCTAAGGAT
TTCTTGAAGCAATGCTTTATGAGAAGTCCATATGATAGACCCACTGCTGATATGCTGCTG
GGCCATCCATTTATTTGTTCTGGATTGGGCAGAGGAGATGATGAAGATAAAGAAGAGCT
AATGTCTGTCCATTCAGTGCAGGCTTCATCTTGCAAATTGTCTGAGCCTCATGATGAATT
CAGAGCTAGCTTTATACCTCTTCTAAACTCAAGCTTTGGAGAAACAGAGGAGGTCCAA
GGGAAGATAACACCTGTAATCTCGGACAGGCTATCAAGTTCTGCCGTCTGCAGTGCAG
CATAG

>LOC107428906(MAPKKK38)

ATGAGCTCCGACGCACCTGAATCGGCGGCATCCGCATCCTGTTCGGCCTCCTCCGACA
AGGATAAACAGAAAGAGAAAGCGAGGGTGAATAGGACTTCTCTCATACTCTGGCACG
CCCATCAAAACGACGCCGCTGCTGTCCGTAAGCTTCTCGAGGAAGACCGCTCTCTTGT
CCAGGCGAGGGACTACGATAAGCGAACGCCGCTTCATGTTGCTTCACTTCACGGTTGG
ATCGGCGTTGCTAAGTGTTTGATCGAGTATGGCGCAGATATCAACGCTCAGGATCGATG
GAAGAACACGCCTCTAGCAGATGCAGAAGGAGCCAAGAAGCATAGCATGATTGAGTT
GTTAAAGTCCTATGGTGGCTTGTCTTATGGCCAAAATGGAAGTCATTTTGAACCTAAGC
CTGTTCCACCCCCACTGCCCAATAAGTGTGACTGGGAAATTGAACCTTCCGAGCTGGA
TTTCTCAAACTCAGCCATCATTGGAAAGGGCTCTTTTGGTGAGATCTTAAAAGCGCATT
GGCGTGGAACACCAGTAGCTGTCAAACGCATCCTTCCATCTCTTTCAGATGATAGATTG
GTTATTCAGGATTTCAGGCATGAAGTTAATTTACTAGTGAAGCTTCGTCATCCTAACATA
GTTCAATTTCTAGGAGCTGTAACTGACAAGAAGCCACTTATGCTAATCACAGAATATTT
ACGAGGGGGTGATCTTCATCAGTACCTCAAGGAAAAGGGTTCACTTAGTCCTTCAACA
GCTGTCAATTTTGCTTTGGACATTGCTAGAGGCATGGCTTACCTTCACAATGAGCCAAA
TGTGATAATTCACCGAGACCTTAAACCAAGGAATGTTCTTTTGGTCAATTCCAGTGCAG
ACCATTTAAAAGTAGGAGATTTTGGGCTGAGCAAGCTTATCAAAGTTCAGAATTCTCAT
GATGTGTACAAAATGACCGGCGAGACTGGAAGTTACCGGTATATGGCTCCTGAAGTTTT
TAAGCACCGGAAATATGATAAGAAGGTTGATGTGTTCTCCTTTGCAATGATACTTTATGA
GATGCTTGAAGGGGATCCTCCACTTTCAACTTATGAGCCTTACGAGGCAGCGAAATATG
TGGCAGAAGGACATAGGCCTTTTTTCCGCTCCAAAGGATTAATCACTGAGCTGAAGGA
GTTAACAGAGCAGTGTTGGGCTACCGACATGAACCAAAGACCTTCTTTCTTGGAAATT

CTCAAAAGGCTTGAGAAGATAAAGGAAAATCTGCCTTCAGATCATCATTGGAACATATT
TAATGCATAA

>LOC107428931(MAPKKK39)

ATGAGCTCCGACGCACCTGAATCGGCGGCATCCGCATCCTGTTCGGCCTCCTCCGAC
AAGGATAAACAGAAAGAGAAAGCGAGGGTGAATAGGACTTCTCTCATACTCTGGCAC
GCCCATCAAAACGACGCCGCTGCTGTCCGTAAGCTTCTCGAGGAAGACCGCTCTCTTG
TCCAGGCGAGGGACTACGATAAGCGAACGCCGCTTCATGTTGCTTCACTTCACGGTTG
GATCGACGTTGCTAAGTGTTTGATCGAGTATGGCGCAGATATCAACGCTCAGGATCGAT
GGAAGAACACGCCTCTAGCAGATGCAGAAGGAGCCAAGAAGCATAGCATGATTGAGT
TGTTAAAGTCCTATGGTGGCTTGTCTTATGGCCAAAATGGAAGTCATTTTGAACCTAAG
CCTGTTCCACCCCCACTGCCCAATAAGTGTGACTGGGAAATTGAACCTTCCGAGCTGG
ATTTCTCAAACTCAGCCATCATTGGAAAGGGCTCTTTTGGTGAGATCTTAAAAGCGCAT
TGGCGTGGAACACCAGTAGCTGTCAAACGCATCCTTCCATCTCTTTCAGATGATAGATT
GGTTATTCAGGATTTCAGGCATGAAGTTAATTTACTAGTGAAGCTTCGTCATCCTAACAT
AGTTCAATTTCTAGGAGCTGTAACTGACAAGAAGCCACTTATGCTAATCACAGAATATT
TACGAGGGGGTGATCTTCATCAGTACCTCAAGGAAAAGGGTTCACTTAGTCCTTCAAC
AGCTGTCAATTTTGCTTTGGACATTGCTAGAGGCATGGCTTACCTTCACAATGAGCCAA
ATGTGATAATTCACCGAGACCTTAAGCCAAGGAATGTTCTTTTGGTCAATTCCAGTGCA
GACCATTTAAAAGTAGGAGATTTTGGGCTGAGCAAGCTTATCAAAGTTCAGAATTCTCA
TGATGTGTACAAAATGACCGGCGAGACTGGAAGTTACCGGTATATGGCTCCTGAAGTTT
TTAAGCACCGGAAATATGATAAGAAGGTTGATGTGTTCTCCTTTGCAATGATACTTTATG
AGATGCTTGAAGGGGATCCTCCACTTTCAACTTATGAGCCTTACGAGGCAGCGAAATAT
GTGGCAGAAGGACATAGGCCTTTTTTCCGCTCCAAAGGATTAATCACTGAGCTGAAGG
AGTTAACAGAGCAGTGTTGGGCTACTGACATGAACCAAAGACCTTCTTTCTTGGAAAT
TCTCAAAAGGCTTGAGAAGATAAAGGAAAATCTGCCTTCAGATCATCATTGGAACATAT
TTAATGCATAA

>LOC107429056(MAPKKK40)

ATGAAGAGATCAAGAGAAGAAGAAGAAGACAAGGGCAACCTTCTTCATCTTCCTCATA
CTAAGAGAATAAGAGAAGAGCACAGTCTTAGCTCCAAAGGCTGTTCATGGGTGAGAG
GTTCAATGATTGGCAAAGGTGGTTTTGGGTCTGTGTTTCTGGCTTTCAACAACAAACCC
ACTTCAAGCTTCAAAGATTTTCCACCTGTTATGGCTGTGAAATCGGTGGAGGCCACTTC
TTGCTCTGAGCTTGTTAAGGAGAAGATTCTTCTGCAGATTGTCAAGGATTCTCCGTTCA
TCATCCGATTTTATGGCGAAGATGTTACAGTGGGTTACAAGGGTAAAATAATTATCAACT
TGTTCTTGGAGTATGCTTCTGGAGGTTCACTGATGGATTTGATTGAAAAATCCAAAGCA
TCCGAAGGAGTTGGGTTGGTTGAATCTCAAGTAAAAAAGATTACCGAATCAATTCTTA
AAGGGATTAAACGCATTCATGAAGCTGGTTATGTGCATTGTGATTTGAAGCCGCAGAAC
ATTTTACTTGTCCCCCAAGAAAACTCCTCTACCACTCCTTTTGTGGCCAAGATTGCAGA
TTTTGGCCTTGCAAAGGCAGCTCAAGGGCTTACCAGGAGTTCCGAAGTGAAAGGAAC
TCTCATGTACTTACCGCCTGAAGCAGTGAACTTTGGTATCCAAGACCAACCCTCTGACG
TTTGGGCTCTTGGTTGTATTGTGCTATGTATGTTGACTGGAAAAATTTATCCATGGGATT
TGAAATCTTGGGAAGGAAATCGCGAGCTTAAGCAGAAGATTTCAGAGGATTGTCCTGA
AATTCCAAATGGGTTATCTAAAAAGGCTGAGGATTTCTTGGAGCAATGCTTTATGAGGA
GTCCATATGATAGACCCACTGCTGATATGTTGCTGAGCCATCCATTTATTTGTTCTGGATT
GGGCAAAGGAGATGATGAAGATAAAGAAGAGCTAATGTCTGTCCATTCAGTACAGGCT

TCATCTTGCAAATTGTCTGAGCCTCGTGATGAATTCAGAGCTAGCTTTATACCTCTTCTA
AACTCAAGCTTTGGAGAAACAGAGGAGGTTCAAGGGAAGATAACACCTGTAATCTCA
GACAGGCTATCAAGTTCTGCCGTCTGTAGTGCGGCATAG

>LOC107430036(MAPKKK41)

ATGCGCATTGCGCACCCTTCCTCCGGCAAGCCACCGGCGATGGCGAGAGCCGACGAG
AAGTCACCGGTGCGTATTTACAGAGCATTAGAGGATCGATGCGAGAGCTTGGAGAAGA
GCCACGAGAGACTCAAAGAGCAGCTCGATAAGTTGGTGAACGAGAAGAAGAAGGAA
GAGGTGGCGGTGATGATGGAGTCAGATTCAGTCGGAGTGTCGTCGGAGTATCCATTTC
CGGTACGTATTCGGGGATGCTTCGTGTCGGGAAGTCCGTACAGAAGCATTTTGGAATCT
TTGGGTCACGCCGTTCATGTTTGCGAAGCTTCCACGGGAGAAATCATATACTGGAATCG
CTCTGCTGAGAACCTCTATGGGTGGAAGAACTATGAAGTTGTTGGACATGGGGTTGGT
GGTGAAATCCTTATCCCTGAAGAACATTTCGTAGCTCTACAGAAAATCATTGAAAGGTT
GAGAAGAGGGCAGTCATGGTCAGGTCAGTTCCCTTTTAAGAAGAGGTCCGGTGAAATA
TTCATGGCTATAGCGACCAAAAGCCCATTATATGAGAATGGCGTGCTTGTTGGTTTTATC
ACTGTTGCCAATGATGCAGCAGCTTTTAATAGAATGGAAGCACGAAACAGGACACATG
AAGATAGTGCCAATTCCCAACATAGAGGATCGCAGTCATACTTGAAAAGGATTCAGTG
GCATGGACACCCACCTATTGCACCAGTTCCAGAGATAGCTTCATCTGTTTCCAATCTGG
CCTCAAAAATTTTTTTGCGGGGACATGGAGATGATGCATGTAACATATCCAAGGACAGA
GAGGATTCTGTAACAGACACCAAAGATGCCAATGCCAAGAAAACTGGTATCCAAGCAG
CAAAATTCTTGGCAAAGCTGCATATCAAGGGAACCGACAATAGTGGAAAGAAAGATGA
TGGAACAACTGTACAAAATGGTTCAAGTGAGACTTTGTCTATGAGCAATGAGCCTAATT
CTAAAAGCGATTCAAAAAAACCAACTTCATGTGGCTACATAGTTGATACAGATTATAAA
GGAGATGGACACCACAGAAAAATCAAGATATCTCTAGCAGCTAAGCGATATGCACGTG
GGCATGAAATACCAAATTCAACTGAAGATGGTTCTGTTCTAGCCTCTTCAAGGGAGTGC
AAGGAGTGTTTGACAGGATTAGAGTGTGATGAGAATTCAAAGGAAGCAGAACCAGAA
GTAGCCAACTTGAATGCAGTTCAGATAGAAGATGGCAAACAATTCTCAAGTTTAGGGG
GAAGCACTGACAGCAATGGAAGTTCATCAAGCAAAGGGGATAATGAATCTAACACCAT
AGTAGATTGTGAGATTCACTGGGAGGACCTGCATTTAAGGGAGGAGATTGGACAGGGT
TCTTGTGCCATTGTTTATCACGGAATTTGGAATGGATCGGATGTTGCTATCAAGGTGTAC
CATGGGAATCAATATACTGAGGGAATTCTACAAGACTACAAAAAGGAGATTGATATAAT
GAAGAGATTGAGACATCCAAATGTATTGTTGTTTATGGGAGCAGCATATTCACAAGAAC
GACTAGCCATTGTCACAGAGTTCTTACCCAGGGGAAGCCTTTTTAGAACACTTCACAA
GAACAATCAGGCACTTGATATCAGACGGCGTCTGAGGATGGCTCTTGATGTTGCTAGA
GGTATGAATTATTTGCATCACAGAAATCCACCTATAGTGCATAGAGATCTTAAATCGTCT
AACCTGCTTGTTGACAAGAATTGGAGTGTCAAGGTTGGAGACTTTGGCCTGTCAAAGT
TGAAGAATGGGACCTTCTTGACAGGAAAATCTGCGAGAGGGACGCCTCAGTGGATGG
CCCCTGAAGTCCTTCGCAATGAACCTTCAAATGAAAAATCCGATGTTTTCAGCTTTGGT
GTGATTCTCTGGGAACTAATGACTGAATCCATTCCATGGAATCACTTAAATTCTTTACAG
GTTGTTGGAGTTGTAGGCTTCATGGATAGAAGATTAGACCTGCCAGAAGGCCTTGATCC
CCACGTAGCATCAGTCATCCAAGACTGTTGGCAAAGTGACCCACAACAACGTCCATCT
TTTGAAGACATAATCCAAAGAATGATGATACTTCAAAGAGCTGCACCATTGCCTACTCG
GAAAAGCTCAGAACCTTAG

>LOC107431473(MAPKKK42)

ATGGCAATCGAAGAAGAAGTGGAGAGCTGTGGCAGCAGAGCCGTGAGTTCATCGCAG

GGGCAGACTCGGCATCACAGACAGAAACTTGATGTGTATAACGAGGTTCTTCATCGAA
TCCAAGAATCGAATTTTGAAGAGGCTAGTCTTCCTGGTTTTGATGATCAGCTTTGGCTC
CATTTCAATCGCCTTCCTGCCAGATACGCATTGGATGTAAACGTGGAGAGGGCAGAAG
ATGTGCTTACACATAAGAGATTGCTGCAATTAGCTAAAGACCATGCCGATCGACCTGCT
ATTGAAGTTCGAATCGTGCAGGTATATCCTGTTGATTCTGATTCTTCAATGAAAGAAGAT
GCACAGAGTTCTTTTAATAATTCAGGCGGACAGGGGATACATCCACCACCTACCTTTGG
TTCATCGCCTAATCTTGAAGCACTTGCACTTCAATCAAACAGATATCTTGTTGAAGATG
GGGATAGTGCTATGAGCGAGACACCTTACTTTCCTAGGCCTATGCATGAGATTACCTTTT
CAACAGCTGACAAGCCTAAACTCCTTAGTCAGTTAACTTCATTACTTTCTGAGATTGGA
TTAAACATTCAAGAAGCTCATGCTTTTTCAACTGTCGATGGATTCTCTCTGGATGTTTTC
GTTGTTGATGGTTGGCGTTGTGAGGAAACTGAGGAGCTTAGAGGTGCTTTGGAAAGGG
AAATATCAAAATATAAGGAGCAATCTTCTTCAAAACAGAATCCAGGTGCTGTTGATATT
GATATTGGAAATGAACAAGCAAAATTGGAGCCCTTATGTAATGTTTTAGAAATACCGAC
TGATGGAATCGATGTATGGGAAATAGATTCCAGTCTGCTGAAATTTGAGAATAAACTTG
GATCTGGGTCATATGGTGATGTGTATAAAGGATCATATTGTAGTCAGGAAGTTGCTGTCA
AAGTCCTCAAACCTGAGCGTGTCACTGCAGAGATGCTGGGAGAGTTTTCACAGGAAG
TTTACATAATGAGGAAAATTCGGCATAAGAATGTTGTGCAATTTCTTGGTGCATGCACT
CAACATCCAAATCTGTGCATTGTGACTGAGTTTATGTCCAGAGGAAGCCTATATGACTT
TTTGCATAAGCAAAAGGGCGTATTTAATCTTGCTTCTCTACTTAAAGTAGCAATTGATAT
TTCTAAGGGGATGAACTATTTGCATCAGAATAACATAATCCACAGGGACCTCAAGACTG
CCAATCTTCTGATGGATGAAAATGAGGTTGTTAAGGTTGCTGATTTTGGGGTTGCCAGA
GTCCAAGCTCAGTCCGGAGTTATGACAGCTGAAACTGGAACATACCGCTGGATGGCAC
CTGAGGTAATTGAACATAAACCATATGATCACAAGACAGATGTTTTCAGTTTCGGTATA
GTTCTTTGGGAGCTTCTAACTGGAGAACTTCCATATTCATTGTTGACTCCAGTACAAGC
AGCAGTTGGTGTGGTACAAAAGGGTCTACGACCTACAATACCTAAGAACACTCATCCA
AGGGTTGCAGAACTACTTGACAGATGTTGGCAGCAAGACCCAACTCGGAGACCTAAC
TTCTCTGAAATTATAGAGATCCTAAAGCATATAGCGAAGGAGGGGAATGATAAACAAGA
CCGGAACAAGGACAGATCTGCAGGCAGGATTTTTTCTGCACTGAAATGGGTCAATCAC
TGA

>LOC107432147(MAPKKK43)

ATGGAAATGCCCGGTAGGAGATCGAACTACACGCTTCTGAGCCAAGTTCCTGACGACC
AGTTCGGCGGTGGGTCCGCCGCCATGGCTGGAACCGGAGCTACGACTTCTTATTATGAA
TCGGTATCGGGTGAGGGTAAGAACAACAAAGGGAAGGTGGAGAGAGGGTTCGATTGG
GACGCCGTTGGTGATCACAGGGCGAACCAGCAAGGGAATCGGATGTTCTCGTCGATCG
GGTTGCAGAGGCAGTCAAGTGGGAGCAGTTTCGGTGAGAGCTCGCTCTCAGGTGAGT
ACTACGCGCCGACGTTATCGACCACGGCGGCGAATGATATGGATGCCTATGGGTATTTG
CACGAGGATGTATTCAAGATTGGCGGTGGCGGAGGGGATTTGAGAGCCAAGGGGGTT
GATGGGGCTGTTGGGACGGGAGGTTCGTCCTGGGGGAAGAGCTGGGCTCAGCAGACG
GAGGAGAGTTATCAGTTACAGTTGGCTTTGGCACTTCGACTTTCATCCGAGGCAACCT
GTGCTGATGATCCCAATTTCTTGGATCCAGTGCCCGATGAATCCGCGTTAAGGTCGTCA
TCGTCGAGCTCGGCCGAGGCCGTTTCTCATCGATTCTGGGTAAATGGCTGCCTCTCATA
CTTTGACAAAGTTCCTGACGGCTTTTACCTAATTCATGGAATGAACCCATATATATGGAC
TGTGTGCACTGATCTGCAAGAGAATGGTCGTATACCATCACTTGAATCACTAAAGTCTG
TTGACCCTAGTATTGAATCCTCAATTGAAGCAATTCTGGTTGATCGACGTAGTGATCCC

AGCTTAAAGGAGCTTCAGAATAGGGTCCATGGCATTTCTAGTGGCTGCATTAACACAAA
AGAGGTTGTAGATCAGCTGGCAAAGCTTGTGTGTAACCGCATGGGGGGTTCAGCTACC
ATTGGAGAAGATGACTTTGTTTCCTTATGGAGGGAGAGTAGTGATGATCTGAAAGAATG
CTTAGGATCTGTAGTTGTTCCATTAGGCAGTCTATCCATTGGCCTATGCAGACATCGTGC
TTTGTTATTCAAAGTGCTAGCTGATACAATTGATTTGCCATGTCGAATTGCAAAGGGCT
GTAAATATTGTACTAGATATGATGCATCCTCTTGCCTTGTCCGGTTTGGGATTGATAGGG
AGTATTTAGTTGATTTGATTGGGAAGCCAGGTTGCTTATGCGAGCCCGATTCATTGCTCA
ATGGTCCATCATCCATCTTAATTTCTTCACCACTGCGGTTTCCAAGATTAAAACCAGTTG
AACCTATCATTGATTTCAGGTCACTGGCCAAACAATATTTCTCAGATTGTCAATCACTTA
ATCTTGTGTTTGATGAAGCTTCAACAGGTCAAAGTGATGATTCTTCTCTTATTGGTAAA
GTTTCTCATCCAGGTGCTAAAGATAGAGATTCCCAACTGTTTAAAACATGTAATCCTGC
TCAAAACATCTTACACTCAACAACCATGGCCAAAGATCCAGCTATCGGGCGTAGAGAG
GCTATGAGGGTGGATTCTTCCAAGAGCTCAAGGTTATTCGAGGGAAGTCAACTGATTC
CTAGTAAGCCAACTAAAGAGTTTACCCTTGATATAGAAGATTTAAACATTCCATGGTGT
GATCTTGTATTAAAAGAGCGAATTGGAGCAGGTTCTTTTGGAACTGTCCATCGGGCTGA
TTGGCATGGCTCAGATGTTGCAGTGAAGATTCTTATGGAACAAGACTTTCATGCAGAAC
GCTTCAAAGAATTTTTGAGGGAGGTTGCAATAATGAAACGCCTACGACATCCAAATATT
GTTCTCTTCATGGGTGCAGTCACAGAGCCACCAAACTTGTCCATTGTCACAGAGTATTT
ATCAAGAGGTAGCTTGTATAGGCTTTTGCATAAAAGCGGTGCAAGGGAGATGTTGGAT
GAAAGGCGTCGCTTGAGTATGGCTTATGATGTGGCAAAGGGAATGAATTACCTTCATAG
AAGGAATCCCCCAATTGTTCATCGAGATTTAAAATCTCCAAATCTTCTGGTTGACAAAA
AGTATACAGTTAAGGTTTGCGATTTTGGACTCTCTCGTTTAAAGGCGAACACATTTCTAT
CATCGAAGTCAGCTGCTGGGACTCCTGAGTGGATGGCACCAGAAGTTCTTCGTGATGA
GCCCTCAAATGAGAAGTCAGATGTTTACAGCTTTGGTGTAATACTGTGGGAACTTGCCA
CATTGCAACAGCCATGGAGTAACCTAAATCCAGCGCAGGTTGTGGCAGCTGTTGGCTT
TAAGGGGAAAAGACCTGAGATTCCACGTGACTTGAATTCTCATGTTGCTTCTATAATTG
AAGCATGCTGGGCAAATGAACCCTGGAAACGGCCCTCATTTGCCAGTATCATGGAATC
TTTAAGGCCATTGATTAAAGCACCCACAGCTCAACCTGGTCGTCCAGACATGTAA
>LOC107409320 （MAPKKK44）
ATGGAGTGGACCAGAGGTTCCATGATTGGCCGTGGCTCTACTGCTACAGTCTCAGTCG
CCATGGATGTCCCATCTGGCGAGCTCTTTGCTGTAAAATCCACTGAGCTTTCTCATTCC
AAGCTCTTGCAAAAAGAACAAAATTTGCTTTCCAAACTGAGTTCTCCATTTATAGTCAA
GTACAGGGGATTTGATATCAGAAATGAATGTAACCAGCCTATTTACAATCTTTTCATGGA
GTACATACCACAAGGGACTCTCTACGATGATATTCAGAGGCATGGAGGTCGCTTGGAA
GAGTCTTTGATTAGAACTTACACGCGGCAGATTCTTCAAGGTTTGGAATACCTTCATGG
AAATGGGTTGGTCCATTGTGATATAAAGAGCCAGAACATCTTGATGGGGAAAAAGAAT
GTCAAGATTGCTGATCTGGGTTGTGCTAGATTGGTGGAAAGAGTTCCCGGAAATGGAG
ATTTTGGCACGGTGACGTTTTGCGGTACACCGGTGTTCATGGCGCCGGAGGTTTTGCGT
GGGGAAGAACAGGGATTTGAAGCTGACTTATGGGCTCTTGGATGCACAATCATTGAGA
TGGCCACGGGTAAAAGTCCATGGTTGGATGTAGATAACCTTGTATCGGCTCTTCATAGG
ATCGGATTTTCCGATGATGTGCCGGAGTTTCCGAGCTGGTTGTCTAAGAAAGCTAAGGA
CTTTCTGGGCATGTGCTTGAGCAGGGAACCAAAACAGAGGATGACAGCTAGAGAGCT
TCTTGAGCATCCATTTCTCGAAGAGAAAGATTCTCAATCAGATCAAGTAGTGAATGAGT
TCTTTATGAATTCTCCAAACAGTGTGTTGGAAAGCCTTTGGGATTCATTTGAAGTCAAT

GGGAGTCCACAGAATCCAAACCATAAAGGTTCTTCTTCGGATTCGAATTCGAATTCTGC
AGCTGCAAGAATAGAGGAGTTGATTGATGGAGCTACCATGGATCCCTCAGTTTCAAATG
AAGCCAATTGGACTTGGAGTGAAGATTGGATCGAAGTTAGAAGCAACAATGATGAGGC
AAACAATGAGTTTTCTGATAAGATGGAAGTGGTATTGCCTACTAATGAACCACCATTAG
CATGTTCATTGCCTGATTGTTCAAGTATCAATATTGAAGAAGAGCTTGGTAGATTAGGGT
TTGATGAAGATTTCTTGTTTAAATTCTCATTTGGACATGTCAAAATTGTTAGAACTGAAA
GAGGATTTGTGATTACATTTGAGATTACAGAAAACACTATTGTATTTAGAAACAAAAAT
TTTATGACGCAGGAAATGAAATTTTTTCTTTTCTTCAATAT

>LOC107408109（MAPKKK45）

ATGGAAAATATAACGGCGCAGTTGAAGCGGGGAATCTCGCGGCAGTTCTCGACGGGGT
CGCTGCGCCGGAATCTGAGCAGGCAGTTCACGCGGCAGTCGTCGCTGGACCCGCGGC
GTCACAACCTCAGGTTCAGCTTCGGCAGGCAGTCTTCGCTCGATCCCATTCGCCGGAG
CCCCTCCGATGATGCCGACCTCACCGTCCCTGAGAACCTTGACTCCACCATGCAGCTTC
TCTTCATGGCCTGCCGAGGAGATACTCTTGGCGTCCAGGATTTGCTCGATGATGGTACC
GATGTTAATAGCATCGATTTGGATGGTCGCACTGCTCTCCATATCGCTGCCTGTGAAGGC
CACGTTGACGTCGTCAAGCTCTTGCTCAGCCGCAAGGCCAATATTGATGCTCGTGATCG
CTGGGGCAGTACGGCAGCTGCTGATGCTAAGTACTATGGACATACAGAAGTTTACAACA
TTTTGAAGGCTCGTGGAGCTAAAGCTCCGAAAACCAGGAAGACGCCAATGGCAGTTG
CAAATCCTCGAGAAGTTCCAGAGTATGAGCTTAATCCGCTAGAGCTTCACATAAGGAA
GAGTGATGGTATTGCAAAGGGAACATATCAAGTGGCTAAATGGAATGGTACAAAGGTT
GCTGTAAAGATACTAGATAAGGACAGCTACTCGGACTCTGAAAGCATAAACGCATTCA
AACATGAGTTAACCTTGTTAGAAAAGGTCCGCCATCCTAATGTTGTTCAGTTTGTTGGA
GCTGTGACACAAAATATGCCTATGATGATTGTTTCAGAATATCATCCAAAAGGTGACTT
GCGAAGCTATCTTCAGAAGAAGGGACGGCTATCTCCATCTAAAGCCCTTAGATTTGCTC
TTGACATTGCCAGGGGCATGAATTATCTTCATGAATGTAAACCAGACCCAATTATTCACT
GTGACTTAAAGCCAAAAAATATTTTGCTGGATAGTGGAGGTCAATTAAAGGTAGCCGG
GTTTGGTGTAATAAGGTTGTCAAAAATTTCACCTGACAAAGCAAAACTAGCGCAGGGT
GGTGGTAACATTGACCCTTCAAATATATATGTGGCACCTGAGATTTATAGAGGTGAAATA
TTTGACAGAAGTGTGGATGCGTATTCTTTTGGTCTCATTTTATATGAGATGATTGAGGGC
GCACTGCTATCCCAACCCAAGCCTCCAGAAGAGACTTTGAGAATGTTGTGCATAGACG
GACAAAGACCACCATTCAAGAGCAAATCAAAAAGTTATCCTCCAGATGTAAAAGAGTT
GATTGAGGAATGTTGGGATCCTGAGCCTGTAGTTAGGCCAACTTTTTCAGAGGTCGTTG
TACGGTTGGACAAGATAGTTTCAAATTGCTCAAAGCAGGGATGGTGGAAAGACACTTT
TAAGCTTCCTTGGAAATAG

>LOC107405634（MAPKKK46）

ATGGAATTGGAGGTAAAGCCACAAGAGCAACCACATCCTCAGGCTGGGGTGGCTAGAT
TCACTCTAGGCAAGCAGTCGTCGCTGGCACCGGACCGGAGTACAGATTCCGCCGGAAC
GGCGGTGGATGAGGTGATCGATCCGCGGGTGAAGCTGATGTACTTGGCCAATGAAGGT
GACTTGGAAGGGATTAAAGAGCTCTTGGACTCCGGCACCAATGTCAATTTCAGTGACA
TCGATGGCCGTACAGCTTTGCATATCGCCGCTTGCCAGGGACTATCCGACGTCGTTCAG
CTCTTGCTCCATCGAGGCGCCAAGGTCGATCCTCAAGATCGCTGGGGAAGCACGCCTC
TTGCAGATGCAGTGTATTACAAAAACCATGACGTGATCAAACTTTTAGAGAAACATGGT
GCAAAGCCTCCGATGGCTCCCATGCATGTTGAAAATGCACGTGAAGTCCCAGAATATG
AGATCAGTCCAAGTGAACTTGATTTTTCTAATAGTGTTGACATAACAAAGGGAACCTTC

CGCATAGCATCTTGGCGTGGAATTCAAGTTGCAGTTAAAACACTAGAGGAAGAATTATT
CACTGATGAGGATAAAGTAAAGGCATTCACAGATGAGCTTGCATTGCTTCAGAAGATA
CGCCATCCAAATGTTGTCCAATTTTTGGGTGCTGTCACACAAAGCAGTCCGATGATGAT
TGTCACAGAATATCTACCCAAGGGAGACCTCTATGCATATTTAAAAAGAAAAGGTGCTC
TAAAGCCAGCAACAGCGGTGAAGTTTGCTCTTGACATTGCTAGGGGCATGAATTATTTA
CATGAGCATAAACCTGAAGCAATTATTCATCGAGATCTTGAGCCTTCAAATATATTGCGG
GATGATTCTGGGCATCTGAAAGTTGCAGACTTCGGGGTGAGCAAACTACTGAAAGTTG
CAAATACAGTTAAAGAAGACAGACCTGTCACTTGTCAAGACACTTCTTGGCGATATGT
GGCTCCTGAGGTTTACAGAAATGAAGAATATGATACTAAAGTTGATGTGTTTTCATTTG
CTTTGATCTTGCAAGAGATGATTGAAGGTTGTCCACCATTTTCCACAAAGCCAGAAAA
AGAAGTTCCTAAAGCATATGTTGCAAATGAGCGCCCACCATTTAGAGCTCCACCAAAGT
ACTATGTGTATGGGTTGAAAGAGTAA
>LOC107407393(MAPKKK47)
ATGAGTTGCAGTGAGAGGTACAGAGGAGGTGAAGAGAGGGAATATGAGCACCAGGCT
TTGCGGAGGTCTGTTGATGAAGCTGAACCCAATTTGGTATCCCAAAATGGTTCCCTAAC
GACCCAACAATTGACGATCGATGAGAACTTGCTGGTCGACCCGAAAGTTCTGTTTATT
GGGTCCAAAATTGGTGAAGGAGCTCATGGCAAAGTCTATGAAGGAAGGTATTGTAATC
GGATTGTTGCTGTTAAAGTTCTCCATCGTGGAAGCACTTCGGAAGAAAGAGCTTCGCT
GGAGAATCGATTTGCTCGTGAAGTTAACATGATGTCTCGAGTTAAACATGAGAACCTTG
TTAAGTTTATTGGAGCTTGTAAAGATCCTTTAATGGTGATAGTTACTGAACTATTACCTG
GGATGTCACTCCGGAAGTATCTAGTTAGTCTTCGTCCTGAAAAATTAGATCTTCACGTG
GCGATAAAATATGCCATTGACATTGCTCGTGCCATGGAATGTCTGCATGCCAATGGGATT
ATACACAGAGATCTGAAACCTGACAATTTGTTGCTTACGGCAAATCAAAAGTCTGTTAA
GCTTGCAGATTTTGGTCTGGCAAGAGAAGAATCTGTGACTGAAATGATGACTGCAGAA
ACTGGTACTTATCGGTGGATGGCTCCTGAGTTGTACAGCACTGTGACATTGCGTCAAGG
AGAGAAGAAGCATTATAATAACAAGGTTGATGTATACAGTTTTGGAATTGTCTTATGGG
AACTATTGACAAACCGCATGCCATTTGAAGGCATGTCCAACTTGCAAGCTGCTTATGCT
GCTGCTTTCAAGCAAGAGAGGCCTAGTCTTCCAGAGGATATATCCCCTGATCTTGCATT
TATCATACAGTCATGTTGGGTTGAGGACCCTAACTTGAGGCCCAGCTTCAGCCAGATAG
TCCGCATGCTCAATTCATTTCTCTTCACACTTTCACCATCACAATCATCCTTACCGGATT
CCGACGATGTTAACGAGGCAGCAGCTAGTAATGTTTCTATGAATGACTTATCTGCTCGA
ACAAGAGGAAAGTTTGCTTTTATTCGTCACCTTTTCGCTGCTAAGAGGACAAAGAACT
TGCAATGA
>LOC107406964(MAPKKK48)
ATGGTTTTTGTTTTGCATCTGGTTTTGCTGCAGTTATCTCACTCCATTAGTTTTT
GGTCTATTGGCTTACTTTTCAGATACAAAAACCAGACTGTTGCAGTTAAAATTGTTCAC
AATGGAGAAACTCCAGAGGAGATCGCCAAGAGGGAAGCACGGTTTGCAAGAGAGGTT
GCAATGTTGTCTAGAGTTCAACATAAAAACTTAGTGAAGTTCATTGGTGCTTGTAAGGA
ACCGGTCATGGTGATAGTTACTGAGCTTTTATTGGGAGGGACATTGCGTAAATACTTATT
GAACACGCGACCAAGGTGCTTGGACACACGAGTTGCTGTTGGTTTTGCACTTGATATT
GCTCGTGCCATGGAGTGCCTACACTCTCATGGGATTATACACCGCGATTTGAAACCTGA
GAACTTACTCTTGACTGCAGACCACAAAACAGTTAAACTAGCAGATTTTGGTTTAGCA
AGAGAAGAGTCTTTGACAGAGATGATGACTGCTGAAACAGGGACATATCGTTGGATGG
CTCCAGAGTTGTACAGTACTGTTACTTTGAGGCAGGGAGAGAAGAAGCATTACAACCA

TAAAGTGGATGCCTATAGCTTTGCAATTGTTTTGTGGGAGCTCTTACACAACAAATTAC
CTTTTGAAGGCATGTCAAATCTCCAGGCAGCGTATGCGGCTGCTTTTAAGAATGTTAGG
CCCAGTGCTGAAAACCTCCCAGAGGAAGTGGCTCTTATTCTAACTTCTTGCTGGCAGG
AGGACCCAAATGCTCGGCCTAATTTCAGCCAAATAATCCAAATGCTACTTAATTACCTTT
ACACCATCTCTCCTCCTGAACCCGTGATTCCTTCTCGGATTTTTGCTTCTGAGAACACT
GTCTTGCCACCGGAGTCTCCTGGTACAAGCTCATTGATGGCAGTGCGAGATGATTCAG
AGGAGACACCAAAAGCAAAGATGGAAAACAAGTCGAGAGGACTTTTCTTCTGCTTCA
ACCAGTGTTATTAA

>LOC107404883(MAPKKK49)

ATGGAAAACTTCAAGTATCCATCTTCACCACCTTCCAAGAGAATGAGGGGAGCAGAAA
CAATAGATTCAGAAGGTCCGTACAGGCTGCTATACTGTTCAAGTAAGGGAGACACAGA
AGGGGTAGTGCAGGAATTAGAGAAAGGTGTGATTTCTAATCTGGCTGATTATGATAAAA
GAACTGCTCTCCATCTGGCATCGTGTGAAGGTTGTACGGAGGTTGTTGTTCTACTTCTA
GAGAAAGGTGCAGATGTCAATTCCATTGATCGTTGGGGGCGAACTCCACTGTCAGATG
CTCGTAGCTTTGGTCATGAAAGCATCTGCAAGATATTGGAGGCTAATGGTGGAATTGAT
CCGGTGGGGCTTGACTCCCAGATTCCATGCTATACTATTGATTATTCTGAGGTGAACATG
AATGACGCAATACTTATTGGAGAGGGGTCATATGGTGAAATATATTTGGTGAAATGGAG
AGGTACAGAAGTTGCTGCAAAGACGATTCGCTCCTCTATTGCATCAAATCAAAGGGTC
AAGAATTCCTTTATGAAAGAACTTGCTTTGTGGCAAAAGTTGCGCCACCCCAATATAGT
TCAGTTCCTTGGTGTTCTAAACAATTGTAGCCGATTGATCTTCCTTACCGAGTATCTTTG
CAATGGAAGTTTGCATGACATATTAAGAAAGAAAGGAAGACTTGATCCTCAAACTGCA
GTTGCTTATGCTCTTGATATTGCCAGAGGTATGAATTATCTCCATCAGCATAAATCACATG
CTATAATTCACAGAGATTTGACCCCAAGGAATGTGTTACAAGATGCAGCTGGCCGACTC
AAGGTTACTGATTTTGGATTGAGCAAAATTGCACAGGAAAAAGATGTTTTAGGTTATAA
AATGACTGGAACAACTGGTTCCTTTCGATACATGGCACCGGAGGTATATCGCCGTGAAT
CTTATGGGAAAGGTGTTGATGTCTTCTCCTTTGCTCTAATCGTACACGAGATGTTTCAA
GGAGGACCATCAAATCTGGCAGATGATCCAGAACAAGTAGCAGATAAACGAGCATATG
AAGATTCCAGACCGCCTCTCTCCTCTTATGTATACCCTGAACCAATTAGGATACTTCTTC
AAAAATGCTGGCACAAGAATCCGGATTTCAGACCTACATTTGAAGATATAATTTTAGAG
CTTGAGTCTATTCAAGACAAGTTTCAGACTACCGAACCAATGCCTTCAAGTTGCAGGT
GTTCCATCCTATGA

>LOC107406505(MAPKKK50)

ATGGGAAAAATGGAATCTGGGAGTAGATTTTTCTCAGCTGATGAGTTCAGATTAGATGC
AAAATGGCTGATTGATCCAAAACATCTCTTTGTTGGACCAAGGATTGGAGAGGGAGCC
CATGCTAAAGTGTACGAGGGCAAATACAAAAACCAGACTGTTGCAGTTAAAATTGTTC
ACAATGGAGAAACTCCAGAGGAGATCGCCAAGAGGGAAGCACGGTTTGCAAGAGAG
GTTGCAATGTTGTCTAGAGTTCAACATAAAAACTTAGTGAAGTTCATTGGTGCTTGTAA
GGAACCGGTCATGGTGATAGTTACTGAGCTTTTATTGGGAGGGACATTGCGTAAATACT
TATTGAACACGCGACCAAGGTGCTTGGACACACGAGTTGCTGTTGGTTTTGCACTTGAT
ATTGCTCGTGCCATGGAGTGCCTACACTCTCATGGGATTATACACCGCGATTTGAAACC
TGAGAACTTACTCTTGACTGCAGACCACAAAACAGTTAAACTAGCAGATTTTGGTTTA
GCAAGAGAAGAGTCTTTGACAGAGATGATGACTGCTGAAACAGGGACATATCGTTGGA
TGGCTCCAGAGTTGTACAGTACTGTTACTTTGAGGCAGGGAGAGAAGAAGCATTACAA
CCATAAAGTGGATGCCTATAGCTTTGCAATTGTTTTGTGGGAGCTCTTACACAACAAATT

ACCTTTTGAAGGCATGTCAAATCTCCAGGCAGCGTATGCGGCTGCTTTTAAGAATGTTA
GGCCCAGTGCTGAAAACCTCCCAGAGGAAGTGGCTCTTATTCTAACTTCTTGCTGGCA
GGAGGACCCAAATGCTCGGCCTAATTTCAGCCAAATAATCCAAATGCTACTTAATTACC
TTTACACCATCTCTCCTCCTGAACCCGTGATTCCTTCTCGGATTTTTGCTTCTGAGAACA
CTGTCTTGCCACCGGAGTCTCCTGGTACAAGCTCATTGATGGCAGTGCGAGATGATTCA
GAGGAGACACCAAAAGCAAAGATGGAAAACAAGTCGAGAGGACTTTTCTTCTGCTTC
AACCAGTGTTATTAA

>LOC107405705(MAPKKK51)

ATGAAGGAAGGCAGTGATGGGTTTGTGAGAGCGGATCAGATTGATCTGAAAAGCTTAG
ATGAACAGCTTGAGAGGCATATGAGCAGGGCTTTGACCATGGAGAAAAGCAAGAAGC
TGAGAGATGAACAAGGTAATACCAACAATTTTCTTACTAGTACTGCTTCTACCACCACC
ACCACCACCACCATGTCTTTTCTAACCCCCAAGAAGCAGAGGCAAGAATGGGAAATCG
ATCCCTCCAAGCTCATCATCAAGGGCGTCATTGCACGTGGCACCTTCGCCACCGTCCAC
CGTGGCGTCTACGACGGTCAGGATGTTGCCGTGAAATTGCTAGACTGGGGTGAAGAGG
GTCACAGGGCAGAAGCTGAGGTTGCTTCACTAAGGGCAGCTTTTACACAAGAAGTTG
CTGTCTGGCATAAACTTGATCATCCGAATGTTACTAAGTTTATAGGAGCAAAAATGGGC
TCGGCAGAACTACAAATTCAGACTGATAATGGTCTAATTGGCATGCCTAGTAATATCTGT
TGTGTTGTTGTGGAATATCTGGCTGGGGGTACTCTGAAATCTTACCTTATAAGGAATATG
AGAAAGAAGTTAGCTTTTAAGATTGTTGTCCAGCTGGGTCTGGATCTTGCTAGAGGTTT
GAGTTACCTTCACTCGCAGAAGATTGTTCACAGAGATGTAAAAACGGAGAACATGTTA
TTGGACAAGACACGTACAGTAAAAATTGCCGATTTTGGGGTTGCTCGTGTTGAAGCTT
CAAATCCTAATGACATGACTGGGGAGACTGGAACACTTGGTTACATGGCTCCTGAGGT
TCTCAATGGCAATCCATATAACAGGAAATGTGATGTCTACAGTTTTGGCATCTGTTTATG
GGAAATATATTGCTGTGACATGCCATATCCCGATCTTACTTTTTCAGAAGTAACTTCAGC
TGTGGTTCGCCAGAATCTGAGGCCAGATATACCGAGATGTTGCCCAAGTTCCCTAGCAA
ATGTAATGAAGCGATGCTGGGATGCCAGTCCAGAGAAGCGGCCAGAGATGGATGAAGT
TGTTTCAATGTTGGAGGCAATCGACACATCAAAAGGTGGCGGCATGATTCCTCCGGAC
CAGGCTCAGGGTTGTCTCTGTTTTCGCAAGTACAGAGGGCCGTGA

>LOC107403422(MAPKKK52)

ATGGATTTGACTGAAGGAGTGGGAGAGAGTTCGTCACCGCCTAGAAGTTTCGGAAGCT
ATAGCAACTATGATGTGAGGAACGATGTTTACAATCGGTTAGTGGAGAGCGGCAACGA
AGACGCTGTTAGCAATCCTGAGTTCCGTGAACAGTTAGACTCTCACTTCAATCGCTTGC
CTGCTAGTTATGGACTCGATGTTAACATGGATAGAGTGGAAGATGTTTTATTACATCAAA
GGCTCCTTGCCTTGGCAAAAGACCCGGAGAAGCGGCCTGTTTACCATATCCGTTTCTTG
GAGAATATTTCTACTAGAACAGAGGATAATGGCAATCAACTATTTACAAGTACTCTTACA
ACACCTAGGTCATTGTGTGATGCAGCTAATGAAGAAGTTCCTGTGTCACATAAGAGCAA
TCATGCAATTGACTTTGAACCCTGTTCTAAGCTTGAGGACTTAAATTTGGATGTTAGAA
ATAAATCCAAGGACGTGGAGGAAAGATATACAATGGTGAAACTTCCCAGAAGGGAAGA
TGTTGCTCCTATTCCAATCCATGAAGTGATATTCTCTACCATTGACAAGCCTAAGCTTCT
TAGCCAGCTTTCTGCTTTGCTGTCTGATATAGGGCTGAACATCCGTGAAGCACACGTTT
TCTCAACAACTGATGGCTACTCCTTGGATGTATTTGTGGTCGATGGATGGCCAGTTGAG
GATACAGATGGTTTATGGGAAGCTATGGAAAAAGCAGTTGCTAGAAGTGAGGGTTCAT
GGTCTAGATCTTCACAATCTCATTCAGCCGTGGAAAAAGCATTGACAGTACAGGCAAA
ACTTGGAGATTGGGAAATTGATAGAAGATTATTGAAAATGGGAGAACGAATTGCATCG

GGATCCTGTGGAGACTTGTACCATGGAGTTTATCTTGGTCAAGACGTTGCAGTTAAGA
TTCTTAGGTCTGAACATTTGAATGATGCTGTAGAGGATGAGTTTGCTCAAGAAGTGGCA
ATTCTCAGGGAAGTCCAGCATAAAAATGTTGTTCGTTTTGTCGGAGCATGTTCACAGTC
TCCACATTTGTGCATAGTGACAGAGTATATGCCTGGAGGAAGTCTATATGACTATTTGCA
CAAGAACCATAACGTGATGAAGCTCCCGCAGCTGCTAAAGTTTGCAATCGATGTCTGC
AGAGGAATGGAGTACTTGCATCAAAATAACATAATTCACAGGGATCTGAAGACGGCAA
ATTTGCTTATGGACACTCATAATGTTGTTAAAGTGGCAGATTTTGGGGTAGCTCGTTTCC
AAAATCAAGGAGGAGTAATGACGGCAGAAACTGGAACATATAGATGGATGGCGCCTGA
GGTTATAAACCATCAACCATATGATCAAAAAGCAGATGTATTCAGTTTTGCAATTGTACT
CTGGGAGCTAGTGACAGCCAAGGTTCCATATGATTCTATGACTCCATTACAAGCTGCAC
TTGGAGTGAGACAGGGACTGCGTCCAGATCTTCCCGATAATGCACACCCCAAACTGTT
AGAATTAATGCAAAGGTCCTGGGATGCTGTTCCTAGCAATCGGCCTTCCTTCTCTGAGA
TAGCAGCTGAACTTGAAAATCTGCTTCAGGAAACTCCAGAAGCAGCAAATGGAACCT
GA
>LOC107435406(MAPKKK53)
ATGGAGGAAGACGCTAGCTCTTGGATCAGAAGAACAAAATTTTCCCACACTGTTTGTC
ATCGTTGGGACTCTTCAAGATTGGCCTCCGTTCCTTTCATTGTTCAGATAGACTGGATTT
CGGGGTTGAACTCTAGACCCACAAACAGGGAAACTCCTGGTAATCATAACGAAAATCC
AAGTTATTCACAGATCCAGAGAAATCCCATTACCAACAAGCAGAGATCTTTATCACCTC
TACCGGAGACTCTACTTTCCGATGAGTTTAAGGAAGCTCGATCTGATCGGAAGAGATTC
TCAACTCCCAACCGTAGGAGGAAAGAGATAGACAAGGGAATTGTGGGAAATGTTTTCC
ACAAGGATTCCCATGTATCCAAGGCATGGAACTCGAGTACTAGCCCTCTCAGGCATCTA
GCTTCCATGAAAGTTAATAACAAGTTGAAGAACCGGAAGGAATCGCCGTGGGCCAAGT
ATTTTGATCATACTGGTGGAAGGGTTACTGCTGTGGAAGCAGCAGATGAATGGAGTGTT
GATATGTCACAGTTATTTCTTGGGCTTAAATTTGCTCATGGAGCTCATAGCCGGCTTTAC
CATGGGATTTACTTTGATGAACCTGTTGCCGTTAAGATTATTAGGGTCCCAGATGATGAC
GAAAGTGGAGCCTTGGCAGCACGATTAGAGAAACAGTTTAGCAGAGAAGTTCATCTTC
TCTCTCGTCTTCACCATCAAAATGTCATAAAGTTCAAAGCAGCATGCAGAAAGCCACC
AGTTTATTGTGTCATCACCGAATATTTATCAGAGGGTTCCTTGAGAGCATACTTGCACAA
ACTCGAACATAAATCTCTACCGTTACAGAAGCTAATTGTTATTGCCTTGGACATAGCAC
GTGGAATGGAATTCATTCACTCACAACGTGTTATTCATAGGGACTTAAAGCCTGAAAAC
ATTCTTATTGATCGAGACTTCCGCCTGAAAGTTGCTGATTTTGGTATAGCATGCGAGGA
GGCATATTGTGATTCTTTGGCAGATGACCCAGGCACTTACCGATGGATGGCACCTGAGT
TGATCAAGCATAAATCCTATGGGCGGAAAGTTGATGTGTACAGTTTTGGATTAATTTTAT
GGGAAATGGTGGCAGGGACAATCCCATATGAGGATATGAACCCCATTCAAGCTGCATTT
GCCGTAGTGAATAAGAATTTGAGGCCTGATATCCCGGGGGACTGTCCGCCTGCTATGCG
AGCTTTGATCGAACAGTGTTGGTCCTTGCAACCAGATAAAAGGCCAGAGTTTTGGCAG
ATAGTTAAGGTACTAGAACAATTTGAATCTTCACTTGCATATGATGGAACTTTGAGTCTT
GTGCTTAACCAAACTGGCCATGATCATAAGAAGAAACTTCTTCTCTGGATTCAGAAGCT
TGGTCCTGTGAATTCTAATAGTTTGTCAATGTCAAAGCCAAAACCAAGGCGAAAATTAT
ATTGA
>LOC107434197(MAPKKK54)
ATGAATTGGGTTTTGTTATTGATTTTCCGAGTAACCAAAGGATCCTCCTTGTTGTTTCTA
CCCATTTTGGTAGAACACAAAATGGATGGAAATCATCAATGGAGATGCCCTGTTCCAGG

TGAATGGGTGAGGGGAAAATTGGTGGGTTCAGGTTCTTTTGGGACCATCCATATGGCTT
TGGCAAAATCTACCGGAAGACTTTTCGTTGTGAAATCTGCACAATCTGGTATTGGTGTT
CAAGCTTTAGAAAACGAAGCCGATATCCTTGAGAGTTTGAATTCCCCACACATAGTCCA
TTGCATAGGAAAGGAATTCTCAATTGACAAAAATGGTGAGCAACAAAATTACAATGTG
TTCTTGGAGTACATGGCAGGAGGAAACTTGTTGGATGTGGTACACATATTTGGTGGGTC
CCTAGATGAGCAAGTTATTAGACTTTACACCAAAGAAATTCTTCTGGGTCTCAAATATC
TTCATGACAATGGGATTGTTCATTGTGATTTGAAATGCAAAAATGTGCTCCTGAGTTCC
TCTGGAAATGTCAAACTGGCAGATTTTGGGAGCGCAAAGAGGATGACCAAAAAAACC
ACCGATGATGAGGGATTTGTGGACTCTTGGCAAAACATTGTTGGAACACCCTTGTGGA
TGGCACCAGAAGTTTTGAGGAAAAAAGAGTTAGATTTAGCTTCGGATATTTGGTCACT
GGGATGCACAGTCATTGAAATGGCAACAGGAAACCCTCCCTGGGATGTGGAAATCACT
TCCAATCCAATGGCTGCAATTCTGAAGATTGCTTGTGGAAATGACAAACCTCAATTTCC
TACAAAGTTTTCGCAAGTGGGTTTGGATTTCTTGGCCAAGTGTTTGGAAAGAGATCCA
AGAAAAAGGTGGAAGGCTGAAGAACTGCTTAACCATCCGTTTGTTTCTGGGGAAAATT
CATCATTGAGAAAATCATCAAGAGAGGAAGTAGAATTAGTGTCATTCTCACCAGCAAG
TGTGTTAGACATTAATGTTAATGGAATATTATTATATGGAGAGGGATCAGATTCAGATCA
GGAAGCAGAAACAAAAACAGAAGCAGAGGATAGTAGATTAATAAATCCATTCGCAAG
GAGGTGTCATGAAGGAAATTGGATGGCAGCAAGACAACAGAGAGACAATCATTTTGAT
TCTTCAGAAAATTGGATTACTGTTAGATGA

>LOC107435407(MAPKKK55)

ATGGAGGGAGAGGTTAGCTCTTGGATTAGAAGAACAAAATTTTCCCACACTGTTTGTC
ATCGTTGGGACTCTTCAAGATTGGCTTCTGTTCCTTTCAATGTTCAGATAGACAGGATTT
CGGGGTTGAAATCCAGACCCCCAAACAGGGAAGCTTCTGGTAATCATAAAGAAAATCC
AAGTTATTCACAGATTCAGAGAAATCCCGTTACCAACAAACAGAGATCTTTATCACCTC
TACCGGAGACTCTACTTTCCGAAGAGTTTAAAGAAGCTCGATCTGATTGGAAGAGATT
CTCAACTCCGAACCGTAGGAGAAAAGAGATAGACAAGGGAATTGTGGGAAATGTTTTC
CACAAGGATTCCCAAGATTCCCAAGTATCCAAGGCATTGAACTCGAGTACTAGCCCTCT
CAGGCATCTTGCTTCCATGAAAGTTAATGACAAGTCGAAGAACCGGAGGGAATCGCCA
TGGGCCAAGTATTTTGATCATGCTGCTGGAAAGGTTACTGCTGTGGAAGCAGCTGATG
AATGGAGTGTTGATATGTCAAAGTTATTTCTTGGTCTTAAATTTGCTCATGGAGCTCATA
GCCGGCTTTACCATGGGATTTACTTTGATGAACCTGTTGCCGTTAAGATTATTAGAGTCC
CAGATGATGACGAAAGCGGAGCTCTGGCAGCTCGTTTAGAGAAACAGTTTAGCAGAG
AAGTTAATCTTCTATCTCGTCTTCACCATCAAAATGTTATAAAGTTCAAAGCAGCATGCG
GAAAGCCACCAGTTTATTGTGTCATCACTGAATATTTATCAGAGGGTTCCTTGAGGGCA
TATTTGCACAAGCTCGAGCATAAATCTCTTCCTTTACAGAAGCTAATTGCTATTGCCTTG
GACATAGCACGTGGAATGGAATTCATTCACTCTCAACATGTTATTCATAGGGACTTAAA
GCCCGAAAACATTCTTATTGATCAAGACTTCCGCCTAAAAGTTGCTGATTTCGGTATAG
CATGCGAGGAGACATATTGTGATTCTTTGGCAGATGACCCCGGCACTTACCGATGGATG
GCACCTGAGTTGATCAAGCATAAATGCTATGGGCGGAAAGTTGATGTGTACAGTTTTGG
ATTAATCTTATGGGAAATGGTGGCAGGGACAATCCCATTTGAGGATATGAACCCCATTC
AAGCTGCATTTGCCGTAGTGAATAAAAATTTGAGGCCTATCATCCCAGGGGACTGTCCG
CCTGCTATGCGAGCTTTGATCGAACAATGTTGGTCCTTGCAACCAGATAAAAGGCCAG
AGTTTTGGCAGATAGTGAAGGTGCTAGAACAGTTTGTAACGTCGCTAGCATACGATGG
AACTTTGAGTCTTGTGCTGAACCAAACTTGCCACGATCATAAGAAGCAGCTTCTTCGC

TGGATCCAGAAGCTTGGTCCTGTGCATTCTACTAGTTCATCTATGCCAAAACCAAAACC
AAAATTCTATTGA

>LOC107435014(MAPKKK56)

ATGCATCACATACCACGAATTTTCAACCGTAGCAAGCGAGAAAAATCCATGGATCCGA
AGAAGAACCCTAGGAAGCCGAGGCTTGAGCGCCGCAATGCTGCGAAGCACATTGATT
ACGAAGCTTCCACGTCTTCTTCGTCGATGGAGTCGACGCCGTCGCCGTCGCTCCACAC
TCGATCGATGGATTTGTCGGACAAGATGAGCTTCCGTGTCGAGGGAATTGACGGCGAG
TTCGACGTAATATGCCGGACCTTGGGGCTTTCGGGTATCGAGGATTTCGCTATCCCGTC
GGCGGCATGGGAGGCTCGGAAGATCCGATCGATCTCCGATATTCTACCTAGGTCCAGGC
TTAATCGGTTAGATAGTTTGAGTGGTTCAGCCAGGGAGGAACTGAAGGATGAGGTTATT
CAAGCTGTGGCCGAATTGAAAGATAGGGTCGTCGGTAGCGTTAGAATTAGGGGTGGTA
ATAATGAGTTGCCTCAGGCTGATCTTGCCGAGTCCCGTGGTTGTTGTGTTGCTAACGAT
GGAGCGTGCGATGGTGATTTCATCGGTGGTGGTGGTGGTGGGGGAGGAGGCGGAGGA
GGAGGAATTAAGGGTGTTCGGCCGCCGGTGCTTAAGCCTCCTCCTTCGATGAGGCTTC
CGGTGATTGATAAGACGTGCTCGACTTGGGACATTCTGAGGGACTTTGCGCCCGATGA
AGAAACATCACCTGCGCAACTTCTCCATAAAACGTATAATTCTTCCGATGAAGAAGAA
GAAAAAGAAGAAGAAAAGCACCACCCAAGAGAAGAAAACCTCCAAGAAGAAGAATC
ACATCAAGAAGAAGAACAAGTCCACACGGAAGAAGTTGTTAGGCAAAGAGAGGAGT
CTGCGGTGAGGCTTCCGGAGAATGCGTTACTCTCCGAGTCGTGTTCGTTCACTACGTCT
AATGACGACGACTCTTCTAGCAGTACCACAGAGCCCACGTCGAATATTTCGCCGAATG
CGAGATTAAGGCCCATCATCACGTATTGGGAAAAGGGCGATCTTCTGGGTCGCGGATC
GTTTGGATCTGTTTATGAAGGAATAACTGATGATGGGTACTTTTTTGCTGTGAAGGAGG
TTTCCTTGCTTGATCAGGGAAGTCAAGGAAAGCAAAGTGTTTATCAACTTGAGCAGGA
GATTGCACTGTTAAGCCAGTTTGAACATGAAAACATTGTTCAATATTTGGGCACTGATA
AGGATGAATCAAAACTTTATATTTTTCTAGAACTTGTAACTAAAGGTTCTCTGCAAAGT
CTTTATCAGAAGTATAACCTTCGAGATTCACAAGTTTCTGCCTACACAAGACAGATACT
GCATGGTCTGAAGTATCTCCATGACCGAAATGTGGTTCACAGGGATATTAAATGTGCAA
ATATATTGGTCTCTGCCAGTGGATCCGTTAAGCTTGCAGATTTTGGGTTGGCTAAGGCA
ACCAAATTGAATGATGTTCACTCTTCCAAGGGGACTGCATGCTGGATGGCACCTGAGG
TTGTTAATAGGAGGAACCAAGGATATGGGCTGCCGGCTGATATATGGAGCCTGGGATGC
ACTGTGTTGGAGATGTTAACCAGACAGATCCCGTACTCTGATTTGGAGTGGATGCAGG
CAATATTTCGAATAGGAAAGGGTGTGCTACCTTCTGTTCCTGATTCTCTCTCGAAAGAT
GCACGGGATTTTATCTTGCAGTGCTTGAAAGTTAATCCAAATGATCGTCCTACTGCTTGT
CAGCTGTTAGACCACCCATTTGTGAAGAGACGTCTTTCCTCATCTTCTGGCTCTGCGTC
TCCTTACCATTTTGGGGGGCGGAGTTAA
